# A first-in-class selective inhibitor of ERK1/2 and ERK5 overcomes drug resistance with a single-molecule strategy

**DOI:** 10.1038/s41392-025-02169-z

**Published:** 2025-02-20

**Authors:** Huan Xiao, Aoxue Wang, Wen Shuai, Yuping Qian, Chengyong Wu, Xin Wang, Panpan Yang, Qian Sun, Guan Wang, Liang Ouyang, Qiu Sun

**Affiliations:** 1 State Key Laboratory of Biotherapy and Cancer Center, Innovation Center of Nursing Research, Nursing Key Laboratory of Sichuan Province, West China Hospital, and Collaborative Innovation Center of Biotherapy, Sichuan University, Chengdu, 610041 China; 2 Department of Pathology, Changhai Hospital, Naval Medical University, Shanghai, 200433 China

**Keywords:** Breast cancer, Medicinal chemistry, Breast cancer, Breast cancer

## Abstract

Despite significant advancements in kinase-targeted therapy, the emergence of acquired drug resistance to targets such as KRAS and MEK remains a challenge. Extracellular-regulated kinase 1/2 (ERK1/2), positioned at the terminus of this pathway, is highly conserved and less susceptible to mutations, thereby garnering attention as a crucial therapeutical target. However, attempts to use monotherapies that target ERK1/2 have achieved only limited clinical success, mainly due to the issues of limited efficacy and the emergence of drug resistance. Herein, we present a proof of concept that extracellular-regulated kinase 5 (ERK5) acts as a compensatory pathway after ERK1/2 inhibition in triple-negative breast cancer (TNBC). By utilizing the principle of polypharmacology, we computationally designed **SKLB-D18**, a first-in-class molecule that selectively targets ERK1/2 and ERK5, with nanomolar potency and high specificity for both targets. **SKLB-D18** demonstrated excellent tolerability in mice and demonstrated superior in vivo anti-tumor efficacy, not only exceeding the existing clinical ERK1/2 inhibitor BVD-523, but also the combination regimen of BVD-523 and the ERK5 inhibitor XMD8-92. Mechanistically, we showed that **SKLB-D18**, as an autophagy agonist, played a role in mammalian target of rapamycin (mTOR)/70 ribosomal protein S6 kinase (p70S6K) and nuclear receptor coactivator 4 (NCOA4)-mediated ferroptosis, which may mitigate multidrug resistance.

## Introduction

Precision medicine has ushered in a new era for cancer treatment, with targeted therapy being a key component that has garnered widespread attention among researchers.^1,2^ Due to the crosstalk and feedback networks among multiple pathways in cancer, the current polypharmacology approach increasingly emphasizes the development of clinically effective drugs based on a holistic strategy targeting several key pathways.^3–5^ Developing a single molecule that simultaneously modulates multiple targets in cancer cannot only effectively exert anti-cancer activity and prevent drug resistance but also overcome the potential off-target effects caused by drug combinations, thereby increasing clinical benefits for patients.

Currently, the abnormal activation of RAS-RAF-MEK1/2-ERK1/2 signaling cascade has been confirmed in more than 33% of tumors, and it is considered one of the most important drivers of malignant progression, invasion, and metastasis of tumors.^6–8^ Human ERK1 and ERK2 share about 84% of the same amino acid sequence, and the known upstream stimuli can lead to the parallel activation of ERK1 and ERK2, and most of their functions are redundant, so they are often collectively referred to as ERK1/2. Due to the position of ERKs at the end of the pathway and their low mutation rate, the development of ERK inhibitors has attracted widespread attention from researchers, and has already entered multiple clinical trials.^9–11^ However, due to adverse effects and unsatisfactory efficacy, none of the ERK1/2 inhibitors has been approved for cancer treatment to date. ERK1/2 inhibitors MK-8353,^9^ GDC0994,^12^ CC-90003^13^ and BVD-523^14^ have been proved to accompany with adverse effects in clinical trials, such as interacting with other drugs or affecting the physiological function of normal cells, limiting their long-term use in the clinic.^15^ In addition, cancer cells are highly adaptable and can easily develop acquired resistance in the course of long-term use of ERK1/2 inhibitors. Research has utilized genomic analysis and structural biology to uncover mechanisms leading to resistance against ERK1/2 inhibitors GDC-0994 and SCH772984. The findings indicated that mutations in *ERK1/2*, amplification and overexpression of *ERK2*, as well as overexpression of epithelial growth factor receptor (*EGFR)* and Erb-B2 receptor tyrosine kinase 2 (*ErbB2*), constituted mechanisms of acquired resistance. Furthermore, activation of other signaling pathways such as PI3K-AKT to bypass the inhibited ERK1/2 pathway and promote cell proliferation and survival.^6,16^ ERK1/2 and ERK5 have certain homology (66%) and possess many common substrates, such as c-Myc, c-Fos, RSK, etc., thus participating in the regulation of the same cellular process.^6,7^ The pharmacological inhibition or knockout of ERK1/2 results in compensatory activation and upregulation of p-ERK5, thereby sustaining the proliferation of cell types in melanoma, colorectal cancer, and pancreatic cancer.^17–19^ The compensatory activation of ERK5 has led to limited success in monotherapy attempts at ERK1/2, and there remains a deficiency in developing corresponding polypharmacological therapeutics.^20–22^ Besides, ERK1/2 or ERK5 inhibition has been identified to involved in induction of autophagy and ferroptosis, both of which are associated with reversion of acquired drug resistance in cancer cells.^23–27^ Thus, developing single molecules that simultaneously target ERK1/2/5 to block the ERK5 compensatory activation based on polypharmacology may be an effective strategy to overcome drug resistance.

Our group has already taken a step in this direction by discovering ADTL-EI1712, a dual-target inhibitor of ERK1/5 previously.^28^ In this study, we investigated the crucial roles of ERK1/2 and ERK5 in driving the proliferation and metastatic capabilities of TNBC cells and proposed the therapeutic strategy that simultaneously target ERK1/2 and ERK5 to overcome the ERK5 compensatory activation. We subsequently designed and evaluated a first-in-class single molecule inhibitor against ERK1/2/5 using computer-aided drug design and polypharmacology protocols in order to obtain new drugs that address the current issues of ERK1/2 inhibitors. Our results revealed that **SKLB-D18** monotherapy exhibits significantly superior efficacy in vitro and in vivo against TNBC, compared to ERK1/2 inhibitors or ERK5 inhibitors alone and in combination. In vitro and in vivo data suggested that **SKLB-D18** has greater advantages than the existing ERK1/2 inhibitor BVD-523. Mechanistically, **SKLB-D18** targets ERK1/2/5, and inducing TNBC ferritinophagy. Together, our findings not only provide insights into a novel regulatory mechanism of ERK1/2/5 but also offer a promising therapeutic strategy for overcoming the resistance of ERK1/2 inhibitors.

## Results

### ERK1/2 inhibition induces compensatory ERK5 phosphorylation and promotes TNBC progression

**Fig. 1:**
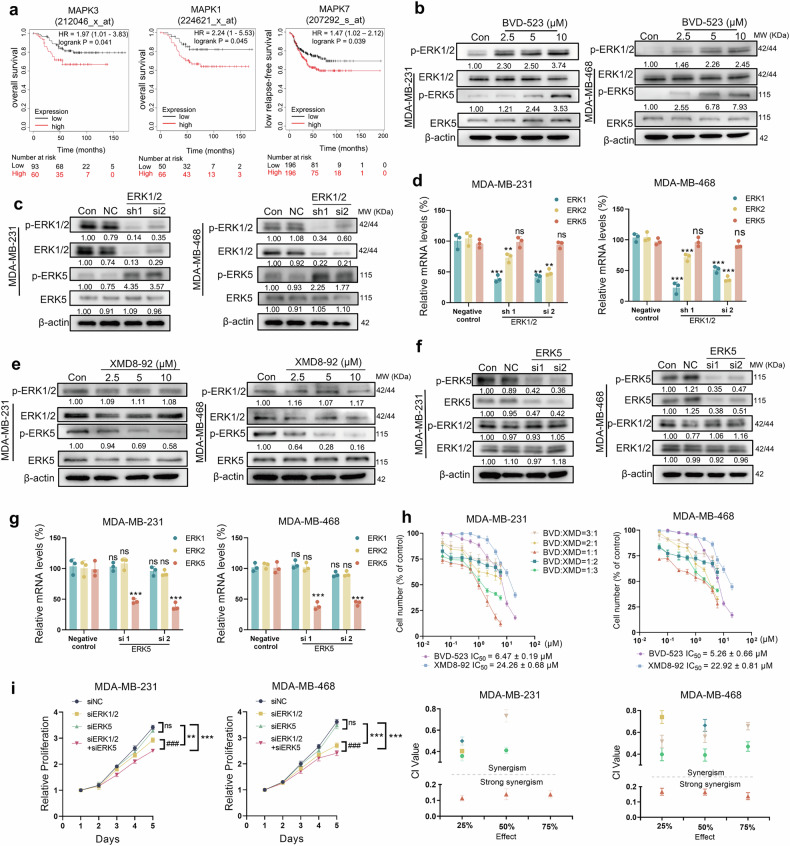
Identification of the biological functions of ERK1/2 and ERK5 in TNBC. **a** Correlation analysis of *MAPK3*, *MAPK1* expression with overall survival and *MAPK7* expression with relapse-free survival of TNBC patients in TCGA database. **b** Immunoblotting analysis of protein levels of ERK5, p-ERK5, ERK1/2, p-ERK1/2 in MDA-MB-231 and MDA-MB-468 cells following treatment with BVD-523 (2.5, 5, 10 μM) for 24 h. **c** Immunoblotting analysis of protein levels of ERK5, p-ERK5, ERK1/2, p-ERK1/2 in MDA-MB-231 and MDA-MB-468 cells transfected with NC or ERK1/2 siRNA for 72 h. **d** Real-time qPCR of *ERK1*, *ERK2*, and *ERK5* mRNA expression levels in cells transfected with NC or *ERK1/2* siRNA for 48 h. **e** Immunoblotting analysis of protein levels of ERK5, p-ERK5, ERK1/2, p-ERK1/2 in MDA-MB-231 and MDA-MB-468 cells following treatment with XMD8-92 (2.5, 5, 10 μM) for 24 h. **f** Immunoblotting analysis of protein levels of ERK5, p-ERK5, ERK1/2, p-ERK1/2 in cells transfected with NC or *ERK5* siRNA for 72 h. **g** Real-time qPCR of *ERK1*, *ERK2*, and *ERK5* mRNA expression levels in MDA-MB-231 and MDA-MB-468 cells transfected with NC or *ERK5* siRNA for 48 h. **h** Cell viability was determined by MTT assay after treatment with BVD-523 and XMD8-92 for 72 h at the indicated concentrations and ratios, and drug combination index of BVD-523 in combination with XMD8-92. **i** MTT assay was performed in cells transfected with ERK1/2, ERK5 and ERK1/2 + ERK5 siRNA, respectively, for 24 to 120 h. Data are presented as Mean ± SD. Compared to the Control group: ns, not significant; **p < 0.01, ***p < 0.001. Com*p*ared to the siERK1/2 group: ^###^*p* < 0.001

ERK1/2 has become a key target for anticancer therapy, yet the compensatory mechanism between ERK1/2 and ERK5 in TNBC remain to be further elucidated.^21,29^ To determine whether ERK1/2 inhibition could induce compensatory activation of ERK5 and thus play an important role in TNBC progression, we firstly investigated whether high expression of ERK1, ERK2 and ERK5 was associated with poor prognosis of TNBC. We found the high expression of *MAPK3* (encoding ERK1) and *MAPK1* (encoding ERK2) was significantly correlated with the low overall survival of TNBC patients, and the high expression of *MAPK7* (encoding ERK5) was significantly correlated with the low relapse-free survival of TNBC patients in the TCGA database (Fig. 1a). ERK1/2 inhibitors have been used in TNBC treatment studies, but have failed to achieve the expected efficacy.^9,10,30^ We further examined ERK5 phosphorylation (p-ERK5) level after ERK1/2 inhibition in MDA-MB-231 and MDA-MB-468 cells. The immunoblotting analysis results demonstrated that pharmacological ERK1/2 inhibition by BVD-523, a specific ERK1/2 inhibitor, significantly increased the phosphorylation of ERK5 in a dose-dependent manner in MDA-MB-231 (KRAS G12D) and MDA-MB-468 cells (KRAS WT) (Fig. 1b). BVD-523 exerts its mechanism by specifically binding to the phosphorylated conformation of ERK1/2, effectively locking ERK1/2 in its phosphorylated active state and sequestering it within the cell nucleus. Following treatment with BVD-523, the downstream substrates of ERK1/2 was downregulated (Supplementary Fig. S1). Furthermore, *ERK1/2* knockdown also induced upregulation of ERK5 phosphorylation (Fig. 1c), which suggested that the ERK5 compensatory activation mechanism was not related to the KRAS mutant status in TNBC. To confirm whether ERK1/2 knockdown could influence ERK5 phosphorylation by upregulate the protein level of ERK5, we analyzed the mRNA level of ERK5 after knocking down *ERK1/2*. It showed that the knockdown of *ERK1/2* did not have impacts on *ERK5* transcription (Fig. 1d). We then examined phospho-ERK1/2 (p-ERK1/2) level after ERK5 inhibition by XMD8-92 and knockdown. On the contrary, the inhibition and knockdown of ERK5 did not increase the ERK1/2 phosphorylation (Fig. 1e, f). Moreover, the knockdown of *ERK5* did not influence the *ERK1/2* transcription (Fig. 1g). We subsequently utilized methyl thiazolyl tetrazolium (MTT) assay to investigate if the co-inhibition of both ERK1/2 and ERK5 could more effectively inhibit the growth and proliferation of TNBC cells. It showed that the combination of BVD-523 and XMD8-92 produced synergistic antiproliferative effects in MDA-MB-231 and MDA-MB-468 cells (Fig. 1h). In particular, when the ratio of BVD-523 and XMD8-92 was 1:1, it showed optimal anti-proliferation activity and the combination index (CI) values were less than 0.2, indicating good synergistic effect. To further explore the role of ERK1/2 and ERK5 in the proliferation of TNBC cells, we utilized 5-Ethynyl-2’-deoxyuridine (EdU) assay and MTT assay to detect proliferative activity of corresponding cells after knockdown of *ERK1/2* and *ERK5*, respectively, and jointly. We found that *ERK1/2* knockdowns alone had a limited effect on cell proliferative activity, while *ERK5* knockdowns had almost no effect. On the contrary, the knockdown of both *ERK1/2* and *ERK5* exhibited significant anti-proliferation activity of MDA-MB-231 and MDA-MB-468 cells, which was consistent with the experimental results of BVD-523 and XMD8-92 inhibitors combined (Figs. 1i, S2a). Subsequently, we utilized wound-healing assay to evaluate the effects of targeted inhibition of both ERK1/2 and ERK5 on cancer cell migration. We found that *ERK1/2* and *ERK5* knockdown showed some anti-migration activity, respectively. The cell wound closure were 18.33% and 22.01% in MDA-MB-231 cells, 21.67% and 21.33% in MDA-MB-468 cells, respectively. In addition, as expected, simultaneous knockdowns of *ERK1/2* and *ERK5* significantly inhibited MDA-MB-231 and MDA-MB-468 cell migration (Supplementary Fig. S2b). These results indicated that simultaneous knockdown of *ERK1/2* and *ERK5* had anti-proliferation and anti-migration activities on TNBC cell lines, suggesting that targeting ERK5 compensatory activation mechanism may be a potentially effective therapeutic strategy for TNBC.

### The inhibition profiles of SKLB-D18 against ERK1/2/5

**Fig. 2:**
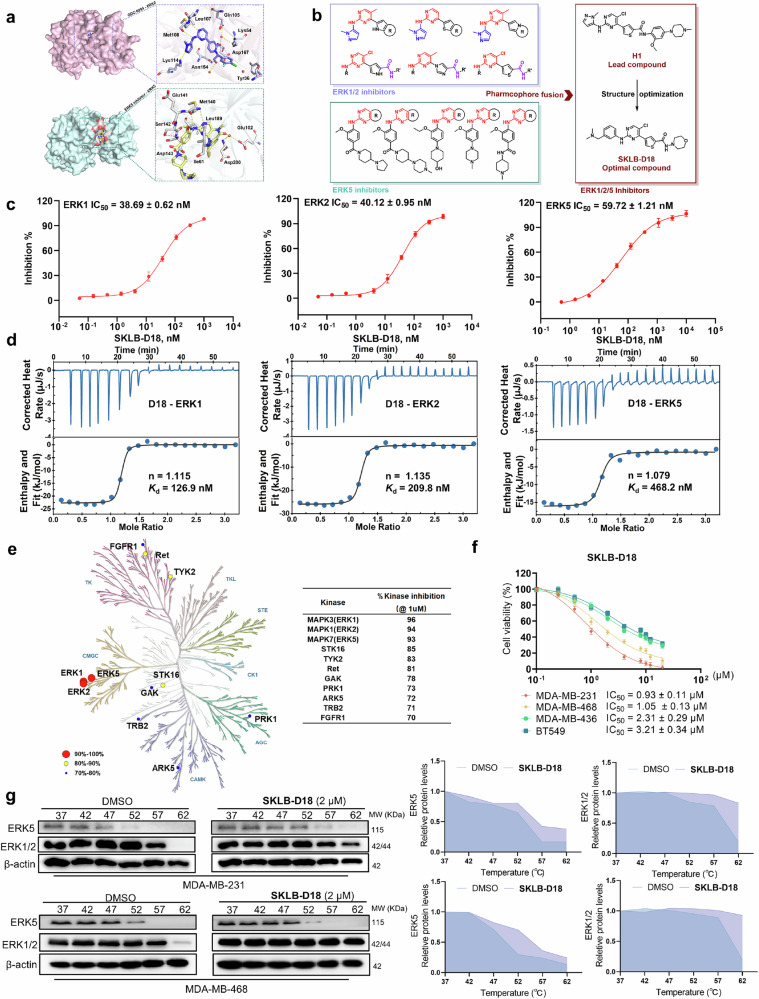
The inhibition profile of **SKLB-D18** against ERK1/2/5. **a** The binding modes of ERK1/2 (PBD code: 5K4I) and ERK5 (PDB code: 4B99) inhibitors with ERK2 and ERK5, respectively. **b** Rational drug design strategy for ERK1/2/5 inhibitors. **c** The inhibitory activity of **SKLB-D18** against ERK1/2/5 is represented as IC_50_ values ± SD in triplicate. **d** The binding affinity of **SKLB-D18** with ERK1/2/5 was validated by ITC assay. **e** The kinase selectivity of **SKLB-D18**. Kinase selectivity analysis was performed on 380 kinases using the DiscoveRx platform. Target inhibition rates exceeding 90%, 80%, and 70% by **SKLB-D18** (1 μM) are indicated by red, yellow, and blue, respectively. **f** The antiproliferative activity of **SKLB-D18** against TNBC cell lines (MDA-MB-231, MDA-MB-468, MDA-MB-436, BT-549) for 72 h was assessed using the MTT assay. **g** CETSA was used to detect the thermostability of **SKLB-D18** (2 μM) to ERK1/2/5 in MDA-MB-231 and MDA-MB-468 cells. After incubating at 37–62 °C (5 °C as the temperature gradient), the expression levels of ERK1/2 and ERK5 in cells were detected

Muti-target inhibitors can overcome resistance associated with dysregulated cell death by addressing target mutations and compensatory pathways. Significant progress in the treatment of diseases has been made with various single-molecule approaches targeting multiple tagets.^31,32^ Here, we aimed to employ a single-molecule approach to identify a novel inhibitor for simultaneously targeting ERK1/2 and ERK5, potentially overcoming resistance to existing ERK1/2 inhibitors. By integrating pharmacophore fusion and computer-aided drug design, we have developed a feasible strategy for the efficient and rational design of ERK1/2/5 inhibitor with superior activity. Initially, we conducted a comprehensive analysis of the key pharmacophores of existing ERK1/2 and ERK5 inhibitors (Fig. 2a). Notably, approximately 80% of ERK1/2 inhibitors entering clinical trials contain a 4-(aromatic heterocyclyl)aminopyrimidine scaffold, highlighting its importance for maintaining ERK1/2 inhibitory activity.^33^ The co-crystal structure of GDC-0994 with ERK2 revealed that the aminopyrimidine forms critical bidentate hydrogen bonds with Met108 in the ATP-binding pocket hinge region (PDB code: 5K4I). Similarly, most ERK5 inhibitors also feature an aminopyrimidine scaffold that interacts with Met140 in hinge region of ERK5 (PDB code: 4B99).^34^ Therefore, we selected the 4-(aromatic heterocyclyl)aminopyrimidine as the core scaffold for developing ERK1/2/5 inhibitors. Our previous studies synthesized a selective ERK1/2 inhibitor **36c** with (thiophen-3-yl)aminopyrimidine scaffold, which exhibited excellent ERK1/2 inhibitory activity.^15^ To maintain potent ERK1/2 inhibitory activity, we retained the (thiophen-3-yl)aminopyrimidine scaffold for subsequent optimization. Additionally, the *N*-methylpyrazole moiety in several ERK1/2 inhibitors was found to interact with Lys114 via hydrogen bonds to exert crucial anti-ERK1/2 activity, thus this moiety was also retained in the lead compound. The methoxyphenyl-aliphatic heterocyclic moiety, commonly found in ERK5 inhibitors, occupied the binding pocket and extends into the solvent region, crucial for binding stability and physicochemical properties. Thus, this moiety was incorporated into the lead compound to enhance ERK5 inhibitory activity and pharmacokinetic properties. Ultimately, we designed and synthesized the lead compound **H1**, which exhibited moderate inhibitory activity against ERK1/2/5 with inhibition rates of 62.45%, 64.20%, and 59.54% at 500 nM, respectively. To further improve its efficacy, we performed structure-based rational drug design to optimize **H1**, resulting in the identification of optimal ERK1/2/5 inhibitor **SKLB-D18** (Fig. 2b). Pivotal structure–activity relationship studies of the compounds targeting ERK1/2/5 are outlined in Supplementary Tables S1–4.

**SKLB-D18** exhibits nanomolar potency against ERK1/2 and ERK5 with half-maximal inhibitory concentration (IC_50_) values of 38.69 ± 0.62 nM, 40.12 ± 0.95 nM and 59.72 ± 1.21 nM, respectively (Fig. 2c). The isothermal titration calorimetry (ITC) assay further confirmed the binding affinity of **SKLB-D18** to ERK1/2/5, with a dissociation constant (*K*_d_) of 126.9 nM, 209.8 nM and 468.2 nM, respectively (Fig. 2d). Subsequent evaluation of kinase selectivity against a broad panel of 380 proteins revealed that **SKLB-D18** has best selectivity for ERK1/2 and ERK5 (Fig. 2e). At 1 µM, only 8 protein kinases outside of the ERK1/2/5 were inhibited by >70%. We performed simultaneous knockdown of *ERK1/2/5* using siRNA co-transfection in MDA-MB-231 and MDA-MB-468 cells respectively. In the corresponding knockdown cell lines, we found that the anti-proliferative activity of **SKLB-D18** was significantly decreased, with IC_50_ values of 14.81 μM and 19.84 μM (Supplementary Fig. S3). Our studies have suggested that simultaneously targeting ERK1/2/5 based on the ERK5 compensatory mechanism may be a potential therapeutic strategy for TNBC. Therefore, we employed the MTT assay to explore the inhibitory activity of **SKLB-D18** against various TNBC cell lines (Fig. 2f). The results demonstrated that **SKLB-D18** inhibits TNBC cells viability in a dose-dependent manner. And **SKLB-D18** showed more potent anti-tumor activity against MDA-MB-231 (IC_50_ = 0.93 ± 0.11 μM) and MDA-MB-468 cells (IC_50_ = 1.05 ± 0.13 μM) than MDA-MB-436 (IC_50_ = 2.31 ± 0.29 μM) and BT549 cells (IC_50_ = 3.21 ± 0.34 μM). To investigate the cellular target engagement of **SKLB-D18**, we performed a cellular thermal shift assay (CETSA) using MDA-MB-231 and MDA-MB-468 cells (Fig. 2g). The results showed an increasing thermal shift for both ERK5 and ERK1/2 proteins after **SKLB-D18** treatment compared to the control group. The results implied that **SKLB-D18** selectively engaged ERK1/2 and ERK5 in intact cells. In summary, **SKLB-D18** was identified as a potent inhibitor simultaneously targeting ERK1/2/5 with excellent binding affinity and kinase selectivity, exhibiting good anti-proliferative activity against MDA-MB-231 and MDA-MB-468 cells, with potential for further investigation.

### Crystal structures of **SKLB-D18** in complex with ERK2/5

**Fig. 3:**
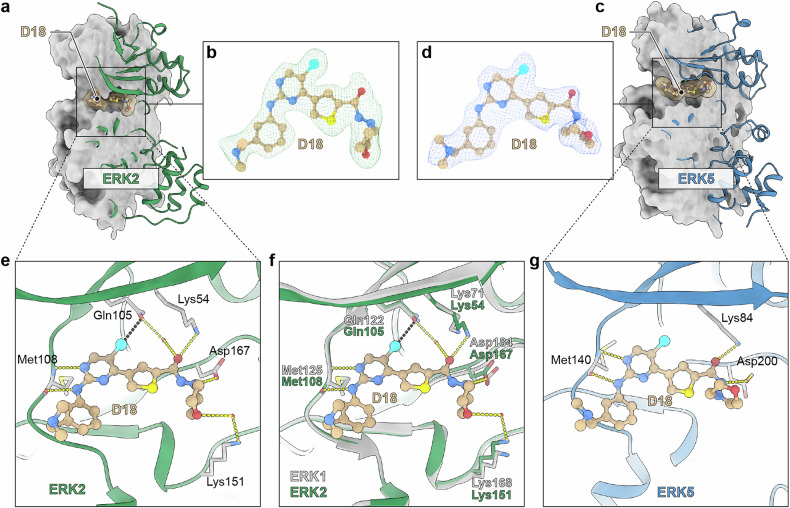
Crystal structures of **SKLB-D18** in complex with ERK2/5. **a**, **c** Overall structure of **SKLB-D18** with ERK2/5. **b**, **d** The electric density map of **SKLB-D18** in crystal structure. **e** Detailed binding mode of **SKLB-D18** with ERK2. **f** Superimposition of ERK1 (PDB code: 6GES) and ERK2 at the binding site. **g** Detailed binding mode of **SKLB-D18** with ERK5. Carbon, nitrogen, oxygen, sulfur, and chlorine atoms are shown in khaki, blue, red, yellow, and cyan, respectively. ERK1/2/5 are shown as green, gray and blue cartoon, respectively. Key interacting residues shown are shown as sticks. Hydrogen bonds and halogen bonds are shown with dashed lines in yellow and black, respectively. Water molecules that mediate hydrogen bonding interaction are shown in red spheres

To elucidate the binding pattern of **SKLB-D18** with ERK1/2/5, we solved the cocrystal structures of ERK2 (Fig. 3a, b) and ERK5 (Fig. 3c, d) in complex with **SKLB-D18** by X-ray crystallography at a resolution of 2.10 and 2.33 Å, respectively (PDB code: 9LNR, 9LTA). X-ray data collection and refinement statistics are detailed in Supplementary Table S5. As shown in Fig. 3e, **SKLB-D18** tightly occupies the ATP-binding sites of ERK2. The 2-aminopyrimidine group is anchored in the ERK2 kinase hinge regions, forming vital bidentate hydrogen bonds with Met108. Additionally, the chlorine atom at the 5-position of pyrimidine forms a halogen bond with the gatekeeper residue Gln105. The carbonyl group on the amide establishes a hydrogen bond with Lys54, while engaging in water-mediated hydrogen bonding with Gln105. The morpholinyl group forms a hydrogen bond with Asp167 via its nitrogen atom, while its oxygen atom participates in additional water-mediated hydrogen bonding with Lys151. Ultimately, the 3-((dimethylamino)methyl)phenyl moiety, extends into the solvent-exposed region, contributing to the enhancement of physicochemical properties. Given the significant homology between ERK1 and ERK2, upon superimposing their crystal structures, we discovered that **SKLB-D18** exhibits a highly analogous binding pattern with both ERK1(PDB code: 6GES) and ERK2 (Fig. 3f). The co-crystal structure of **SKLB-D18** with ERK5 (Fig. 3g) revealed that **SKLB-D18** also occupies the ATP-binding site of ERK5, while a significant bidentate hydrogen bond is established between the 2-aminopyrimidine moiety and Met140. Furthermore, the carbonyl of amide group establishes a hydrogen bond with Lys84, and the NH of amide group forms a water-mediated hydrogen bond with Asp200. Additionally, the morpholinyl and 3-((dimethylamino)methyl)phenyl group are oriented towards the solvent regions on either side, respectively. Structural analysis revealed that the **SKLB-D18** in complex with ERK1/2 establishes a more extensive interaction network compared to its binding with ERK5, as evidenced by (1) additional hydrogen bonds at the binding interface, (2) enhanced hydrophobic complementarity, and (3) optimized electrostatic interactions. These structural features are consistent with the observed higher binding affinity and enzyme inhibitory activity of **SKLB-D18** for ERK1/2 (*K*_d_ = 126.9/209.8 nM, IC_50_ = 38.69/40.12 nM) compared to ERK5 (*K*_d_ = 468.2 nM, IC_50_ = 59.72 nM), as determined by ITC and ADP-Glo Assay, respectively.

### **SKLB-D18** targets the ERK1/2-ERK5 compensatory pathway thereby effectively impeding TNBC cell proliferation and metastasis in vitro

**Fig. 4:**
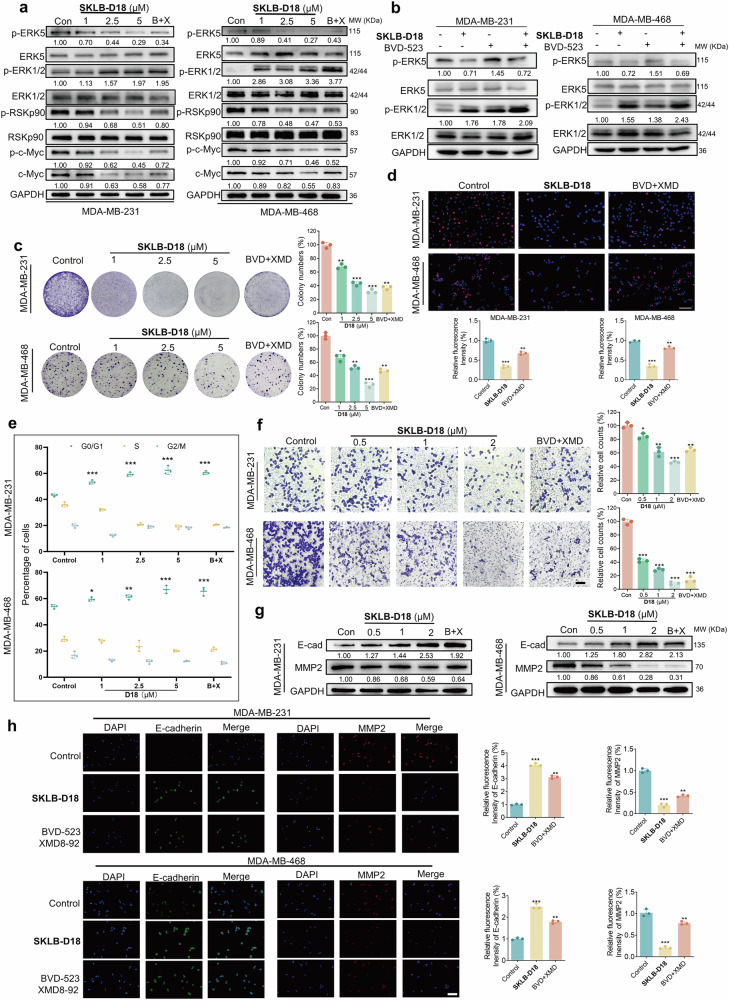
**SKLB-D18** inhibited TNBC cell proliferation and metastasis in vitro. **a** Immunoblotting analysis of protein levels of ERK5, p-ERK5, ERK1/2, p-ERK1/2, p-RSKp90, RSKp90, p-c-Myc, and c-Myc in MDA-MB-231 and MDA-MB-468 cells following 24 h incubation with **SKLB-D18** (1, 2.5, 5 μM), and combination of BVD-523 (5 μM) and XMD8-92 (5 μM). **b** Immunoblotting analysis of p-ERK1/2 and p-ERK5 expression levels following 24 h incubation with **SKLB-D18** (5 μM) in MDA-MB-231 and MDA-MB-468 cells with or without pretreatment of BVD-523 (5 μM). **c** The antiproliferative capacity of cells following incubation with **SKLB-D18** (1, 2.5, 5 μM), and combination of BVD-523 (5 μM) and XMD8-92 (5 μM) by clonogenic assay. **d** Quantification of fluorescence intensity and cell number via EDU staining following 24 h incubation with **SKLB-D18** (5 μM), and combination of BVD-523 (5 μM) and XMD8-92 (5 μM). **e** Flow cytometry analysis of cell cycle arrest following 24 h incubation with **SKLB-D18** (1, 2.5, 5 μM), and combination of BVD-523 (5 μM) and XMD8-92 (5 μM). **f** Transwell assay to quantify the number of cells migrating to the lower chamber following 16 h incubation with **SKLB-D18** (0.5, 1, 2 μM), and combination of BVD-523 (2 μM) and XMD8-92 (2 μM). **g** Immunoblottng analysis of E-cadherin and MMP2 levels following 24 h incubation with **SKLB-D18** (0.5, 1, 2 μM), and combination of BVD-523 (2 μM) and XMD8-92 (2 μM). **h** Immunofluorescence analysis of E-cadherin and MMP2 levels in cells following 24 h incubation with **SKLB-D18** (2 μM), and combination of BVD-523 (2 μM) and XMD8-92 (2 μM). Data are presented as Mean ± SD. Compared to the Control group: *p < 0.05, **p < 0.01, ***p < 0.001

ERK1/2/5 aggregate in the nucleus after activation, activate downstream substrates by regulating transcription factors or performing modifications such as phosphorylation, and play corresponding functions in various physiological activities such as cell survival and proliferation.^6,35^ Therefore, we tested the ability of **SKLB-D18** to inhibit the activation of ERK1/2/5 and its downstream substrate phosphorylation in MDA-MB-231 and MDA-MB-468 cells by immunoblotting, and used the combination of BVD-523 and XMD8-92 in an optimal 1: 1 ratio as a positive control (Fig. 4a). **SKLB-D18** demonstrated superior inhibition of p-ERK5, p-RSKP90, p-c-Myc, and c-Myc at 5 μM compared to the combination group. Consistent with the combination of BVD-523 and XMD8-92, **SKLB-D18** induced the accumulation of p-ERK1/2. Previous studies have reported a dynamic equilibrium between activated ERK1/2 in the open state and inactivated ERK1/2 in the closed state. Some short-chain ATP-competitive inhibitors, such as GDC-0994 and BVD-523, tend to bind to the dually phosphorylated activated state of ERK1/2. This binding type may lead to the accumulation of p-ERK1/2 while inhibiting downstream substrates.^36–38^ We hypothesize that the **SKLB-D18** may exhibit a mode of action similar to that of BVD-523, preferentially binding to the phosphorylated conformation of ERK1/2 and locking it in a dominant conformational state. This prevents the activation of downstream substrates, thereby inhibiting the biological functions of ERK1/2. Subsequently, we investigated whether **SKLB-D18** could inhibit the compensatory activation of ERK5 induced by ERK1/2 inhibitors in cellular models. Immunoblotting results indicated that **SKLB-D18** significantly inhibited the BVD-523-induced upregulation of ERK5 phosphorylation levels across two cell lines (Fig. 4b). Similarly, in *ERK1/2* knockdown cell lines, we observed upregulation of ERK5 phosphorylation in the nucleus, which can be inhibited by **SKLB-D18** (Supplementary Fig. S4). The RAS-RAF-MEKs-ERKs pathway is a classical mitogen-activated protein kinase (MAPK) signaling cascade, where the active state of MEKs directly influences the activity of downstream ERKs.^7^ To further validate that the inhibition of ERK1/2/5 activation by **SKLB-D18** is due to its direct targeting and not due to modulation of upstream signaling pathways, we examined the impact of **SKLB-D18** on the activity of the upstream signaling cascade of ERK1/2 and ERK5. As shown in Supplementary Fig. S5, consistent with the BVD-523 and XMD8-92 combination, **SKLB-D18** did not cause any changes in the activity of upstream MEK1/2 and MEK5 in cells up to 5 μM. This indicated that **SKLB-D18** directly targets ERK1/2/5 to exert its activity.

**SKLB-D18** inhibited colony formation and proliferation of MDA-MB-231 and MDA-MB-468 cells in a dose-dependent manner, with superior efficacy at 5 μM compared to BVD-523 and XMD8-92 combination (Fig. 4c, d). Furthermore, we investigated the effect of **SKLB-D18** on the cell cycle by flow cytometry (Fig. 4e), which indicated that **SKLB-D18** induced cell cycle arrest at the G0/ G1 phase in a dose-dependent manner, thus exerting anti-tumor effects on MDA-MB-231 and MDA-MB-468 cells. In addition, we found that **SKLB-D18** induced apoptosis in a dose-dependent manner (Supplementary Fig. S6). Previous studies have demonstrated that ERK1/2 and ERK5 are involved in cell migration by regulating various substrates.^21,39,40^ We further employed the wound-healing assay and transwell assay to investigate whether **SKLB-D18** has the capacity to inhibit the migration of MDA-MB-231 and MDA-MB-468 cells (Supplementary Fig. S7, Fig. 4f). The results showed that with increasing concentrations of **SKLB-D18**, the cell migration rates and cell numbers in the lower chamber gradually decreased, which was superior to the combination of BVD-523 and XMD8-92 at the same concentration. To further elucidate the molecular mechanism, we examined the expression of E-cadherin and matrix metallopeptidase 2 (MMP2) using immunoblotting and immunofluorescence (Fig. 4g, h). The results revealed that **SKLB-D18** upregulated E-cadherin levels and downregulated MMP2 levels to exert anti-migratory effects in both MDA-MB-231 and MDA-MB-468 cells, outperforming the combination of BVD-523 and XMD8-92 at the same concentration. In conclusion, these results indicated that SKLB-D18 can overcome the ERK1/2-ERK5 compensatory mechanism effectively hindering the proliferation and metastasis of TNBC cells in vitro.

### **SKLB-D18** activates mTOR/p70S6K-regulated intact autophagic flux in TNBC cells

**Fig. 5:**
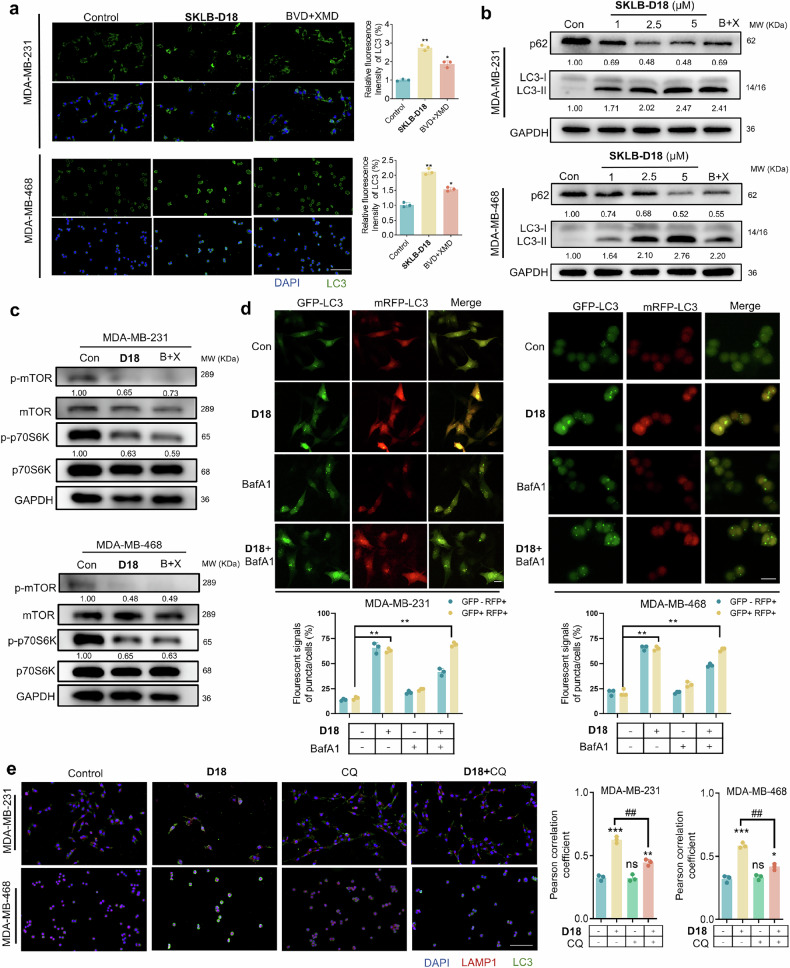
**SKLB-D18** induced complete autophagy in MDA-MB-231 and MDA-MB-468 cells. **a** Immunofluorescence analysis of LC3 levels following treatment with **SKLB-D18** (5 μM) in MDA-MB-231 and MDA-MB-468 cells for 24 h. **b** Immunoblotting analysis of protein levels of p62, LC3I/II in MDA-MB-231 and MDA-MB-468 cells following treatment with **SKLB-D18** (1, 2.5, 5 μM) for 24 h. **c** Immunoblotting analysis of protein levels of p-mTOR, mTOR, p-p70S6K, p70S6K following treatment with **SKLB-D18** (5 μM) and combination of BVD-523 (5 μM) and XMD8-92 (5 μM) for 24 h. **d** Immunofluorescence analysis in cells following co-incubation of **SKLB-D18** (5 µM) and BafA1 (10 nM) after transfecting with GFP-mRFP-LC3 adenovirus. **e** Immunofluorescence analysis of co-localization of LC3 and LAMP1 in cells following co-incubation of **SKLB-D18** (5 µM) and CQ (1 μM). Scale bar, 100 μm. Data are presented as Mean ± SD. Compared to the Control group: ns, not significant; *p < 0.05, **p < 0.01, ***p < 0.001. Compared to the **SKLB-D18** treatment group: ##*p* < 0.01

Autophagy regulation has been served as a promising therapeutic approach, providing targets for the development of anti-TNBC drugs.^41^ It has been reported that ERKs play an important role in the regulation of autophagy.^42–44^ Therefore, we examined whether **SKLB-D18** could regulate autophagy levels to promote cell death. Immunofluorescence results showed that **SKLB-D18** enhanced microtubule-associated protein 1 light chain 3 (LC3) fluorescence intensity, which was consistent with the results of BVD-523 and XMD8-92 combination with ratio of 1:1 (Fig. 5a). We next utilized immunoblotting to evaluate conversion of endogenous LC3B-I to LC3B-II and the level of p62. The results showed that **SKLB-D18** could upregulate the accumulation of LC3B-II and downregulate the p62 level, induce autophagy in TNBC cells (Fig. 5b). mTOR and downstream p70S6K are important proteins of autophagy modulation. Consistent with induced autophagy, we found that consistent with BVD-523 and XMD8-92 combination, targeting of ERK1/2 and ERK5 by **SKLB-D18** inhibited the phosphorylation of mTOR and p70S6K in MDA-MB-231 and MDA-MB-468 cells (Fig. 5c). To identify if **SKLB-D18** induced complete autophagic flux, we evaluated the autophagic flux in TNBC cells transfected with autophagy double-labeled adenovirus (mRFP-GFP-LC3). The treatment of **SKLB-D18** caused increased autophagic flux which could be blocked when autophagosome degradation was inhibited with bafilomycin A1 (Fig. 5d). Furthermore, we assessed the co-localization of LC3 and lysosome-associated membrane proteins-1 (LAMP1) to characterize the fusion of autophagosome and lysosome. We found that **SKLB-D18** lead to continuous co-localization of LC3 and LAMP1 in a time-dependent manner, which could be also blocked by late-stage autophagy inhibitor, chloroquine (CQ) (Supplementary Fig. S8a, Fig. 5e). We also observed upregulated LC3 and LAMP1 level when the co-localization of LC3 and LAMP1 enhanced, suggesting that **SKLB-D18** may enhance the lysosome biogenesis and activity in autophagy and thus affect autophagic lysosome production (Supplementary Fig. S8a). Hence, we tested the intracellular lysosome activity with Lyso-Tracker Red probes. It showed that consistent with the combination of BVD-523 and XMD8-92, **SKLB-D18** significantly enhanced the lysosome activity (Supplementary Fig. S8b). These results indicated that **SKLB-D18** upregulated autophagy and induced autophagy-dependent cell death in TNBC.

### **SKLB-D18** induces ferritinophagy of TNBC cells and promotes cell death

**Fig. 6:**
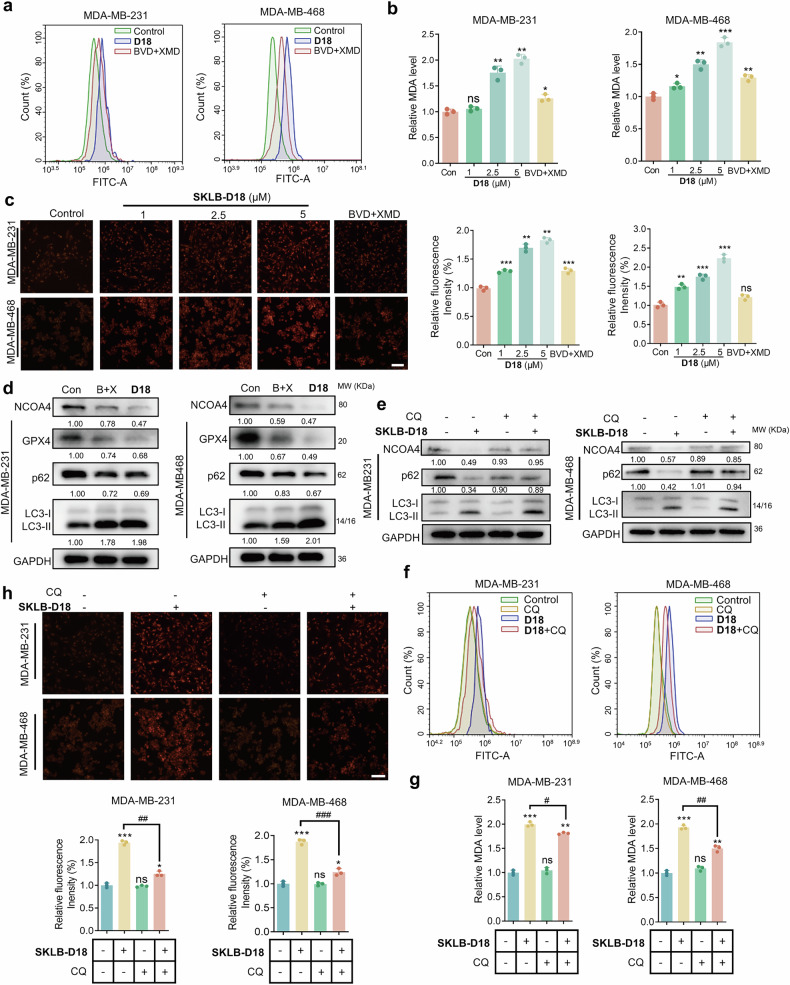
**SKLB-D18** induced ferritinophagy in MDA-MB-231 and MDA-MB-468 cells. **a** Flow cytometry was used to evaluate the ROS levels after treatment of **SKLB-D18** (5 μM) and combination of BVD-523 (5 μM) and XMD9-82 (5 μM). **b** MDA levels were detected after 24 h treatment of **SKLB-D18** (1, 2.5, 5 μM) and combination of BVD-523 (5 μM) and XMD9-82 (5 μM). **c** Immunofluorescence analysis of free ferrous ions levels by FerroOrange probe after 24 h treatment of **SKLB-D18** (1, 2.5, 5 μM) and combination of BVD-523 (5 μM) and XMD9-82 (5 μM). **d** Immunoblotting analysis of protein levels of NCOA4, GPX4, p62 and LC3I/II following treatment with **SKLB-D18** (5 μM) and combination of BVD-523 (5 μM) and XMD9-82 (5 μM) for 24 h. **e** Immunoblotting analysis of protein levels of NCOA4, p62 and LC3I/II after 24 h co-incubation of SKLB-D18 (5 μM) and CQ (1 μM). **f** Flow cytometry was used to evaluate the ROS levels after 24 h co-incubation of SKLB-D18 (5 μM) and CQ (1 μM). **g** MDA levels were detected after 24 h co-incubation of SKLB-D18 (5 μM) and CQ (10 μM). **h** Immunofluorescence analysis of free ferrous ions levels by FerroOrange probe after 24 h co-incubation of SKLB-D18 (5 μM) and CQ (1 μM). Scale bar, 100 μm. Data are presented as Mean ± SD. Compared to the Control group: ns, not significant; *p < 0.05, **p < 0.01, ***p < 0.001. Compared to the **SKLB-D18** treatment group: #*p* < 0.05, ##*p* < 0.01, ###*p* < 0.001

Ferroptosis is the regulated necrosis driven by iron-dependent lipid peroxidation of polyunsaturated-fatty-acid in cell membranes or organelle membranes, involving the onset of oxidative stress and energy stress responses. It has been reported that MAPK signaling pathway acts as an important regulator of oxidative stress, which could further influence the ferroptosis. Thus, we first examined the effect of **SKLB-D18** on intracellular reactive oxygen species (ROS) level using a DCFH-DA probe. **SKLB-D18** enhanced ROS levels more significantly compared with the combination of BVD-523 and XMD8-92, suggesting potential intracellular lipid peroxidation (Fig. 6a). Subsequently, we assessed the Malondialdehyde (MDA) level after the treatment of a series of concentration gradients of **SKLB-D18**. When cells undergo oxidative stress, some fatty acids decompose gradually after oxidation. As one of the decomposition products, MDA could indirectly reflect the lipid oxidation level of cells. We found that **SKLB-D18** increased intracellular MDA level in a dose-dependent manner (Fig. 6b). Utilizing FerroOrange probe, we then analyzed ferrous ions level upon treatment of **SKLB-D18**. As expected, with the increase of the concentration of **SKLB-D18**, the fluorescence intensity of ferrous ions gradually increased, indicating that **SKLB-D18** induced the increase of free ferrous ions in MDA-MB-231 cells and MDA-MB-468 cells (Fig. 6c). To verify the mechanism of **SKLB-D18** regulating ferroptosis in TNBC, we performed immunoblotting to analyze the variation of key proteins in process of ferroptosis, including glutathione peroxidase 4 (GPX4) and NCOA4. The results showed that **SKLB-D18** downregulate the content of GPX4 and NCOA4. The process was synchronized with enhanced autophagy, suggesting ferroptosis induced by **SKLB-D18** may related to autophagy (Fig. 6d). Ferritinophagy generally refers to an autophagy process in which cells selectively degrade the iron-storing protein ferritin heavy polypeptide 1 through the autophagy pathway to regulate iron metabolism and activate iron death by up-regulating the content of free ferrous ions. In this process, NCOA4, a marker of iron autophagy, plays a key role as a specific autophagy receptor. Therefore, we first investigated whether the changes in intracellular NCOA4 levels induced by **SKLB-D18** were regulated by autophagy through the introduction of CQ. By inhibiting autophagy lysosome degradation with CQ, NCOA4 downregulation was rescued with late inhibition of autophagy, suggesting that autophagy degradation mediates the downregulation of NCOA4 induced by **SKLB-D18** (Fig. 6e). Subsequently, we investigated whether inhibition of autophagolysosomal degradation could affect the intracellular ROS accumulation induced by the **SKLB-D18**. Inhibition of autophagy degradation by CQ significantly downregulated the increase of ROS content induced by **SKLB-D18**, suggesting that autophagy induced by **SKLB-D18** was involved in intracellular ROS accumulation (Fig. 6f). Next, we further explored whether autophagy is involved in the increase in MDA levels during ferroptosis. **SKLB-D18** upregulated MDA levels in MDA-MB-231 and MDA-MB-468 cells, and this effect was significantly weakened by the inhibition of autophagy degradation induced by CQ (Fig. 6g). Moreover, inhibition of autophagolysosomal fusion by CQ significantly downregulated the level of free ferrous ions induced by **SKLB-D18** (Fig. 6h). Hence, we concluded that **SKLB-D18** involved in regulating the intracellular iron metabolism pathway by inducing NCOA4-mediated selective autophagy and promote ferroptosis in TNBC cells.

### **SKLB-D18** monotherapy retards tumor growth in TNBC xenograft mouse models

To further evaluate the antitumor efficacy of **SKLB-D18** in vivo, we firstly conducted preliminary evaluations of its druggability through studies on liver microsome metabolism, plasma protein binding rate, and transport characteristics in the Caco-2 cell monolayer model. **SKLB-D18** exhibits a biological half-life (T_1/2_) of 32.52 min and a clearance rate (CL_int_) of 0.0426 mL/min/mg in liver microsomes of SD rats. Meanwhile, in human liver microsomes, the T_1/2_ is 91.61 min with CL_int_ of 0.0151 mL/min/mg (Supplementary Table S6). These results indicate that **SKLB-D18** demonstrates moderate liver microsome metabolic stability in humans, which is superior to its stability in rats. The plasma protein binding rates of **SKLB-D18** was 18.41 ± 0.37%. Overall, the relatively low plasma protein binding rate suggested that **SKLB-D18** may predominantly exist in its free form in vivo, facilitating rapid distribution to exert its pharmacological effects. The results of the Caco-2 cell transport study indicated that **SKLB-D18** possessed favorable intestinal transport properties, with an apparent permeability coefficient (*P*_app_) of 2.232 × 10^−6 ^cm/s and a low efflux ratio of 13.7%, suggesting good oral bioavailability and suitability for oral administration (Supplementary Table S7). Subsequently, we investigated the pharmacokinetic characteristics of **SKLB-D18** in SD rats through intravenous injection (iv, 1 mg/kg) and intragastric administration (po, 10 mg/kg) (Supplementary Table S8). Following intragastric administration, the maximum plasma concentration (C_max_) of **SKLB-D18** was 53.71 ng/mL, comparable to that achieved via intravenous injection. The area under the plasma concentration-time curve (AUC_0-∞_ and AUC_0-t_) were 456.92 ng∙h/mL and 303.93 ng∙h/mL, respectively, indicating higher total absorption and drug exposure levels post intragastric administration compared to intravenous injection. Additionally, **SKLB-D18** exhibited a longer half-life (T_1/2_) of 3.71 h following intragastric administration, with the oral bioavailability (*F*) of 22.42%. These experimental results collectively suggested that **SKLB-D18** has acceptable pharmacokinetic properties, indicating potential for in vivo anti-tumor efficacy evaluation via intragastric administration.

**Fig. 7:**
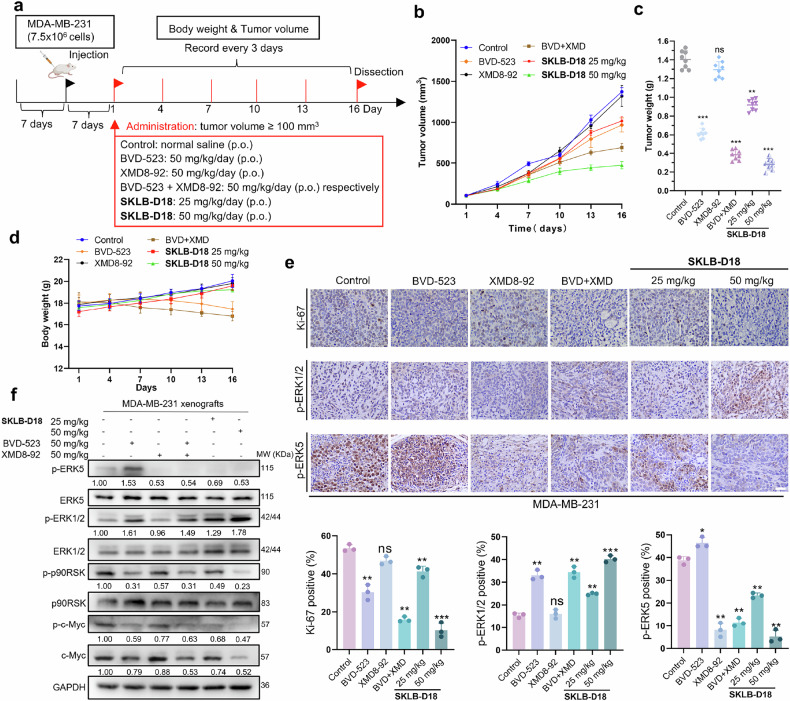
**SKLB-D18** monotherapy retards tumor growth in MDA-MB-231 xenograft model. **a** Establishment of the MDA-MB-231 xenograft model and the treatment regimen for the in vivo study. **b** Tumors volume growth curves in each group during the treatment period. **c** Tumors weight of each group. **d** Changes in body weight of nude mice in each group during the treatment period. **e** Immunohistochemical analysis of Ki-67, p-ERK1/2, and p-ERK5 in MDA-MB-231 xenograft tumor tissues in each group. Scale bar, 40 μm. **f** The expression levels of ERK1/2, p-ERK1/2, ERK5, p-ERK5, p90RSK, p-p90RSK, p-c-Myc, and c-Myc were analyzed by immunoblotting. Data are presented as Mean ± SD. Compared to the Control group: ns, not significant; *p < 0.05, **p < 0.01, ***p < 0.001

We next established a TNBC xenograft tumor model using MDA-MB-231 cells and administered **SKLB-D18** orally at doses of 25 mg/kg and 50 mg/kg daily for 16 days (Fig. 7a). BVD-523 (50 mg/kg) and XMD8-92 (50 mg/kg) monotherapy and combination therapy by intragastric administration were served as positive controls. **SKLB-D18** dose-dependently inhibited tumor growth in the TNBC xenograft model, with a tumor inhibition rate of 79.88% at 50 mg/kg dose, which was superior to the combination treatment of BVD-523 and XMD8-92 (72.66%). The monotherapies of BVD-523 and XMD8-92 exhibited lower tumor inhibition rates of 36.01% and 7.75%, respectively (Fig. 7b, c). No significant weight loss or adverse effects were observed in the **SKLB-D18** treatment groups (Fig. 7d). Furthermore, Hematoxylin Eosin (H&E) staining results indicated no significant toxicity for **SKLB-D18** to major organs (Supplementary Fig. S9). To further elucidate the anti-tumor mechanism of **SKLB-D18** in vivo, we employed immunohistochemistry (IHC) and immunoblotting to detect the expression of relevant proteins in tumor tissues (Fig. 7e, f). The proliferation marker Ki-67 is a crucial indicator of cancer cell viability. IHC results showed that over 50% of tumor tissue in the control group was Ki-67 positive. The **SKLB-D18**-treated groups demonstrated a dose-dependent decrease in Ki-67 positive, with less than 20% Ki-67 positive area in the 50 mg/kg **SKLB-D18**, indicating superior anti-tumor activity compared to the combination therapy of BVD-523 and XMD8-92. Both IHC and immunoblotting experiments showed that **SKLB-D18** treatment increased p-ERK1/2 levels in tumor tissues in a dose-dependent manner. The 50 mg/kg **SKLB-D18** group had higher p-ERK1/2 levels than BVD-523 and combination therapy groups, aligning with in vitro results. BVD-523 increased p-ERK5 expression, indicating ERK5 compensatory activation in BVD-523-treated TNBC xenografts. **SKLB-D18** dose-dependently inhibited p-ERK5, with the 50 mg/kg dose showing stronger inhibition than XMD8-92 and combination therapy groups. In conclusion, **SKLB-D18** has acceptable pharmacokinetic properties, targeting ERK1/2/5 and blocking ERK5 compensatory activation. Collectively, above in vivo data suggest that **SKLB-D18** may be better tolerated compared to previous ERK1/2 inhibitor BVD-523, and show anti-tumor efficacy exceeding the monotherapies and combination therapy of BVD-523 and XMD8-92, warranting further investigation as an oral anti-tumor agent.

## Discussion

Studies have indicated that targeting the ERK1/2 pathway could serve as a potential therapeutic strategy for TNBC. However, the application of corresponding inhibitors has not demonstrated the desired therapeutic effects. The emergence of acquired drug resistance means that kinase-targeted drugs rarely have sustained monotherapy activity. Studies have shown that the inhibition of ERK1/2 activity leads to the phosphorylation and activation of the ERK5 pathway in various cancers, promoting the malignant proliferation of cancer cells, which may be a potential key reason why existing ERK1/2-targeted inhibitors have not achieved the expected effects. In this study, our research demonstrated the potential of simultaneously targeting ERK1/2 and ERK5 in anti-tumor therapy. **SKLB-D18** exhibited superior anti-tumor activity over the clinical ERK1/2 inhibitor BVD-523 by inhibiting ERK1/2 activity and ERK5-driven resistance, providing a potential clinical monotherapy strategy to overcome resistance encountered in targeted ERK1/2 therapy for TNBC.

Based on these findings, we used computer-aided drug design and pharmacophore fusion strategy to obtain a single-molecule inhibitor against ERK1/2 and ERK5, named **SKLB-D18**. The co-crystal structures revealed that **SKLB-D18** occupies the ATP-binding sites of both ERK2 and ERK5 simultaneously. Meanwhile, the structural alignment showed that the binding mode of **SKLB-D18** to ERK1 is similar to that of ERK2. The bidentate hydrogen bonds formed between the 2-aminopyrimidine scaffold and ERK1/2/5 are crucial for the stable binding. Additionally, several hydrogen bonds, halogen bonds are also established. These multiple interaction networks collectively enhanced the binding affinity and selectivity of **SKLB-D18** towards ERK1/2/5. Evaluation of kinase selectivity against a broad panel of 380 proteins revealed that **SKLB-D18** is bestly selective for ERK1/2 and ERK5. At 1 µM, only 8 of 380 kinases outside of the ERK1/2/5 were inhibited by >70%.

The successful discovery of **SKLB-D18** highlighted the potential of pharmacophore fusion, computer-aided drug design and structure-based optimization strategies in drug discovery, particularly in the development of multi-target inhibitors. In pharmacodynamic evaluations, **SKLB-D18** exhibited potent antiproliferative activity across various TNBC cell lines, with particularly pronounced effects in MDA-MB-231 and MDA-MB-468 cells. **SKLB-D18** not only exhibited highly selective inhibition of ERK1/2 and ERK5 but also demonstrated more significant anti-tumor activity than clinical ERK1/2 inhibitor BVD-523 in vivo assays, and possessed acceptable pharmacokinetic properties.

**SKLB-D18** could target ERK1/2/5 to overcome drug resistance caused by ERK5 compensation and inhibit the migration and proliferation of TNBC. **SKLB-D18** activated autophagy by regulating the mTOR/p70S6K signaling pathway and induced NCOA4-mediated ferritinophagy. Cancer cells may enhance the defenses against oxidative stress by negatively regulating the ferroptosis, leading to the drug resistance. Therefore, **SKLB-D18** may exert anti-tumor activity and reverse the drug resistance by inducing ferroptosis.^24^ In addition, we found that it induced downregulation of platelet-derived growth factor receptors β (PDGFR-β), which was potentially associated with compensatory phosphorylation activation of ERK5 after ERK1/2 inhibition.^17^ Therefore, we speculated that **SKLB-D18** could exert enhanced dual inhibition of ERK5 phosphorylation through direct inhibition of ERK5 and autophagic degradation of PDGFR-β, thus exerting excellent anti-drug resistance and anti-tumor activity (Supplementary Fig. S10).

Despite **SKLB-D18** showing good application potential in preliminary studies, there are still some issues that require further research. For instance, whether it will induce new resistance mechanisms during long-term administration and its metabolic pathways in the complex in vivo environment are not yet fully clear. In addition, the toxicity assessment and long-term safety studies of **SKLB-D18** are also key for the next phase of research to ensure its safety and efficacy in clinical application. We will further explore the combination therapy strategies of **SKLB-D18** with other anti-tumor drugs and its adaptability in different types of tumors, especially its potential in overcoming resistance to existing ERK1/2 and ERK5 inhibitors. At the same time, structural optimization can be considered to enhance the liver metabolic stability of **SKLB-D18** to extend its duration of action in the body. We will further optimize it as a lead compound to obtain candidates with better druggability and stronger anti-tumor activity.

In summary, our research has revealed the existence of an ERK1/2-ERK5 compensatory mechanism in TNBC and found that simultaneous inhibition of ERK1/2/5 is superior to the inhibition of ERK1/2 or ERK5 alone in exhibiting better in vitro anti-TNBC proliferation and anti-migration activity. **SKLB-D18** has achieved precise regulation of multiple signaling pathways in the form of a single molecule, demonstrating significant anti-TNBC activity, providing theoretical and experimental support for the potential application of single-molecule targeting of the ERK5 compensatory mechanism in cancer therapy. The experimental results suggest that **SKLB-D18** has the potential to confer resistance to clinical ERK1/2 inhibitors by overcoming compensatory mechanisms. As such, **SKLB-D18** emerges as a valuable tool compound and chemical probe for in-depth exploration of the biological interplay and compensatory mechanisms between ERK1/2 and ERK5, paving the way for the development of more potent, less toxic, and less susceptible-to-resistance ERK inhibitors. Furthermore, with further optimization and development, **SKLB-D18** holds promise as an antitumor agent with improved druggability, offering novel therapeutic possibilities for TNBC. Furthermore, based on previous studies and mechanistic investigations, it is speculated that **SKLB-D18** may also exert efficacious effects in other tumor types, such as melanoma^17,45^^,46^, colorectal cancer (CRC)^18^, and pancreatic ductal adenocarcinoma (PDAC)^19^. This will be further explored in our future research endeavors.

## Materials and methods

### Bioinformatics analysis

Kaplan-Meier plotter was used to analyze the correlation between genes in the TNBC database and patient survival. Select “mRNA gene - start KM plotter for breast cancer” on the analysis platform, input *MAPK1*, *MAPK3*, and *MAPK7*, respectively, select automatic optimal, and screen TNBC samples by setting ER, PR and HER2 negative. The overall survival time and disease-free survival time were selected for analysis.

### Cell culture

MDA-MB-231, MDA-MB-468, MDA-MB-436, and BT549 cells were purchased from American Type Culture Collection. MDA-MB-468 and MDA-MB-436 cells were cultured in Leibovitz’s L-15 medium supplemented with 10% fetal bovine serum. MDA-MB-231 and BT549 cells were maintained in Dulbecco’s modified Eagle’s medium containing 10% fetal bovine serum. All of the cells were cultured at 37 °C in a humidified atmosphere containing 5% CO_2_.

### Immunoblotting analysis

MDA-MB-231 and MDA-MB-468 cells were starved for 24 h in a six-well plate, treated with a series of concentrations of **SKLB-D18**, BVD-523, and XMD8-92. Then, the cells were washed twice with PBS and stimulated with cell lysis buffer. After 13,000 rpm centrifugation for 20 min, an enhanced bicinchoninic acid (BCA) protein assay kit was (Beyotime Biotechnology, Jiangsu, China) used to quantify the protein level of the supernatural. After being separated by 12.5% sodium dodecyl sulfate-polyacrylamide gel electrophoresis (SDS-PAGE), the equivalent concentrations of total proteins were transferred to nitrocellulose membranes. Furthermore, membranes were blocked with 5% skim milk. The membranes were incubated by the corresponding primary antibodies. After being washed twice with TBST solution, the membranes were incubated with horseradish peroxidase (HRP)-conjugated secondary antibodies and visualized by employing ECL (electrochemiluminescence) as the HRP substrate.

Tumor tissues were lysed with RIPA supplemented with protease and phosphatase inhibitor cocktail (Beyotime Biotechnology, Jiangsu, China). Protein concentrations were determined using an enhanced BCA protein assay kit for normalization of the samples. Equal amounts of protein were loaded on SDS-PAGE gels for blotting. Finally, statistical analysis was quantified using Image Lab.

### Transient transfection

MDA-MB-231 and MDA-MB-468 cells were seeded in a 6-well plate at an 80% confluence the day before and transfected with atotal amount of 500 ng of sh*MAPK1/3*, 5 μg of si*MAPK1*/*3*, si*MAPK7* plasmid/well, respectively. The transient transfection processes were performed according to Lipofectamine 3000 transfection procedure details. The transfected cells were used for subsequent experiments after 48 h or 72 h.

sh*MAPK1/3* was obtained from Corues BiotechnologyCo. LTD, China: pRNA3-SV40-EGFP(2A)Puro-U6-ERK1/2-i1; interference site: AGCAATGACCATATCTGCTA.

Sequence of si*MAPK1/3*:

GGACCGGAUGUUAACCUUU(dT)(dT); AAAGGUUAACAUCCGGUCC(dT)(dT)

Sequence of si*MAPK7*:

GCGAAUUCAAAUCUGUCUA(dT)(dT); UAGACAGAUUUGAAUUCGC(dT)(dT)

GGCUGUCCAGAUGUUGAAA(dT)(dT); UUUCAACAUCUGGACAGCC(dT)(dT)

### Fluorescent quantitative PCR

Commercial RNA extraction kit was used to extract RNA from cell samples, and the corresponding cDNA template was obtained by PCR reverse transcription. Then real-time fluorescence quantitative PCR was performed by sybr green dye method. Each sample was required to detect the target genome and internal reference genome, and 3 replicates were set for each sample. In the system, the upstream and downstream primers were 0.5 μL, the cDNA template was 1 μL, the sybr green dye was 4 μL, and sterile, enzyme-free water was 5 μL. The reaction system was as follows: wait time was 10 s, PCR amplification temperature was 95 °C for 10 s, annealing and extension temperature was 60 °C for 20 s, and 40 cycles were performed. The final results were analyzed and quantified by the Design and Analysis PCR program according to the RNA levels of the tested genes by the method of ΔΔCT.

### Cell viability assay

Cancer cells were seeded in a density in 96-well plates (2 × 10^3^ cells per well). After incubation for 24 h, cancer cells were treated with compounds (0–20 μM) for 72 h. The antiproliferative activities of the respective compounds were evaluated with MDA-MB-231, MDA-MB-468, MDA-MB-436, and BT549 cells by MTT assay, as previously described.

### EdU proliferative activity detection

MDA-MB-231 and MDA-MB-468 cells were uniformly inoculated into 24-well plates placed on sterile slides, cultured for 24 h, and treated with a series of compounds of specified concentration for 24 h. Discard the medium, wash with PBS and replace with normal medium. EdU working liquid incubated at 37 °C was added and mixed to make the final concentration of 10 μM, and cultured for 2 h. The cells were then fixed with 4% paraformaldehyde and permeated with 0.2% Triton X on the ice surface for 3 min, then discarded and washed with PBS in a horizontal shaker for 3 times for 3 min each time. After cleaning, add 0.5 ml Click reaction solution to each well and incubate at room temperature for 30 min away from light, then clean again. Add 500 μL Hoechst 33342 staining solution to each well and incubate for 10 min at room temperature and avoid light. After incubation, wash with PBS 3 times for 5 min each time to remove specific binding. The anti-fluorescence quencher was added to the slide and analyzed with a positive fluorescence microscope after sealing.

### Wound-healing assay

The cells were cultured in a six-well plate, and the cell surface was scratch-wounded using sterilized pipettes. Then, the cells were washed with phosphate-buffered saline (PBS) and treated with **SKLB-D18** (0.5, 1, 2 μM), BVD-523 (2 μM), XMD8-92 (2 μM) or normal medium. After incubation for 24 h, images were taken using an Opera Phenix Plus High-Content Screening System (PerkinElmer).

### Synthesis of compounds

The synthesis scheme and structural characterization for compounds are included in the Supplementary Information.

### Protein expression and purification

The full-length human ERK1 or ERK2 with His-tag was cloned into a pET28a vector. hERK1/2 were expressed in *Escherichia coli* BL21(DE3), and then were purified using sequential Ni-NTA and Superdex 200 column steps. The SEC purified sample was then concentrated to 10 mg/ml and stored at −80 °C with buffer containing 20 mM HEPES pH 7.5, 150 mM NaCl. ERK5 (residues 48–395) containing His and GST tags was cloned into the pFastBac vector, and the recombinant plasmid was extracted. The recombinant plasmid was transformed into the DH10bac strain, and monoclonal was selected for culture. The recombinant Bacmid was then extracted according to the instructions of the Baculovirus Shuttle Vector Bacmid Mini Preparation Kit (Beyotime) and validated by PCR. 1 × 10^6^/well Sf9 cells were counted for transfection and then amplified to obtain P3 ERK5 recombinant baculovirus. It was added to 2 × 10^6^/mL Sf9 cells and incubated at 27 °C for 72 h on a shaker to express ERK5. Cells were lysed and supernatant was collected on a Ni-NTA for purification, and a 250 mM imidazole eluate was collected. Next, digestion by TEV protease was performed overnight while dialysis was performed in approximately 1000 mL of imidazole-free buffer. The digested ERK5 protein was sequentially purified on the Ni-NTA and GST-tag Purification Resin, followed by size exclusion chromatography on a Superdex 75 column. ERK5 protein was collected and concentrated to 12 mg/ml and stored at −80 °C in buffer containing 50 mM HEPES pH 8.0, 200 mM NaCl, 10% (v/v) glycerol.

### Crystallization and structure determination

ERK2 (9 mg/mL) and ERK5 (8 mg/mL) was incubated with **SKLB-D18** in ratio of 1:5 for 2 h on ice. The samples were the clarified by centrifugation (10 min, 12,000 × *g*, 4 °C). The ERK2 crystals was grown in 1 μL of protein and 1 μL of reservoir solution comprising 0.1 M MES pH 7.5, 25% PEG MME 2000, 0.2 M ammonium sulfate, 0.02 M Dithiothreitol (DTT) through the hanging-drop vapor diffusion method at 20 °C. The seed stock was prepared by transferring the ERK2 crystals into a centrifuge tube containing a stabilization buffer, and the crystals were crushed by Vortex Mixer. Drops were immediately seeded with the seed stock. The ERK2 crystals usually reached their optimal size in eight days. High-quality ERK5 crystals was grown in 1 μL of protein and 1 μL of reservoir solution comprising 0.1 M HEPES 7.5, 10% PEG4000, 7% isopropanol at 4 °C. For data collection, the ERK2/5 crystals were transferred to freezing buffer (reservoir solution containing 20% glycerol) and rapidly frozen in liquid nitrogen. Diffraction data were collected on the BL19U1 beamlines of the Shanghai Synchrotron Radiation Facility (SSRF). All data were integrated with X-ray detector software (XDS) and scaled with Aimless. Crystal structures of ERK2/5 were determined by molecular replacement with Phaser MR in CCP4 using the previously published complex structures of ERK2/5 with inhibitors (PDB code: 3I5Z, 6HKM) as the model. Model building and refinement were performed with COOT and PHENIX software, respectively. Data processing and refinement statistics are summarized in Supplementary Table S5.

### ERK1/2/5 inhibitory assays

ERK1/2/5 kinase activity assay was conducted using ADP-Glo™ Kinase Assay. 2× ATP solution and 2× kinase & Metal solution were prepared using assay buffer were prepared using assay buffer containing 50 mM Hepes pH 7.5, 10 mM MgCl_2_; 0.01% Brij35, 1 mM EGTA, 2 mM DTT. Transfer 40 nL compounds to 384 assay plate. Add 2 μL of 2× kinase & Metal solution were mixed and incubated in a 384-assay plate for 10 min at 25 °C. 2 μL of 2×ATP solution was added and incubated at 25 °C for 60 min. And then, each well was supplemented with 4 µL of ADP-Glo^TM^ reagent and incubated at 25 °C for 40 min. Subsequently, 8 µL of Kinase Detection Reagent was added and incubated at 25 °C for 40 min. Finally, the luminescence signals were recorded. The IC_50_ values were calculated using GraphPad Prism 9.5 software.

### Kinase profiling

380 kinases inhibitory activities of **SKLB-D18** were conducted by Eurofins Cerep SA. The result was illustrated by kinase tree (http://www.kinhub.org/kinmap/).

### Isothermal titration calorimetry (ITC) assay

Calorimetric experiments were carried on an Affinity ITC (Waters, Shanghai) with 1 mM **SKLB-D18** and 0.1 mM ERK1/2/5. Experiments were carried out at 25 °C while stirring at 125 rpm in ITC buffer. All injections were performed using a 20 injection of 2.5 μL with a spacing of 200 s between injections. The data were analyzed with the NanoAnalyze software employing a single binding site model. A blank control for the 1 mM **SKLB-D18** titration buffer was subtracted. The first data point was excluded from the analysis.

### Cellular thermal shift assay

MDA-MB-231 and MDA-MB-468 cells were incubated with DMSO or **SKLB-D18** (2 μM) for 2 h. Cells were collected by trypsin and then resuspended in PBS. Following heating at the indicated temperatures (37, 42, 47, 52, 57, and 62 °C) for 3 min, cell suspensions were lysed by three freeze−thaw cycles with liquid nitrogen and thawed at room temperature. The lysates were separated by centrifugation at 17,000 × *g* for 20 min. The supernatants were collected and prepared for Immunoblotting to detect the stability of ERK1/2 and ERK5.

### Clonogenicity assay

To determine the proliferation potential of cells treated with **SKLB-D18**, BVD-523 and XMD8-92, MDA-MB-231 cells (1 × 10^3^ cells/well), MDA-MB-468 cells (1.2 × 10^3^ cells/well) were seeded in a six-well plate. After 24 h of incubation, the cells were treated with compounds for 14 days. Then, the cells were fixed with 4% paraformaldehyde and stained with 0.5% crystal violet. The number of colonies was calculated. Data represent the mean ± SD from three independent experiments performed in triplicate wells.

### Flow cytometry assay

According to the manufacturer’s instructions and reference methods, the cell cycle and apoptosis analysis kit (Beyotime Biotechnology, Jiangsu, China) and Annexin-V-FLUOS staining kit (Roche, Germany) were used to evaluate the cell cycleprocess and the apoptotic ratio after compound **SKLB-D18**, BVD-523 and XMD8-92 treatment, respectively. Then, cell cycle distribution and cell apoptosis were measured on a flow cytometer (BD FACS Calibur). Finally, statistical analysis was quantified using FlowJo 10.0 software. PDGFRβ were stained using PDGFRβ antibody (Abclonal, A19531) and Alexa Fluor 488 antibody and surface expression were analyzed by flow cytometry.

### Transwell experiment

The Transwell chamber was cleaned with sterile PBS and irradiated with UV in a biosafety cabinet for at least 12 h. MDA-MB-231 and MDA-MB-468 cells of logarithmic growth phase were uniformly inoculated into the upper chamber of Transwell placed on a 24-well plate, with a volume of about 200 μL (about 3 × 10^4^ cells) and a volume of 500 μL in the lower chamber. After 4 to 6 h of cell adhesion, the upper chamber cell medium was changed to serum-free medium containing a series of specified concentrations of compounds (**SKLB-D18** (0.5, 1, 2 μM), BVD-523 (2 μM) and XMD8-92 (2 μM)), and the culture was continued for 16 h. Then the chamber was removed, cleaned with PBS, and gently wiped the cells on the surface of the upper chamber with a cotton swab, taking care not to touch the cells that migrated to the surface of the lower chamber. After the cells were fixed with 4% paraformaldehyde at room temperature, the cells were stained with 0.1% crystal violet solution at room temperature for 30 min. After cleaning with PBS, the cells on the surface of the upper chamber were gently wiped with a cotton swab to remove specific binding. After drying, an inverted microscope was used to take pictures, and statistical maps were drawn using GraphPad Prism 9.5 software.

### Immunofluorescence assay

MDA-MB-231 and MDA-MB-468 cells were seeded on glass coverslips placed in a 24-well plate. After treatment with 25c followed by MPP+, cells were fixed with 4% paraformaldehyde (Biosharp Life Science, AnHui, China), permeabilized with 0.2% Triton X-100 (Sigma-Aldrich, 9002-93-1), and blocked with immunol staining blocking buffer (Beyotime Biotechnology, Jiangsu, China). Fixed cells were incubated with primary antibody overnight at 4 °C and then washed with cold PBST and incubated with a secondary antibody at indoor temperature for 1 h. Subsequently, the cell nucleus was stained with 4′,6-diamidino-2-phenylindole (DAPI) in the dark for 15 min. Finally, cells were observed with a fluorescence microscope (Ni-E, Nikon, Japan).

### Liver microsomal metabolism

An appropriate amount of SD rat and human liver microsomal solution was incubated at 37 °C with a specified concentration of candidate compounds for 5 min, and then NADPH reaction system was added to initiate metabolic reaction. The reaction was terminated at a series of time points by adding acetonitrile solution containing an internal standard compound. Centrifuge at 12,000 rpm for 20 min, take the supernatant solution, and place it in a vacuum centrifuge to dry. After acetonitrile was dissolved, the supernatant solution was centrifugated, and the remaining compounds were quantified by LC-MS to evaluate the metabolic stability of liver microsomes.

### Plasma protein binding rate

Appropriate amount of SD rat plasma was taken and incubated with the specified concentration of compound at 37 °C for a specified time, and unbound compound solution was obtained by ultrafiltration method, and acetonitrile-containing internal standard compound was added to terminate the reaction, and the remaining compound was quantized by LC-MS to calculate the plasma protein binding rate. Calculation formula: Plasma protein binding rate (%) = (Total blood−Ultrafiltrate)/Total blood * 100%.

### Caco-2 bidirectional transport detection

Caco-2 cells were inoculated in a Transwell chamber with a pore size of 0.4 μm, and cultured for 15–20 days, the formation of the monolayer cell model was verified by the permeability determination of fluorescein sodium. Then a certain concentration of culture-medium containing candidate compounds was added to the upper and lower sides of different groups of cells, and the culture-medium at the corresponding position was absorbed after the specified time. After the internal standard compound was added, the content of candidate compounds was determined by LC-MS/MS to investigate the apparent permeability and effection rate, and to analyze the bidirectional transport characteristics of candidate compounds in the Caco-2 cell monolayer model.

### Pharmacokinetic studies

**SKLB-D18** was formulated using a composition of 10% DMSO, 10% Solutol, and 80% (20% HP-β-CD). **SKLB-D18** was administered via intravenous injection (1 mg/kg) and intragastric administration (10 mg/kg) on SD rats. At various time points post-administration (0.25 h, 0.5 h, 1 h, 2 h, 4 h, 6 h, 8 h, 24 h), blood samples (0.2 mL) were collected and centrifuged (6800 × *g*, 6 min, 2–8 °C) to separate plasma. Plasma samples were precipitated with methanol, vortexed, and centrifuged (18,000 × *g*, 4 °C, 7 min); the supernatant was analyzed using LC-MS/MS. Parameters such as AUC_0-t_, AUC_0-∞_, C_max_, T_max_, and T_1/2_ were calculated using Phoenix WinNonlin 7.0.

### In vivo xenograft studies

All procedures were performed in accordance with the Laboratory Animals Welfare Act, the Guide for the Care and Use of Laboratory Animals and the Guidelines and Policies. The rodent experiment was approved by IACUC (Institutional Animal Care and Use Committee) in the Experimental Animal Ethics Committee of West China Hospital (Approval No. 2021672A). 48 female nude mice (BALB/c-nu, 5 weeks, 15–18 g, purchased from Beijing HFK Bio-Technology Co., LTD) were acclimatized for one week in an SPF-grade animal facility (ventilated, 20–25 °C, 50–60% humidity). Each mouse (6 weeks, 17–19 g) was subcutaneously injected with 100 μL of MDA-MB-231 cell suspension (7.5 × 10^6^ cells/mouse) into the left anterior axilla. When the tumor volume reached approximately 100 mm^3^ (V = L × W^2^/2), the mice were randomly divided into six groups (*n* = 8 per group): (1) Control group (normal saline); (2) 25 mg/kg **SKLB-D18**-treated group; (3) 50 mg/kg **SKLB-D18**-treated group; (4) 50 mg/kg BVD-523-treated group; (5) 50 mg/kg XMD8-92-treated group; (6) BVD-523 (50 mg/kg) and XMD8-92 (50 mg/kg) combined treatment group. Drugs were freshly prepared with 5% DMSO, 10% Solutol HS-15, and 85% normal saline on the day of administration. Intragastric administration occurred once daily at the same time for 16 consecutive days. And the body weight and tumor volume of mice were measured every three days. After the administration period, all nude mice were euthanized by cervical dislocation the next day. Tumors and vital organs (heart, liver, spleen, lung, kidney) were dissected, and tumors were weighed, then immediately fixed in 4% paraformaldehyde or stored in −80 °C. Tumor growth inhibition rate (TGI) was calculated as TGI = (1 − tumor weight of the treatment group/tumor weight of the control group) * 100%.

### H&E staining

First, the fixed major organs of nude mice were dehydrated, embedded, and sectioned. The sections were stained with hematoxylin for 10–20 min, rinsed briefly, and differentiated with hydrochloric acid alcohol. After bluing in warm or weak alkaline water and a final rinse, the sections were immersed in 85% alcohol for 3–5 min. Eosin staining was then performed for 3–5 min, followed by dehydration in graded alcohol, clearing with xylene, and mounting with neutral resin. Staining results and tissue morphology were analyzed under a microscope.

### IHC analysis

Briefly, tumor tissues were dehydrated, embedded, and sectioned. The paraffin sections were deparaffinized and rehydrated. Sections for p-ERK1/2, p-ERK5, and Ki-67 staining underwent antigen retrieval using EDTA or citrate buffer. Endogenous peroxidase was blocked with hydrogen peroxide, and sections were blocked with bovine serum albumin at room temperature. Primary antibodies were incubated overnight, followed by HRP polymer-conjugated secondary antibody treatment. DAB substrate was used for staining, with Meyer’s hematoxylin counterstaining. Microscopic analysis and imaging were performed, analyzed with ImageJ, and statistical graphs were created using GraphPad Prism 9.5. All specimens followed standard SOPs for pathological examination.

### mRFP-GFP-LC3 adenovirus transfection

The cells were uniformly inoculated into 24-well plates and cultured for 24 h, 0.1 μL mRFP-GFP-LC3 adenovirus was added and cultured for 24 h. Subsequently, a series of compounds with a specified concentration were added and treated for 24 h. After washing with PBS for 3 times, the normal medium was replaced and the intracellular fluorescence was observed by fluorescence microscope to evaluate the autophagy flow.

### Lysosomal fluorescent label staining

The cells were inoculated into a 24-well plate with sterile slides, cultured for 24 h, and treated with the specified concentration of compound for the corresponding time. Configure 50 nM Lyso-Tracker Red working fluid. After preincubation at 37 °C, the cells were incubated for 60 min, and nuclear staining was performed using DAPI. Fluorescence intensity was observed and quantitatively analyzed by standing fluorescence microscope.

### Hoechst 33258

The cells were inoculated into a 24-well plate with sterile slides, cultured for 24 h, and treated with the specified concentration of compound for the corresponding time. The cell medium was discarded and washed 3 times with PBS. After the cells were fixed with 4% paraformaldehyde at room temperature, 500 μL Hoechst 33258 staining solution was added to each well and incubated for 10 min at room temperature and away from light. After incubation, the cells were cleaned with PBS to remove specific binding. The anti-fluorescence quencher was added to the slide and analyzed with a positive fluorescence microscope after sealing.

### ROS detection

The cells in the logarithmic growth phase were inoculated in a small dish (about 4 × 10^5^ cells per well). Add the specified concentration of compound and treat for 24 h. DCFH-DA solution was prepared with serum-free medium at the ratio of 1:1000 for each well, and the probe was loaded in situ and incubated for 20 min. Intact cells were collected with pancreatic enzymes, and the cells were suspended with PBS. The fluorescence intensity of the cells was detected by flow cytometry.

### MDA detection

The cell samples with the specified concentration of compound were collected and cleaved, and the supernatant of 100 μL was extracted by centrifugation at 12,000 × *g* for 10 min. According to the instructions, each sample was mixed with TBA dilution, TBA storage solution and antioxidant as the working solution for MDA detection. After adding the sample, it was heated at 100 °C for 15 min, and centrifugated at 1000 × *g* room temperature for 10 min. Then the absorbance of 532 nm was detected by enzyme label. The content of MDA in samples was calculated by standard curve.

### FerroOrange probe staining

The cells were inoculated in a 6-well plate and treated with a series of compounds at a specified concentration for 24 h. After the cells were washed with PBS, FerroOrange solution was added to HBSS solution at the volume ratio of 1:1000 for each sample, and 1 μM working solution was mixed, and 1 mL working solution was added to each well and incubated for 30 min. The fluorescence intensity was observed and quantitatively analyzed by inverted fluorescence microscope.

### Statistical analysis

All data were represented as means and standard deviations and analyzed with GraphPad Prism 9.5 software. Statistical comparison between different groups was performed by one-way ANOVA or two-way ANOVA. Differences were considered statistically significant when *p* < 0.05 (*p < 0.05; **p < 0.01; ***p < 0.001; ^#^*p* < 0.05; ^##^*p* < 0.01; ^###^*p* < 0.001).

## Supplementary information

Supplementary material

## Data Availability

Cocrystal structures that support the findings of this study were deposited to the PDB under accession numbers 9LNR and 9LTA and are listed in the pertinent figure legends and Supplementary Table S5. Data supporting the findings of this study are available from the corresponding author upon reasonable request.

## References

[1] Wahida, A. et al. The coming decade in precision oncology: six riddles. *Nat. Rev. Cancer* **23**, 43–54 (2022). 36434139 10.1038/s41568-022-00529-3

[2] Bedard, P. L., Hyman, D. M., Davids, M. S. & Siu, L. L. Small molecules, big impact: 20 years of targeted therapy in oncology. *Lancet* **395**, 1078–1088 (2020). 32222192 10.1016/S0140-6736(20)30164-1

[3] Kabir, A. & Muth, A. Polypharmacology: the science of multi-targeting molecules. *Pharmacol. Res.* **176**, 106055 (2022). 34990865 10.1016/j.phrs.2021.106055

[4] Tomaselli, D., Lucidi, A., Rotili, D. & Mai, A. Epigenetic polypharmacology: a new frontier for epi‐drug discovery. *Med. Res. Rev.* **40**, 190–244 (2019). 31218726 10.1002/med.21600 PMC6917854

[5] Chen, Z. et al. Flexible scaffold-based cheminformatics approach for polypharmacological drug design. *Cell* **187**, 2194–2208.e2122 (2024). 38552625 10.1016/j.cell.2024.02.034

[6] Pan, X. et al. Development of small molecule extracellular signal-regulated kinases (ERKs) inhibitors for cancer therapy. *Acta Pharm. Sin. B.* **12**, 2171–2192 (2022). 35646548 10.1016/j.apsb.2021.12.022 PMC9136582

[7] Degirmenci, U., Wang, M. & Hu, J. Targeting aberrant RAS/RAF/MEK/ERK signaling for cancer therapy. *Cells* **9**, 198 (2020). 31941155 10.3390/cells9010198 PMC7017232

[8] Lavoie, H., Gagnon, J. & Therrien, M. ERK signalling: a master regulator of cell behaviour, life and fate. *Nat. Rev. Mol. Cell Biol.* **21**, 607–632 (2020). 32576977 10.1038/s41580-020-0255-7

[9] Moschos, S. J. et al. Development of MK-8353, an orally administered ERK1/2 inhibitor, in patients with advanced solid tumors. *JCI Insight* **3**, e92352 (2018). 29467321 10.1172/jci.insight.92352 PMC5916243

[10] Aronchik, I. et al. Efficacy of a covalent ERK1/2 inhibitor, CC-90003, in KRAS-mutant cancer models reveals novel mechanisms of response and resistance. *Mol. Cancer Res.* **17**, 642–654 (2019). 30275173 10.1158/1541-7786.MCR-17-0554

[11] Ward, R. A. et al. Discovery of a potent and selective oral inhibitor of ERK1/2 (AZD0364) that is efficacious in both monotherapy and combination therapy in models of nonsmall cell lung cancer (NSCLC). *J. Med. Chem.* **62**, 11004–11018 (2019). 31710489 10.1021/acs.jmedchem.9b01295

[12] Weekes, C. et al. A phase Ib study to evaluate the MEK inhibitor cobimetinib in combination with the ERK1/2 inhibitor GDC-0994 in patients with advanced solid tumors. *Oncologist* **25**, 833–e1438 (2020). 32311798 10.1634/theoncologist.2020-0292 PMC7543243

[13] Belair, D. G. et al. Investigation into the role of ERK in tyrosine kinase inhibitor-induced neuropathy. *Toxicol. Sci.* **181**, 160–174 (2021). 33749749 10.1093/toxsci/kfab033

[14] Wu, J. et al. Characterization and management of ERK inhibitor associated dermatologic adverse events: analysis from a nonrandomized trial of ulixertinib for advanced cancers. *Invest. N. Drugs* **39**, 785–795 (2021). 10.1007/s10637-020-01035-9 PMC9282166 33389388

[15] Shuai, W. et al. Structure-guided discovery and preclinical assessment of novel (thiophen-3-yl)aminopyrimidine derivatives as potent ERK1/2 inhibitors. *J. Med. Chem.* **67**, 6425–6455 (2024). 38613499 10.1021/acs.jmedchem.3c02392

[16] Jaiswal, B. S. et al. ERK mutations and amplification confer resistance to ERK-inhibitor therapy. *Clin. Cancer Res.* **24**, 4044–4055 (2018). 29760222 10.1158/1078-0432.CCR-17-3674

[17] Adam, C. et al. Efficient suppression of NRAS-driven melanoma by co-inhibition of ERK1/2 and ERK5 MAPK pathways. *J. Invest. Dermatol.* **140**, 2455–2465.e2410 (2020). 32376279 10.1016/j.jid.2020.03.972

[18] de Jong, P. R. et al. ERK5 signalling rescues intestinal epithelial turnover and tumour cell proliferation upon ERK1/2 abrogation. *Nat. Commun.* **7**, 11551–11565 (2016). 27187615 10.1038/ncomms11551 PMC4873670

[19] Vaseva, A. V. et al. KRAS suppression-induced degradation of MYC is antagonized by a MEK5-ERK5 compensatory mechanism. *Cancer Cell* **34**, 807–822.e807 (2018). 30423298 10.1016/j.ccell.2018.10.001 PMC6321749

[20] Zhang, W. et al. Aurora-A/ERK1/2/mTOR axis promotes tumor progression in triple-negative breast cancer and dual-targeting Aurora-A/mTOR shows synthetic lethality. *Cell Death Dis.* **10**, 606 (2019). 31406104 10.1038/s41419-019-1855-z PMC6690898

[21] Xu, Q. et al. The extracellular-regulated protein kinase 5 (ERK5) enhances metastatic burden in triple-negative breast cancer through focal adhesion protein kinase (FAK)-mediated regulation of cell adhesion. *Oncogene* **40**, 3929–3941 (2021). 33981002 10.1038/s41388-021-01798-2 PMC8195737

[22] Bhatt, A. B. et al. Diverse and converging roles of ERK1/2 and ERK5 pathways on mesenchymal to epithelial transition in breast cancer. *Transl. Oncol.* **14**, 101046 (2021). 33761370 10.1016/j.tranon.2021.101046 PMC8020482

[23] Wang, X. et al. The emerging roles of MAPK-AMPK in ferroptosis regulatory network. *Cell Commun. Signal.* **21**, 200–216 (2023). 37580745 10.1186/s12964-023-01170-9 PMC10424420

[24] Zhang, C. et al. Ferroptosis in cancer therapy: a novel approach to reversing drug resistance. *Mol. Cancer* **21**, 47 (2022). 35151318 10.1186/s12943-022-01530-y PMC8840702

[25] Chen, F. et al. A fluorescent prodrug to fight drug-resistant lung cancer cells via autophagy-driven ferroptosis. *Sens. Actuat B-Chem.* **400**, 134871 (2024).

[26] Liu, K. et al. Induction of autophagy-dependent ferroptosis to eliminate drug-tolerant human retinoblastoma cells. *Cell Death Dis.* **13**, 521 (2022). 35654783 10.1038/s41419-022-04974-8 PMC9163041

[27] Miller, D. C. et al. Modulation of ERK5 activity as a therapeutic anti-cancer strategy. *J. Med. Chem.* **66**, 4491–4502 (2023). 37002872 10.1021/acs.jmedchem.3c00072 PMC10108346

[28] Wang, G. et al. Discovery of a novel dual-target inhibitor of ERK1 and ERK5 that induces regulated cell death to overcome compensatory mechanism in specific tumor types. *J. Med. Chem.* **63**, 3976–3995 (2020). 32078308 10.1021/acs.jmedchem.9b01896

[29] Shu, L. et al. NRG1 regulates Fra-1 transcription and metastasis of triple-negative breast cancer cells via the c-Myc ubiquitination as manipulated by ERK1/2-mediated Fbxw7 phosphorylation. *Oncogene* **41**, 907–919 (2022). 34992218 10.1038/s41388-021-02142-4

[30] Frank, K. J. et al. Extensive preclinical validation of combined RMC-4550 and LY3214996 supports clinical investigation for KRAS mutant pancreatic cancer. *Cell Rep. Med.* **3**, 100815–100828 (2022). 36384095 10.1016/j.xcrm.2022.100815 PMC9729824

[31] Ye, J., Wu, J. & Liu, B. Therapeutic strategies of dual-target small molecules to overcome drug resistance in cancer therapy. *Biochim. Biophys. Acta Rev. Cancer* **1878**, 188866 (2023). 36842765 10.1016/j.bbcan.2023.188866

[32] Whitehead, C. E. et al. A first-in-class selective inhibitor of EGFR and PI3K offers a single-molecule approach to targeting adaptive resistance. *Nat. Cancer* **5**, 1250–1266 (2024). 38992135 10.1038/s43018-024-00781-6 PMC11357990

[33] Blake, J. F. et al. Discovery of (S)-1-(1-(4-chloro-3-fluorophenyl)-2-hydroxyethyl)-4-(2-((1-methyl-1H-pyrazol-5-yl)amino)pyrimidin-4-yl)pyridin-2(1H)-one (GDC-0994), an extracellular signal-regulated kinase 1/2 (ERK1/2) inhibitor in early clinical development. *J. Med. Chem.* **59**, 5650–5660 (2016). 27227380 10.1021/acs.jmedchem.6b00389

[34] Elkins, J. M. et al. X-ray crystal structure of ERK5 (MAPK7) in complex with a specific inhibitor. *J. Med. Chem.* **56**, 4413–4421 (2013). 23656407 10.1021/jm4000837 PMC3683888

[35] Liu, F., Yang, X., Geng, M. & Huang, M. Targeting ERK, an Achilles’ Heel of the MAPK pathway, in cancer therapy. *Acta Pharm. Sin. B* **8**, 552–562 (2018). 30109180 10.1016/j.apsb.2018.01.008 PMC6089851

[36] Pegram, L. M. et al. Activation loop dynamics are controlled by conformation-selective inhibitors of ERK2. *Proc. Natl Acad. Sci. USA* **116**, 15463–15468 (2019). 31311868 10.1073/pnas.1906824116 PMC6681744

[37] Chaikuad, A. et al. A unique inhibitor binding site in ERK1/2 is associated with slow binding kinetics. *Nat. Chem. Biol.* **10**, 853–860 (2014). 25195011 10.1038/nchembio.1629 PMC4687050

[38] Kidger, A. M. et al. Dual-mechanism ERK1/2 inhibitors exploit a distinct binding mode to block phosphorylation and nuclear accumulation of ERK1/2. *Mol. Cancer Ther.* **19**, 525–539 (2020). 31748345 10.1158/1535-7163.MCT-19-0505

[39] Fang, C. Y. et al. Antimetastatic potentials of salvianolic acid A on oral squamous cell carcinoma by targeting MMP-2 and the c-Raf/MEK/ERK pathway. *Environ. Toxicol.* **33**, 545–554 (2018). 29385302 10.1002/tox.22542

[40] Pramanik, K. K. & Mishra, R. ERK-mediated upregulation of matrix metalloproteinase-2 promotes the invasiveness in human oral squamous cell carcinoma (OSCC). *Exp. Cell Res.* **411**, 112984 (2022). 34951997 10.1016/j.yexcr.2021.112984

[41] Liao, M. R. et al. Targeting regulated cell death (RCD) with small-molecule compounds in triple-negative breast cancer: a revisited perspective from molecular mechanisms to targeted therapies. *J. Hematol. Oncol.* **15**, 44 (2022). 35414025 10.1186/s13045-022-01260-0 PMC9006445

[42] Gámez-García, A. et al. ERK5 inhibition induces autophagy-mediated cancer cell death by activating ER stress. *Front. Cell Dev. Biol.* **9**, 742049–742054 (2021). 34805151 10.3389/fcell.2021.742049 PMC8600073

[43] Nikooie, R., Moflehi, D. & Zand, S. Lactate regulates autophagy through ROS-mediated activation of ERK1/2/m-TOR/p-70S6K pathway in skeletal muscle. *J. Cell Commun. Signal* **15**, 107–123 (2021). 33398722 10.1007/s12079-020-00599-8 PMC7905011

[44] Xu, X. et al. ERK1/2/mTOR/Stat3 pathway-mediated autophagy alleviates traumatic brain injury-induced acute lung injury. *Biochim. Biophys. Acta Mol. Basis Dis.* **1864**, 1663–1674 (2018). 29466698 10.1016/j.bbadis.2018.02.011

[45] Tusa, I. et al. ERK5 is activated by oncogenic BRAF and promotes melanoma growth. *Oncogene* **37**, 2601–2614 (2018). 29483645 10.1038/s41388-018-0164-9 PMC5945581

[46] Benito-Jardón, L. et al. Resistance to MAPK Inhibitors in Melanoma Involves Activation of the IGF1R-MEK5-Erk5 Pathway. *Cancer Res.* **79**, 2244–2256 (2019). 30833419 10.1158/0008-5472.CAN-18-2762

