# Supplementary material for: A first-in-class selective inhibitor of ERK1/2 and ERK5 overcomes drug resistance with a single-molecule strategy
Source: Signal Transduct Target Ther. 2025 Feb 20;10:70. doi: 10.1038/s41392-025-02169-z (PMC11842588; doi:10.1038/s41392-025-02169-z)
Supplement: Supplementary file 1 — Supplementary material [file 41392_2025_2169_MOESM1_ESM.docx]

Supplementary Materials for

**A first-in-class selective inhibitor of ERK1/2 and ERK5 overcomes drug resistance with a single-molecule strategy**

Huan Xiao^#1^, Aoxue Wang^#1^, Wen Shuai^#1^, Yuping Qian^2^, Chengyong Wu^1^, Xin Wang^1^, Panpan Yang^1^, Qian Sun^1^, Guan Wang*^1^, Liang Ouyang*^1^, Qiu Sun*^1^

Corresponding to: Guan Wang (guan8079@163.com), Liang Ouyang (ouyangliang@scu.edu.cn), Qiu Sun (sunqiu@scu.edu.cn)

**This PDF file includes:**

Supplementary Fig. S1 to 10

Supplementary Table S1 to 8

Chemistry

Kinase Selectivity Analysis

Original films of immunoblots


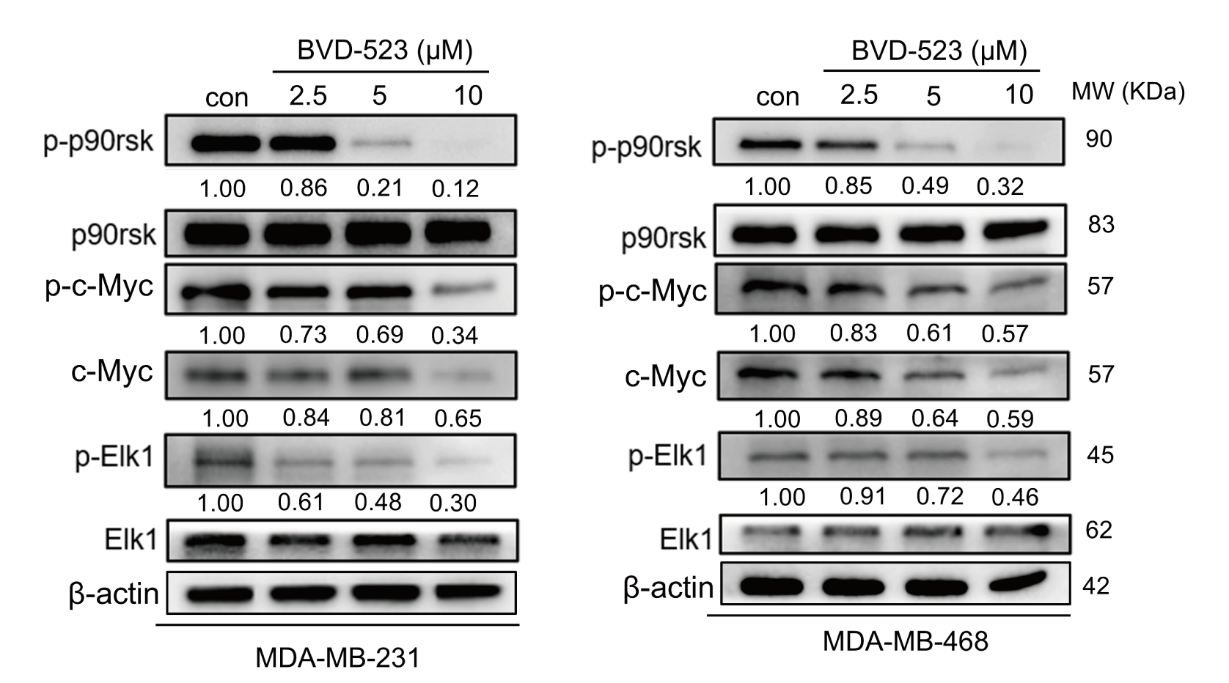


**Fig.S1** Immunoblotting analysis of protein levels of p-RSKp90, RSKp90, p-c-Myc, c-Myc, p-ELK1 and ELK1 in MDA-MB-231 and MDA-MB-468 cells following 24 h incubation with BVD-523 (2.5, 5, 10 μM).

**
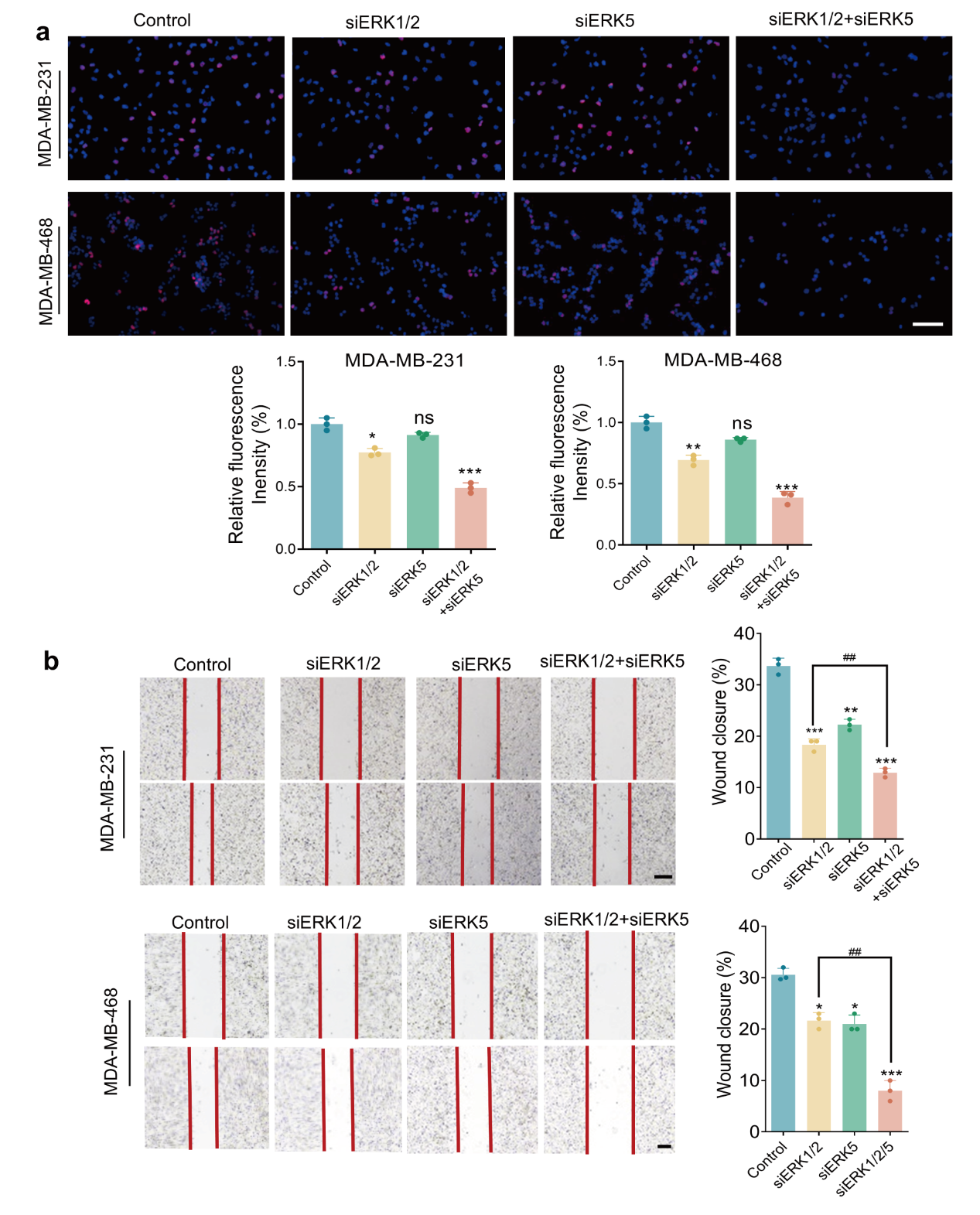
**

**Fig.S2** Biological functions of ERK1/2 and ERK5 in the proliferation and metastasis of TNBC cells. **a.** Cell viability was determined by EDU assay after transfecting with ERK1/2, ERK5 and ERK1/2/5 siRNA respectively. Scale bar, 100 μm. **b.** Wound healing assay was performed after transfecting with ERK1/2, ERK5 and ERK1/2/5 siRNA respectively. Scale bar, 50 μm. Data are presented as Mean ± SD. Compared to the Control group: ns, not significant; **p*<0.05, ***p*<0.01, ****p*<0.001. Compared to the siERK1/2 group: ##*p*<0.01.


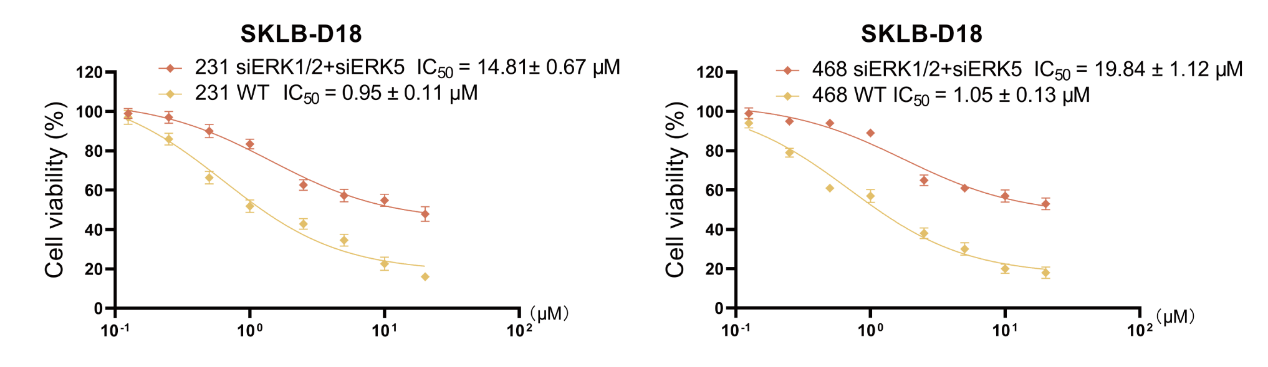


**Fig.S3** The antiproliferative activity of **SKLB-D18** against TNBC cell lines (MDA-MB-231, MDA-MB-468 and corresponding knockdown cell lines) was assessed using the MTT assay.


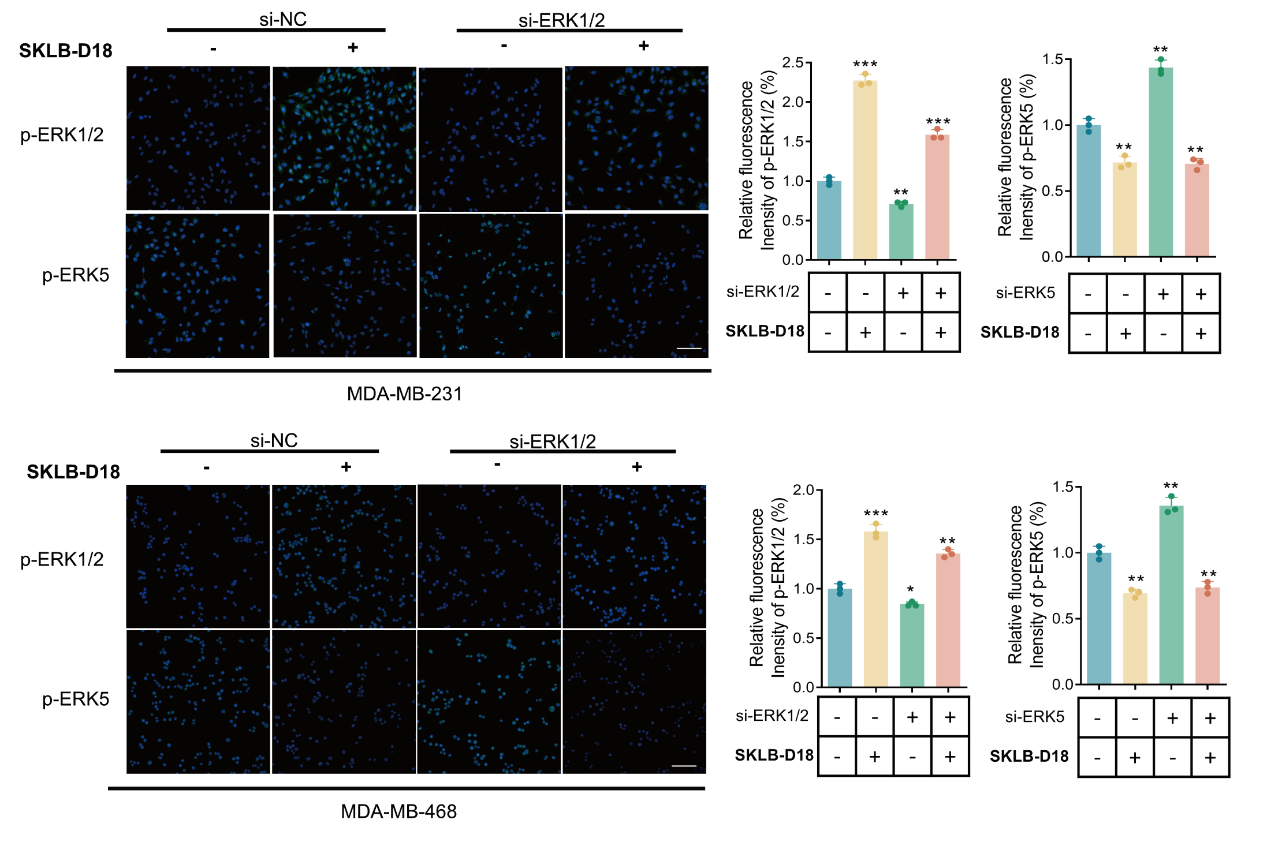


**Fig. S4** Immunofluorescence analysis of p-ERK1/2 and p-ERK5 expression levels in MDA-MB-231 and MDA-MB-468 cells following 24 h incubation with **SKLB-D18** (5 μM) and transfection with siRNA-ERK1/2 and siRNA-NC. Data are presented as Mean ± SD. Compared to the Control group: *p<0.05, **p<0.01, ***p<0.001.


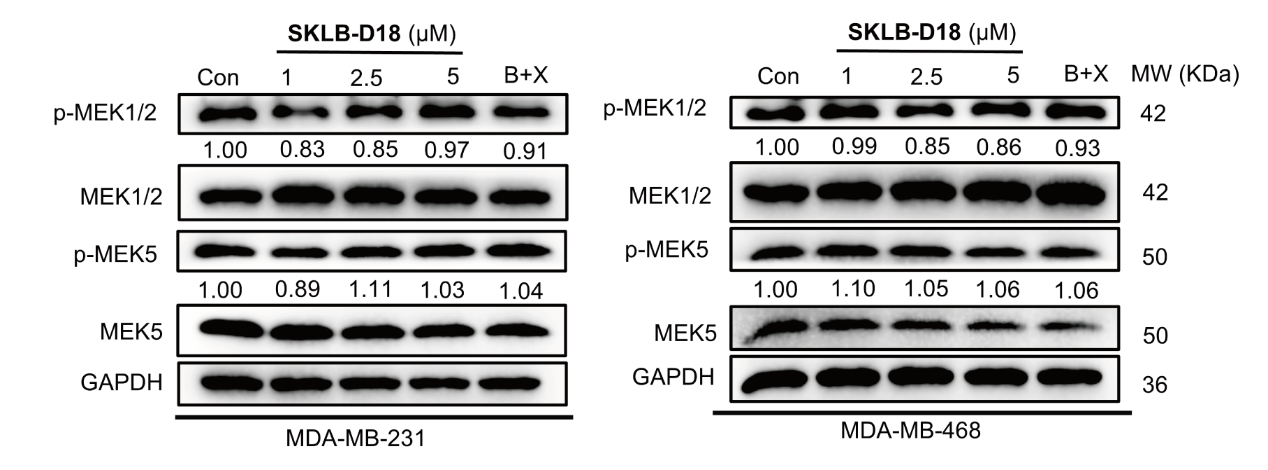


**Fig. S5** Immunoblotting analysis of p-MEK5 and p-MEK1/2 levels in cells following 24 h incubation with **SKLB-D18** (1, 2.5, 5 μM), and combination of BVD-523 (5 μM) and XMD8-92 (5 μM).


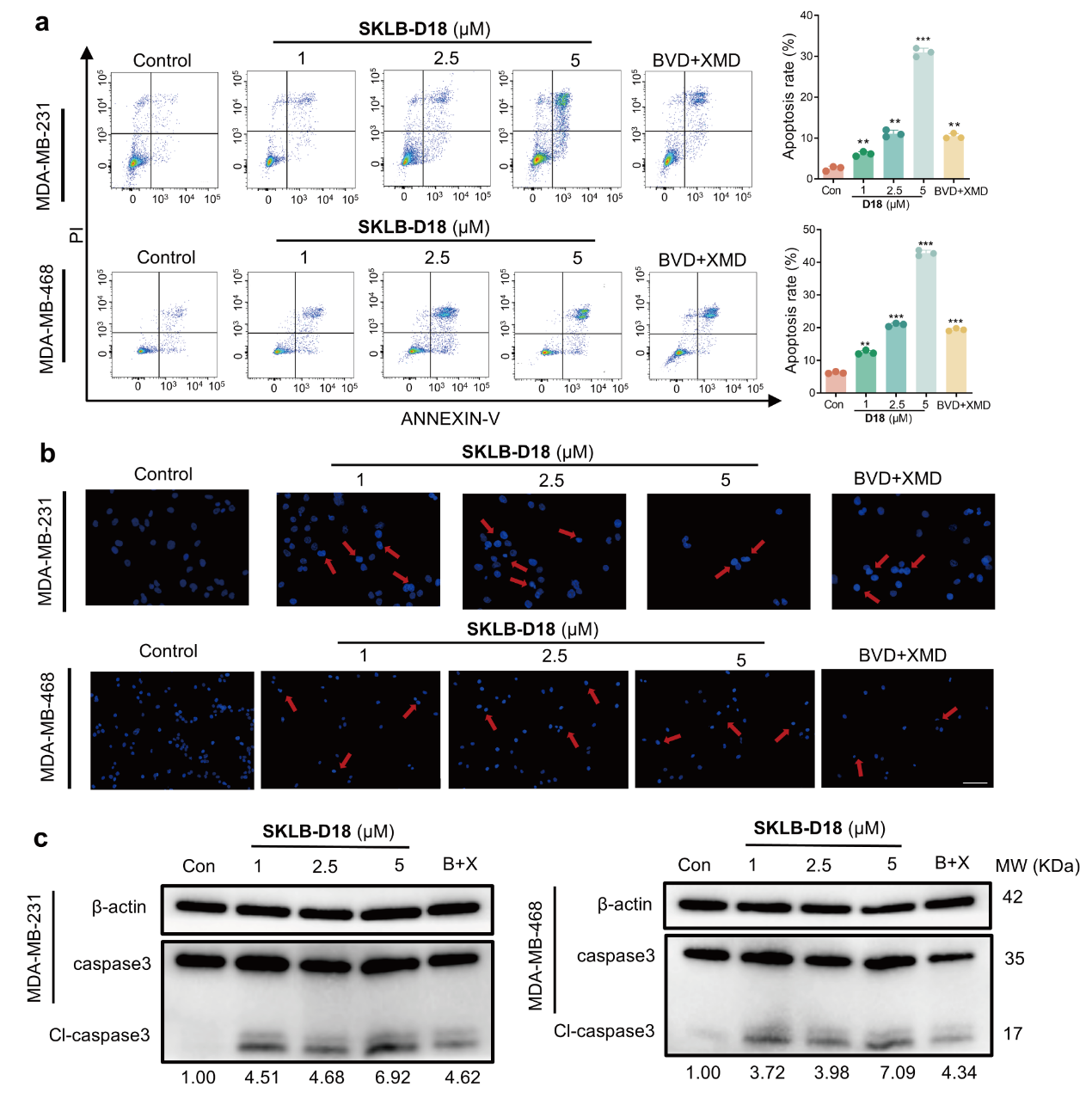


**Fig. S6** **SKLB-D18** induced apoptosis in dose-dependent way. **a.** Flow cytometry analysis of cell apoptosis following 24 h incubation with **SKLB-D18** (1, 2.5, 5 μM), and combination of BVD-523 (5 μM) and XMD8-92 (5 μM). **b.** Hoechst stainning after treatment of **SKLB-D18** (1, 2.5, 5 μM), and combination of BVD-523 (5 μM) and XMD8-92 (5 μM). **c.** Immunobloting analysis of caspase 3 following 24 h incubation with **SKLB-D18** (1, 2.5, 5 μM), and combination of BVD-523 (5 μM) and XMD8-92 (5 μM). Data are presented as Mean ± SD. Compared to the Control group: ***p*<0.01, ****p*<0.001.


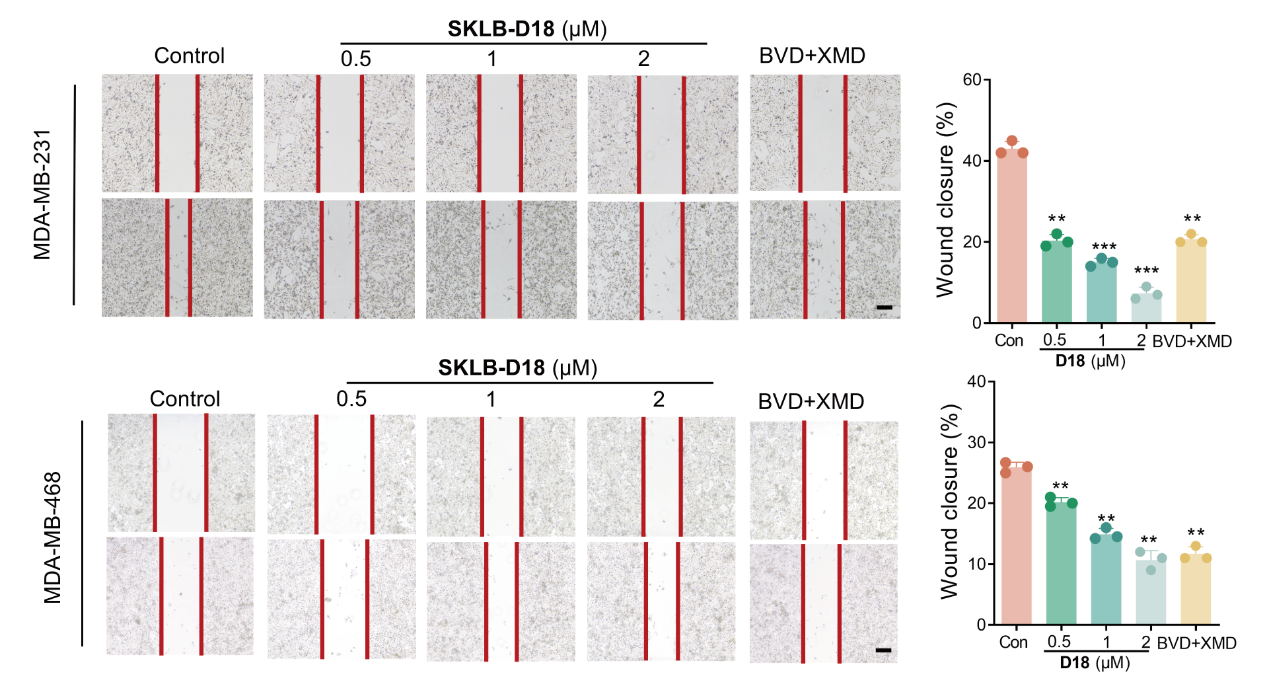


**Fig. S7** Wound healing assay to evaluate wound closure and corresponding migration rate of cells following 48 h incubation with **SKLB-D18** (0.5, 1, 2 μM), and combination of BVD-523 (2 μM) and XMD8-92 (2 μM). Scale bar, 40 μm. Data are presented as Mean ± SD. Compared to the Control group: ***p*<0.01, ****p*<0.001.


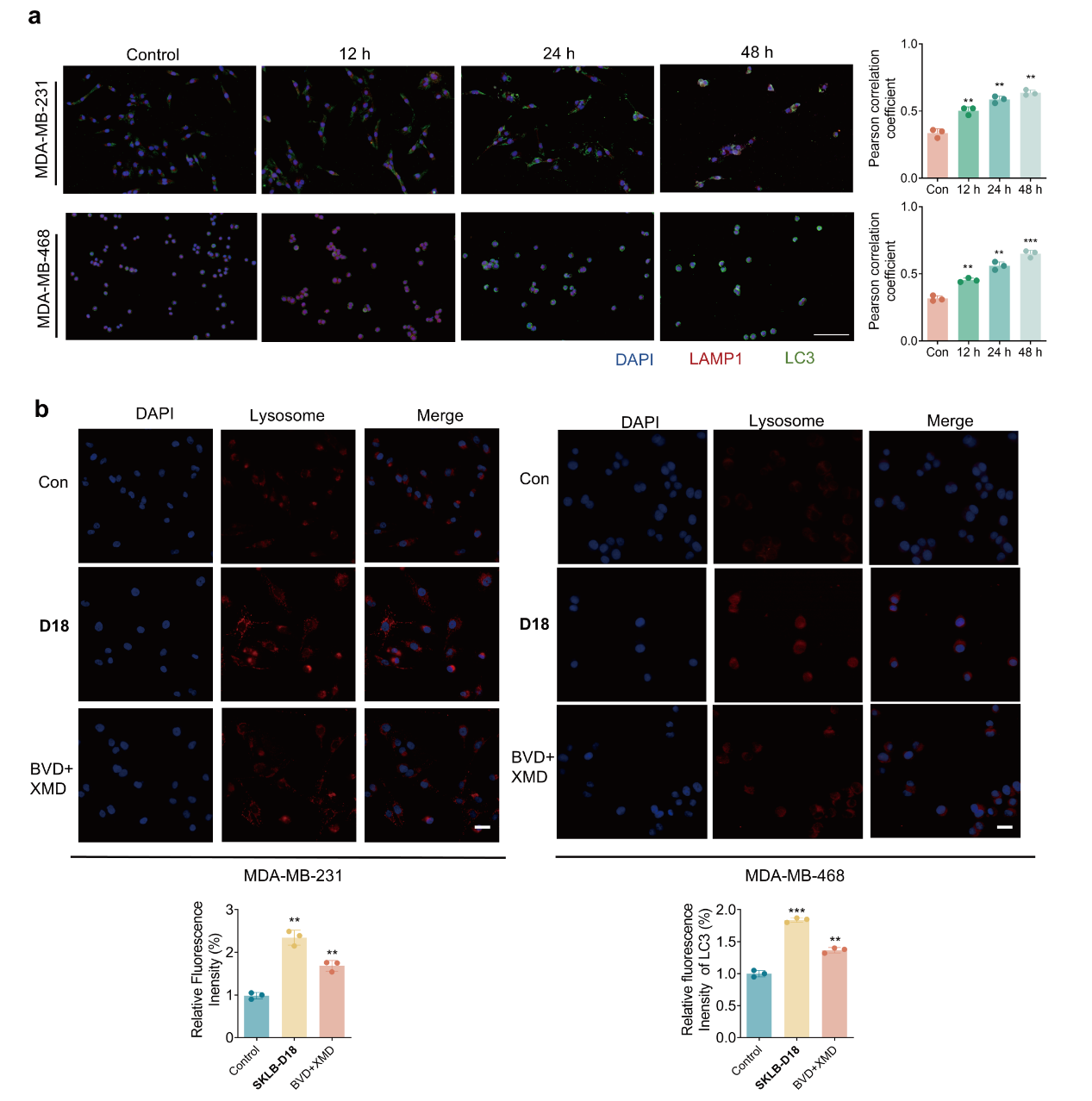


**Fig. S8 SKLB-D18** induced complete autophagy in MDA-MB-231 and MDA-MB-468 cells. **a**. Immunofluorescence analysis of co-localization of LC3 and LAMP1 in cells following treatment with **SKLB-D18** (12 h, 24 h, 48 h). Scale bar, 100 μm. **b**. Immunofluorescence analysis of lysosome activity in cells following treatment with **SKLB-D18** (5 μM), and combination of BVD-523 (5 μM) and XMD8-92 (5 μM). Scale bar, 100 μm. Data are presented as Mean ± SD. Compared to the Control group: ns, not significant; **p*<0.05, ***p*<0.01, ****p*<0.001.


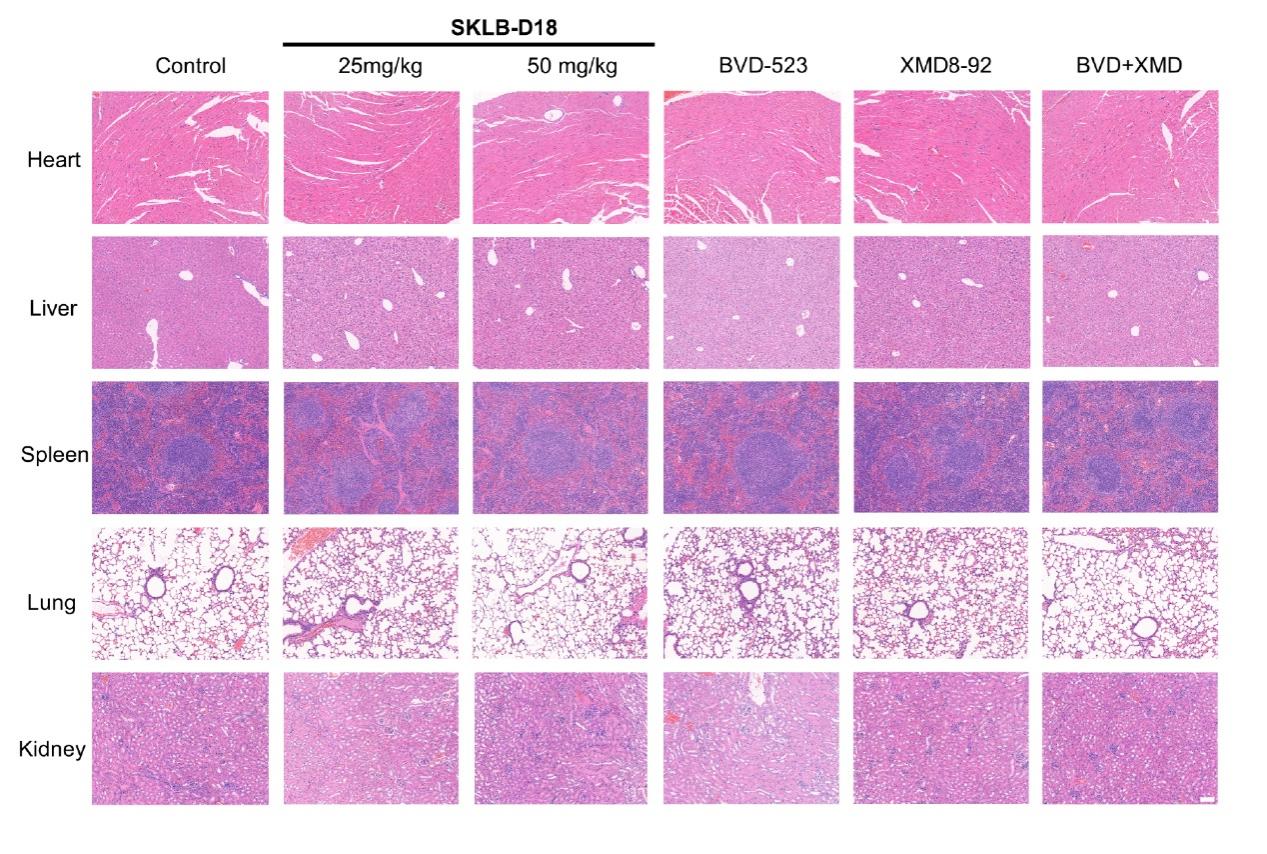


**Fig. S9** H&E staining analysis of major organ tissues (heart, liver, spleen, lung, kidney) from MDA-MB-231 xenograft tumor tissues in each group, scale bar 100 μm.


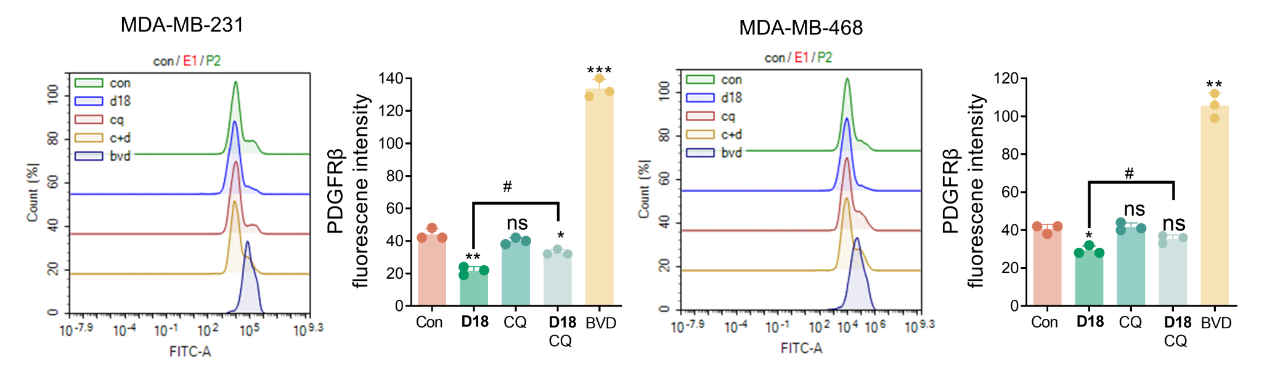


**Fig. S10** Flow Cytometry to evaluate PDGFRβ expression levels of TNBC cells flowing 24 h incubation with **SKLB-D18** (5 μM), BVD-523 (5 μM) and combination of **SKLB-D18** (5 μM) and CQ (1 μM). Data are presented as Mean ± SD. Compared to the Control group: ns, not significant; **p*<0.05, ***p*<0.01. Compared to the **SKLB-D18** treatment group: #*p*<0.01.

**Structural optimization and structure-activity relationship study of ERK1/2/5 inhibitors**

**Table S1** The *in vitro* ERK1/2/5 inhibitory activity and antiproliferative activity of compounds **6a-ag**.

^^

| **No.** | **R_1_** | **Enzyme inhibitory activity**  **(500 nM, %) ^[a]^** | | | **Anti-proliferative activity**  **(10 μM, %) ^[b]^** | | |
| --- | --- | --- | --- | --- | --- | --- | --- |
|  |  | **ERK1** | **ERK2** | **ERK5** | **MDA-MB-231** | **MDA-MB-468** | |
| **6a** |  | 95.72 ± 0.65 | 97.44 ± 3.45 | 63.80 ± 0.32 | 15.06 ± 0.35 | 10.78 ± 1.32 |  |
| **6b** |  | 40.45 ± 1.23 | 39.03 ± 2.13 | 28.02 ± 1.34 | 3.15 ± 1.22 | 25.81 ± 2.43 |  |
| **6c** |  | 21.57 ± 2.34 | 14.38 ± 0.24 | 19.12 ± 2.15 | 5.71 ± 1.21 | 3.51 ± 0.77 |  |
| **6d** |  | 93.12 ± 1.89 | 91.52 ± 0.31 | 34.57 ± 1.42 | 37.21 ± 0.72 | 23.35 ± 2.34 |  |
| **6e** |  | 97.82 ± 0.43 | 99.00 ± 0.56 | 70.61 ± 0.98 | 11.49 ± 1.48 | 0.70 ± 0.45 |  |
| **6f** |  | 89.31 ± 0.87 | 89.54 ± 2.31 | 35.94 ± 1.21 | 11.85 ± 2.31 | 57.68 ± 1.09 |  |
| **6g** |  | 70.32 ± 0.91 | 72.75 ± 2.14 | 73.61 ± 3.12 | 6.82 ± 2.13 | 20.23 ± 0.32 |  |
| **6h** |  | 35,78 ± 1.24 | 34.56 ± 0.98 | 17.98 ± 0.89 | 23.65 ± 1.27 | 7.43 ± 3.45 |  |
| **6i** |  | 62.90 ± 1.09 | 65.23 ± 1.21 | 73.52 ± 1.34 | 5.65 ± 0.22 | -12.07 ± 2.31 |  |
| **6j** |  | 58.08 ± 2.54 | 60.98 ±1.23 | 22.5 ± 2.31 | 25.73 ± 0.57 | 10.11 ± 0.76 |  |
| **6k** |  | 59.39 ± 3.21 | 57.85 ± 0.88 | 36.99 ± 0.32 | 39.12 ± 1.23 | 23.41 ± 0.48 |  |
| **6l** |  | 47.12 ± 9.23 | 46.65 ± 0.32 | 32.34 ± 0.89 | 33.90 ± 1.22 | 23.35 ± 2.37 |  |
| **6m** |  | 50.98 ± 3.23 | 53.43 ± 0.43 | 35.45 ± 1.32 | 34.62 ± 1.24 | 30.41 ± 0.68 |  |
| **6n** |  | 67.08 ± 0.98 | 66.54 ± 2.11 | 18.64 ±2.32 | 43.93 ± 0.21 | 33.21 ± 0.54 |  |
| **6o** |  | 25,67 ± 1.52 | 23.49 ± 0.99 | 44.36 ± 0.89 | 7.20 ± 1.34 | 2.11 ± 1.21 |  |
| **6p** |  | 86.34 ± 0.79 | 84.67 ± 0.65 | 77.49 ± 0.89 | 33.51 ± 0.32 | 28.42 ± 1.21 |  |
| **6q** |  | 80.23 ± 0.68 | 82.11 ±0.61 | 65.90 ± 3.12 | 29.43 ± 3.25 | 33.20± 0.44 |  |
| **6r** |  | 88.34 ± 1.78 | 89.45 ± 1.55 | 67.34 ± 3.20 | 15.53 ± 0.90 | 30.21 ± 1.11 |  |
| **6s** |  | 90.32 ± 4.32 | 91.32 ± 0.06 | 63.41 ± 3.21 | 21.34 ± 2.12 | 27.45 ± 0.88 |  |
| **6t** |  | 93.24 ± 2.45 | 92.10 ± 0.66 | 67.45 ± 0.78 | 26.82 ± 1.34 | 22.09 ± 2.33 |  |
| **6u** |  | 93.78 ± 3.41 | 94.56 ± 0.98 | 71.36 ± 2.48 | 15.92 ± 1.23 | 4.61 ± 0.98 |  |
| **6v** |  | 95.09 ± 1.45 | 96.11 ± 2.31 | 75.19 ± 1.21 | 25.42 ± 0.66 | 40.59 ± 2.31 |  |
| **6w** |  | 77,46 ± 1.57 | 75.32 ± 1.22 | 33.12 ± 0.77 | 19.86 ± 0.94 | 22.85 ± 1.32 |  |
| **6x** |  | 67.23 ± 0.50 | 66.33 ± 2.31 | 35.78 ± 0.82 | 5.52 ± 1.25 | 3.46 ± 0.74 |  |
| **6y** |  | 50.78 ± 0.89 | 51.08 ±2.31 | 23.21 ± 3.45 | 8.87 ± 1.11 | 9.83 ± 0.93 |  |
| **6z** |  | 55.23 ± 2,41 | 53.45 ± 1.22 | 31.23 ± 0.78 | 4.52 ± 2.87 | 13.92 ± 1.32 |  |
| **6aa** |  | 81.34 ± 0.59 | 83.31 ± 2.33 | 54.09 ± 0.90 | 9.74 ± 1.32 | 10.42 ± 2.12 |  |
| **6ab** |  | 98.88 ± 0.80 | 99.30 ± 0.56 | 73.12 ± 0.06 | 0.53 ± 1.23 | 22.51 ± 1.51 |  |
| **6ac** |  | 95.98 ± 1.02 | 97.67 ± 0.90 | 69.90 ± 2.56 | 22.57 ± 1.11 | 18.49 ± 0.23 |  |
| **6ad** |  | 95.32 ± 2.10 | 94.17 ± 2.31 | 63.32 ± 0.23 | 21.31 ± 0.88 | 16.31 ± 0.03 |  |
| **6ae** |  | 93.45 ± 1.80 | 95.32 ± 0.08 | 61.02 ± 1.22 | 15.96 ± 0.09 | 4.62 ± 0.89 |  |
| **6af** |  | 95.24 ± 2.01 | 93.56 ± 2.31 | 76.63 ± 0.78 | 13.54 ± 2.12 | 9.46 ± 0.82 |  |
| **6ag** |  | 92.13 ± 1.04 | 94.53 ± 1.21 | 65.13 ± 0.90 | 33.67 ± 0.08 | 25.61 ± 0.45 |  |

^a/b^ Reported compounds were tested in triplicate. Data are presented as mean ± SD.

**Table S2** The *in vitro* ERK1/2/5 inhibitory activity and antiproliferative activity of compounds **9a-i**.

| **No.** | **R_2_** | **Enzyme inhibitory activity**  **(500 nM, %) ^[a]^** | | | **Anti-proliferative activity**  **(10 μM, %) ^[b]^** | |
| --- | --- | --- | --- | --- | --- | --- |
|  |  | **ERK1** | **ERK2** | **ERK5** | **MDA-MB-231** | **MDA-MB-468** |
| **9a** |  | 41.09 ± 1.05 | 45.57 ± 0.34 | 21.12 ± 2.34 | 1.23 ± 1.20 | 8.70 ± 3.21 |
| **9b** |  | 62.45 ± 0.91 | 65.43 ± 0.04 | 52.16 ± 3.21 | 3.91 ± 0.76 | 16.90 ± 0.33 |
| **9c** |  | 80.98 ± 0.96 | 83.12 ± 2.31 | 54.34 ± 0.89 | 14.32 ± 3.42 | 10.92 ± 1.45 |
| **9d** |  | 45.29 ± 1.30 | 43.22 ± 0.23 | 23.12 ± 3.21 | 7.54 ± 1.23 | 24.33 ± 0.84 |
| **9e** |  | 77.32 ± 2.34 | 76.45 ± 0.01 | 45.32 ± 0.09 | 23.33 ± 0.56 | 25.10 ± 1.53 |
| **9f** |  | 82.54 ± 2.10 | 85.34 ± 0.78 | 75.42 ± 1.03 | 43.32 ± 0.32 | 59.24 ± 2.10 |
| **9g** |  | 95.67 ± 0.78 | 94.74 ± 1.23 | 80.17 ± 0.46 | 75.23 ± 0.47 | 60.52 ± 0.12 |
| **9h** |  | 77.02 ± 3.21 | 78.90 ± 2.31 | 65.54 ± 0.88 | 11.21 ± 0.47 | 20.15 ± 0.77 |
| **9i** |  | 72.10 ± 1.54 | 69.65 ± 2.11 | 65.51 ± 1.47 | 1.12 ± 0.88 | -2.92 ± 0.75 |

^a/b^ Reported compounds were tested in triplicate. Data are presented as mean ± SD.

**Table S3** The *in vitro* ERK1/2/5 inhibitory activity and antiproliferative activity of compounds **15a-e**.

| **No.** | **R_3_** | **X** | **Enzyme inhibitory activity**  **(500 nM, %) ^[a]^** | | | **Anti-proliferative activity**  **(10 μM, %) ^[b]^** | |
| --- | --- | --- | --- | --- | --- | --- | --- |
|  |  |  | **ERK1** | **ERK2** | **ERK5** | **MDA-MB-231** | **MDA-MB-468** |
| **15a** | H | S | 63.23 ± 2.45 | 65.76 ± 3.22 | 43.12 ± 1.21 | 33.12 ± 1.35 | 21.22 ± 0.78 |
| **15b** | CH_3_ | S | 42.31 ± 1.43 | 44.37 ± 1.09 | 47.79 ± 0.06 | 21.1 ± 0.38 | 9.02 ± 3.21 |
| **15c** | F | S | 80.45 ± 0.79 | 78.90 ± 0.09 | 86.11 ± 0.77 | 55.61 ± 0.67 | 43.4 ± 1.25 |
| **15d** | Cl | O | 82.32 ± 3.65 | 84.45 ± 2.11 | 66.78 ± 1.22 | 55.21 ± 0.83 | 52.1 ± 2.01 |
| **15e** | Cl | NH | 89.01 ± 1.42 | 87.34 ± 0.84 | 53.22 ± 4.10 | 63.2 ± 0.04 | 52.8 ± 0.92 |

^a/b^ Reported compounds were tested in triplicate. Data are presented as mean ± SD.

**Table S4** The *in vitro* ERK1/2/5 inhibitory activity and antiproliferative activity of compounds **18a-p**.

| **No.** | **R_4_** | **Enzyme inhibitory activity**  **(500 nM, %) ^[a]^** | | | **Anti-proliferative activity**  **(10 μM, %) ^[b]^** | |
| --- | --- | --- | --- | --- | --- | --- |
|  |  | **ERK1** | **ERK2** | **ERK5** | **MDA-MB-231** | **MDA-MB-468** |
| **18a** |  | 25.34 ± 4.21 | 22.81 ± 2.13 | 78.27 ± 3.21 | 85.82 ± 0.83 | 60.42 ± 1.02 |
| **18b** |  | 30.45 ± 1.23 | 29.44 ± 0.89 | 69.91 ± 3.42 | 78.21 ± 2.41 | 60.13 ± 0.09 |
| **18c** |  | 10.20 ± 2.31 | 7.82 ± 0.46 | 73.68 ± 4.51 | 61.12 ± 1.03 | 40.41 ± 2.91 |
| **18d** |  | 25,14 ± 0.92 | 23.97 ± 0.90 | 78.85 ± 2.67 | 85.64 ± 1.02 | 50.45 ± 2.12 |
| **18e** |  | 95.21 ± 0.80 | 92.34 ± 2.31 | 35.99 ± 1.09 | 32.29 ± 0.23 | -4.82 ± 1.09 |
| **18f** |  | 96.49 ± 1.71 | 98.66 ± 0.77 | 52.50 ± 0.43 | 86.27 ± 0.92 | 39.21 ± 1.42 |
| **18g** |  | 45.02 ± 0.91 | 43.70 ± 1.24 | 53.11 ± 2.41 | 46.16 ± 0.90 | 33.82 ± 1.02 |
| **18h** |  | 98.06 ± 0.79 | 99.76 ± 0.90 | 89.83 ± 0.21 | 69.8 ± 2.10 | 30.63 ± 0.03 |
| **18i** |  | 2.13 ± 1.56 | -1.29 ± 0.02 | 71.14 ± 0.43 | 41.35 ± 1.20 | 30.29 ± 0.83 |
| **18j** |  | 7.98 ± 0.60 | 5.97 ± 1.02 | 81.84 ± 2.10 | 39.25 ± 0.21 | 59.17 ± 2.12 |
| **18k** |  | 45.23 ± 2.12 | 43.61 ± 1.45 | 86.64 ± 0.89 | 39.77 ± 0.31 | -3.93 ± 1.21 |
| **18l** |  | -3.90 ± 2.11 | -7.5 ± 1.32 | 90.65 ± 0.32 | 28.64 ± 0.78 | -5.74 ± 1.35 |
| **18m** |  | 92.34 ± 0.89 | 90.78 ± 1.34 | 63.38 ± 0.23 | 62.91 ± 0.21 | 80.55 ± 0.90 |
| **18n** |  | 90.27 ± 1.78 | 91.03 ± 1.23 | 79.74 ± 0.07 | 54.82 ± 1.21 | 86.37 ± 0.43 |
| **18o**  **(D18)** |  | 95.62 ± 0.84 | 93.19 ± 1.45 | 93.14 ± 1.03 | 93.44 ± 1.21 | 92.18 ± 0.93 |
| **18p** |  | 90.32 ± 1.37 | 92.23 ± 0.92 | 33.60 ± 0.89 | 33.4 ± 2.31 | 28.24 ± 0.42 |

^a/b^ Reported compounds were tested in triplicate. Data are presented as mean ± SD.

**Table S5.** X-Ray diffraction data and refinement statistics for the crystal structures of **SKLB-D18** in complex with ERK2/5.

| **Protein** | | **ERK2** | | **ERK5** |
| --- | --- | --- | --- | --- |
| **PDB ID** | | **9LNR** | | **9LTA** |
| **Data collection** |  | |  | |
| Space Group | | P212121 | | P1211 |
| Unit Cell (Å) | | 44.01 70.48 120.07 | | 46.64 156.92 49.73 |
| Unit Cell (°) | | 90.00 90.00 90.00 | | 90.00 90.49 90.00 |
| Wavelength (Å) | | 0.97918 | | 0.97861 |
| Resolution (Å) | | 60.78-2.10 | | 49.73-2.33 |
| R_merge_ | | 0.237 (3.102) | | 0.142 (0.929) |
| CC_1/2_ | | 99.7 (46.5) | | 99.6 (82.5) |
| I/sigma | | 8.3 (1.1) | | 10.7 (2.7) |
| Completeness (%) | | 100.0 (99.9) | | 97.1 (99.2) |
| Redundancy | | 13.1 (13.4) | | 6.6 (6.5) |
| **Structure refinement** | | | |  |
| Number of measured reflections | | 297094 | | 194849 |
| Number of unique reflections | | 22718 | | 29547 |
| R_work_ / R_free_ (%) | | 21.02/27.70 | | 24.93/29.16 |
| No. atoms | | 2905 | | 5482 |
| Protein | | 2724 | | 5369 |
| Other | | 181 | | 113 |
| Average B value (Å^2^) | | 42.13 | | 35.40 |
| Protein | | 41.92 | | 35.32 |
| Other | | 45.19 | | 39.38 |
| **R.m.s. deviations** | | | |  |
| Bonds (Å) | | 0.008 | | 0.004 |
| Angle (°) | | 1.033 | | 0.786 |
| **Ramachandran plot statistics (%)** | | | |  |
| Most favorable | | 96.64 | | 94.07 |
| allowed | | 3.06 | | 5.62 |
| Disallowed | | 0.31 | | 0.30 |

**Table S6.** Liver microsome metabolic stability of **SKLB-D18** in different species.

| Species | Residual rate (%) | T_1/2_ (min) | CL_int_（ml/min/mg） |
| --- | --- | --- | --- |
| SD rat | 25.8 | 32.51 | 0.0426 |
| Human | 69.42 | 91.61 | 0.0151 |

**Table S7.** Bidirectional transport characteristics of **SKLB-D18** in the Caco-2 cell monolayer model.

| Time (min) | AP→BL Papp  (× 10^-6^ cm/s) | BL→AP Papp  (× 10^-6^ cm/s) | Efflux rate (%) |
| --- | --- | --- | --- |
| 30 | 0.106 ± 0.01 | 0.011 ± 0.01 | 10.02 ± 1.1  1 |
| 60 | 0.318 ± 0.06 | 0.117 ± 0.02 | 13.7 ± 0.4 |
| 120 | 2.232 ± 0.17 | 0.305 ± 0.04  7 | 13.7 ± 0.5 |

**Table S8.** Pharmacokinetic parameters of **SKLB-D18** in SD rats.

| Parameter | SD rats^a^ | |
| --- | --- | --- |
|  | i.v. (1 mg/kg) | p.o. (10 mg/kg) |
| T_1/2_ (h) | 2.01 ± 0.16 | 3.71 ± 0.40 |
| C_max_ (ng/mL) | 58.72 ± 7.22 | 53.71 ± 10.70 |
| AUC_0-t_ (ng·h/mL) | 135.56 ± 5.73 | 303.93 ± 39.11 |
| AUC_0-∞_ (ng·h/mL) | 143.25 ± 6.60 | 456.92 ± 53.14 |
| V_ss_(mL/kg) | 16990.99 ± 567.62 | - |
| V_z_ (mL/kg) | 20240.48 ± 1384.08 | - |
| CL (mL/(min·kg)) | 116.52 ± 5.45 | - |
| *F* (%) | - | 22.42 ± 2.89 |

**Chemistry**

**General Information for Compounds Synthesis and Analysis**

All reagents and solvents were obtained from Bidepharm Ltd. (Shanghai, China) and used without further purification. Anhydrous solvents were purchased from Aladdin Ltd. (Shanghai, China). Column chromatography was carried out on silica gel (200−300 and 100−200 meshes). The reaction progress was monitored by thin-layer chromatography (TLC), and silica gel plates with fluorescence F-254 were utilized and investigated with UV light. Nuclear magnetic resonance (NMR) spectral data were determined using a Bruker AV II-400 spectrometer and Bruker AV II-600. The following abbreviations were used to describe the spectral data. Coupling constants (*J*) were expressed in hertz (Hz). Proton coupling patterns were described as singlet (s), doublet (d), triplet (t), doublet of doublet (dd), quartet (q), and multiplet (m). Chemical shifts were reported in regions per million (ppm, δ) relative to tetramethylsilane (TMS) as an internal standard. All compounds are over 95% pure, which was determined by a model LC-2030 high-performance liquid chromatography (HPLC) instrument (Shimadzu Vietnam, Co., Ltd., Kyoto, Japan) with a GL-C18 reverse phase column (4.6 mm × 150 mm, 5 μm) with MeCN and water as the mobile phase. Melting points were acquired on an electrothermal Micromelting point apparatus (INESA Physico-Optical Instrument Co.,Ltd., Shanghai). High-resolution mass spectra were recorded on Quadrupole-electrostatic field orbital trap high resolution mass spectrometry system (Q Exactive plus, Thermo Fisher Scientific, Waltham, MA, USA).

**Scheme 1. Synthesis route of compounds 6a−ag.**

Reagents and conditions: (i) B_2_Pin_2_, Pd(dppf)Cl_2_, KOAc, 1,4-dioxane, N_2_, 100 ℃, 84%; (ii) 2,4,5-trichloropyrimidine, Pd(PPh_3_)_4_, Cs_2_CO_3_,1,4-dioxane/H_2_O, N_2_, 85 ℃, 2 h, 68%; (iii) 1-methyl-5-aminopyrazole, Pd(OAc)_2_, BINAP, Cs_2_CO_3_, 1,4-dioxane, N_2_, 100 ℃, 64%; (iv) LiOH·H_2_O, THF, 80 ℃, 95%; (v) HATU, DIPEA, DMF, r.t. 40-70%.

**Scheme 2. Synthesis route of compounds 9a−i.**

Reagents and conditions: (i) Pd(OAc)_2_, BINAP, Cs_2_CO_3_, 1,4-dioxane, N_2_, 100 ℃, 45-56%; (ii) LiOH·H_2_O, THF, 80 ℃, 88-92%; (iii) 4-methoxyaniline, HATU, DIPEA, DMF, r.t. 44-68%.

**Scheme 3. Synthesis route of compounds 15a−e.**

Reagents and conditions: (i) B_2_Pin_2_, Pd(dppf)Cl_2_, KOAc, 1,4-dioxane, N_2_, 100 ℃, 66-72%; (ii) Pd(PPh_3_)_4_, Cs_2_CO_3_,1,4-dioxane/H_2_O, N_2_, 85℃, 2 h, 58-63%; (iii) 3-((dimethylamino)methyl)-aniline, Pd(OAc)_2_, BINAP, Cs_2_CO_3_, 1,4-dioxane, N_2_, 100℃, 57-60%; (iv) LiOH·H2O, THF, 80 ℃, 85-88%; (v) HATU, DIPEA, DMF, r.t. 43-66%.

**Scheme 4. Synthesis route of compounds 18a−p.**

Reagents and conditions: (i) 3-((dimethylamino)methyl)- aniline, Pd(OAc)_2_, BINAP, Cs_2_CO_3_, 1,4-dioxane, N_2_, 100 ℃, 55%, ; (ii) LiOH·H_2_O, THF, 80 ℃, 89%; (iii) HATU, DIPEA, DMF, r.t. 43-72%.

**General Procedures for Compounds Synthesis**

**Synthesis of *methyl 4-(4,4,5,5-tetramethyl-1,3,2-dioxaborolan-2-yl)thiophene-2-carboxylate* (2):**

4-Bromothiophene-2-carboxylate (compound **1**, 5 g, 22.60 mmol, 1 equiv) was dissolved in anhydrous 1,4-dioxane (80 mL). Bis(pinacolato)diboron (B_2_Pin_2_, 6.9 g, 27.15 mmol, 1.2 equiv) and Pd(dppf)Cl_2_ (1.64 mg, 2.25 mmol, 0.1 equiv) were added, followed by KOAc (6.65 g, 67.85 mmol, 3 equiv) as a base. The reaction mixture was purged with N_2_ and stirred at 100°C for 4 h. After monitoring the reaction completion by TLC, the mixture was cooled to room temperature and filtered to remove solid impurities. The solvent (1,4-dioxane) was evaporated under reduced pressure distillation to yield the crude product. Then it was purified using column chromatography on silica gel (200-300 mesh), eluting with a gradient of petroleum ether (PE) and ethyl acetate (EA) from 40:1 to 20:1, yielding white solid compound 4-(4,4,5,5-tetramethyl-1,3,2-dioxaborolan-2-yl)thiophene-2-carboxylate (**2**) with a yield of 84%.

^1^H NMR (400 MHz, DMSO-*d*_6_), *δ* (ppm): 8.32, 8.32, 7.86, 7.86, 3.83, 1.29.

**Synthesis of *methyl 4-(2,5-dichloropyrimidin-4-yl)thiophene-2-carboxylate* (3):**

Compound **2** (4.5 g, 16.80 mmol, 1 equiv) was dissolved in a mixture of 1,4-dioxane and water (6:1, 60 mL). Then, 2,4,5-trichloropyrimidine (3.69 g, 20.15 mmol, 1.2 equiv), Pd(PPh_3_)_4_ (1.94 g, 1.7 mmol, 0.1 equiv), and Cs_2_CO_3_ (16.4 g, 50.35 mmol, 3 equiv) were added. The reaction mixture was purged with N_2_ and stirred at 85°C for 4 h. After completion (monitored by TLC), the reaction was quenched with water and extracted with EA (500 mL). The organic layer was washed with water and saturated NaCl solution, dried over anhydrous Na_2_SO_4_, and concentrated under reduced pressure distillation to yield the crude product. The crude product was purified using column chromatography on silica gel (200-300 mesh), eluting with a gradient of PE and EA from 20:1 to 10:1, yielding intermediate **3** with a yield of 68%.

^1^H NMR (400 MHz, DMSO-*d*_6_), *δ* (ppm): 8.98 (d, *J* = 1.4 Hz, 1H), 8.88 (t, *J* = 1.4 Hz, 1H), 8.33 (d, *J* = 1.7 Hz, 1H), 3.88 (s, 3H).

**Synthesis of methyl *4-(5-chloro-2-((1-methyl-1H-pyrazol-5-yl)amino)pyrimidin-4-yl)thiophene-2-carboxylate* (4):**

Compound **3** (3 g, 10.4 mmol, 1 equiv) and 1-methyl-1H-pyrazol-5-ylamine (1.21 g, 12.45 mmol, 1.2 equiv) were dissolved in anhydrous 1,4-dioxane (50 mL). Pd(OAc)2 (232.95 mg, 1.04 mmol, 0.1 equiv), BINAP (646.10 mg, 1.04 mmol, 0.1 equiv), and Cs_2_CO_3_ (10.15 g, 31.15 mmol, 3 equiv) were added. The mixture was purged with N_2_ and stirred at 100°C for 4 h. After the reaction is completed, the mixture was filtered and the solvent evaporated under reduced pressure distillation. The crude product was purified using column chromatography, eluting with DCM and MeOH from 40:1 to 20:1, yielding intermediate **4** with a yield of 64%.

**Synthesis of *4-(5-chloro-2-((1-methyl-1H-pyrazol-5-yl)amino)pyrimidin-4-yl)thiophene-2-carboxylic acid* (5):**

Compound **4** (2.4 g, 6.85 mmol, 1 equiv) was dissolved in a mixture of THF and water (THF: H_2_O = 2: 1). LiOH·H_2_O (1.44 g, 34.3 mmol, 5 equiv) was added, and the mixture was stirred at 80°C for 1 h. After completion (monitored by TLC), the organic phase was evaporated under reduced pressure distillation. The aqueous phase was adjusted to pH 4-5 with dilute HCl, precipitating a yellow solid. The precipitate was filtered, washed with water, and dried to yield intermediate acid **5** with a yield of 95%.

^1^H NMR (400 MHz, DMSO-*d_6_*), *δ* (ppm): 9.72 (s, 1H), 8.72 – 8.61 (m, 1H), 8.60 (s, 1H), 8.24 (s, 1H), 7.37 (s, 1H), 6.26 (s, 1H), 3.69 (s, 3H).

**Synthesis of compounds 6a-ag:**

Compound **5** (80 mg, 0.24 mmol, 1 equiv) was dissolved in DMF (10 mL). HATU (99.66 mg, 0.26 mmol, 1.1 equiv) and DIPEA (124.50 µL, 0.71 mmol, 3 equiv) were added, followed by various amine derivatives (0.26 mmol, 1.1 equiv). The reaction was stirred at room temperature for 2 h. After completion (monitored by TLC), the mixture was extracted with EA (100 mL) and water (100 mL). The organic layer was washed with water and saturated NaCl solution, dried over anhydrous Na_2_SO_4_, and concentrated under reduced pressure distillation. The crude product was purified using Preparative Thin-Layer Chromatography (PTLC), eluting with DCM: MeOH = 10:1, yielding the target compound **6a-ag** with a yield of 40-70%.

**Synthesis of compounds 9a-i:**

In Scheme 2, compounds **9a-i** were synthesized through a reaction sequence similar to that in Scheme 1, except that different amine derivatives were coupled with intermediate **3** via Buchwald-Hartwig coupling to yield compounds **7a-i.** These were sequentially demethylated to obtain compounds **8a-i**, followed by condensation with 4-methoxyaniline to produce compounds **9a-i** with a yield of 44-68%.

**Synthesis of compounds 15a-e:**

Similarly, compounds **15a-e** were synthesized through a procedure in Scheme 3. Various five-membered ring compounds **10a-e** underwent Miyaura borylation with B_2_Pin_2_ to produce intermediates **11a-e**. These intermediates were then subjected to Suzuki coupling with 5-H/F/CH3-2,4-dichloropyrimidine to afford compounds **12a-e**. The subsequent steps involved Buchwald-Hartwig coupling, LiOH·H_2_O-mediated demethylation, and acid-amide condensation to yield the final products **15a-e** with a yield of 43-66%.

**Synthesis of compounds 18a-p:**

The synthetic route depicted in Scheme 4 is similar to that in Scheme 1, except that intermediate **3** underwent Buchwald-Hartwig coupling with 3-(dimethylamino)methyl)aniline to produce intermediate **16**. ^1^H NMR (400 MHz, DMSO-*d*_6_), *δ* (ppm): 9.91 (s, 1H), 8.82 (d, *J* = 1.5 Hz, 1H), 8.63 (s, 1H), 8.44 (d, *J* = 1.6 Hz, 1H), 7.85 (s, 1H), 7.54 (d, *J* = 8.1 Hz, 1H), 7.26 (t, *J* = 7.8 Hz, 1H), 6.93 (d, *J* = 7.5 Hz, 1H), 3.88 (s, 3H), 3.42 (s, 2H), 2.19 (s, 6H). Compound **16** was then demethylated to obtain intermediate **17**, which was subsequently condensed with various amine derivatives to generate the desired compounds **18a-p** with a yield of 43-72%.

**Synthesis of *4-(5-chloro-2-((3-((dimethylamino)methyl)phenyl) amino) pyrimidin-4-yl)-N-morpholinothiophene-2-carboxamide* (18o/SKLB-D18):**

4-(5-chloro-2-((3-((dimethylamino)methyl)phenyl)amino)pyrimidin-4-yl)thiophene-2-carboxylic acid (**17**, 80 mg, 0.21 mmol, 1 equiv) was dissolved in DMF(10 mL). HATU (87.4 mg, 0.23 mmol, 1.1 equiv) and DIPEA (109.73 µL, 0.63 mmol, 3 equiv) were added, followed by N-Aminomorpholine (23.46mg, 0.23 mmol, 1.1 equiv). The reaction was stirred at room temperature for 2 h. After completion (monitored by TLC), the mixture was extracted with EA (100 mL) and water (100 mL). The organic layer was washed with water and saturated NaCl solution, dried over anhydrous Na_2_SO_4_, and concentrated under reduced pressure distillation. The crude product was purified using Preparative Thin-Layer Chromatography (PTLC), eluting with DCM: MeOH = 10:1, yielding the target compound **SKLB-D18** with a yield of 55.78%.

**The Structural Characterization of Compound 6a-6ag:**

*****4-(5-chloro-2-((1-methyl-1H-pyrazol-5-yl)amino)pyrimidin-4-yl)-N-(4-methylpiperazin-1-yl)thiophene-2-carboxamide (6a).*** ^1^H NMR (400 MHz, DMSO-*d*_6_) *δ* (ppm): 9.70 (s, 1H), 9.17 (s, 1H), 8.60 – 8.56 (m, 2H), 8.50 (d, *J* = 6.9 Hz, 1H), 7.36 (d, *J* = 1.9 Hz, 1H), 6.26 (d, *J* = 2.0 Hz, 1H), 3.70 (s, 3H), 3.01-2.84 (m, 4H), 2.83-2.70 (m, 4H), 2.23 (s, 3H). ^13^C NMR (100 MHz, DMSO-*d*_6_) *δ* (ppm): 161.67, 159.63, 158.92, 156.59, 138.09, 137.80, 135.87, 135.11, 133.27, 128.88, 117.41, 99.42, 55.62, 54.86, 54.50, 54.20, 45.92, 35.96. HRMS (ESI)^+^ calculated for C_18_H_22_ClN_8_OS, [M + H] ^+^: *m/z* 433.1320, found 433.1316. HPLC purity 98.58%. White solid, m.p. 134.2-136.6℃, yield 50.04%.

** ***(4-(5-chloro-2-((1-methyl-1H-pyrazol-5-yl)amino)pyrimidin-4-yl)thiophen-2-yl)(4-methylpiperazin-1-yl)methanone* (6b).** ^1^H NMR (400 MHz, DMSO-*d*_6_) *δ* (ppm): 9.71 (s, 1H), 8.67 – 8.55 (m, 2H), 7.86 (s, 1H), 7.36 (d, *J* = 1.9 Hz, 1H), 6.26 (d, *J* = 1.9 Hz, 1H), 3.69 (s, 3H), 3.65 (t, *J* = 5.1 Hz, 4H), 2.37 (t, *J* = 5.0 Hz, 4H), 2.23 (s, 3H). ^13^C NMR (100 MHz, DMSO-*d*6) *δ* (ppm): 162.03, 159.76, 158.85, 156.12, 138.12, 137.79, 136.82, 133.63, 129.80, 117.43, 99.45, 55.00, 46.02, 35.95, 35.92. HRMS (ESI)^+^ calculated for C_18_H_20_ClN_7_OS, [M + H] ^+^: *m/z* 418.1211, found 418.1208. HPLC purity 98.22%. White solid, m.p. 201.3-203.9℃, yield 53.44%.

 ***(4-(5-chloro-2-((1-methyl-1H-pyrazol-5-yl)amino)pyrimidin-4-yl)thiophen-2-yl)(4-isopropylpiperazin-1-yl)methanone* (6c).** ^1^H NMR (400 MHz, DMSO-*d*_6_) *δ* (ppm): 9.71 (s, 1H), 8.59 (d, *J* = 6.2 Hz, 2H), 7.86 (s, 1H), 7.35 (s, 1H), 6.27 (s, 1H), 3.69 (m, 3H), 3.63 (m, 3H), 2.75-2.66 (m, 1H), 2.48 (d, *J* = 5.1 Hz, 4H), 0.99 (d, *J* = 6.6 Hz, 6H). ^13^C NMR (100 MHz, DMSO-*d*_6_) *δ* (ppm): 161.88, 159.76, 158.87, 156.12, 138.11, 137.88, 137.71, 136.81, 133.58, 129.76, 117.43, 99.45, 54.17, 48.66, 35.96, 18.54. HRMS (ESI)^+^ calculated for C_20_H_25_ClN_7_OS, [M + H] ^+^: *m/z* 446.1522, found 446.1522. HPLC purity 99.38%. White solid, m.p.160.3-163.2℃, yield 55.91%.

*****4-(5-chloro-2-((1-methyl-1H-pyrazol-5-yl)amino)pyrimidin-4-yl)-N-(tetrahydro-2H-thiopyran-4-yl)thiophene-2-carboxamide* (6d).** ^1^H NMR (400 MHz, DMSO-*d*_6_) *δ* (ppm): 9.75 (s, 1H), 8.83 – 8.57 (m, 2H), 8.49 (s, 1H), 8.46 – 8.32 (m, 1H), 7.38 (d, *J* = 1.9 Hz, 1H), 6.28 (d, *J* = 2.2 Hz, 1H), 3.93-3.74 (m, 1H), 3.70 (s, 3H), 3.01 – 2.60 (m, 4H), 2.27 – 2.04 (m, 2H), 1.82 – 1.62 (m, 2H). ^13^C NMR (100 MHz, DMSO-*d*_6_) *δ* (ppm): 160.17, 159.59, 158.88, 156.89, 140.85, 138.21, 137.75, 137.58, 134.64, 129.01, 117.50, 99.56, 48.19, 44.89, 35.95, 34.01, 27.74. HRMS (ESI)^+^ calculated for C_18_H_20_ClN_6_OS_2_, [M + H] ^+^: *m/z* 435.0823, found 435.0823. HPLC purity 99.04%. White solid, m.p. 137.2-141.3℃, yield 57.31%.

*****4-(5-chloro-2-((1-methyl-1H-pyrazol-5-yl)amino)pyrimidin-4-yl)-N-morpholino-thiophene-2-carboxamide* (6e).** ^1^H NMR (400 MHz, DMSO-*d*_6_) *δ* (ppm): 9.70 (d, *J* = 7.4 Hz, 1H), 9.26 (s, 1H), 8.59 – 8.57 (m, 1H), 8.50 (d, *J* = 9.8 Hz, 1H), 7.36 (d, *J* = 1.9 Hz, 1H), 6.26 (s, 1H), 3.85 (d, *J* = 11.4 Hz, 2H), 3.69 (s, 3H), 3.66 (d, *J* = 9.1 Hz, 2H), 2.96 - 2.88 (m, 2H), 2.89 - 2.65 (m, 2H). ^13^C NMR (100 MHz, DMSO-*d*_6_) *δ* (ppm): 161.67, 159.65, 158.93, 156.56, 138.01, 137.80, 135.97, 135.17, 133.33, 128.97, 117.42, 99.43, 66.49, 66.18, 56.22, 54.96, 35.96. HRMS (ESI)^+^ calculated for C_17_H_19_ClN_7_O_2_S, [M + H] ^+^: *m/z* 420.1004, found 420.1000. HPLC purity 98.12%. White solid, m.p. 206.7-209.1℃, yield 61.28%.

*****4-(5-chloro-2-((1-methyl-1H-pyrazol-5-yl)amino)pyrimidin-4-yl)-N-((1r,4r)-4-hydroxycy-clohexyl)thiophene-2-carboxamide* (6f).** ^1^H NMR (400 MHz, DMSO-*d*_6_) *δ* (ppm): 9.71 (s, 1H), 8.59 (s, 1H), 8.48 (d, *J* = 9.7 Hz, 2H), 8.36 (s, 1H), 7.36 (d, *J* = 1.9 Hz, 1H), 6.26 (d, *J* = 1.9 Hz, 1H), 4.71 – 4.48 (m, 1H), 3.69 (s, 3H), 3.41 (s, 2H), 1.84 (t, *J* = 14.8 Hz, 4H), 1.43 – 1.33 (m, 2H), 1.29 – 1.22 (m, 2H). ^13^C NMR (100 MHz, DMSO-*d*_6_) *δ* (ppm): 160.38, 159.56, 158.92, 156.89, 141.05, 138.11, 137.82, 137.56, 134.48, 128.81, 117.43, 99.54, 68.76, 48.51, 35.94, 35.91, 34.64, 30.74. HRMS (ESI)^+^ calculated for C_19_H_22_ClN_6_O_2_S, [M + H] ^+^: *m/z* 433.1208, found 433.1208. HPLC purity 97.27%. White solid, m.p. 118.5-121.7℃, yield 59.21%.

*****4-(5-chloro-2-((1-methyl-1H-pyrazol-5-yl)amino)pyrimidin-4-yl)-N-(2-methylpyridin-4-yl)thiophene-2-carboxamide* (6g).** ^1^H NMR (400 MHz, DMSO-*d*_6_) *δ* (ppm): 10.79 (s, 1H), 9.75 (s, 1H), 8.67 (d, *J* = 1.9 Hz, 1H), 8.63 (s, 2H), 8.36 (d, *J* = 5.6 Hz, 1H), 7.66 (s, 1H), 7.59 (d, *J* = 5.6 Hz, 1H), 7.36 (d, *J* = 1.9 Hz, 1H), 6.29 (d, *J* = 1.9 Hz, 1H), 3.71 (s, 3H), 2.45 (s, 3H). ^13^C NMR (100 MHz, DMSO-*d*_6_) *δ* (ppm): 160.74, 159.70, 159.08, 158.94, 156.68, 150.12, 146.23, 139.90, 138.07, 137.85, 136.45, 130.79, 117.51, 113.50, 112.01, 99.57, 35.97, 24.80. HRMS (ESI)^+^ calculated for C_19_H_17_ClN_7_OS, [M + H] ^+^: *m/z* 426.0898, found 426.0895. HPLC purity 99.35%. Off-White solid, m.p. 120.1-123.2℃, yield 59.03%.

*****4-(5-chloro-2-((1-methyl-1H-pyrazol-5-yl)amino)pyrimidin-4-yl)-N-(pyrazin-2-ylmethyl)thiophene-2-carboxamide* (6h).** ^1^H NMR (400 MHz, DMSO-*d*_6_) *δ* (ppm): 9.72 (s, 1H), 9.45 (t, *J* = 5.9 Hz, 1H), 8.66 (d, *J* = 1.5 Hz, 1H), 8.61 (d, *J* = 2.6 Hz, 2H), 8.56 (d, *J* = 2.6 Hz, 1H), 8.52 (d, *J* = 1.4 Hz, 1H), 8.43 (d, *J* = 1.5 Hz, 1H), 7.36 (d, *J* = 1.9 Hz, 1H), 6.28 (d, *J* = 1.9 Hz, 1H), 4.62 (d, *J* = 5.6 Hz, 2H), 3.69 (s, 3H). ^13^C NMR (100 MHz, DMSO-d6) *δ* (ppm): 161.49, 159.65, 158.89, 156.72, 154.46, 144.50, 144.03, 143.78, 140.08, 138.10, 137.83, 137.73, 135.03, 129.41, 117.43, 99.46, 43.18, 35.95. HRMS (ESI)^+^ calculated for C_18_H_15_ClN_8_OS, [M + H]^+^: *m/z* 427.0851, found 427.0849. HPLC purity 97.67%. White solid, m.p. 194.9-198.9℃, yield 57.43%.

*****4-(5-chloro-2-((1-methyl-1H-pyrazol-5-yl)amino)pyrimidin-4-yl)-N-phenylthiophene-2-carboxamide* (6i).** ^1^H NMR (400 MHz, DMSO-*d*_6_) *δ* (ppm): 10.55 (s, 1H), 9.79 (s, 1H), 8.62 (s, 2H), 8.60 (d, *J* = 1.4 Hz, 1H), 7.75 (d, *J* = 8.0 Hz, 2H), 7.40 – 7.35 (m, 3H), 7.13 (t, *J* = 7.4 Hz, 1H), 6.29 (d, *J* = 2.0 Hz, 1H), 3.71 (s, 3H). ^13^C NMR (100 MHz, DMSO-*d*_6_) *δ* (ppm): 162.78, 159.97, 159.67, 158.93, 156.75, 140.75, 139.02, 138.11, 137.84, 137.76, 135.60, 129.95, 129.19, 124.45, 121.01, 117.47, 99.49, 35.94. HRMS (ESI)^+^ calculated for C_19_H_16_ClN_6_OS, [M + H] ^+^: *m/z* 411.0789, found 411.0785. HPLC purity 98.74%. White solid, m.p. 170.5-173.5℃, yield 62.67%.

*****4-(5-chloro-2-((1-methyl-1H-pyrazol-5-yl)amino)pyrimidin-4-yl)-N-(3-fluorophenyl)thiophene-2-carboxamide* (6j).** ^1^H NMR (400 MHz, DMSO-*d*_6_) *δ* (ppm): 10.65 (s, 1H), 9.74 (s, 1H), 8.63 (s, 1H), 8.62 (s, 1H), 8.61 (s, 1H), 7.71 (m, 1H), 7.54 (d, *J* = 8.0 Hz, 1H), 7.45 – 7.38 (m, 1H), 7.37 (d, *J* = 1.9 Hz, 1H), 7.02-6.91 (m, 1H), 6.29 (d, *J* = 2.0 Hz, 1H), 3.71 (s, 3H). ^13^C NMR (100 MHz, DMSO-*d*_6_) *δ* (ppm): 162.52 (*J* = 240.0 Hz), 160.19, 159.70, 158.94, 156.69, 140.74, 140.24, 138.07, 137.78, 136.00, 130.89, 130.25, 117.49, 116.60, 110.91 (*J* = 21.0 Hz), 107.64 (*J* = 26.0 Hz), 99.53, 35.97. HRMS (ESI)^+^ calculated for C_19_H_15_ClFN_6_OS, [M + H] ^+^: *m/z* 429.0695, found 429.0694. HPLC purity 95.73%. White solid, m.p. 101.2-105.5℃, yield 61.46%.

*****4-(5-chloro-2-((1-methyl-1H-pyrazol-5-yl)amino)pyrimidin-4-yl)-N-(3-chlorophenyl)thiophene-2-carboxamide* (6k).** ^1^H NMR (400 MHz, DMSO-*d*_6_) *δ* (ppm): 10.63 (s, 1H), 9.74 (s, 1H), 8.71 – 8.53 (m, 3H), 7.93 (s, 1H), 7.69 (d, *J* = 8.3 Hz, 1H), 7.41 (t, *J* = 8.1 Hz, 1H), 7.37 (d, *J* = 1.9 Hz, 1H), 7.19 (dd, *J* = 7.9, 2.1 Hz, 1H), 6.29 (d, *J* = 1.9 Hz, 1H), 3.71 (s, 3H). ^13^C NMR (100 MHz, DMSO-*d*_6_) *δ* (ppm): 160.18, 159.69, 158.93, 156.68, 140.53, 140.20, 138.08, 137.85, 137.80, 136.02, 133.48, 130.91, 130.28, 124.12, 120.35, 119.23, 117.49, 99.52, 35.97. HRMS (ESI)^+^ calculated for C_19_H_15_C_l2_N_6_OS, [M + H] ^+^: *m/z* 445.0400, found 445.0400. HPLC purity 97.83%. White solid, m.p. 189.2-192.3℃, yield 60.85%.

*****N-(3-bromophenyl)-4-(5-chloro-2-((1-methyl-1H-pyrazol-5-yl)amino) pyrimidin-4-yl)thiophene-2-carboxamide* (6l).** ^1^H NMR (400 MHz, DMSO-*d*_6_) *δ* 10.64 (s, 1H), 9.74 (s, 1H), 8.62 (d, *J* = 4.2 Hz, 3H), 8.06 (s, 1H), 7.74 (d, *J* = 7.4 Hz, 1H), 7.39 – 7.31 (m, 3H), 6.30 (d, *J* = 1.9 Hz, 1H), 3.71 (s, 3H). ^13^C NMR (100 MHz, DMSO-*d*_6_) *δ* (ppm): 160.17, 159.69, 158.93, 156.69, 140.66, 140.19, 138.08, 137.86, 137.81, 136.03, 131.22, 130.30, 127.01, 123.19, 121.92, 119.62, 117.50, 99.53, 35.94. HRMS (ESI)^+^ calculated for C_19_H_15_BrClN_6_OS, [M + H] ^+^: *m/z* 488.9894, found 488.9893. HPLC purity 98.07%. White solid, m.p. 175.9-180.4℃, yield 57.18%.

*****4-(5-chloro-2-((1-methyl-1H-pyrazol-5-yl)amino) pyrimi-din-4-yl)-N-(3-(trifluoromethyl) phenyl)thiophene-2-carboxamide* (6m).** ^1^H NMR (400 MHz, DMSO-*d*_6_) *δ* (ppm): 10.80 (s, 1H), 9.74 (s, 1H), 8.64 (d, *J* = 4.5 Hz, 3H), 8.20 (d, *J* = 2.4 Hz, 1H), 8.05 (d, *J* = 8.2 Hz, 1H), 7.63 (t, *J* = 8.0 Hz, 1H), 7.49 (d, *J* = 7.7 Hz, 1H), 7.37 (d, *J* = 1.8 Hz, 1H), 6.30 (s, 1H), 3.71 (s, 3H). ^13^C NMR (100 MHz, DMSO-*d*_6_) *δ* (ppm): 160.35, 159.69, 158.94, 156.68, 140.08, 139.88, 138.08, 137.84, 136.13, 130.47, 129.90 (*J* = 32.0 Hz), 124.37, 124.58 (*J* = 271.0 Hz), 120.70, 117.50, 116.94, 99.52, 35.94. HRMS (ESI)^+^ calculated for C_20_H_15_ClF_3_N_6_OS, [M + H] ^+^: *m/z* 479.0663, found 479.0663. HPLC purity 98.62%. White solid, m.p. 116.1-120.3℃, yield 51.35%.

*****4-(5-chloro-2-((1-methyl-1H-pyrazol-5-yl)amino) pyrimidin-4-yl)-N-(3-(trifluoromethoxy)phenyl) thiophene-2-carboxamide* (6n).** ^1^H NMR (400 MHz, DMSO-*d*_6_) *δ* (ppm): 10.74 (s, 1H), 9.74 (s, 1H), 8.63 (d, *J* = 4.2 Hz, 3H), 7.89 (s, 1H), 7.76 (dd, *J* = 8.2, 2.0 Hz, 1H), 7.51 (t, *J* = 8.2 Hz, 1H), 7.36 (d, *J* = 1.9 Hz, 1H), 7.16 – 7.08 (m, 1H), 6.29 (d, *J* = 2.0 Hz, 1H), 3.71 (s, 3H). ^13^C NMR (100 MHz, DMSO-*d*_6_) *δ* (ppm): 160.29, 159.70, 158.94, 156.68, 148.89, 140.75, 140.11, 138.08, 137.84, 137.80, 136.10, 130.96, 130.37, 181.50 (*J* = 255.0 Hz), 119.42, 117.50, 116.48, 112.90, 99.53, 35.94. HRMS (ESI)^+^ calculated for C_20_H_15_ClF_3_N_6_O_2_S, [M + H] ^+^: *m/z* 495.0612, found 495.0612. HPLC purity 98.47%. White solid, m.p. 173.4-177.3℃, yield 55.65%.

*****4-(5-chloro-2-((1-methyl-1H-pyrazol-5-yl)amino)***

***pyrimidin-4-yl)-N-(3-chloro-2-fluorophenyl)thiophene-2-carboxamide* (6o).** ^1^H NMR (400 MHz, DMSO-*d*_6_) *δ* (ppm): 10.62 (s, 1H), 9.76 (s, 1H), 8.69 – 8.54 (m, 3H), 7.61 – 7.52 (m, 1H), 7.52 – 7.40 (m, 1H), 7.37 (t, *J* = 2.1 Hz, 1H), 7.26 (t, *J* = 8.1 Hz, 1H), 6.27 (dd, *J* = 17.1, 1.9 Hz, 1H), 3.70 (d, *J* = 5.2 Hz, 3H). ^13^C NMR (100 MHz, DMSO-*d*_6_) *δ* (ppm): 159.98, 159.66, 158.93, 156.74, 140.74, 139.04, 138.13, 137.84, 137.77, 135.59, 129.98, 129.19, 124.44, 121.01, 117.45, 99.46, 35.97. HRMS (ESI)^+^ calculated for C_19_H_14_C_l2_FN_6_OS, [M + H] ^+^: *m/z* 463.0305, found 463.0305. HPLC purity 97.25%. White solid, m.p. 87.3-90.9℃, yield 60.10%.

*****N-(3-aminophenyl)-4-(5-chloro-2-((1-methyl-1H-pyrazol-5-yl)amino)pyrimidin-4-yl)thiophene-2-carboxamide* (6p).** ^1^H NMR (400 MHz, DMSO-*d*_6_) *δ* (ppm): 10.20 (s, 1H), 9.73 (s, 1H), 8.62 (s, 1H), 8.59 – 8.48 (m, 2H), 7.37 (d, *J* = 1.9 Hz, 1H), 7.05 – 6.95 (m, 2H), 6.85 (d, *J* = 7.9 Hz, 1H), 6.33 (dd, *J* = 7.9, 1.3 Hz, 1H), 6.29 (d, *J* = 1.9 Hz, 1H), 5.12 (s, 2H), 3.70 (s, 3H). ^13^C NMR (100 MHz, DMSO-*d*_6_) *δ* (ppm): 159.68, 158.92, 156.84, 149.48, 141.21, 139.55, 138.10, 137.85, 137.70, 135.31, 129.63, 129.38, 117.49, 110.53, 108.93, 106.68, 99.50, 35.94. HRMS (ESI)^+^ calculated for C_19_H_17_ClN_7_OS, [M + H] ^+^: *m/z* 426.0898, found 426.0895. HPLC purity 96.52%. White solid, m.p. 97.8-101.5℃, yield 49.89%.

*****4-(5-chloro-2-((1-methyl-1H-pyrazol-5-yl)amino)pyrimidin-4-yl)-N-(m-tolyl)thiophene-2-carboxamide* (6q).** ^1^H NMR (400 MHz, DMSO-*d*_6_) *δ* (ppm): 10.42 (s, 1H), 9.73 (s, 1H), 8.63 (s, 1H), 8.60 (d, *J* = 1.4 Hz, 1H), 8.58 (d, *J* = 1.4 Hz, 1H), 7.59 – 7.53 (m, 2H), 7.37 (d, *J* = 1.9 Hz, 1H), 7.25 (t, *J* = 7.8 Hz, 1H), 6.95 (d, *J* = 7.5 Hz, 1H), 6.30 (d, *J* = 2.0 Hz, 1H), 3.71 (s, 3H), 2.32 (s, 3H). ^13^C NMR (100 MHz, DMSO-*d*_6_) *δ* (ppm): 159.90, 159.67, 158.93, 156.79, 140.85, 138.93, 138.37, 138.09, 137.85, 137.76, 135.57, 129.84, 129.03, 125.15, 121.53, 118.17, 117.49, 99.51, 35.94, 21.66. HRMS (ESI)^+^ calculated for C_20_H_18_ClN_6_OS, [M + H] ^+^: *m/z* 425.0946, found 425.0945. HPLC purity 98.31%. White solid, m.p. 155.0-159.2℃, yield 58.35%.

*****4-(5-chloro-2-((1-methyl-1H-pyrazol-5-yl)amino)pyrimidin-4-yl)-N-(3-ethylphenyl)thiophene-2-carboxamide* (6r).** ^1^H NMR (400 MHz, DMSO-*d*_6_) *δ* (ppm): 10.43 (s, 1H), 9.73 (s, 1H), 8.71 – 8.47 (m, 3H), 7.60 – 7.56 (m, 2H), 7.37 (d, *J* = 1.9 Hz, 1H), 7.27 (t, *J* = 7.7 Hz, 1H), 6.98 (d, *J* = 7.6 Hz, 1H), 6.29 (d, *J* = 1.9 Hz, 1H), 3.71 (s, 3H), 2.62 (q, *J* = 7.6 Hz, 2H), 1.20 (t, *J* = 7.6 Hz, 3H). ^13^C NMR (100 MHz, DMSO-*d*_6_) *δ* (ppm): 159.91, 159.66, 158.93, 156.80, 144.74, 140.86, 139.00, 138.10, 137.85, 137.77, 135.56, 129.84, 129.08, 123.98, 120.36, 118.44, 117.49, 99.51, 35.97, 28.73, 15.98. HRMS (ESI)^+^ calculated for C_21_H_20_ClN_6_OS, [M + H] ^+^: *m/z* 439.1102, found 439.1102. HPLC purity 98.49%. White solid, m.p. 127.3-131.2℃, yield 58.75%.

***4-(5-chloro-2-((1-methyl-1H-pyrazol-5-yl)amino)pyrimidin-4-yl) -N-(3-methoxyphenyl)***

***thiophene-2-carboxamide* (6s).** ^1^H NMR (400 MHz, DMSO-*d*_6_) *δ* (ppm): 10.45 (s, 1H), 9.74 (s, 1H), 8.63 (s, 1H), 8.59 (s, 2H), 7.42 (t, *J* = 2.2 Hz, 1H), 7.38 – 7.31 (m, 2H), 7.27 (t, *J* = 8.1 Hz, 1H), 6.74 – 6.68 (m, 1H), 6.29 (d, *J* = 2.0 Hz, 1H), 3.76 (s, 3H), 3.71 (s, 3H). ^13^C NMR (100 MHz, DMSO-*d*_6_) *δ* (ppm): 159.98, 159.93, 159.67, 158.92, 156.77, 140.73, 140.21, 138.11, 137.83, 137.75, 135.68, 129.99, 129.92, 117.51, 113.14, 109.99, 106.62, 99.52, 55.49, 35.98. HRMS (ESI)^+^ calculated for C_20_H_18_ClN_6_O_2_S, [M + H] ^+^: *m/z* 441.0895, found 441.0895. HPLC purity 99.01%. White solid, m.p. 156.2-159.7℃, yield 61.68%.

*****4-(5-chloro-2-((1-methyl-1H-pyrazol-5-yl)amino)pyrimidin-4-yl) -N-(3-(methylthio)phenyl)***

***thiophene-2-carboxamide* (6t).** ^1^H NMR (400 MHz, DMSO-*d*_6_) *δ* (ppm): 10.52 (s, 1H), 9.74 (s, 1H), 8.63 (s, 1H), 8.61 (d, *J* = 1.3 Hz, 1H), 8.59 (d, *J* = 1.4 Hz, 1H), 7.71 (t, *J* = 1.9 Hz, 1H), 7.55 (dd, *J* = 8.1, 1.1 Hz, 1H), 7.36 (d, *J* = 1.9 Hz, 1H), 7.31 (t, *J* = 8.0 Hz, 1H), 7.02 (ddd, *J* = 7.9, 1.9, 0.9 Hz, 1H), 6.29 (d, *J* = 1.9 Hz, 1H), 3.70 (s, 3H), 2.49 (s, 3H). ^13^C NMR (100 MHz, DMSO-*d*_6_) *δ* (ppm): 160.05, 159.67, 158.93, 156.76, 140.58, 139.61, 139.08, 138.09, 137.85, 137.78, 135.77, 130.06, 129.71, 121.78, 117.92, 117.50, 117.36, 99.53, 35.97, 15.09. HRMS (ESI)^+^ calculated for C_20_H_18_ClN_6_OS_2_, [M + H] ^+^: *m/z* 457.0667, found 457.0667. HPLC purity 99.06%. White solid, m.p. 158.3-162.1℃, yield 62.35%.

*****4-(5-chloro-2-((1-methyl-1H-pyrazol-5-yl)amino)***

***pyrimi-din-4-yl)-N-(3-isopropoxyphenyl)thiophene-2-carboxamide* (6u).** ^1^H NMR (400 MHz, DMSO-*d*_6_) *δ* (ppm): 10.44 (s, 1H), 9.74 (s, 1H), 8.63 (s, 1H), 8.59 (t, *J* = 1.6 Hz, 2H), 7.41 (t, *J* = 2.2 Hz, 1H), 7.37 (d, *J* = 2.0 Hz, 1H), 7.29 (d, *J* = 8.3 Hz, 1H), 7.24 (t, *J* = 8.0 Hz, 1H), 6.73-6.63 (m, 1H), 6.30 (d, *J* = 1.9 Hz, 1H), 4.57 (hept, *J* = 6.0 Hz, 1H), 3.71 (s, 3H), 1.29 (d, *J* = 6.1 Hz, 6H). ^13^C NMR (100 MHz, DMSO-*d*_6_) *δ* (ppm): 159.97, 159.66, 158.93, 158.13, 156.77, 140.76, 140.22, 138.09, 137.85, 137.75, 135.63, 129.97, 129.93, 117.50, 112.96, 111.77, 108.25, 99.52, 69.72, 35.94, 22.33. HRMS (ESI)^+^ calculated for C_22_H_22_ClN_6_O_2_S, [M + H] ^+^: *m/z* 469.1208, found 469.1208. HPLC purity 99.31%. White solid, m.p. 104.1-108.5℃, yield 61.92%.

*****4-(5-chloro-2-((1-methyl-1H-pyrazol-5-yl)amino)***

***pyrimidin-4-yl)-N-(3-((dimethylamino) methyl)phenyl)***

***thiophene-2-carboxamide* (6v).** ^1^H NMR (400 MHz, DMSO-*d*_6_) *δ* (ppm): 10.48 (s, 1H), 9.74 (s, 1H), 8.63 (d, *J* = 3.1 Hz, 2H), 8.58 (d, *J* = 1.4 Hz, 1H), 7.70 (d, *J* = 6.1 Hz, 2H), 7.37 (d, *J* = 1.9 Hz, 1H), 7.33-7.28 (m, 1H), 7.04 (d, *J* = 7.6 Hz, 1H), 6.30 (d, *J* = 2.0 Hz, 1H), 3.71 (s, 3H), 3.34 (s, 2H), 2.17 (s, 6H). ^13^C NMR (100 MHz, DMSO-*d*_6_) *δ* (ppm): 159.91, 159.66, 158.92, 156.80, 140.85, 140.04, 139.01, 138.10, 137.84, 137.80, 135.60, 129.88, 128.92, 124.77, 121.12, 119.54, 117.49, 99.49, 63.85, 45.43, 35.98, 35.95. HRMS (ESI)^+^ calculated for C_22_H_23_ClN_7_OS, [M + H] ^+^: *m/z* 468.1368, found 468.1368. HPLC purity 97.98%. White solid, m.p. 86.3-89.9℃, yield 54.77%.

*****4-(5-chloro-2-((1-methyl-1H-pyrazol-5-yl)amino)***

***pyrimidin-4-yl)-N-(4-fluorophenyl)thiophene-2-carboxamide* (6w).** ^1^H NMR (400 MHz, DMSO-*d*_6_) *δ* (ppm): 10.55 (s, 1H), 9.73 (s, 1H), 8.63 (s, 1H), 8.59 (d, *J* = 2.3 Hz, 2H), 7.79 – 7.73 (m, 2H), 7.37 (d, *J* = 2.0 Hz, 1H), 7.25 – 7.19 (m, 2H), 6.29 (d, *J* = 1.9 Hz, 1H), 3.70 (s, 3H). ^13^C NMR (100 MHz, DMSO-*d*_6_) *δ* (ppm): 159.92, 159.68, 158.96 (*J* = 239 Hz), 158.92, 157.76, 156.74, 140.52, 138.09, 137.76, 135.64, 135.35, 129.95, 122.90 (*J* = 8 Hz), 122.90 (*J* = 8 Hz), 117.50, 115.80 (*J* = 22 Hz), 115.80 (*J* = 22 Hz), 99.52, 35.94. HRMS (ESI)^+^ calculated for C_19_H_15_ClFN_6_OS, [M + H] ^+^: *m/z* 429.0695, found 429.0695. HPLC purity 99.44%. White solid, m.p. 142.9-147.2℃, yield 63.21%.

**4-(5-chloro-2-((1-methyl-1H-pyrazol-5-yl)amino)**

**pyrimidin-4-yl)-N-(4-chlorophenyl)thiophene-2-carboxamide (6x).** ^1^H NMR (400 MHz, DMSO-*d*_6_) *δ* (ppm): 10.63 (s, 1H), 9.74 (s, 1H), 8.62 (d, *J* = 8.9 Hz, 3H), 7.79 (d, *J* = 8.2 Hz, 2H), 7.43 (d, *J* = 8.3 Hz, 2H), 7.37 (s, 1H), 6.29 (s, 1H), 3.71 (s, 3H). ^13^C NMR (100 MHz, DMSO-*d*_6_) *δ* (ppm): 160.05, 159.68, 158.93, 156.72, 140.38, 138.08, 138.02, 137.85, 137.78, 135.83, 130.17, 129.11, 128.09, 122.50, 117.49, 99.52, 35.94. HRMS (ESI)^+^ calculated for C_19_H_15_C_l2_N_6_OS, [M + H] ^+^: *m/z* 445.0400, found 445.0400. HPLC purity 98.62%. White solid, m.p. 105.2-108.9℃, yield 61.29%.

 ***4-(5-chloro-2-((1-methyl-1H-pyrazol-5-yl)amino) pyrimidin-4-yl)-N-(4-(trifluoromethyl)phenyl) thiophene-2-carboxamide* (6y).** ^1^H NMR (400 MHz, DMSO-*d*_6_) *δ* (ppm): 10.84 (s, 1H), 9.73 (d, *J* = 19.6 Hz, 1H), 8.68 – 8.56 (m, 3H), 7.99 (d, *J* = 8.5 Hz, 2H), 7.74 (d, *J* = 8.6 Hz, 2H), 7.35 (dd, *J* = 5.5, 1.9 Hz, 1H), 6.27 (dd, *J* = 12.1, 1.9 Hz, 1H), 3.69 (d, *J* = 8.1 Hz, 3H). ^13^C NMR (100 MHz, DMSO-*d*_6_) *δ* (ppm): 160.41, 159.69, 158.93, 156.67, 142.76, 140.10, 138.08, 137.83, 136.19, 130.58, 126.46, 124.83 (*J* = 268.0 Hz), 124.34 (*J* = 31.0 Hz), 120.75, 117.50, 99.52, 66.82, 35.97. HRMS (ESI)^+^ calculated for C_20_H_15_ClF_3_N_6_OS, [M + H] ^+^: *m/z* 479.0663, found 479.0663. HPLC purity 99.24%. White solid, m.p. 119.5-122.6℃, yield 66.32%.

*****4-(5-chloro-2-((1-methyl-1H-pyrazol-5-yl)ami-no)***

***pyrimidin-4-yl)-N-(4-(trifluoromethoxy) phenyl)***

***thiophene-2-carboxamide* (6z).** ^1^H NMR (400 MHz, DMSO-*d*_6_) *δ* (ppm): 10.68 (s, 1H), 9.74 (s, 1H), 8.62 (d, *J* = 6.6 Hz, 3H), 7.87 (d, *J* = 9.1 Hz, 2H), 7.44 – 7.34 (m, 3H), 6.29 (d, *J* = 1.9 Hz, 1H), 3.71 (s, 3H). ^13^C NMR (100 MHz, DMSO-*d*_6_) *δ* (ppm): 160.12, 159.68, 158.93, 156.71, 144.55, 140.29, 138.24, 138.08, 137.85, 137.79, 135.88, 130.23, 122.36, 122.03, 120.62 (*J* = 31.0 Hz), 117.50, 99.52, 35.96. HRMS (ESI)^+^ calculated for C_20_H_14_ClF_3_N_6_O_2_S, [M + H] ^+^: *m/z* 495.0612, found 495.0612. HPLC purity 99.78%. White solid, m.p. 128.3-130.5℃, yield 58.93%.

*****4-(5-chloro-2-((1-methyl-1H-pyrazol-5-yl)amino)***

***pyrimidin-4-yl)-N-(p-tolyl)thiophene-2-carboxamide* (6aa).** ^1^H NMR (400 MHz, DMSO-*d*_6_) *δ* (ppm): 10.47 (s, 1H), 9.74 (s, 1H), 8.61 (d, *J* = 6.6 Hz, 2H), 8.57 (s, 1H), 7.63 (d, *J* = 8.4 Hz, 2H), 7.36 (d, *J* = 2.0 Hz, 1H), 7.17 (d, *J* = 8.2 Hz, 2H), 6.30 (d, *J* = 1.9 Hz, 1H), 3.71 (s, 3H), 2.29 (s, 3H). ^13^C NMR (100 MHz, DMSO-*d*_6_) *δ* (ppm): 159.81, 159.65, 158.92, 156.80, 140.89, 138.10, 137.85, 137.75, 136.46, 135.43, 133.47, 129.78, 129.57, 121.06, 117.50, 99.53, 35.97, 20.95. HRMS (ESI)^+^ calculated for C_20_H_18_ClN_6_OS, [M + H] ^+^: *m/z* 425.0946, found 425.0945. HPLC purity 99.23%. Off-White solid, m.p. 161.1-164.3℃, yield 63.45%.

*****4-(5-chloro-2-((1-methyl-1H-pyrazol-5-yl)amino)***

***pyri-midin-4-yl)-N-(4-methoxyphenyl)thiophene-2-carboxamide* (6ab).** ^1^H NMR (400 MHz, DMSO-*d*_6_) *δ* (ppm): 10.37 (s, 1H), 9.72 (s, 1H), 8.62 (s, 1H), 8.59 – 8.38 (m, 2H), 7.63 (d, *J* = 9.1 Hz, 2H), 7.37 (d, *J* = 1.9 Hz, 1H), 6.95 (d, *J* = 9.1 Hz, 2H), 6.29 (d, *J* = 1.9 Hz, 1H), 3.75 (s, 3H), 3.70 (s, 3H). ^13^C NMR (100 MHz, DMSO-*d*_6_) *δ* (ppm): 159.67, 159.62, 158.92, 156.79, 156.26, 140.94, 138.09, 137.85, 137.73, 135.31, 131.96, 129.55, 122.66, 117.49, 114.34, 99.51, 55.65, 35.96. HRMS (ESI)^+^ calculated for C_20_H_18_ClN_6_O_2_S, [M + H] ^+^: *m/z* 441.0895, found 441.0894. HPLC purity 97.58%. Off-White solid, m.p. 180.6-184.1℃, yield 63.35%.

*****4-(5-chloro-2-((1-methyl-1H-pyrazol-5-yl)amino)pyri-midin-4-yl)-N-(4-(methylthio)phenyl)thiophene-2-carboxamide* (6ac).** ^1^H NMR (400 MHz, DMSO-*d*_6_) *δ* (ppm): 10.50 (s, 1H), 9.73 (s, 1H), 8.62 (s, 1H), 8.59 (s, 2H), 7.72 (s, 1H), 7.70 (s, 1H), 7.36 (d, *J* = 2.0 Hz, 1H), 7.30 (d, *J* = 2.1 Hz, 1H), 7.28 (d, *J* = 2.1 Hz, 1H), 6.29 (d, *J* = 1.9 Hz, 1H), 3.70 (s, 3H), 2.47 (s, 3H). ^13^C NMR (100 MHz, DMSO-*d*_6_) *δ* (ppm): 159.85, 159.67, 158.92, 156.76, 140.69, 138.09, 137.85, 137.76, 136.40, 135.60, 133.28, 129.88, 127.30, 121.62, 117.49, 99.52, 35.94, 15.82. HRMS (ESI)^+^ calculated for C_20_H_18_ClN_6_OS_2_, [M + H] ^+^: *m/z* 457.0667, found 457.0667. HPLC purity 99.39%. Off-White solid, m.p. 155.1-159.2℃, yield 59.03%.

*****4-(5-chloro-2-((1-methyl-1H-pyrazol-5-yl)amino)***

***pyri-midin-4-yl)-N-(4-ethoxyphenyl)thiophene-2-carboxamide* (6ad).** ^1^H NMR (400 MHz, DMSO-*d*_6_) *δ* (ppm): 10.38 (s, 1H), 9.72 (s, 1H), 8.62 (s, 1H), 8.56 (s, 2H), 7.64 – 7.60 (m, 2H), 7.36 (d, *J* = 2.0 Hz, 1H), 6.95 – 6.91 (m, 2H), 6.29 (d, *J* = 1.9 Hz, 1H), 4.01 (q, *J* = 6.9 Hz, 2H), 3.70 (s, 3H), 1.33 (t, *J* = 7.0 Hz, 3H). ^13^C NMR (100 MHz, DMSO-*d*_6_) *δ* (ppm): 159.65, 159.61, 158.92, 156.81, 155.52, 140.96, 138.10, 137.85, 137.73, 135.27, 131.87, 129.58, 122.66, 117.49, 114.83, 99.51, 63.58, 35.94, 15.17. HRMS (ESI)^+^ calculated for C_21_H_20_ClN_6_O_2_S, [M + H] ^+^: *m/z* 455.1051, found 455.1051. HPLC purity 98.90%. Off-White solid, m.p. 165.0-169.2℃, yield 54.37%.

*****4-(5-chloro-2-((1-methyl-1H-pyrazol-5-yl)amino) pyrimidin-4-yl)-N-(4-isopropoxyphenyl)thioph-ene-2-carboxamide* (6ae).** ^1^H NMR (400 MHz, DMSO-*d*_6_) *δ* (ppm): 10.37 (s, 1H), 9.73 (s, 1H), 8.62 (s, 1H), 8.56 (s, 2H), 7.62 (s, 1H), 7.60 (s, 1H), 7.37 (d, *J* = 1.9 Hz, 1H), 6.93 (d, *J* = 2.2 Hz, 1H), 6.91 (d, *J* = 2.2 Hz, 1H), 6.30 (d, *J* = 2.0 Hz, 1H), 4.58 (p, *J* = 6.0 Hz, 1H), 3.71 (s, 3H), 1.27 (s, 3H), 1.26 (s, 3H). ^13^C NMR (100 MHz, DMSO-*d*_6_) *δ* (ppm): 159.66, 159.60, 158.92, 156.80, 154.41, 140.96, 138.09, 137.85, 137.72, 135.28, 131.79, 129.55, 122.69, 117.49, 116.19, 99.50, 69.83, 35.94, 22.33. HRMS (ESI)^+^ calculated for C_22_H_22_ClN_6_O_2_S, [M + H] ^+^: *m/z* 469.1208, found 469.1208. HPLC purity 99.54%. Off-White solid, m.p. 118.5-121.8℃, yield 57.87%.

*****4-(5-chloro-2-((1-methyl-1H-pyrazol-5-yl)amino)***

***pyrimidin-4-yl)-N-(4-((dimethylamino) methyl)***

***phenyl)thiophene-2-carboxamide* (6af).** ^1^H NMR (400 MHz, DMSO-*d*_6_) *δ* (ppm): 10.46 (s, 1H), 9.73 (s, 1H), 8.63 (s, 1H), 8.58 (s, 2H), 7.68 (d, *J* = 8.4 Hz, 2H), 7.36 (d, *J* = 1.9 Hz, 1H), 7.28 (d, *J* = 8.5 Hz, 2H), 6.29 (d, *J* = 1.9 Hz, 1H), 3.70 (s, 3H), 3.37 (s, 2H), 2.15 (s, 6H). ^13^C NMR (100 MHz, DMSO-*d*_6_) *δ* (ppm): 159.88, 159.68, 158.93, 156.79, 140.80, 138.08, 137.84, 137.75, 135.56, 129.84, 129.61, 120.77, 117.49, 99.51, 63.31, 45.28, 35.97, 35.94. HRMS (ESI)^+^ calculated for C_22_H_23_ClN_7_OS, [M + H] ^+^: *m/z* 468.1368, found 468.1365. HPLC purity 98.65%. White solid, m.p. 185.6-188.7℃, yield 54.67%.

*****4-(5-chloro-2-((1-methyl-1H-pyrazol-5-yl)amino)pyri-midin-4-yl)-N-(3,4-dimethoxyphenyl)thiophene-2-carboxamide* (6ag).** ^1^H NMR (400 MHz, DMSO-*d*_6_) *δ* (ppm): 10.36 (s, 1H), 9.72 (s, 1H), 8.62 (s, 1H), 8.57 (d, *J* = 1.8 Hz, 2H), 7.41 (d, *J* = 2.4 Hz, 1H), 7.37 (d, *J* = 2.0 Hz, 1H), 7.29 (dd, *J* = 8.7, 2.4 Hz, 1H), 6.95 (d, *J* = 8.8 Hz, 1H), 6.29 (d, *J* = 2.0 Hz, 1H), 3.77 (s, 3H), 3.75 (s, 3H), 3.70 (s, 3H). ^13^C NMR (100 MHz, DMSO-*d*_6_) *δ* (ppm): 159.63, 158.93, 156.80, 148.94, 145.87, 140.96, 138.10, 137.87, 137.85, 137.75, 135.38, 132.41, 129.53, 117.50, 113.02, 112.35, 106.04, 99.54, 56.18, 55.90, 35.92. HRMS (ESI)^+^ calculated for C_21_H_20_ClN_6_O_3_S, [M + H] ^+^: *m/z* 471.1001, found 471.1001. HPLC purity 97.71%. White solid, m.p. 96.8-100.7℃, yield 50.81%.

**The Structural Characterization of Compound 9a-i：**

*****4-(5-chloro-2-((1-methyl-1H-pyrazol-3-yl)amino) pyrimidin-4-yl)-N-(4-methoxyphenyl)thiophene-2-carboxamide* (9a).** ^1^H NMR (400 MHz, DMSO-*d*6) *δ* (ppm): 10.39 (s, 1H), 10.04 (s, 1H), 8.57 (d, *J* = 1.7 Hz, 3H), 7.63 (d, *J* = 9.0 Hz, 2H), 7.58 (d, *J* = 2.2 Hz, 1H), 6.94 (d, *J* = 9.1 Hz, 2H), 6.61 (d, *J* = 2.2 Hz, 1H), 3.75 (s, 6H). ^13^C NMR (100 MHz, DMSO-*d*6) *δ* (ppm): 159.68, 159.39, 158.30, 156.42, 156.24, 148.04, 140.89, 138.11, 135.05, 132.00, 131.39, 129.62, 122.66, 116.23, 114.32, 97.33, 55.69, 38.74. HRMS (ESI)^+^ calculated for C_20_H_17_ClN_6_O_2_S, [M + H] ^+^: *m/z* 441.0895, found 441.0895. HPLC purity 98.60%. Off-White solid, m.p. 219.7-224.3℃, yield 62.31%.

***4-(5-chloro-2-((1-methyl-1H-pyrazol-4-yl)amino)***

***pyrimidin-4-yl)-N-(4-methoxyphenyl)thiophene-2-carboxamide* (9b).** ^1^H NMR (400 MHz, DMSO-d_6_) *δ* (ppm): 10.41 (s, 1H), 10.03 (s, 1H), 8.58 (d, *J* = 5.0 Hz, 3H), 7.64 (d, *J* = 9.0 Hz, 2H), 7.58 (d, *J* = 2.2 Hz, 1H), 6.94 (d, *J* = 9.0 Hz, 2H), 6.61 (d, *J* = 2.2 Hz, 1H), 3.75 (s, 6H). ^13^C NMR (100 MHz, DMSO-d_6_) *δ* (ppm): 159.68, 159.38, 158.30, 156.44, 156.23, 148.04, 140.90, 138.11, 135.04, 132.01, 131.40, 129.65, 122.67, 116.23, 114.31, 97.33, 55.65, 38.74. HRMS (ESI)^+^ calculated for C_20_H_18_ClN_6_O_2_S, [M + H] ^+^: *m/z* 441.0895, found 441.0895. HPLC purity 98.20%. White solid, m.p.223.4-226.8℃, yield 64.24%.

***4-(5-chloro-2-((1,3-dimethyl-1H-pyrazol-4-yl) amino)pyrimidin-4-yl)-N-(4-methoxyphenyl)***

***thiophene-2-carboxamide* (9c).** ^1^H NMR (400 MHz, DMSO-*d*_6_) *δ* (ppm): 10.41 (s, 1H), 9.11 (s, 1H), 8.59 (d, *J* = 3.3 Hz, 2H), 8.52 (s, 1H), 8.00-7.79 (d, 1H), 7.65 (d, *J* = 8.4 Hz, 2H), 6.95 (d, *J* = 2.2 Hz, 1H), 6.93 (d, *J* = 2.3 Hz, 1H), 3.75 (s, 6H), 2.13 (s, 3H). ^13^C NMR (100 MHz, DMSO-*d*_6_) *δ* (ppm): 159.68, 159.32, 156.21, 140.81, 138.13, 137.40, 135.01, 132.07, 129.72, 122.59, 122.54, 119.64, 115.46, 114.33, 55.64, 38.90, 11.71. HRMS (ESI)^+^ calculated for C_21_H_20_ClN_6_O_2_S, [M + H] ^+^: *m/z* 455.1051, found 455.1051. HPLC purity 98.19%. White solid, m.p. 119.1-203.1℃, yield 49.90%.

***4-(5-chloro-2-((1,3,5-trimethyl-1H-pyrazol-4-yl) amino)pyrimidin-4-yl)-N-(4-methoxyphenyl)thio-phene-2-carboxamide* (9d).** ^1^H NMR (400 MHz, DMSO-*d*_6_) *δ* (ppm): 10.38 (s, 1H), 8.71 (s, 1H), 8.52 (s, 2H), 8.42 (s, 1H), 7.63 (d, *J* = 8.5 Hz, 2H), 6.94 (d, *J* = 9.1 Hz, 2H), 3.75 (s, 3H), 3.64 (s, 3H), 2.06 (s, 3H), 1.96 (s, 3H). ^13^C NMR (100 MHz, DMSO-*d*_6_) *δ* (ppm): 160.86, 159.67, 159.31, 156.21, 146.47, 142.65, 140.72, 138.08, 134.78, 132.05, 129.80, 122.56, 116.90, 115.42, 114.33, 55.64, 36.50, 11.87, 9.65. HRMS (ESI)^+^ calculated for C_22_H_22_ClN_6_O_2_S, [M + H] ^+^: *m/z* 469.1208, found 469.1208. HPLC purity 99.01%. Off-White solid, m.p. 204.7-209.0℃, yield 50.09%.

***4-(5-chloro-2-(phenylamino)pyrimidin-4-yl)-N-(4-methoxy-phenyl)thiophene-2-carboxamide* (9e).** ^1^H NMR (400 MHz, DMSO-*d*_6_) *δ* (ppm): 10.33 (s, 1H), 9.51 (s, 1H), 8.50 (s, 1H), 8.43 (s, 1H), 8.40 (s, 1H), 7.94 (d, *J* = 2.0 Hz, 1H), 7.66 – 7.62 (m, 2H), 7.58 (d, *J* = 7.8 Hz, 1H), 7.21 (t, *J* = 7.8 Hz, 1H), 6.97 – 6.93 (m, 2H), 6.84 (d, *J* = 7.4 Hz, 1H), 3.75 (s, 3H), 3.36 (s, 2H), 2.40 (s, 3H), 2.12 (s, 6H). ^13^C NMR (100 MHz, DMSO-*d*_6_) *δ* (ppm): 159.69, 159.28, 158.59, 156.47, 156.20, 140.97, 140.46, 137.99, 135.15, 135.10, 132.10, 129.86, 129.10, 122.64, 122.38, 119.60, 116.66, 114.33, 114.26, 55.72. HRMS (ESI)^+^ calculated for C_22_H_18_ClN_4_O_2_S, [M + H] ^+^: *m/z* 437.0834, found 437.0833. HPLC purity 99.50%. White solid, m.p. 245.3-249.1℃, yield 48.99%.

***4-(5-chloro-2-((4-((dimethylamino) methyl)***

***phenyl)amino)pyrimidin-4-yl)-N-(4-methoxyphenyl)thiophene-2-carboxamide* (9f).** ^1^H NMR (400 MHz, DMSO-*d*_6_) *δ* (ppm): 10.42 (s, 1H), 9.92 (s, 1H), 8.65 – 8.60 (m, 3H), 7.74 (d, *J* = 8.3 Hz, 2H), 7.65 (d, *J* = 9.1 Hz, 2H), 7.26 (d, *J* = 8.2 Hz, 2H), 6.95 (d, *J* = 9.0 Hz, 2H), 3.76 (s, 3H), 3.44 (s, 2H), 2.20 (s, 6H). ^13^C NMR (100 MHz, DMSO-*d*_6_) *δ* (ppm): 159.66, 159.33, 158.58, 156.39, 156.24, 140.91, 139.50, 137.97, 135.20, 132.03, 129.84, 129.72, 122.63, 119.36, 116.57, 114.33, 63.04, 55.65, 44.92. HRMS (ESI)^+^ calculated for C_25_H_24_ClN_5_O_2_S, [M + H] ^+^: *m/z* 494.1412, found 494.1412. HPLC purity 97.98%. White solid, m.p. 204.9-209.0℃, yield 63.24%.

***4-(5-chloro-2-((3-((dimethylamino)methyl)***

***phenyl)amino)pyrimidin-4-yl)-N-(4-methoxyphenyl)thiophene-2-carboxamide* (9i).** ^1^H NMR (400 MHz, DMSO-*d*_6_) *δ* (ppm): 10.42 (s, 1H), 9.90 (s, 1H), 8.68 (d, *J* = 1.4 Hz, 1H), 8.65 (s, 1H), 8.64 (s, 1H), 7.92 (s, 1H), 7.66 (d, *J* = 2.2 Hz, 1H), 7.64 (d, *J* = 2.2 Hz, 1H), 7.59 – 7.55 (m, 1H), 7.26 (t, *J* = 7.8 Hz, 1H), 6.97 – 6.91 (m, 3H), 3.76 (s, 3H), 3.41 (s, 2H), 2.15 (s, 6H). ^13^C NMR (100 MHz, DMSO-*d*_6_) *δ* (ppm): 159.67, 159.53, 158.54, 156.21, 156.01, 140.91, 140.41, 139.61, 138.07, 135.19, 132.06, 129.75, 128.81, 122.87, 122.55, 119.87, 118.35, 116.49, 114.32, 64.03, 55.64, 45.33. HRMS (ESI)^+^ calculated for C_25_H_25_ClN_5_O_2_S, [M + H] ^+^: *m/z* 494.1412, found 494.1412. HPLC purity 96.11%. White solid, m.p. 152.9-157.1℃, yield 58.90%.

***4-(5-chloro-2-((3-(dimethylamino)phenyl)amin-o)pyrimidin-4-yl)-N-(4-methoxyphenyl)thio-phene-2-carboxamide* (9h).** ^1^H NMR (400 MHz, DMSO-*d*_6_) *δ* (ppm): 10.38 (s, 1H), 9.71 (s, 1H), 8.65 – 8.61 (m, 2H), 8.61 (d, *J* = 1.3 Hz, 1H), 7.64 (d, *J* = 2.2 Hz, 1H), 7.63 (d, *J* = 2.2 Hz, 1H), 7.37 (s, 1H), 7.10 (t, *J* = 8.0 Hz, 1H), 7.04 (d, *J* = 8.4 Hz, 1H), 6.97 – 6.93 (m, 2H), 6.38 (dd, *J* = 8.0, 1.5 Hz, 1H), 3.75 (s, 3H), 2.86 (s, 6H). ^13^C NMR (100 MHz, DMSO-*d*_6_) *δ* (ppm): 159.62, 159.33, 158.69, 156.24, 156.17, 151.39, 141.19, 140.81, 138.11, 134.97, 132.02, 129.82, 129.37, 122.56, 116.32, 114.35, 108.08, 107.16, 103.77, 55.69, 55.65. HRMS (ESI)^+^ calculated for C_24_H_24_ClN_5_O_2_S, [M + H] ^+^: *m/z* 480.1255, found 480.1255. HPLC purity 96.06%. White solid, m.p. 155.7-189.1℃, yield 61.56%.

***4-(5-chloro-2-((3-(dimethyl-carbamoyl)phenyl)***

***amino)pyrimidin-4-yl)-N-(4-methoxyphenyl)***

***thiophene-2-carboxamide* (9i).** ^1^H NMR (400 MHz, DMSO-*d*_6_) *δ* (ppm): 10.36 (s, 1H), 10.07 (s, 1H), 8.67 (s, 2H), 8.60 (s, 1H), 7.97 (s, 1H), 7.74 (d, *J* = 8.0 Hz, 1H), 7.66 – 7.61 (m, 2H), 7.37 (t, *J* = 7.9 Hz, 1H), 7.02 (d, *J* = 7.7 Hz, 1H), 6.97 – 6.93 (m, 2H), 3.75 (s, 3H), 2.92 (s, 6H). ^13^C NMR (100 MHz, DMSO-*d*_6_) *δ* (ppm): 170.68, 159.62, 159.53, 158.45, 156.31, 156.16, 140.97, 140.48, 137.95, 137.17, 135.29, 131.93, 129.53, 128.99, 122.85, 120.76, 120.38, 118.18, 116.98, 114.30, 55.69, 55.64, 35.22. HRMS (ESI)^+^ calculated for C_25_H_23_ClN_5_O_3_S, [M + H] ^+^: *m/z* 508.1205, found 508.1205. HPLC purity 97.13%. Off-White solid, m.p. 168.2-172.5℃, yield 59.14%.

**The Structural Characterization of Compound 15a-e:**

***4-(2-((3-((dimethylamino) methyl) phenyl)***

***amino)pyrimidin-4-yl)-N-(4-methoxy-phenyl)thiophene-2-carboxamide* (15a).** ^1^H NMR (400 MHz, DMSO-*d*_6_) *δ* (ppm): 10.37 (s, 1H), 9.67 (s, 1H), 8.66 (d, *J* = 1.3 Hz, 1H), 8.62 – 8.56 (m, 2H), 8.01 (s, 1H), 7.65 (dd, *J* = 9.7, 2.9 Hz, 3H), 7.29 – 7.23 (m, 2H), 6.98 – 6.93 (m, 2H), 6.90 (d, *J* = 7.5 Hz, 1H), 3.76 (s, 3H), 3.47 (s, 2H), 2.20 (s, 6H). ^13^C NMR (100 MHz, DMSO-d_6_) *δ* (ppm): 160.65, 159.78, 159.55, 156.19, 149.12, 141.79, 140.95, 140.93, 139.46, 132.16, 132.13, 128.77, 127.79, 122.34, 119.75, 118.19, 114.38, 108.25, 64.08, 55.69, 55.64, 45.37. HRMS (ESI)^+^ calculated for C_25_H_26_N_5_O_2_S, [M + H] ^+^: *m/z* 460.1802, found 460.1802. HPLC purity 99.83%. White solid, m.p. 161.9-165.2℃, yield 63.54%.

***4-(2-((3-((dimethyl-amino)methyl)phenyl)***

***amino)-5-methylpyri-midin-4-yl)-N-(4-methoxyphenyl)thiophene-2-carboxamide* (15b).** ^1^H NMR (400 MHz, DMSO-*d*_6_) *δ* 10.35 (s, 1H), 9.51 (s, 1H), 8.51 (s, 1H), 8.43 (s, 1H), 8.40 (s, 1H), 7.95 (s, 1H), 7.65 (d, *J* = 2.1 Hz, 1H), 7.64 (d, *J* = 2.3 Hz, 1H), 7.59 (d, *J* = 10.2 Hz, 1H), 7.22 (t, *J* = 7.8 Hz, 1H), 6.95 (d, *J* = 9.0 Hz, 2H), 6.85 (d, *J* = 7.5 Hz, 1H), 3.76 (s, 3H), 3.37 (s, 2H), 2.40 (s, 3H), 2.12 (s, 6H). ^13^C NMR (100 MHz, DMSO-*d*_6_) *δ* (ppm): 165.83, 165.72, 164.57, 163.81, 163.39, 160.95, 145.98, 145.48, 144.39, 138.15, 138.02, 136.83, 134.72, 134.61, 133.50, 127.37, 126.68, 124.02, 122.57, 122.34, 119.08, 68.99, 60.43, 50.20, 50.14, 21.79. HRMS (ESI)^+^ calculated for C_26_H_28_N_5_O_2_S, [M + H] ^+^: *m/z* 474.1958 , found 474.1958. HPLC purity 95.02%. White solid, m.p. 142.8-146.2℃, yield 57.89%.

***4-(2-((3-((dimethyl-amino)methyl)phenyl)amino)***

***-5-fluoropyri-midin-4-yl)-N-(4-methoxyphenyl)***

***thiophene-2-carboxamide* (15c).** ^1^H NMR (400 MHz, DMSO-*d*_6_) *δ* (ppm): 10.48 (s, 1H), 9.74 (s, 1H), 8.69 – 8.64 (m, 2H), 8.61 (s, 1H), 7.97 (s, 1H), 7.66 (d, *J* = 9.0 Hz, 2H), 7.57 (d, *J* = 8.9 Hz, 1H), 7.25 (t, *J* = 7.8 Hz, 1H), 6.98 – 6.93 (m, 2H), 6.89 (d, *J* = 7.5 Hz, 1H), 3.76 (s, 3H), 3.40 (s, 2H), 2.15 (s, 6H). ^13^C NMR (100 MHz, DMSO-*d*_6_) *δ* (ppm): 159.67, 156.91, 156.22, 150.69, 148.35, 148.17, 148.10, 146.36, 146.25, 141.84, 140.89, 139.81, 135.78, 134.79, 132.07, 128.78, 122.49, 119.20, 117.77, 114.31, 64.18, 55.71, 45.49, 45.44. HRMS (ESI)^+^ calculated for C_25_H_25_FN_5_O_2_S, [M + H] ^+^: *m/z* 478.1708, found 478.1709. HPLC purity 98.01%. Off-White solid, m.p. 160.4-164.2℃, yield 68.53%.

***4-(5-chloro-2-((3-((dimethylamino)methyl)***

***phenyl)amino)pyri- midin-4-yl)-N-(4-methoxyphenyl)furan-2-carboxamide* (15d).** ^1^H NMR (400 MHz, DMSO-*d*_6_) *δ* (ppm): 10.35 (s, 1H), 9.87 (s, 1H), 8.82 (s, 1H), 8.61 (s, 1H), 7.97 (s, 1H), 7.90 (s, 1H), 7.717.67 (m, *J* = 2.3 Hz, 1H), 7.67-7.65 (m, *J* = 2.3 Hz, 1H), 7.56 (d, *J* = 8.0 Hz, 1H), 7.27 (t, *J* = 7.8 Hz, 1H), 6.95 (s, 1H), 6.93 (s, 1H), 6.91 (s, 1H), 3.75 (s, 3H), 3.42 (s, 2H), 2.19 (s, 6H). ^13^C NMR (100 MHz, DMSO-*d*_6_) *δ* (ppm): 164.02, 163.90, 163.22, 161.00, 160.76, 158.79, 153.32, 152.84, 152.68, 145.15, 144.44, 136.55, 133.66, 129.92, 127.60, 127.33, 124.50, 123.15, 121.31, 119.06, 118.86, 68.83, 60.39, 50.09, 34.23. HRMS (ESI)^+^ calculated for C_25_H_25_ClN_5_O_3_, [M + H] ^+^: *m/z* 478.1640, found 478.1640. HPLC purity 96.38%. Off-White solid, m.p. 175.8-189.6℃, yield 51.53%.

***4-(5-chloro-2-((3-((dimethylamino)methyl)***

***phenyl)amino)pyri-midin-4-yl)-N-(4-methoxyphenyl)-1H-pyrrole-2-carboxamide* (15e).** ^1^H NMR (400 MHz, DMSO-*d*_6_) *δ* (ppm): 12.33 (d, *J* = 3.1 Hz, 1H), 10.04 (s, 1H), 9.64 (s, 1H), 8.47 (s, 1H), 7.91 (s, 3H), 7.68 (d, *J* = 2.2 Hz, 1H), 7.66 (d, *J* = 2.3 Hz, 1H), 7.58 (d, *J* = 7.8 Hz, 1H), 7.26 (t, *J* = 7.8 Hz, 1H), 6.95 (d, *J* = 2.3 Hz, 1H), 6.93 (d, *J* = 2.2 Hz, 1H), 6.90 (d, *J* = 7.5 Hz, 1H), 3.75 (s, 3H), 3.43 (s, 2H), 2.18 (s, 6H). ^13^C NMR (100 MHz, DMSO-*d*_6_) *δ* (ppm): 159.07, 158.72, 158.34, 156.66, 155.80, 140.80, 132.59, 128.79, 127.85, 126.24, 122.44, 122.12, 121.49, 119.56, 118.06, 115.42, 114.31, 114.25, 112.24, 64.23, 55.69, 55.60, 45.44. HRMS (ESI)^+^ calculated for C_25_H_26_ClN_6_O_2_, [M + H] ^+^: *m/z* 477.1800, found 477.1801. HPLC purity 95.11%. Off-White solid, m.p. 228.2-232.1℃, yield 52.70%.

**The Structural Characterization of Compound 18a-p:**

***4-(5-chloro-2-((3-((dimethylamino)methyl)***

***phenyl)amino)pyri-midin-4-yl)-N-(3-((dimethylamino)methyl)phenyl)thiophene-2-carboxamide* (18a).** ^1^H NMR (400 MHz, DMSO-*d*_6_) *δ* (ppm): 10.53 (s, 1H), 9.91 (s, 1H), 8.72 (d, *J* = 1.4 Hz, 1H), 8.69 (d, *J* = 1.3 Hz, 1H), 8.65 (s, 1H), 7.93 (s, 1H), 7.71 (d, *J* = 6.6 Hz, 2H), 7.56 (d, *J* = 7.3 Hz, 1H), 7.34 – 7.29 (m, 1H), 7.26 (t, *J* = 7.8 Hz, 1H), 7.05 (d, *J* = 7.5 Hz, 1H), 6.93 (d, *J* = 7.5 Hz, 1H), 3.43 (s, 4H), 2.20 (s, 6H), 2.14 (s, 6H). ^13^C NMR (100 MHz, DMSO-*d*_6_) *δ* (ppm): 160.00, 159.53, 158.54, 156.03, 140.76, 140.42, 139.60, 139.29, 139.08, 138.12, 135.49, 130.17, 128.95, 128.84, 124.84, 122.95, 121.21, 119.97, 119.60, 118.46, 116.52, 63.68, 45.26, 45.18. HRMS (ESI)^+^ calculated for C_27_H_30_ClN_6_OS, [M + H] ^+^: *m/z* 521.1885, found 521.1885. HPLC purity 97.12%. White solid, m.p. 115.7-119.5℃, yield 57.03%.

***4-(5-chloro-2-((3-((dimethylamino)methyl)***

***phenyl) amino)pyrimidin-4-yl)-N-(4-((dimethylamino)methyl)phenyl)thiophene-2-carboxamide* (18b).** ^1^H NMR (400 MHz, DMSO-*d*_6_) *δ* (ppm): 10.50 (s, 1H), 9.89 (s, 1H), 8.70 (d, *J* = 6.9 Hz, 2H), 8.64 (s, 1H), 7.93 (s, 1H), 7.70 (d, *J* = 5.8 Hz, 2H), 7.55 (d, *J* = 8.0 Hz, 1H), 7.33 – 7.28 (m, 1H), 7.25 (t, *J* = 7.8 Hz, 1H), 7.03 (d, *J* = 7.6 Hz, 1H), 6.93 – 6.89 (m, 1H), 3.39 (s, 4H), 2.16 (s, 6H), 2.10 (s, 6H). ^13^C NMR (100 MHz, DMSO-*d*_6_) *δ* (ppm): 159.97, 159.55, 158.54, 140.76, 140.40, 140.10, 139.95, 139.04, 138.13, 135.49, 130.09, 128.93, 128.78, 124.73, 122.80, 121.04, 119.79, 119.43, 118.26, 116.79, 116.47, 114.77, 64.18, 63.89, 45.45. HRMS (ESI)^+^ calculated for C_27_H_30_ClN_6_OS, [M + H] ^+^: *m/z* 521.1885, found 521.1885. HPLC purity 98.64%. White solid, m.p. 110.3-114.3℃, yield 60.46%.

***4-(5-chloro-2-((3-((dimethylamino)methyl)***

***phenyl)amino)pyri-midin-4-yl)-N-(3-(dimethylcarbamoyl)phenyl)thiophene-2-carboxamide* (18c).** ^1^H NMR (400 MHz, DMSO-*d*_6_) *δ* (ppm): 10.66 (d, *J* = 4.1 Hz, 1H), 9.93 (s, 1H), 8.72 (s, 2H), 8.65 (s, 1H), 7.93 (s, 1H), 7.82 (d, *J* = 9.1 Hz, 2H), 7.57 (d, *J* = 8.1 Hz, 1H), 7.44 (t, *J* = 7.8 Hz, 1H), 7.27 (t, *J* = 7.8 Hz, 1H), 7.15 (d, *J* = 7.4 Hz, 1H), 6.94 (d, *J* = 7.5 Hz, 1H), 3.48 (s, 2H), 3.00 (s, 3H), 2.95 (s, 3H), 2.18 (s, 6H). ^13^C NMR (100 MHz, DMSO-*d*_6_) *δ* (ppm): 170.29, 160.19, 159.56, 158.54, 155.97, 140.43, 140.41, 139.04, 138.11, 137.36, 135.72, 130.37, 129.22, 128.90, 123.08, 122.78, 121.57, 120.10, 119.36, 118.60, 116.56, 63.66, 60.23, 44.99, 35.23, 14.55. HRMS (ESI)^+^ calculated for C_27_H_28_ClN_6_O_2_S, [M + H] ^+^: *m/z* 535.1677, found 535.1677. HPLC purity 96.90%. White solid, m.p. 111.2-113.6℃, yield 63.21%.

***4-(5-chloro-2-((3-((dimethylamino)methyl)phenyl)amino)pyri-midin-4-yl)-N-(3-(dimethylamino)phenyl)thiophene-2-carboxamide* (18d).** ^1^H NMR (400 MHz, DMSO-*d*_6_) *δ* (ppm): 10.31 (s, 1H), 9.90 (s, 1H), 8.73 – 8.65 (m, 2H), 8.64 (s, 1H), 7.92 (s, 1H), 7.57 (d, *J* = 8.0 Hz, 1H), 7.26 (t, *J* = 7.8 Hz, 1H), 7.15 (d, *J* = 7.8 Hz, 3H), 6.92 (d, *J* = 7.5 Hz, 1H), 6.51 (dt, *J* = 7.8, 1.9 Hz, 1H), 3.41 (s, 2H), 2.90 (s, 6H), 2.14 (s, 6H). ^13^C NMR (100 MHz, DMSO-*d*_6_) *δ* (ppm): 159.89, 159.52, 158.55, 156.04, 151.24, 141.05, 140.41, 139.80, 139.58, 138.05, 135.33, 129.87, 129.48, 128.82, 122.89, 119.90, 118.37, 116.51, 109.11, 108.93, 104.98, 64.02, 45.32, 29.51. HRMS (ESI)^+^ calculated for C_26_H_28_ClN_6_OS, [M + H] ^+^: *m/z* 507.1728, found 507.1725. HPLC purity 99.14%. White solid, m.p. 171.5-176.3℃, yield 51.09%.

***4-(5-chloro-2-((3-((dimethylamino)methyl)***

***phenyl)amino)py-rimidin-4-yl)-N-(4-(trifluoromethoxy)benzyl)thiophene-2-carboxamide* (18e).** ^1^H NMR (400 MHz, DMSO-*d*_6_) *δ* (ppm): 9.87 (s, 1H), 9.36 (t, *J* = 6.0 Hz, 1H), 8.67 – 8.58 (m, 2H), 8.49 (s, 1H), 7.84 (s, 1H), 7.58 (d, *J* = 7.9 Hz, 1H), 7.47 (d, *J* = 8.6 Hz, 2H), 7.35 (d, *J* = 8.2 Hz, 2H), 7.24 (t, *J* = 7.8 Hz, 1H), 6.90 (d, *J* = 7.5 Hz, 1H), 4.50 (d, *J* = 5.9 Hz, 2H), 3.38 (s, 2H), 2.15 (s, 6H). ^13^C NMR (100 MHz, DMSO-*d*_6_) *δ* (ppm): 161.37, 161.31, 159.49, 158.52, 156.02, 147.74, 140.41, 140.29, 139.31, 138.06, 137.47, 134.85, 130.18, 129.74, 129.30, 128.80, 122.86, 121.70, 121.50, 120.57 (*J* = 255.0 Hz), 119.87, 118.34, 116.45, 63.98, 45.34, 42.46. HRMS (ESI)^+^ calculated for C_26_H_24_ClF_3_N_5_O_2_S, [M + H] ^+^: *m/z* 562.1286, found 562.1281. HPLC purity 98.61%. White solid, m.p. 160.1-164.0℃, yield 52.73%.

***4-(5-chloro-2-((3-((dimethylamino)methyl)***

***phenyl)amino)pyri-midin-4-yl)-N-(3-chloro-4-fluorobenzyl)thiophene-2-carboxamide* (18f).** ^1^H NMR (400 MHz, DMSO-*d*_6_) *δ* (ppm): 9.87 (s, 1H), 9.37 (t, *J* = 6.0 Hz, 1H), 8.69 – 8.58 (m, 2H), 8.48 (d, *J* = 1.4 Hz, 1H), 7.84 (s, 1H), 7.61-7.51 (m, 2H), 7.43 – 7.34 (m, 2H), 7.25 (t, *J* = 7.8 Hz, 1H), 6.91 (d, *J* = 7.5 Hz, 1H), 4.45 (d, *J* = 5.9 Hz, 2H), 2.90 (s, 2H), 2.15 (s, 6H). ^13^C NMR (100 MHz, DMSO-*d*_6_) *δ* (ppm): 161.34, 159.48, 158.51, 156.72 (*J* = 244.0 Hz), 156.00, 140.38, 140.14, 138.06, 137.72, 134.89, 129.97, 129.40, 128.81, 128.67, 128.60, 122.89, 119.89, 119.65 (*J* = 18.0 Hz), 118.35, 117.26 (*J* = 21.0 Hz), 116.46, 63.96, 45.32, 42.09. HRMS (ESI)^+^ calculated for C_25_H_23_C_l2_FN_5_OS, [M + H] ^+^: *m/z* 530.0979, found 530.0979. HPLC purity 99.41%. White solid, m.p. 139.2-143.0℃, yield 60.34%.

***(S)-4-(5-chloro-2-((3-((dimethylamino)methyl)***

***phenyl)amino)pyri-midin-4-yl)-N-(2-hydroxy-1-phenylethyl)thiophene-2-carboxamide* (18g).** ^1^H NMR (400 MHz, DMSO-*d*_6_) *δ* (ppm): 9.89 (s, 1H), 9.04 (d, *J* = 8.1 Hz, 1H), 8.63 (s, 1H), 8.59 (d, *J* = 1.5 Hz, 2H), 7.83 (s, 1H), 7.64 (d, *J* = 8.1 Hz, 1H), 7.40 (d, *J* = 7.3 Hz, 2H), 7.34 (t, *J* = 7.5 Hz, 2H), 7.26 (td, *J* = 7.5, 4.4 Hz, 2H), 6.94 (d, *J* = 7.5 Hz, 1H), 5.06 (d, *J* = 5.7 Hz, 1H), 3.76 – 3.66 (m, 2H), 3.44 (s, 3H), 2.18 (s, 6H). ^13^C NMR (100 MHz, DMSO-*d*_6_) *δ* (ppm): 161.03, 159.40, 158.56, 156.30, 141.50, 140.78, 140.43, 139.24, 137.96, 134.64, 129.31, 128.84, 128.63, 127.51, 127.42, 122.97, 120.00, 118.47, 116.53, 64.77, 63.82, 56.53, 45.23. HRMS (ESI)^+^ calculated for C_26_H_27_ClN_5_O_2_S, [M + H] ^+^: *m/z* 508.1568, found 508.1568. HPLC purity 98.82%. White solid, m.p. 178.8-182.2℃, yield 54.37%.

***(S)-4-(5-chloro-2-((3-((dimethylamino)methyl)***

***phenyl)amino)pyri-midin-4-yl)-N-(2-hydroxy-1-phenylethyl)thiophene-2-carboxamide* (18h).** ^1^H NMR (400 MHz, DMSO-d_6_) *δ* (ppm): 9.89 (s, 1H), 9.05 (d, *J* = 8.1 Hz, 1H), 8.63 (s, 1H), 8.59 (s, 2H), 7.82 (s, 1H), 7.64 (d, *J* = 8.0 Hz, 1H), 7.41 (d, *J* = 7.5 Hz, 2H), 7.34 (t, *J* = 7.5 Hz, 2H), 7.26 (td, *J* = 7.6, 4.0 Hz, 2H), 6.94 (d, *J* = 7.5 Hz, 1H), 5.09 – 5.03 (m, 1H), 3.76 – 3.66 (m, 2H), 3.43 (s, 3H), 2.18 (s, 6H). ^13^C NMR (100 MHz, DMSO-d_6_) *δ* (ppm): 161.04, 159.39, 158.56, 156.29, 141.50, 140.76, 140.42, 137.97, 134.63, 129.32, 128.83, 128.63, 127.50, 127.42, 122.96, 119.99, 118.45, 116.53, 64.76, 63.85, 56.55, 45.25. HRMS (ESI)^+^ calculated for C_26_H_27_ClN_5_O_2_S, [M + H] ^+^: *m/z* 508.1568, found 508.1568. HPLC purity 99.45%. White solid, m.p. 178.5-181.5℃, yield 53.47%.

***4-(5-chloro-2-((3-((dimethylamino)methyl) phenyl)amino)pyrimidin-4-yl)-N-(pyrimidin-4-yl)thiophene-2-carboxamide* (18i).** ^1^H NMR (400 MHz, DMSO-*d*_6_) *δ* (ppm): 11.73 (s, 1H), 9.93 (s, 1H), 9.00 – 8.96 (m, 2H), 8.77 (d, *J* = 1.4 Hz, 1H), 8.74 (d, *J* = 5.8 Hz, 1H), 8.64 (d, *J* = 2.6 Hz, 1H), 8.18 (dd, *J* = 5.8, 1.4 Hz, 1H), 7.90 (s, 1H), 7.60 (d, *J* = 8.0 Hz, 1H), 7.28 (t, *J* = 7.8 Hz, 1H), 6.96 (d, *J* = 7.5 Hz, 1H), 3.50 (s, 2H), 2.20 (s, 6H). ^13^C NMR (100 MHz, DMSO-*d*_6_) *δ* (ppm): 161.61, 159.58, 158.89, 158.74, 158.51, 158.39, 140.44, 139.15, 138.43, 137.19, 132.26, 128.92, 120.15, 118.64, 116.55, 111.22, 44.96. HRMS (ESI)^+^ calculated for C_22_H_21_ClN_7_OS, [M + H]^+^: *m/z* 466.1211, found 466.1211. HPLC purity 99.51%. White solid, m.p. 105.2-109.1℃, yield 62.18%.

***4-(5-chloro-2-((3-((dimethylamino) methyl)phenyl)amino)pyrimidin-4-yl)-N-(2-methylpyridin-4-yl)thiophene-2-carboxamide* (18j).** ^1^H NMR (400 MHz, DMSO-*d*_6_) *δ* (ppm): 10.81 (s, 1H), 9.92 (s, 1H), 8.75 (s, 2H), 8.65 (s, 1H), 8.36 (d, *J* = 5.6 Hz, 1H), 7.92 (s, 1H), 7.66 (s, 1H), 7.60 (d, *J* = 5.7 Hz, 1H), 7.56 (d, *J* = 7.2 Hz, 1H), 7.26 (t, *J* = 7.8 Hz, 1H), 6.92 (d, *J* = 7.5 Hz, 1H), 3.40 (s, 2H), 2.46 (s, 3H), 2.12 (s, 6H). ^13^C NMR (100 MHz, DMSO-*d*_6_) *δ* (ppm): 160.79, 159.57, 159.07, 158.55, 155.84, 150.13, 146.25, 140.37, 139.85, 139.81, 138.19, 136.32, 130.95, 128.80, 122.84, 119.82, 118.31, 116.48, 113.43, 111.95, 64.11, 45.39, 24.82, 24.79. HRMS (ESI)^+^ calculated for C_24_H_24_ClN_6_OS, [M + H] ^+^: *m/z* 479.1415, found 479.1414. HPLC purity 99.02%. White solid, m.p. 88.7-91.6℃, yield 50.19%.

***4-(5-chloro-2-((3-((dimethylamino) methyl)phenyl)amino)pyrimidin-4-yl)-N-(1-methyl-1H-pyrazol-5-yl)thiophene-2-carboxamide* (18k).** ^1^H NMR (400 MHz, DMSO-*d*_6_) *δ* (ppm): 10.63 (s, 1H), 9.90 (s, 1H), 8.74 (d, *J* = 1.3 Hz, 1H), 8.67 (s, 1H), 8.64 (s, 1H), 7.84 (s, 1H), 7.61 (d, *J* = 9.5 Hz, 1H), 7.42 (d, *J* = 2.0 Hz, 1H), 7.26 (t, *J* = 7.8 Hz, 1H), 6.92 (d, *J* = 7.5 Hz, 1H), 6.23 (d, *J* = 2.0 Hz, 1H), 3.71 (s, 3H), 3.39 (s, 2H), 2.16 (s, 6H). ^13^C NMR (100 MHz, DMSO-*d*_6_) *δ* (ppm): 160.44, 159.59, 158.55, 155.85, 140.36, 139.74, 138.92, 138.21, 138.00, 136.08, 136.01, 130.94, 128.82, 122.89, 119.88, 118.33, 116.49, 101.35, 64.06, 45.43, 36.24, 36.21. HRMS (ESI)^+^ calculated for C_22_H_23_ClN_7_OS, [M + H] ^+^: *m/z* 468.1368, found 468.1371. HPLC purity 95.06%. White solid, m.p. 88.6-91.7℃, yield 58.21%.

***4-(5-chloro-2-((3-((dimethylamino) methyl)phenyl)***

***amino)pyrimidin-4-yl)-N-(1-methyl-1H-pyrazol-4-yl)thiophene-2-carboxamide* (18l).** ^1^H NMR (400 MHz, DMSO-*d*_6_) *δ* (ppm): 10.72 (s, 1H), 9.89 (s, 1H), 8.66 (d, *J* = 1.4 Hz, 1H), 8.64 (s, 1H), 8.57 (s, 1H), 7.99 (s, 1H), 7.90 (s, 1H), 7.56 (d, *J* = 5.6 Hz, 2H), 7.25 (t, *J* = 7.8 Hz, 1H), 6.91 (d, *J* = 7.7 Hz, 1H), 3.83 (s, 3H), 3.37 (s, 2H), 2.12 (s, 6H). ^13^C NMR (100 MHz, DMSO-*d*_6_) *δ* (ppm): 159.53, 158.54, 158.30, 155.95, 140.40, 140.27, 140.05, 138.12, 135.04, 130.62, 129.38, 128.77, 122.77, 122.19, 121.66, 119.74, 118.21, 116.44, 64.20, 45.52, 39.22, 39.19. HRMS (ESI)^+^ calculated for C_22_H_22_ClN_7_OS, [M + H] ^+^: *m/z* 468.1368, found 468.1368. HPLC purity 95.52%. White solid, m.p. 98.7-102.3℃, yield 53.11%.

***4-(5-chloro-2-((3-((dimethylamino) methyl)***

***phenyl)amino)pyrimidin-4-yl)-N-(tetrahydro-2H-pyran-4-yl)thiophene-2-carboxamide* (18m).** ^1^H NMR (400 MHz, DMSO-*d*_6_) *δ* (ppm): 9.88 (s, 1H), 8.66 – 8.58 (m, 3H), 8.47 (d, *J* = 1.5 Hz, 1H), 7.88 (s, 1H), 7.57 (d, *J* = 8.0 Hz, 1H), 7.25 (t, *J* = 7.8 Hz, 1H), 6.93 (d, *J* = 7.5 Hz, 1H), 4.04-3.94 (m, *J* = 7.8, 4.1 Hz, 1H), 3.91 (d, *J* = 2.6 Hz, 1H), 3.88 (d, *J* = 2.8 Hz, 1H), 3.42 (s, 4H), 2.18 (s, 6H), 1.78 (dd, *J* = 12.3, 2.4 Hz, 2H), 1.60 (tt, *J* = 12.0, 6.2 Hz, 2H). ^13^C NMR (100 MHz, DMSO-*d*_6_) *δ* (ppm): 160.46, 159.42, 158.53, 156.15, 140.85, 140.41, 139.56, 137.93, 134.54, 129.04, 128.78, 122.88, 119.88, 118.34, 116.47, 66.62, 63.98, 46.40, 45.36, 32.90. HRMS (ESI)^+^ calculated for C_23_H_27_ClN_5_O_2_S, [M + H]^+^: *m/z* 472.1568, found 472.1567. HPLC purity 96.97%. White solid, m.p. 190.6-194.3℃, yield 64.23%.

***4-(5-chloro-2-((3-((dimethylamino) methyl)phenyl)amino)pyrimidin-4-yl)-N-(4-methylpiperazin-1-yl)thiophene-2-carboxamide* (18n).** ^1^H NMR (400 MHz, DMSO-*d*_6_) *δ* (ppm): 9.86 (d, *J* = 6.6 Hz, 1H), 9.19 (s, 1H), 8.70 (d, *J* = 1.7 Hz, 1H), 8.64 – 8.54 (m, 2H), 7.86 (d, *J* = 10.6 Hz, 1H), 7.56 (d, *J* = 8.1 Hz, 1H), 7.26-1.21 (m, 1H), 6.90 (d, *J* = 7.6 Hz, 1H), 2.95 – 2.89 (m, 2H), 2.78 (d, *J* = 8.5 Hz, 2H), 2.43 (s, 2H), 2.35 – 2.26 (m, 2H), 2.24 – 2.14 (m, 9H). ^13^C NMR (100 MHz, DMSO-*d*_6_) *δ* (ppm): HRMS (ESI)^+^ calculated for C_23_H_29_ClN_7_OS, [M + H] ^+^: *m/z* 486.1837, found 486.1835. HPLC purity 96.41%. White solid, m.p. 193.9-197.7℃, yield 63.57%.

***4-(5-chloro-2-((3-((dimethylamino)methyl)phenyl) amino)pyrimidin-4-yl)-N-morpholinothiophene-2-carboxamide* (18o/SKLB-D18).** ^1^H NMR (400 MHz, DMSO-*d*_6_) *δ* (ppm): 9.84 (s, 1H), 9.28 (s, 1H), 8.69 (s, 1H), 8.62 (s, 1H), 8.59 (s, 1H), 7.84 (d, *J* = 8.0 Hz, 1H), 7.57 (d, *J* = 8.0 Hz, 1H), 7.25 (t, *J* = 7.8 Hz, 1H), 6.92 (d, *J* = 7.5 Hz, 1H), 3.86 (d, *J* = 11.4 Hz, 2H), 3.77 – 3.62 (m, 4H), 2.96 – 2.89 (m, 2H), 2.78 (d, *J* = 9.9 Hz, 2H), 2.19 (s, 3H), 2.16 (s, 3H). ^13^C NMR (100 MHz, DMSO-*d*_6_) *δ* (ppm): 161.73, 159.49, 158.51, 158.51, 140.44, 138.08, 136.25, 135.30, 134.70, 133.21, 128.78, 122.90, 119.93, 118.32, 116.46, 66.46, 66.20, 63.96, 56.22, 55.02, 45.32, 45.32. HRMS (ESI)^+^ calculated for C_22_H_26_ClN_6_O_2_S, [M + H] ^+^: *m/z* 473.1521, found 473.1520. HPLC purity 97.68%. White solid, m.p. 208.2-211.7℃, yield 55.78%.

***4-(5-chloro-2-((3-((dimethylamino)methyl)***

***phenyl)amino)pyrimidin-4-yl)-N-(2-morpholinoethyl)thiophene-2-carboxamide* (18p).** ^1^H NMR (400 MHz, DMSO-*d*_6_) *δ* (ppm): 9.87 (s, 1H), 8.72 (t, *J* = 5.7 Hz, 1H), 8.66 – 8.53 (m, 2H), 8.41 (d, *J* = 1.4 Hz, 1H), 7.85 (s, 1H), 7.59 (d, *J* = 8.2 Hz, 1H), 7.25 (t, *J* = 7.8 Hz, 1H), 6.92 (d, *J* = 7.5 Hz, 1H), 3.57 (dd, *J* = 5.6, 3.6 Hz, 4H), 3.41 (s, 4H), 2.47 (d, *J* = 7.0 Hz, 2H), 2.42 (t, *J* = 4.7 Hz, 4H), 2.18 (s, 6H). ^13^C NMR (100 MHz, DMSO-*d*_6_) *δ* (ppm): 161.17, 159.46, 158.52, 156.08, 140.73, 140.41, 139.60, 137.96, 134.48, 128.91, 128.80, 122.85, 119.87, 118.32, 116.44, 66.65, 64.01, 57.83, 53.78, 45.39, 37.01. HRMS (ESI)^+^ calculated for C_24_H_30_ClN_6_O_2_S, [M + H] ^+^: *m/z* 501.1834, found 501.1834. HPLC purity 99.01%. White solid, m.p. 121.2-126.0, yield 63.45%.

**The ^1^H NMR, ^13^C NMR, HRMS and HPLC spectrum of compounds.**

The ^1^H-NMR spectrum of compound **6a**


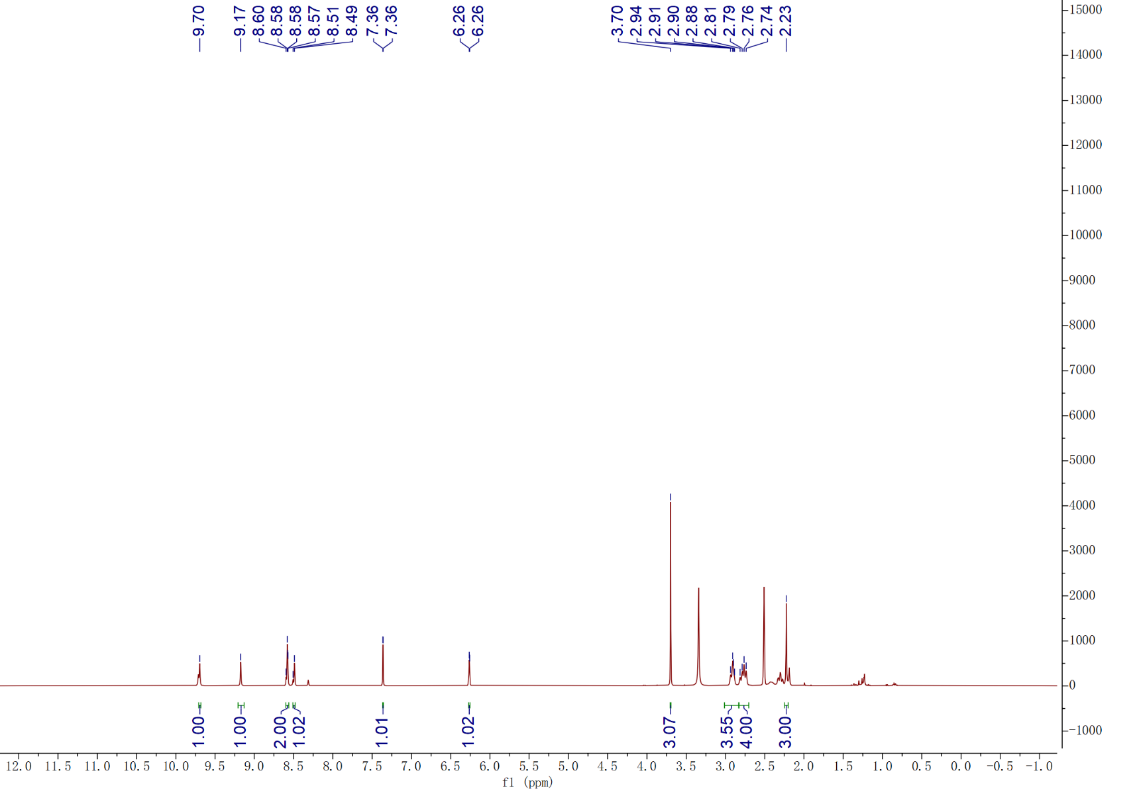


The ^13^C-NMR spectrum of compound **6a**


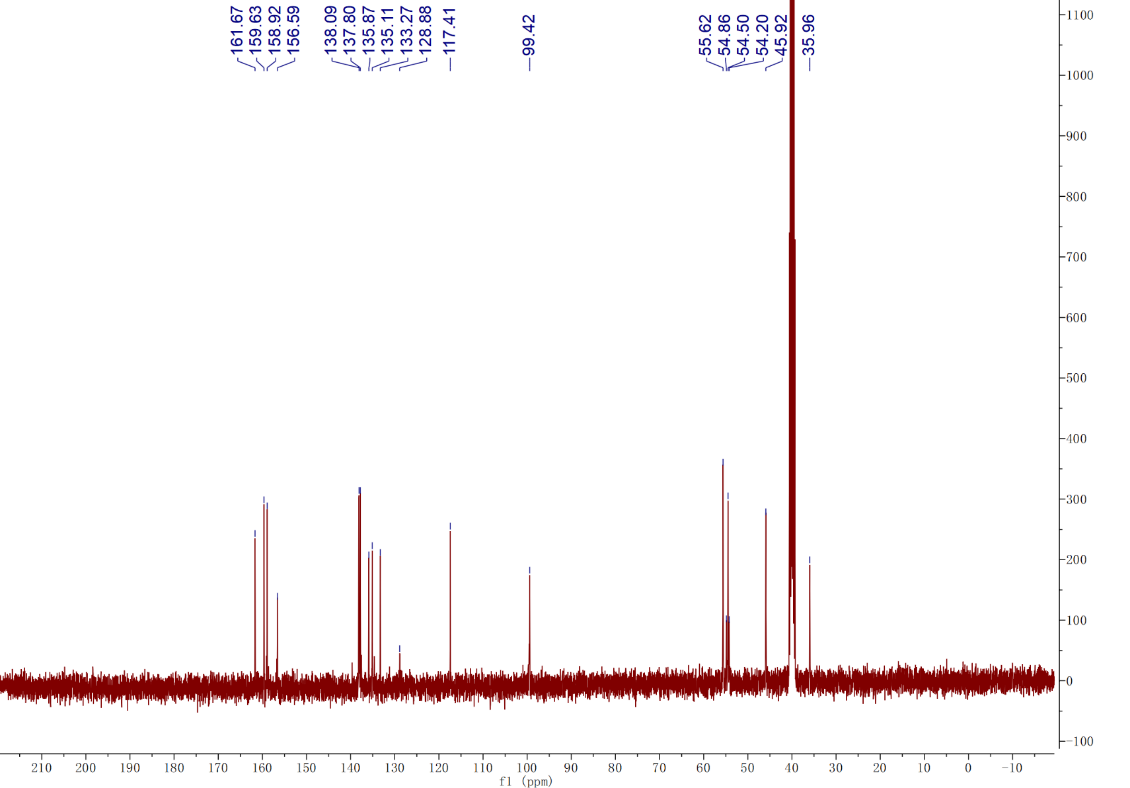


The MS data of compound **6a**


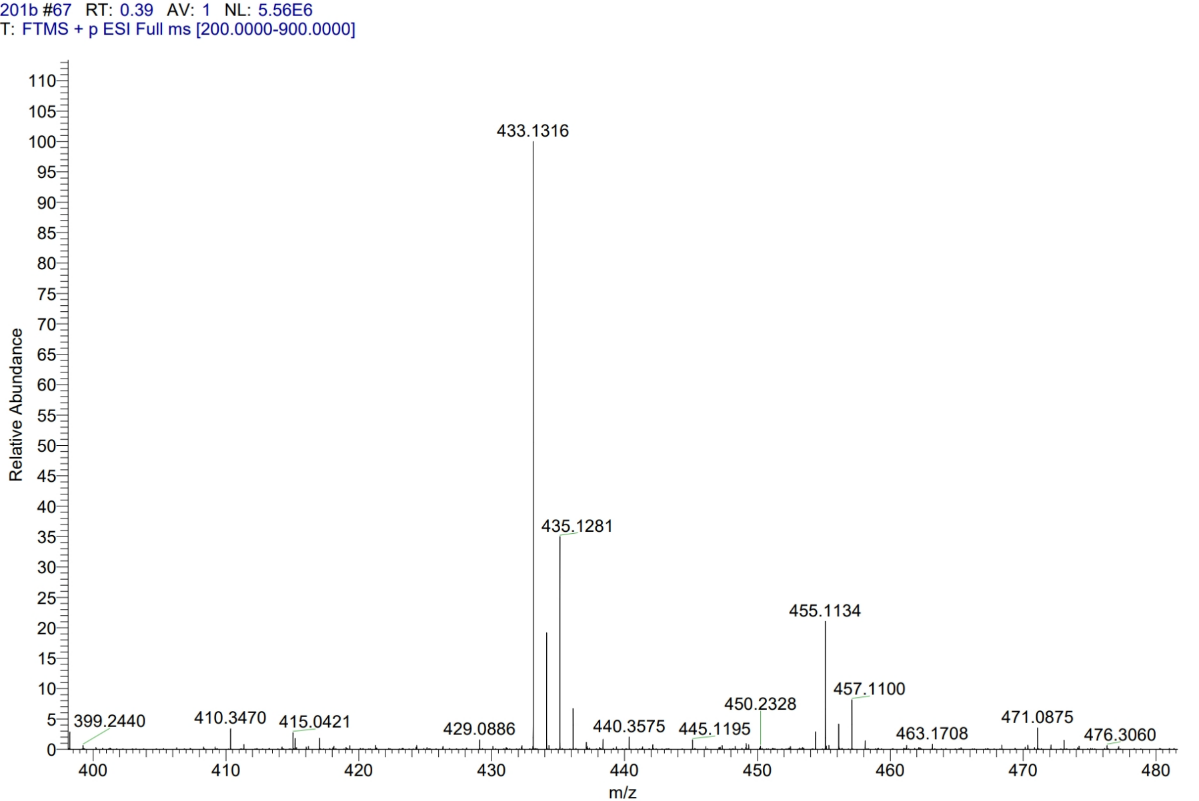


The HPLC spectrum of compound **6a**
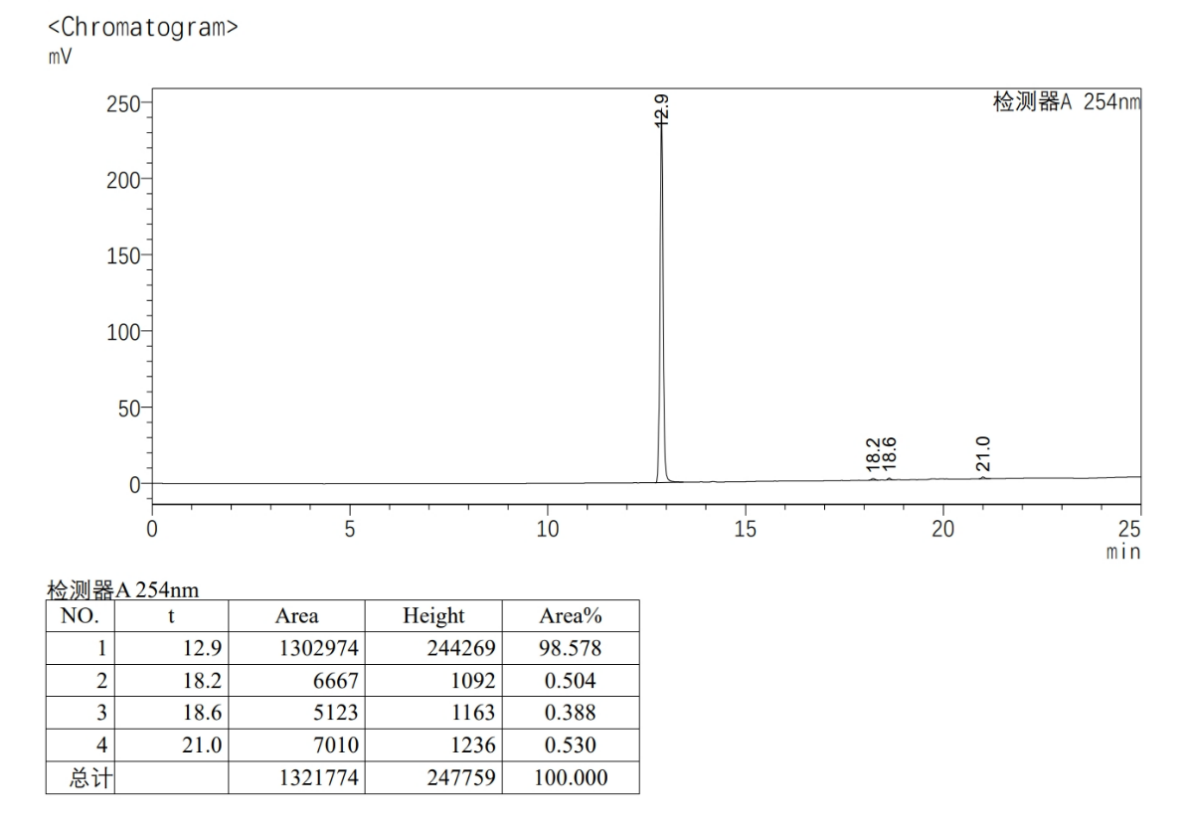


The ^1^H-NMR spectrum of compound **6b**


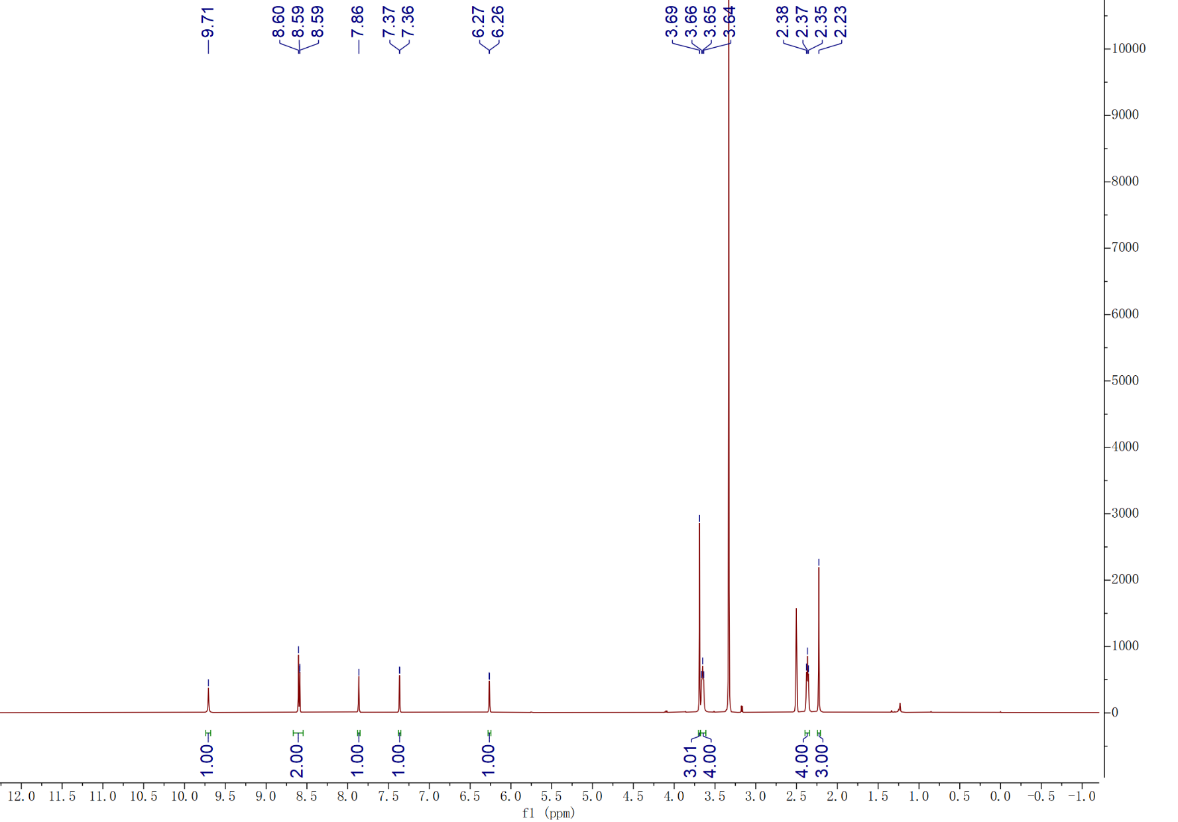


The ^13^C-NMR spectrum of compound **6b**
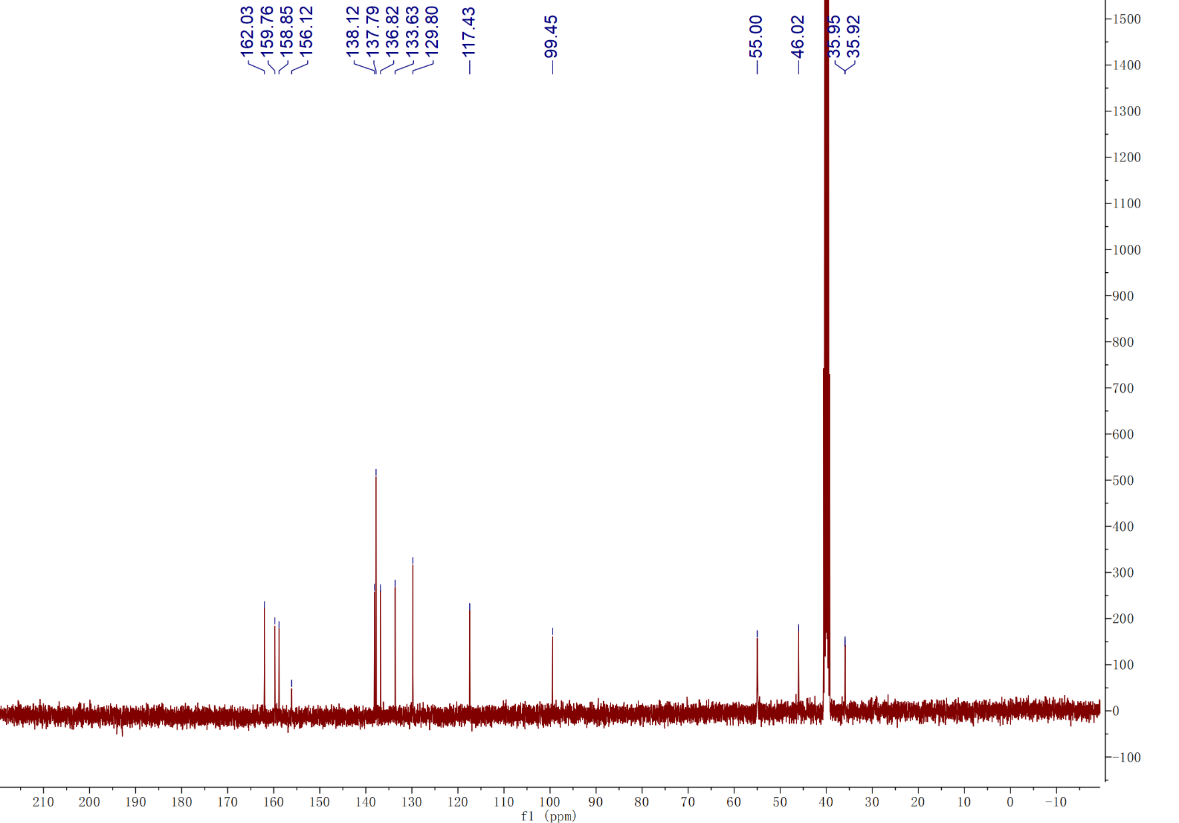


The MS data of compound **6b**


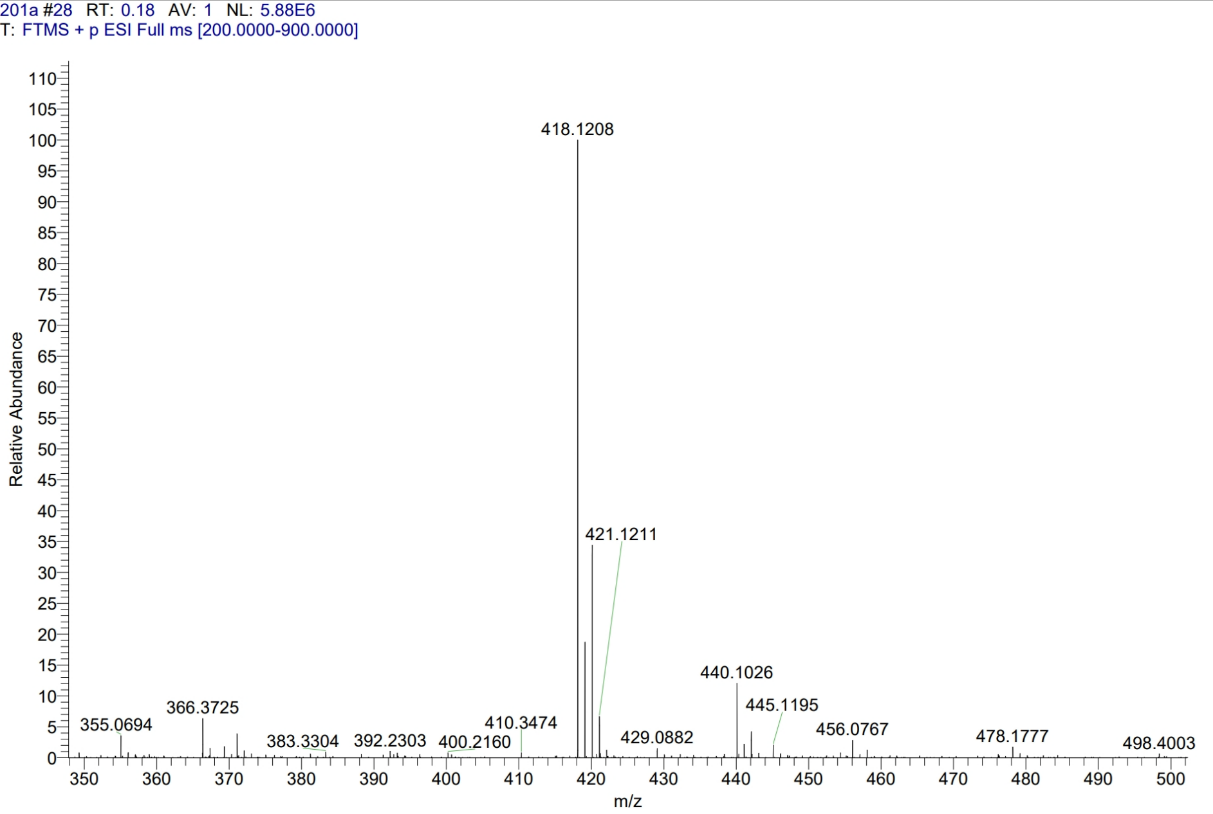


The HPLC spectrum of compound **6b**


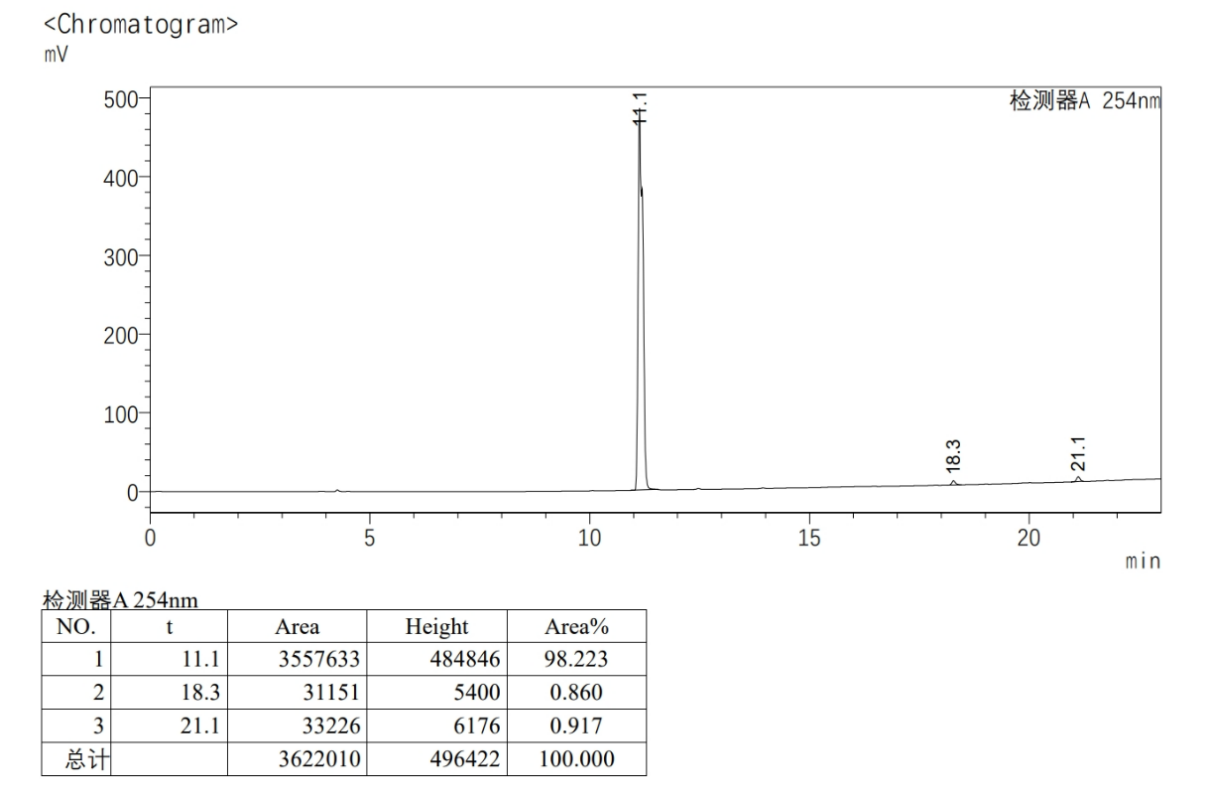


The ^1^H-NMR spectrum of compound **6c**


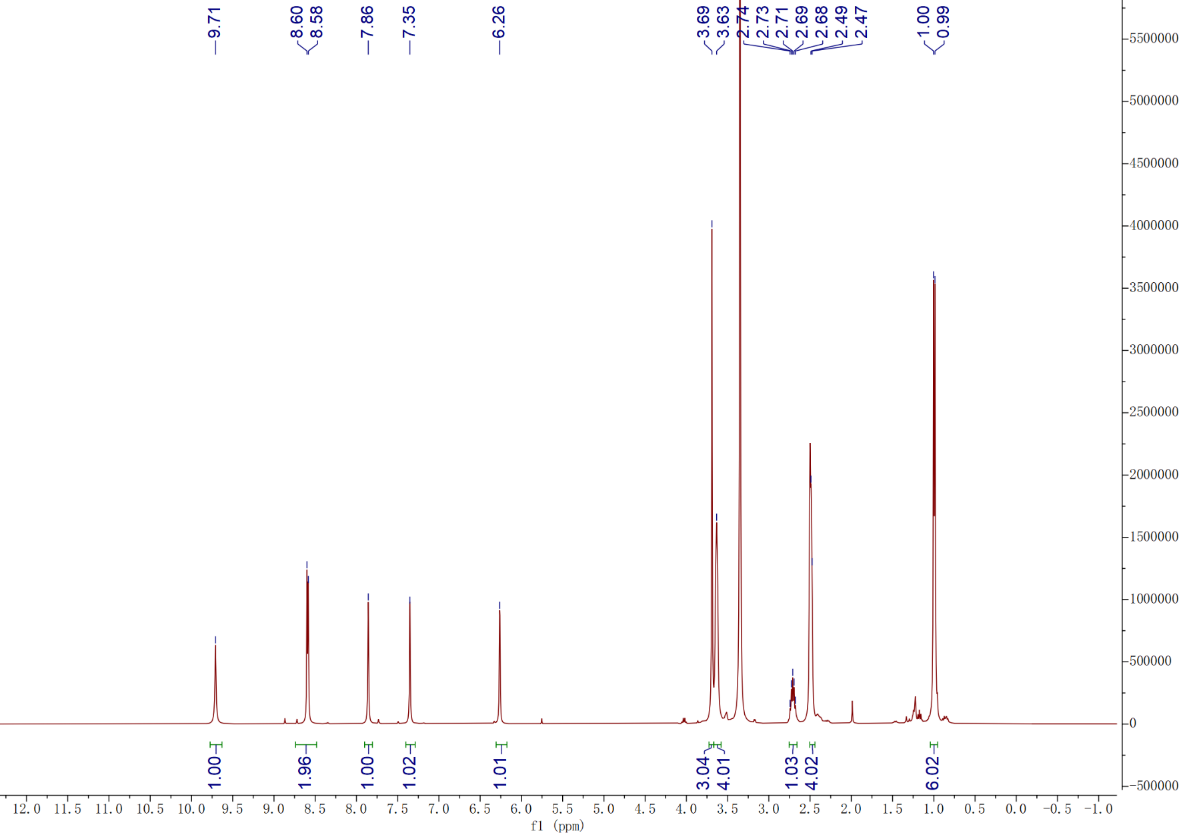


The ^13^C-NMR spectrum of compound **6c**


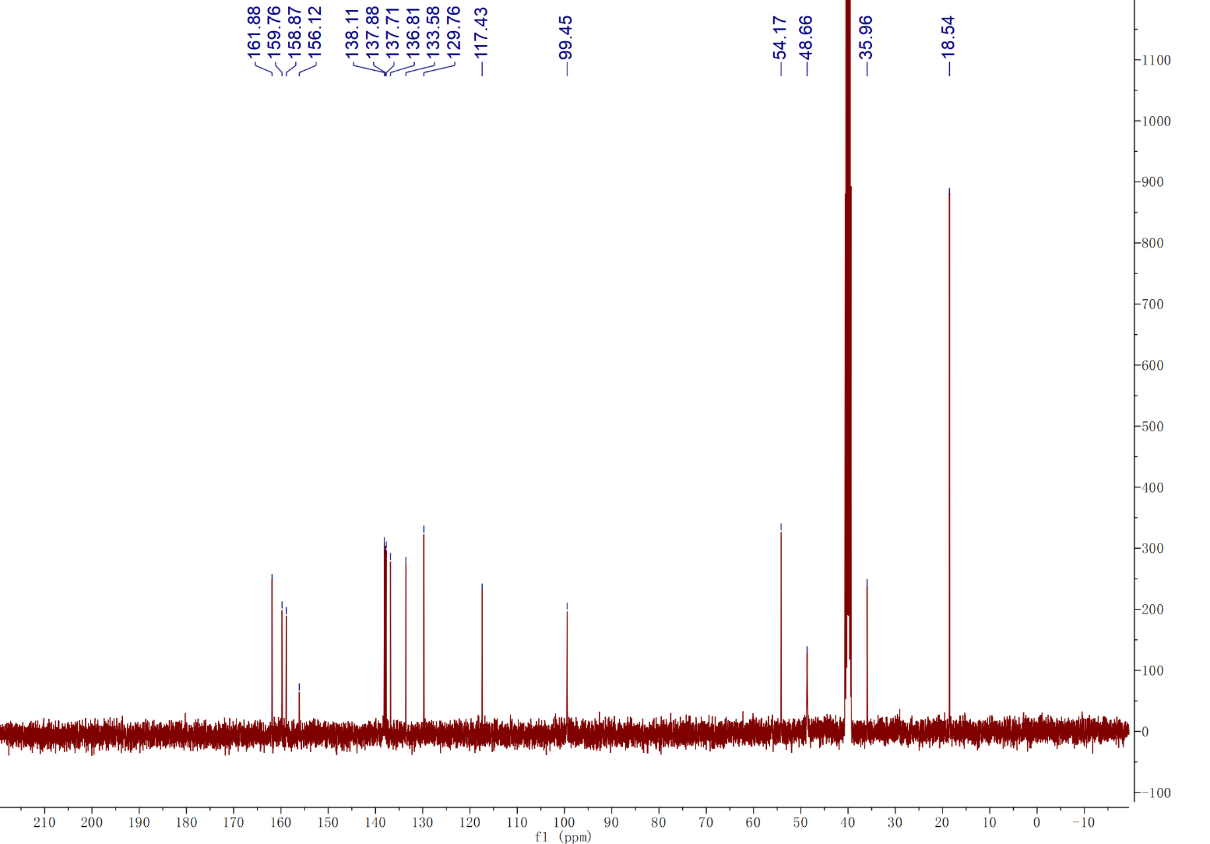


The MS data of compound **6c**


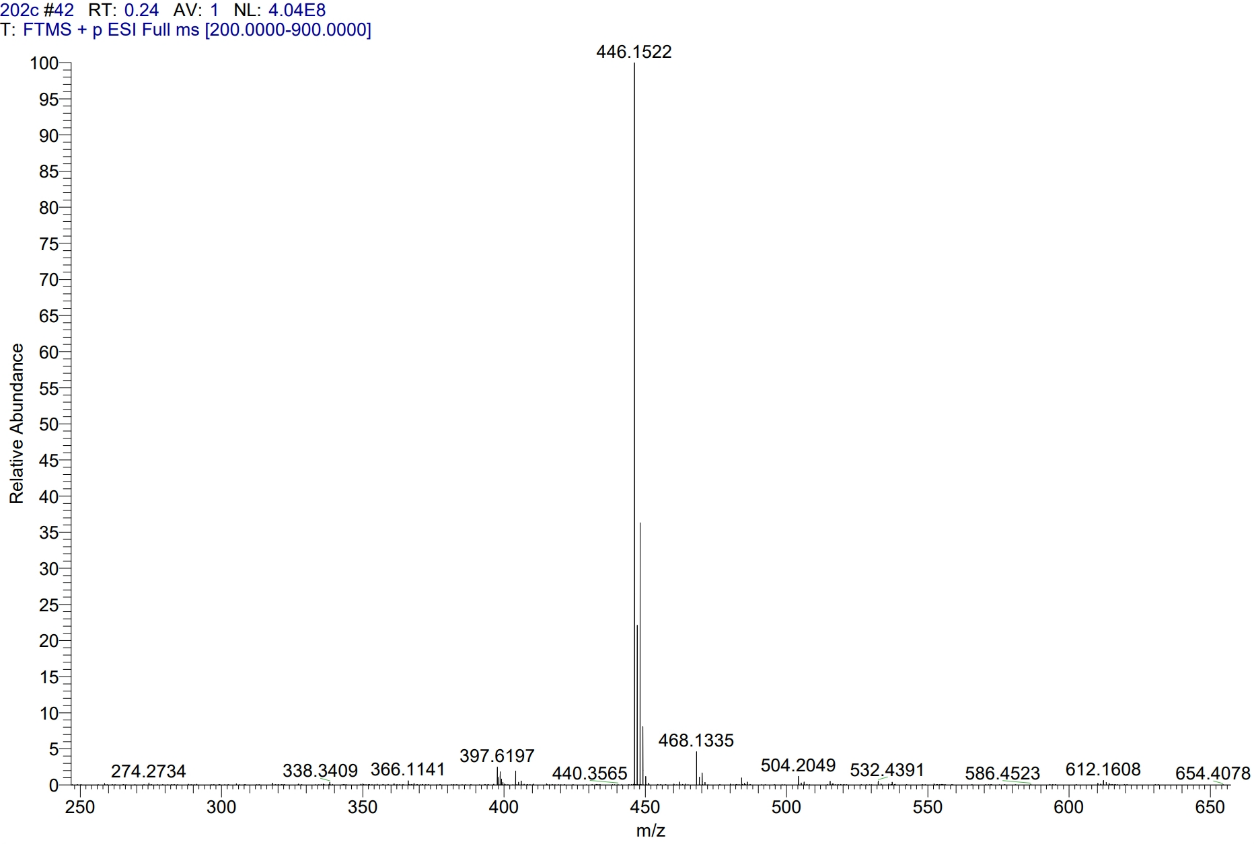


The HPLC spectrum of compound **6c**


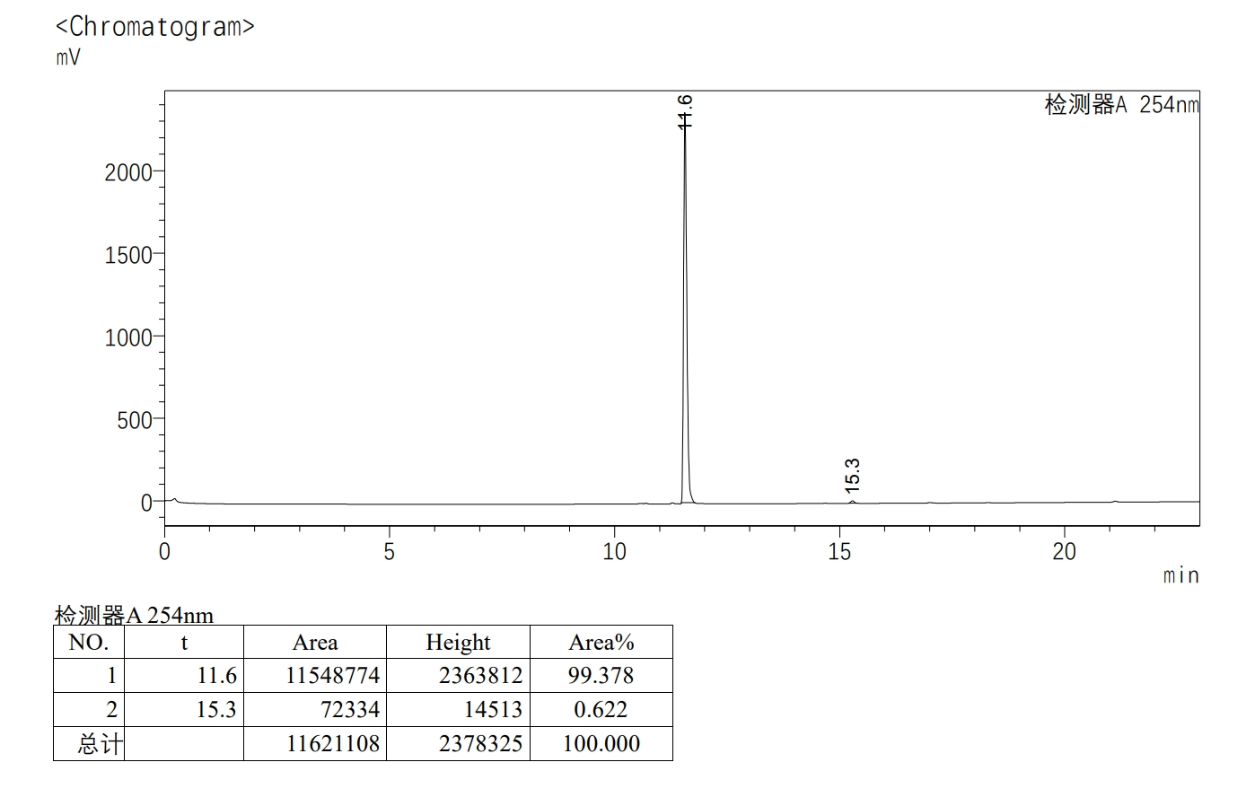


The ^1^H-NMR spectrum of compound **6d**


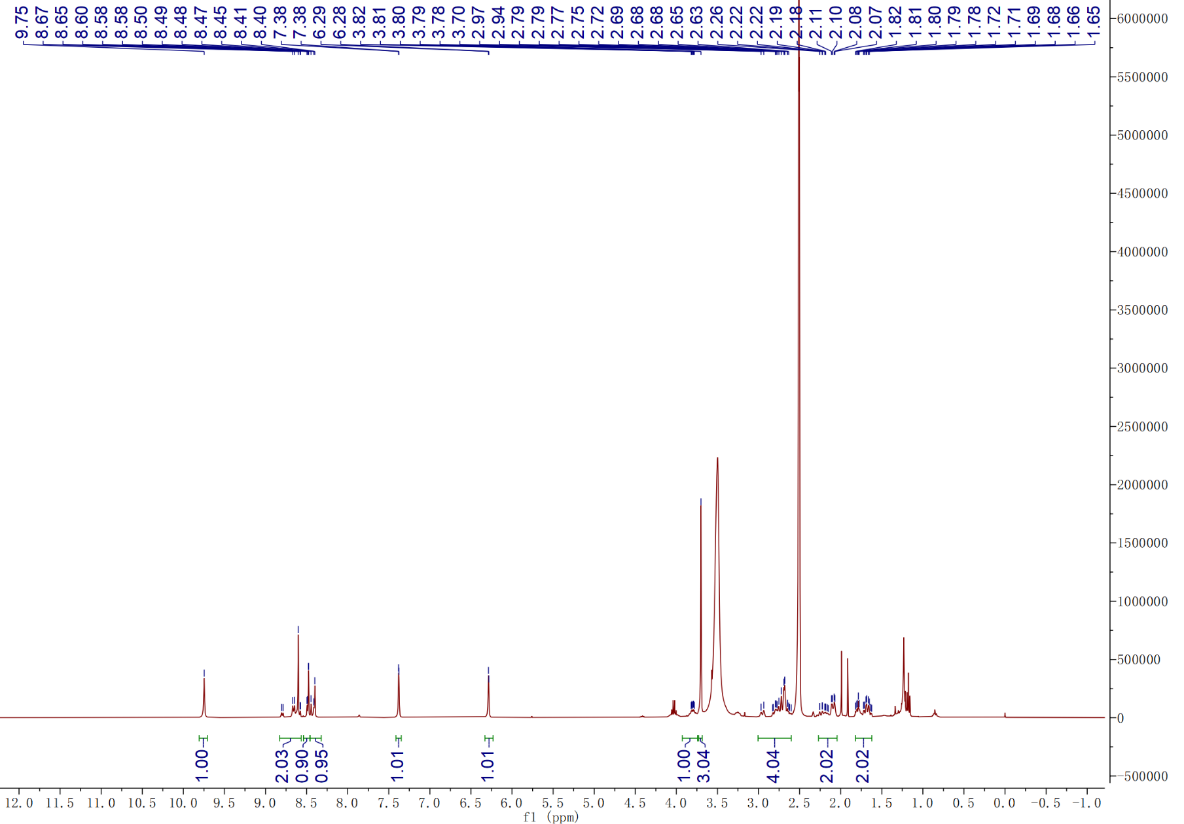


The ^13^C-NMR spectrum of compound **6d**


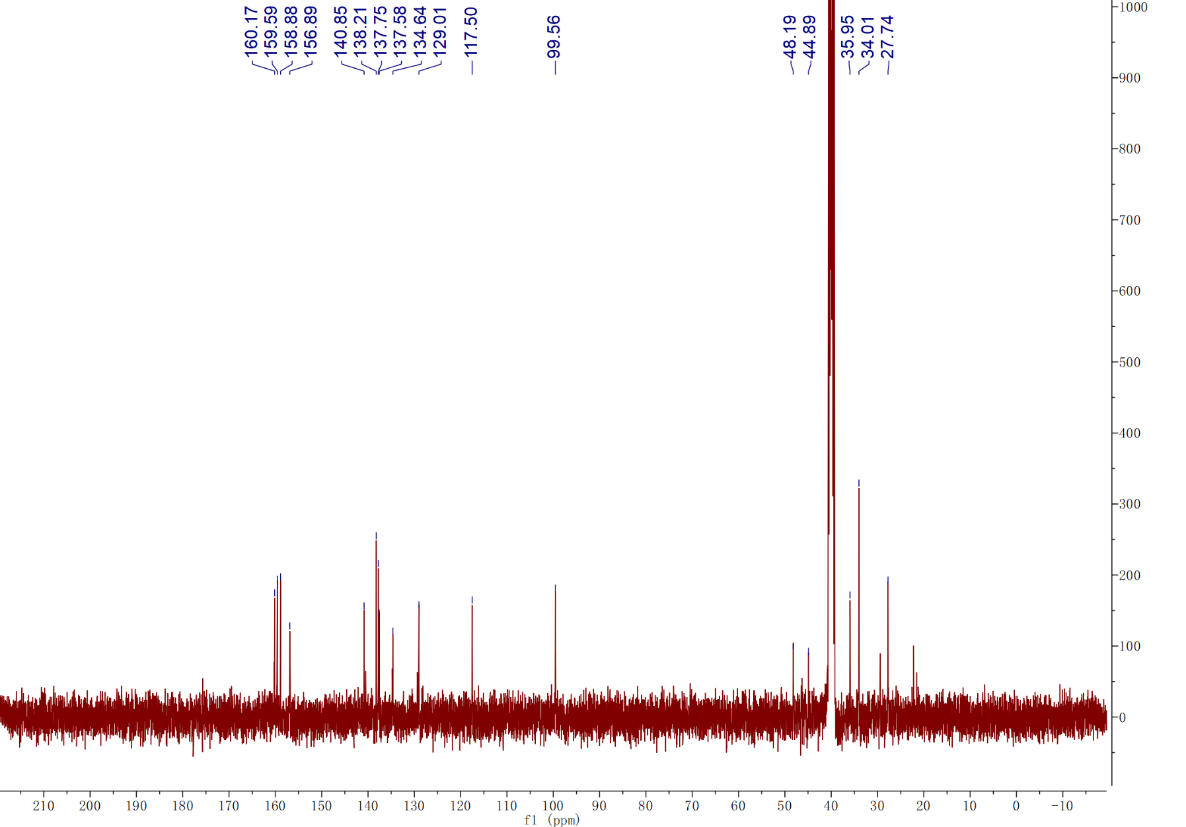


The MS data of compound **6d**
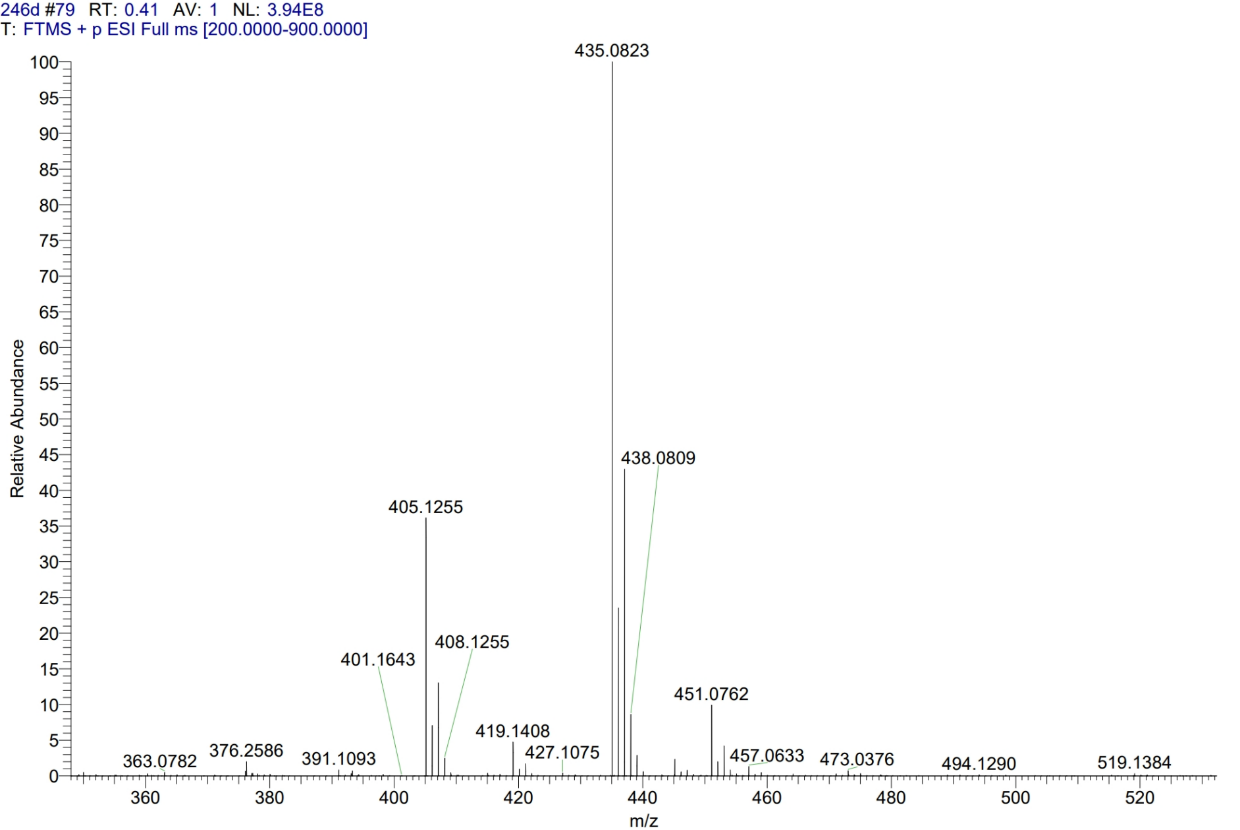


The HPLC spectrum of compound **6d**


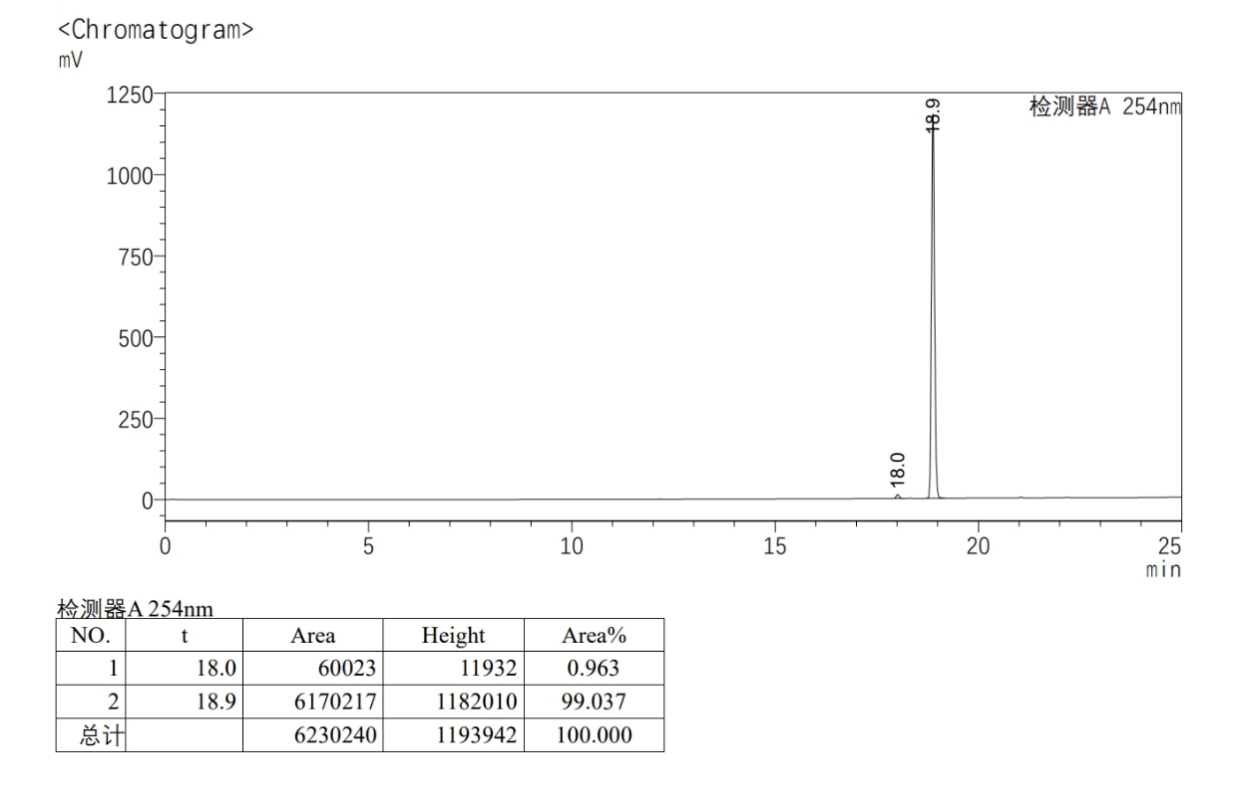


The ^1^H-NMR spectrum of compound **6e**


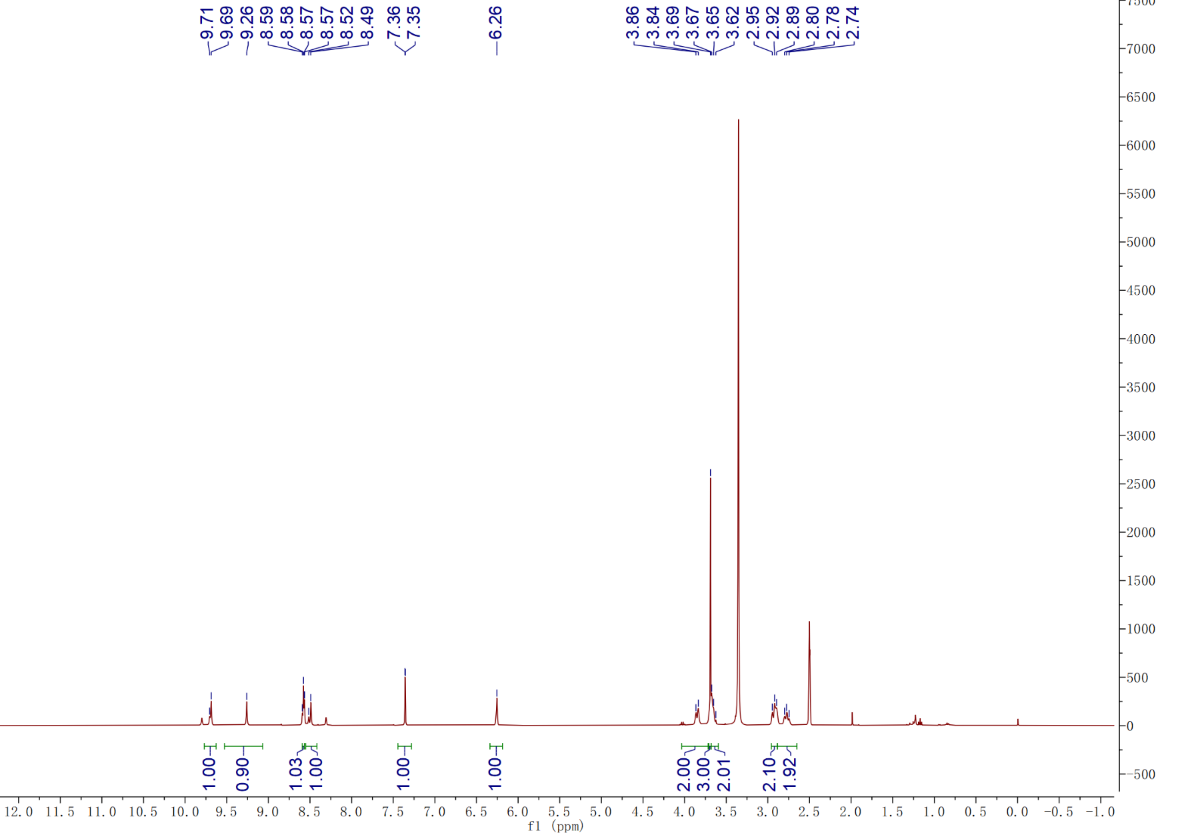


The ^13^C-NMR spectrum of compound **6e**


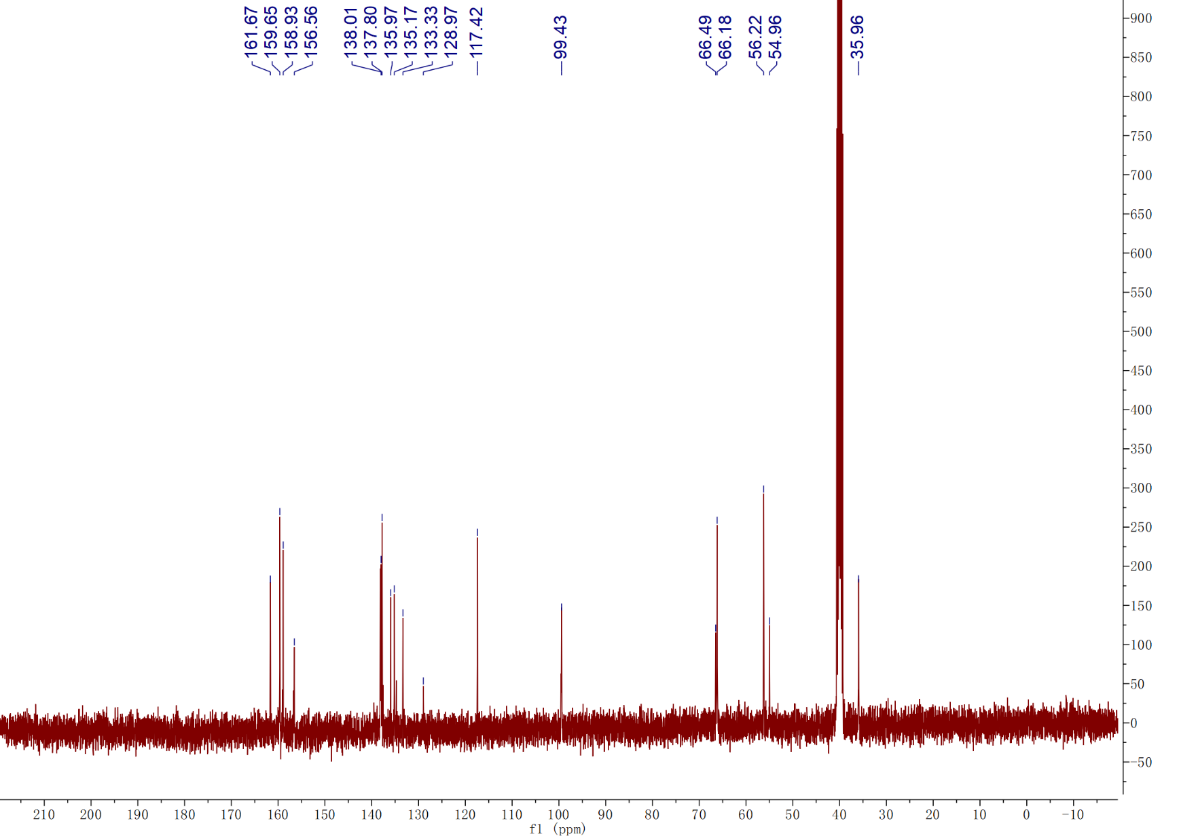


The MS data of compound **6e**


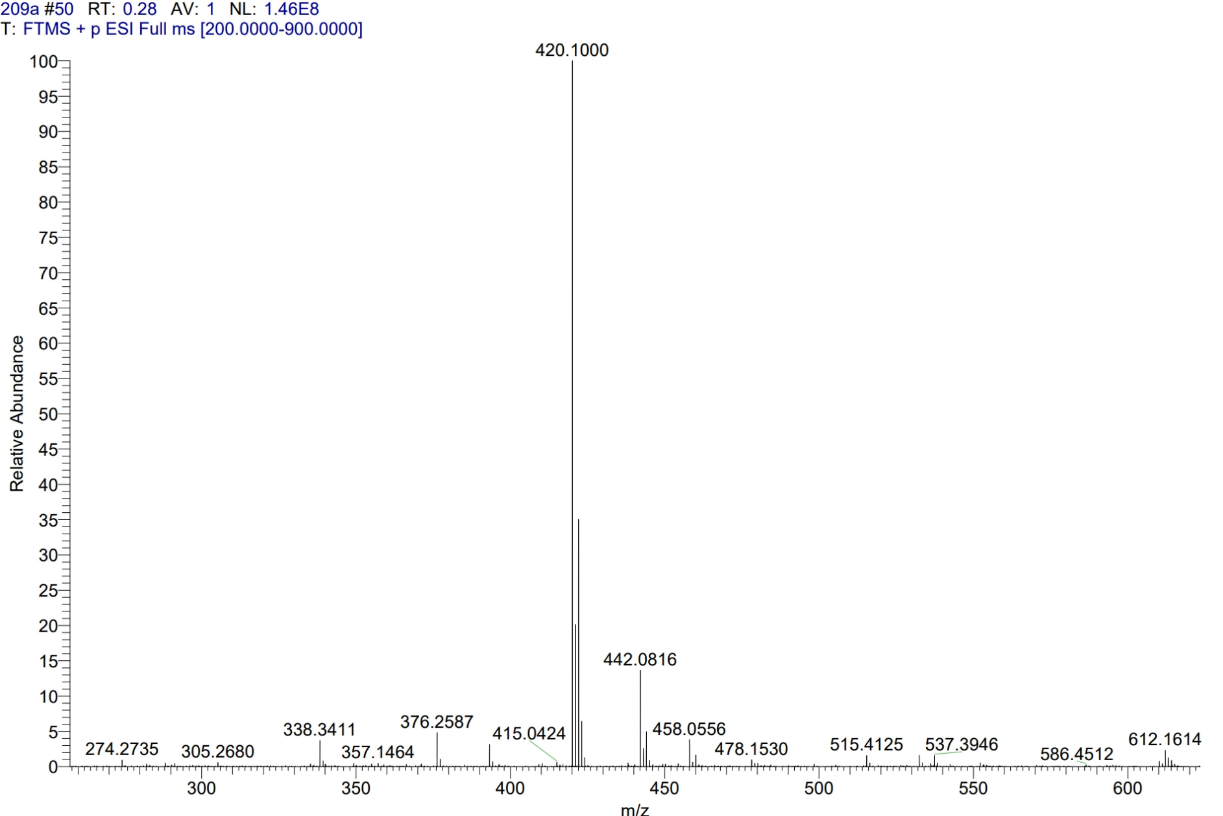


The HPLC spectrum of compound **6e**


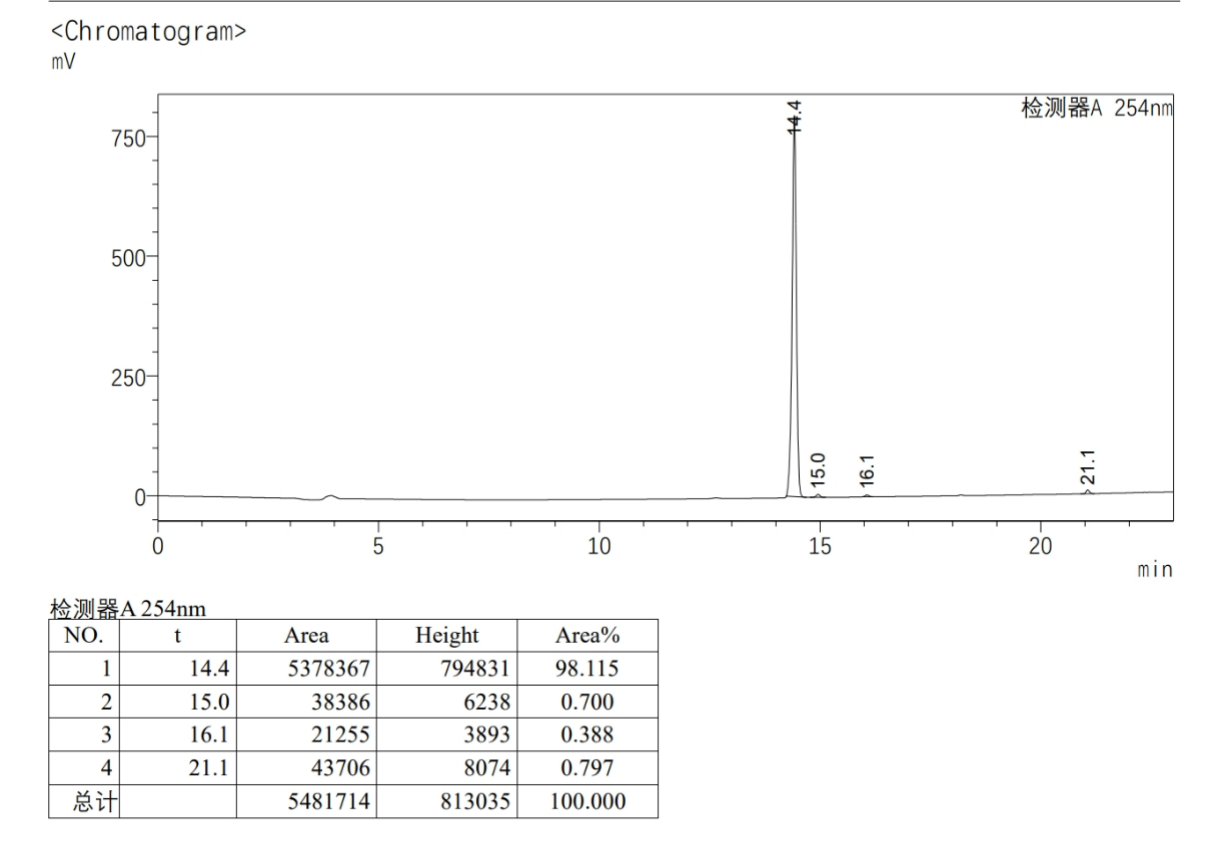


The ^1^H-NMR spectrum of compound **6f**


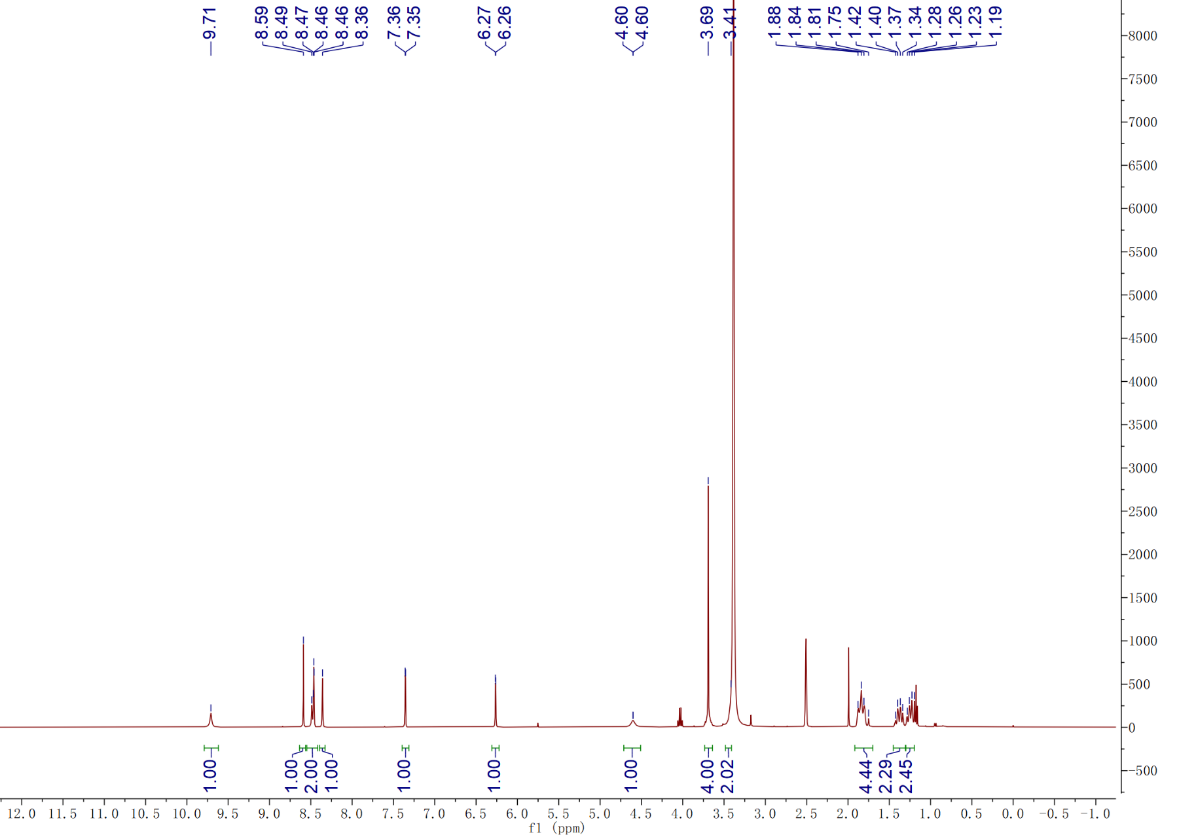


The ^13^C-NMR spectrum of compound **6f**


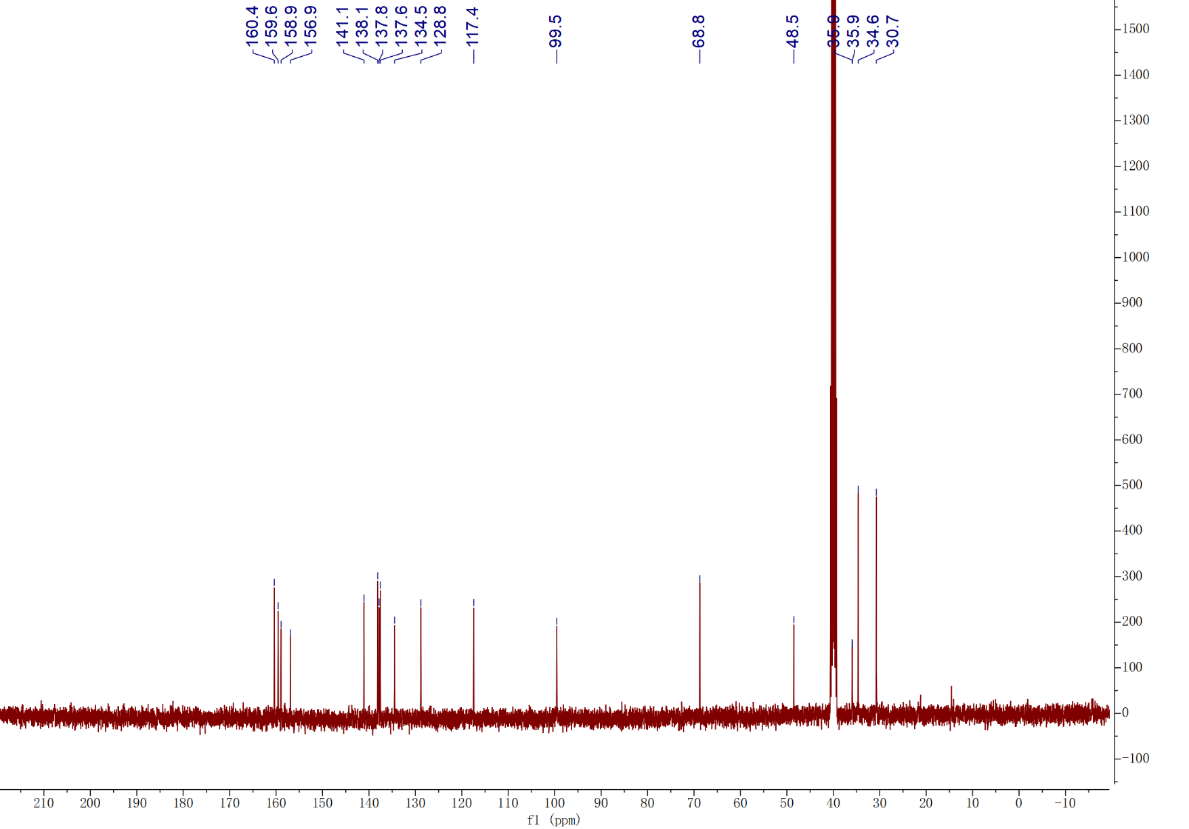


The MS data of compound **6f**


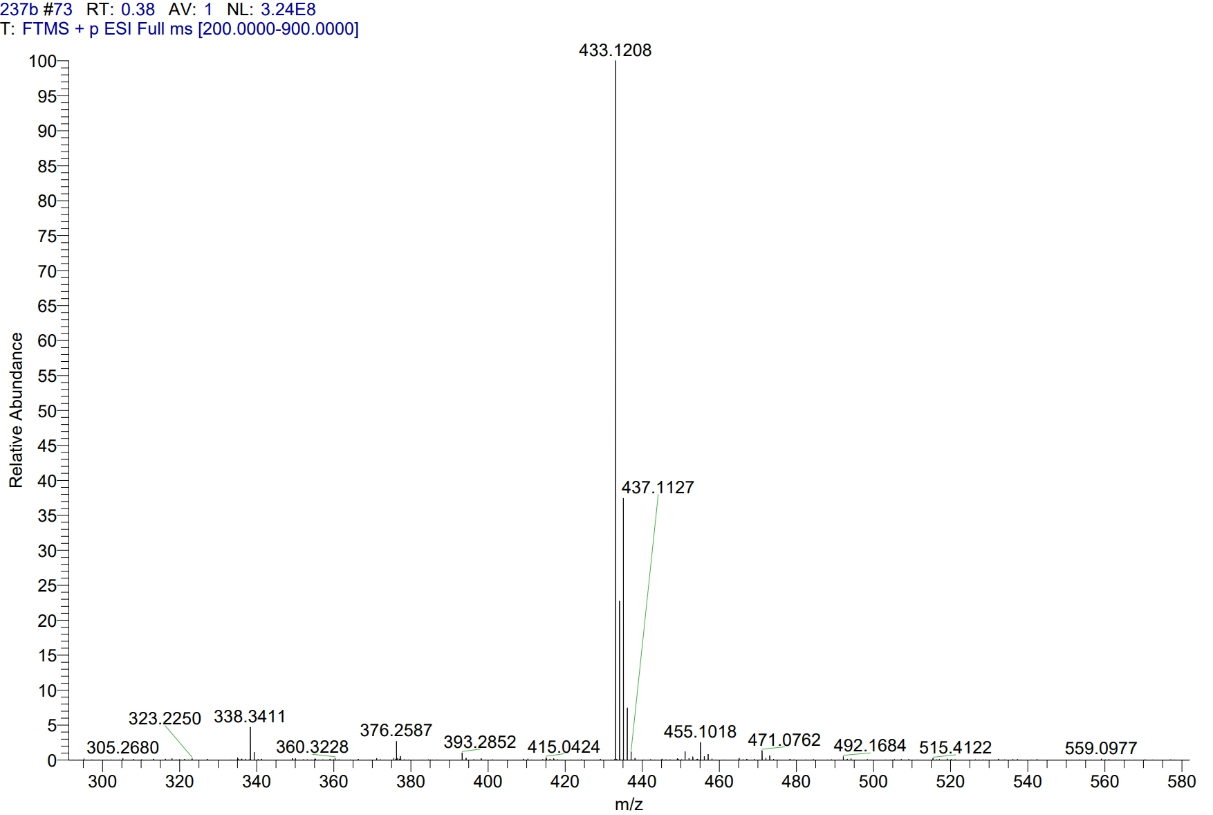


The HPLC spectrum of compound **6f**


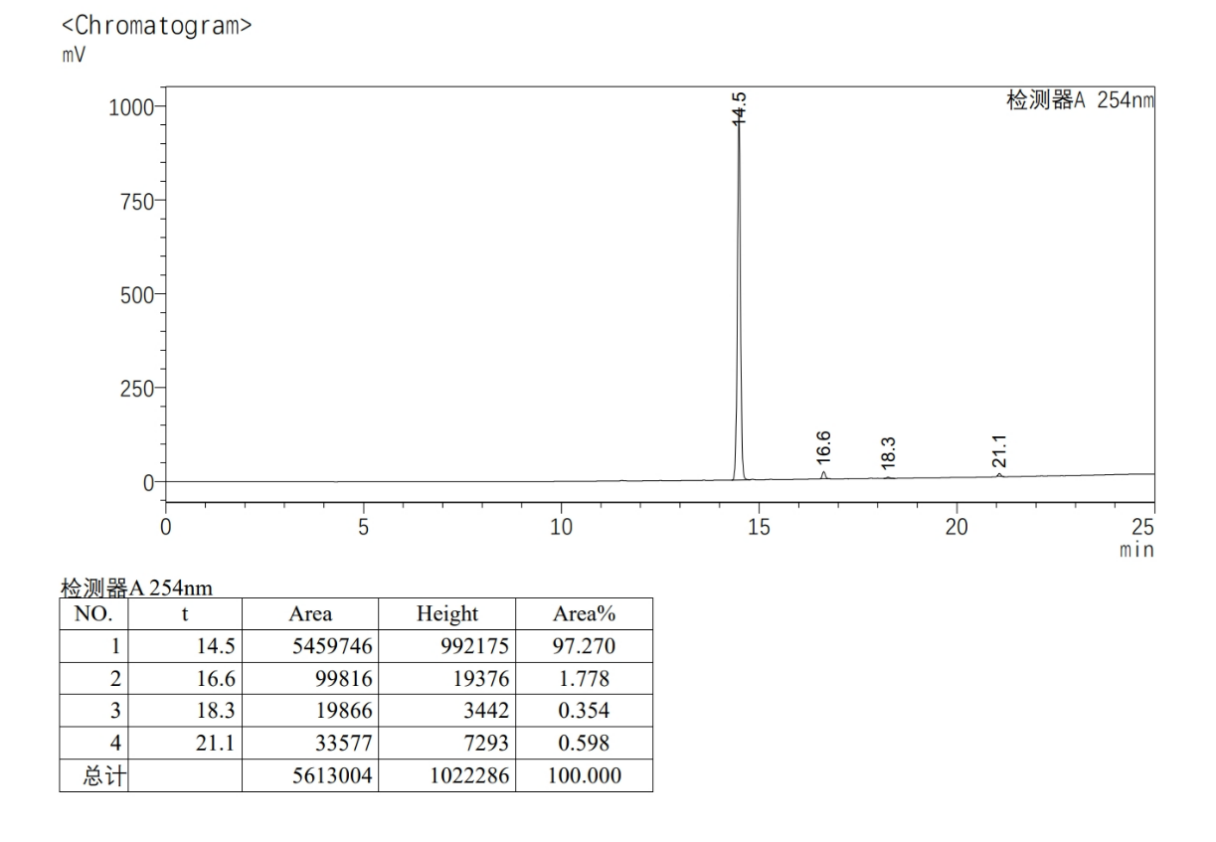


The ^1^H-NMR spectrum of compound **6g**


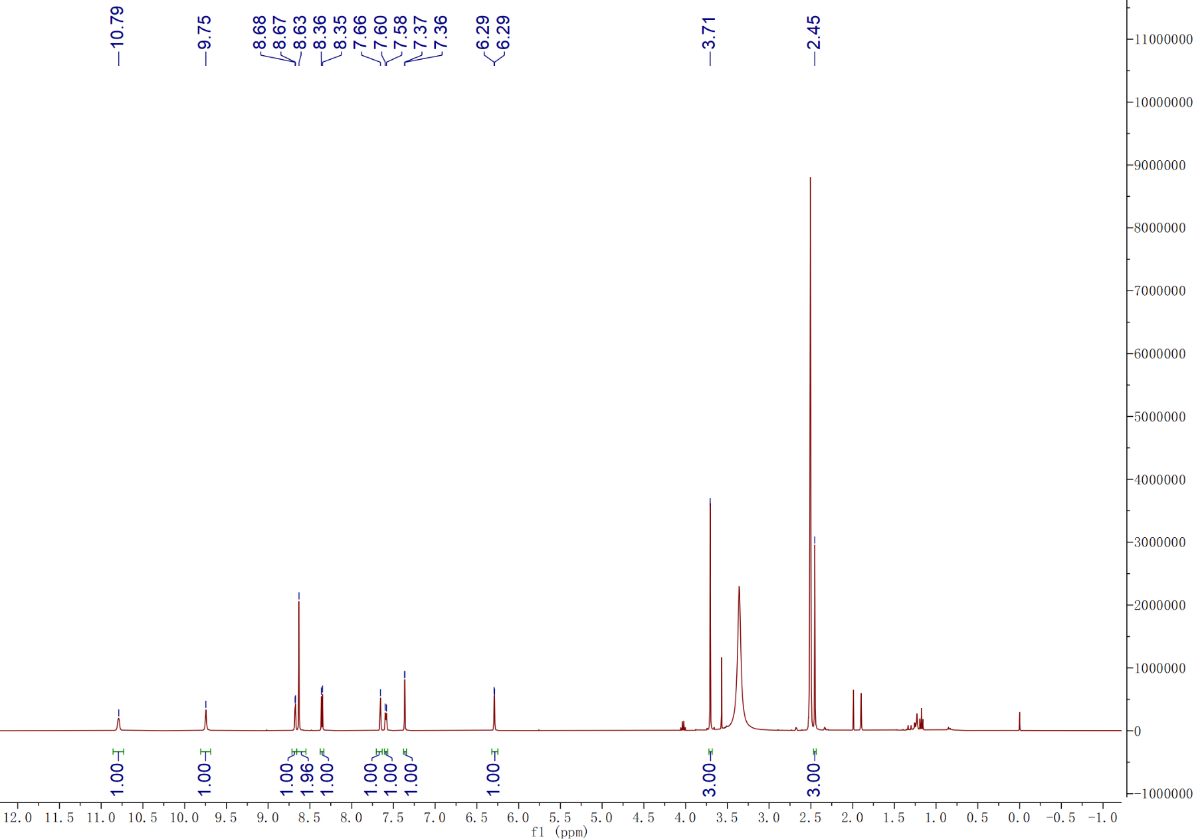


The ^13^C-NMR spectrum of compound **6g**


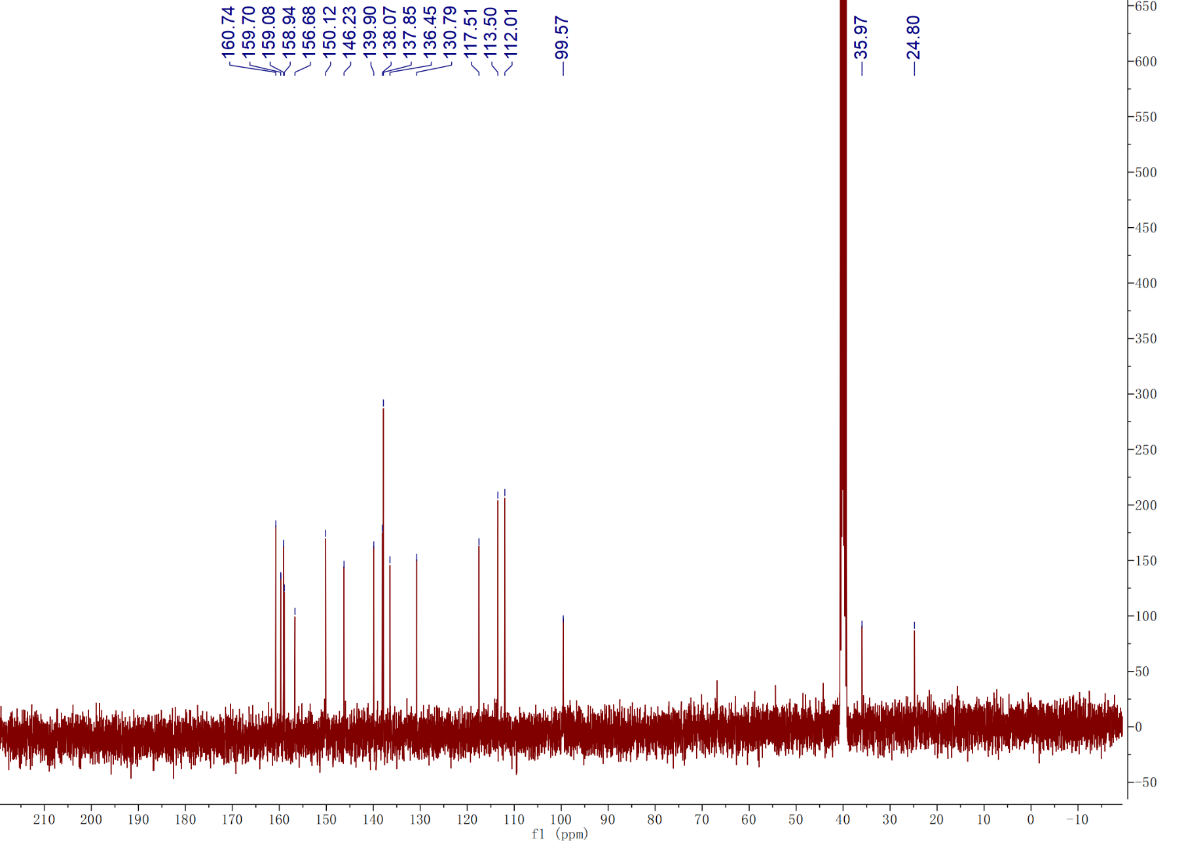


The MS data of compound **6g**


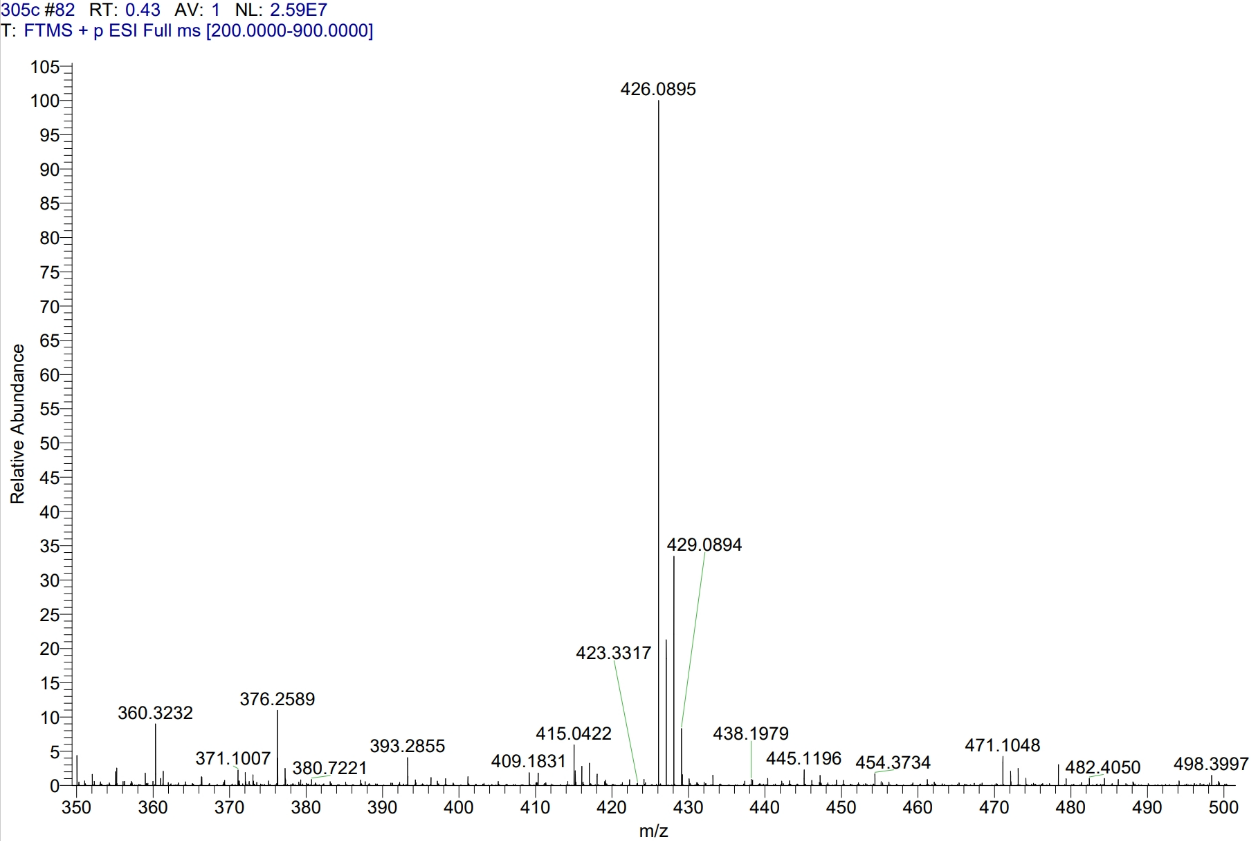


The HPLC spectrum of compound **6g**


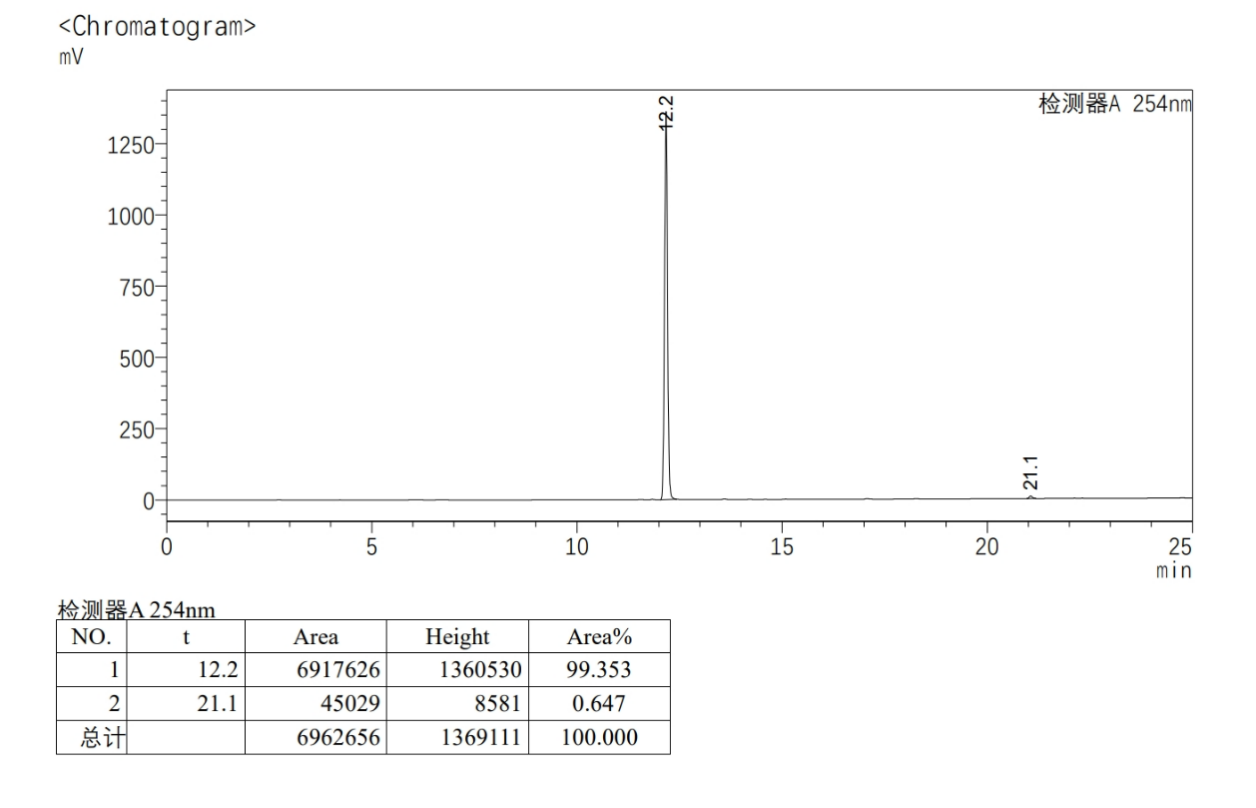


The ^1^H-NMR spectrum of compound **6h**


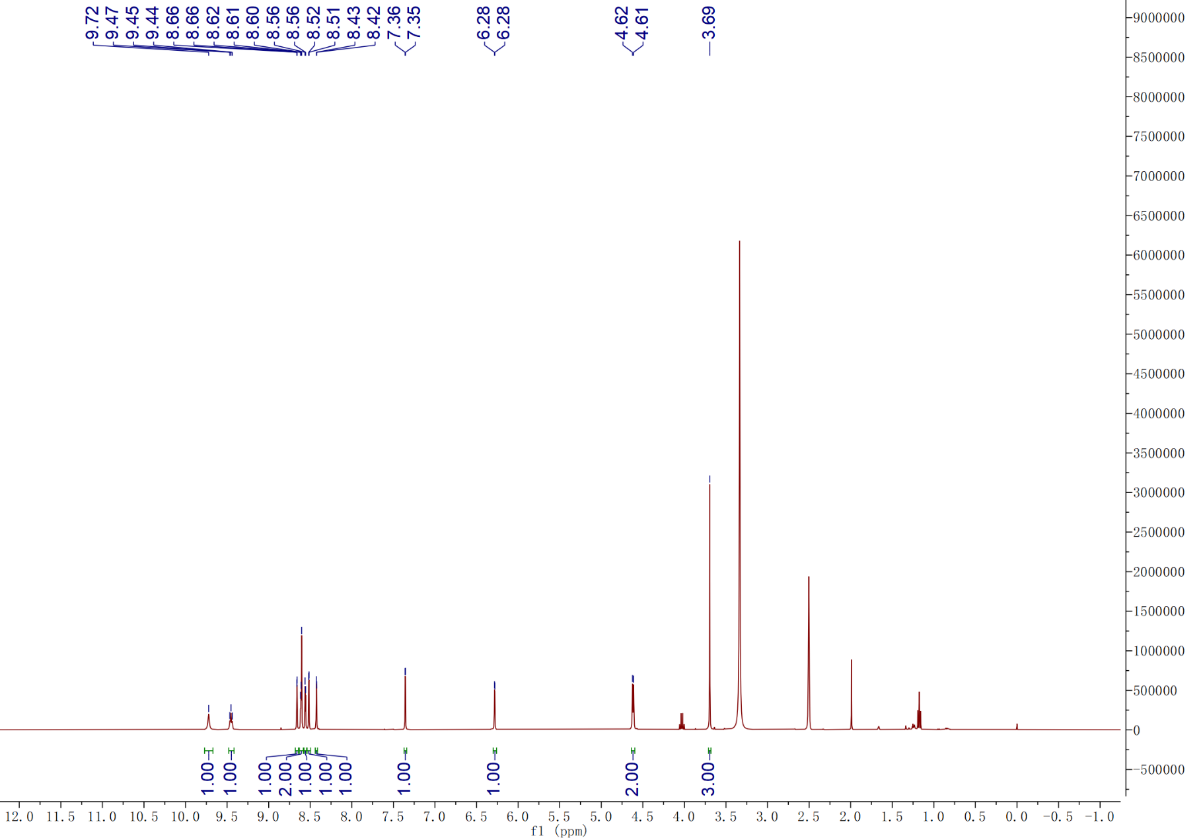


The ^13^C-NMR spectrum of compound **6h**


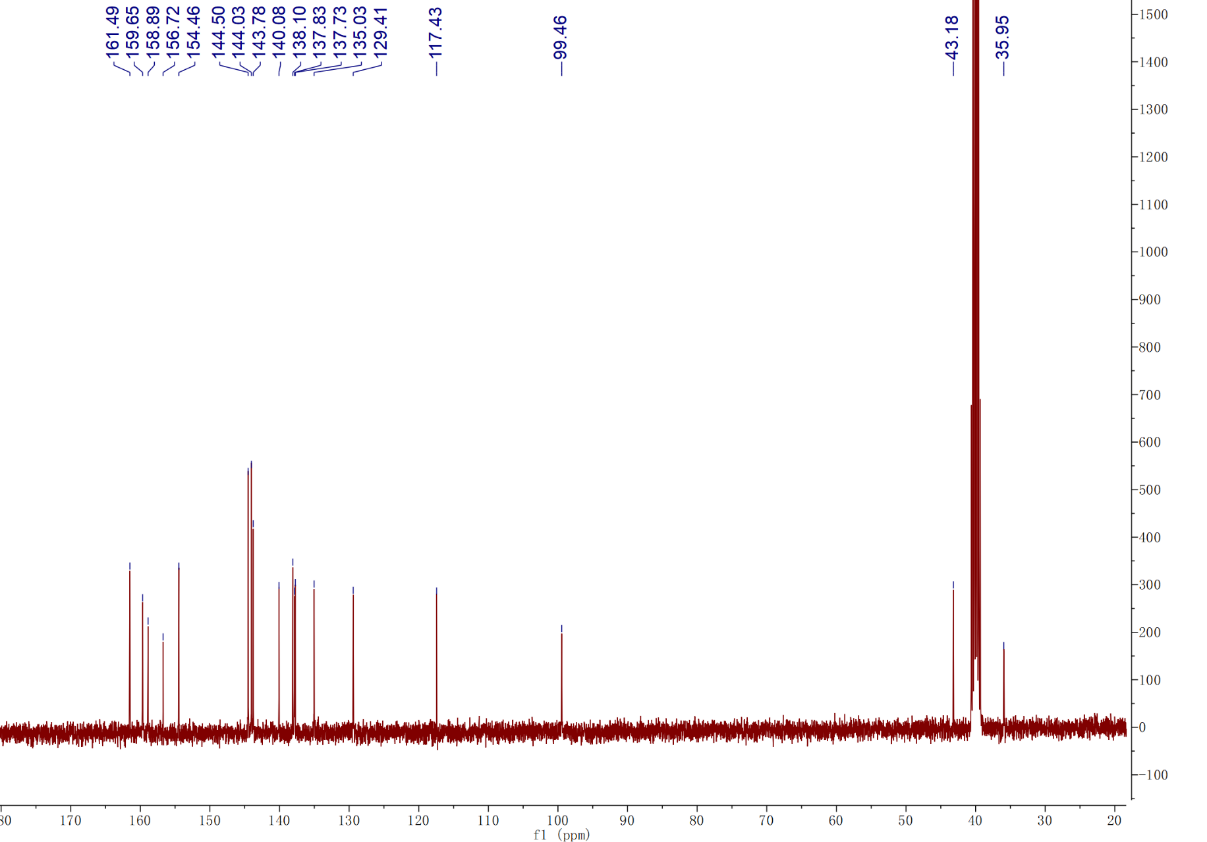


The MS data of compound **6h**


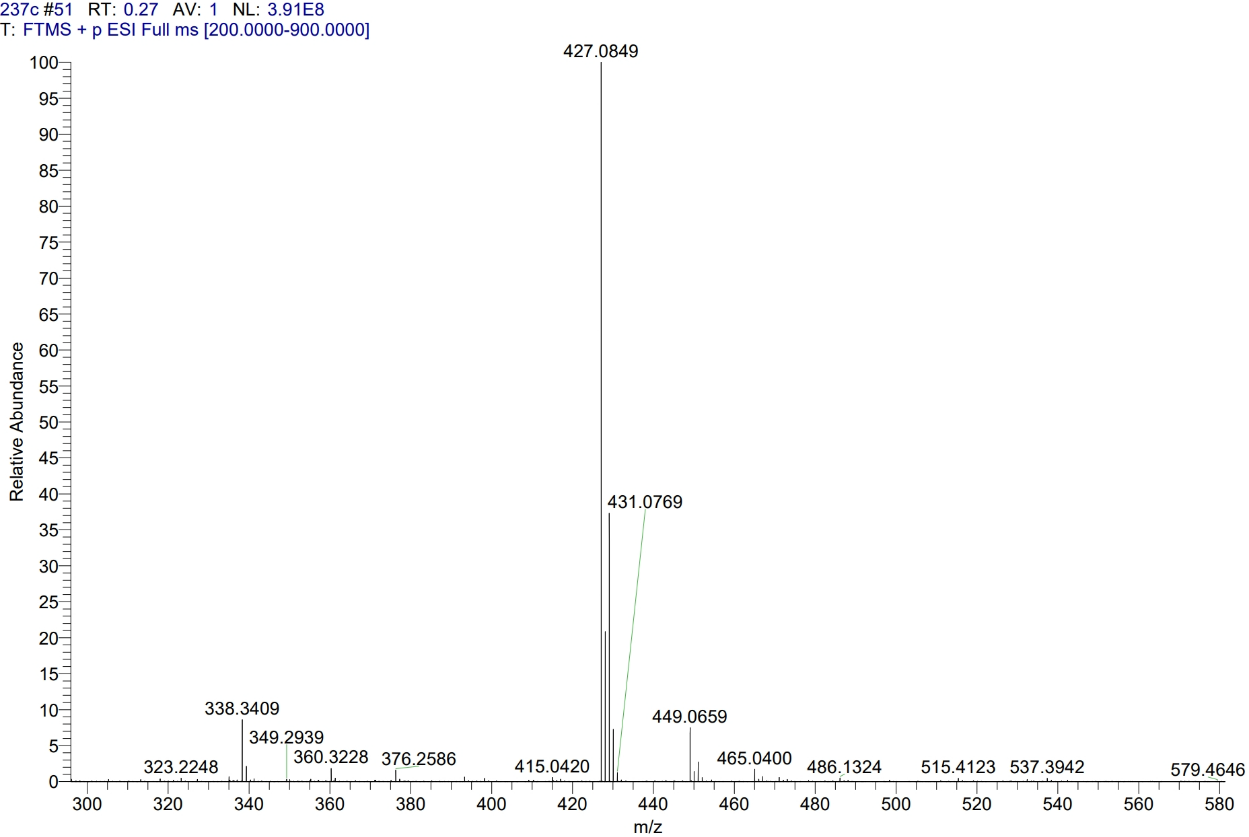


The HPLC spectrum of compound **6h**


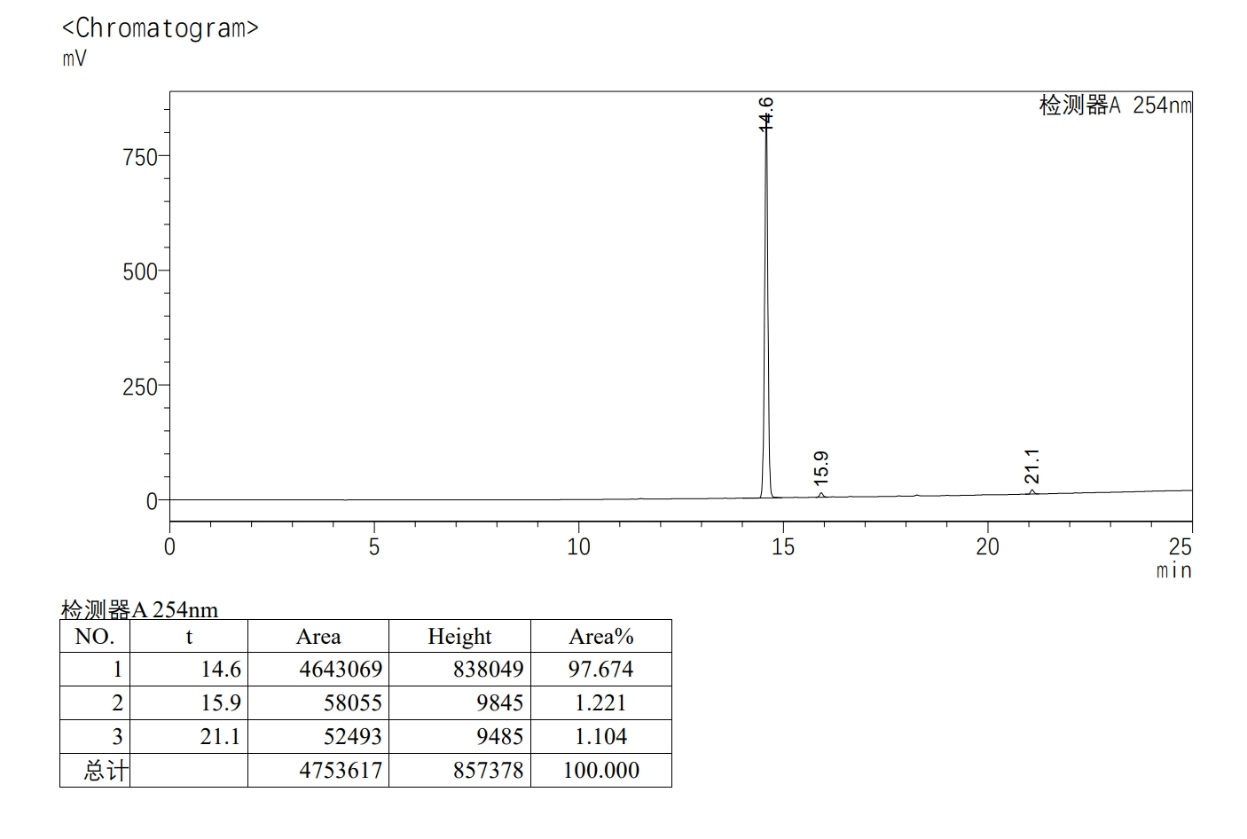


The ^1^H-NMR spectrum of compound **6i**


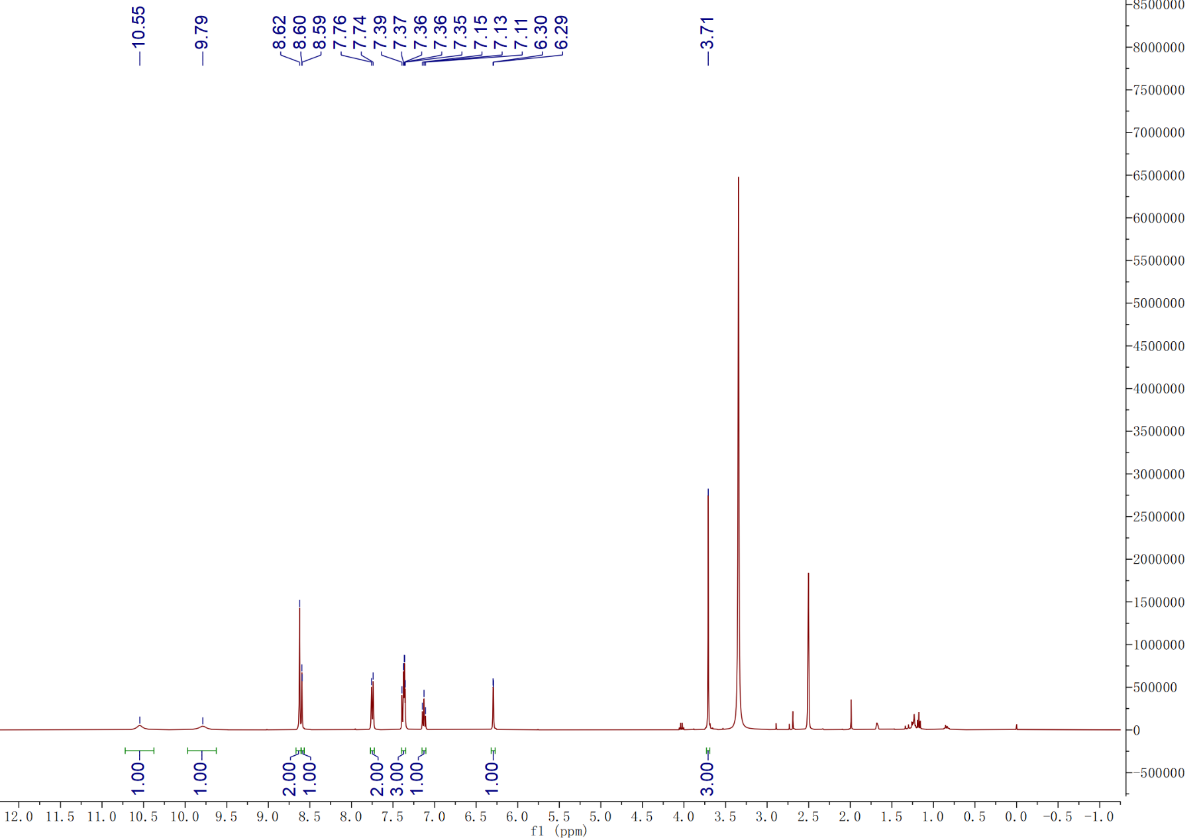


The ^13^C-NMR spectrum of compound **6i**


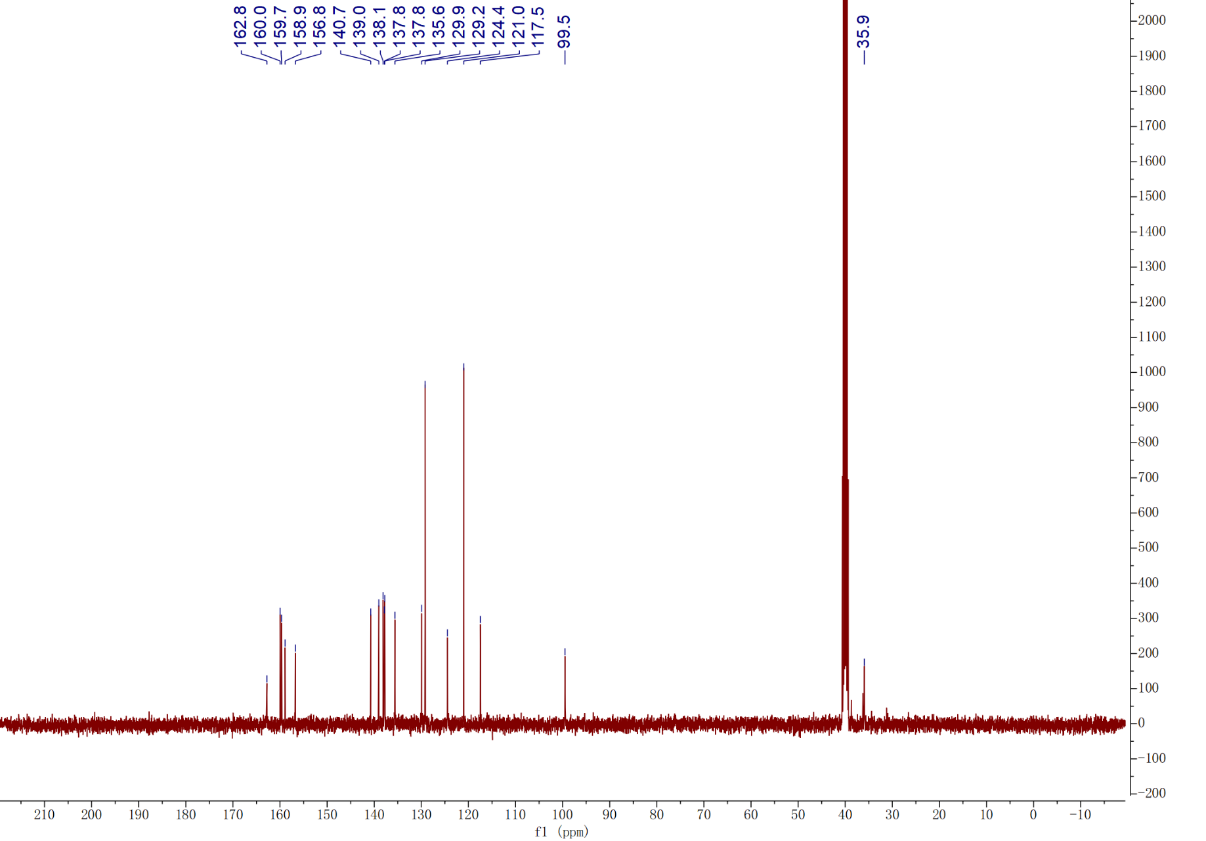


The MS data of compound **6i**


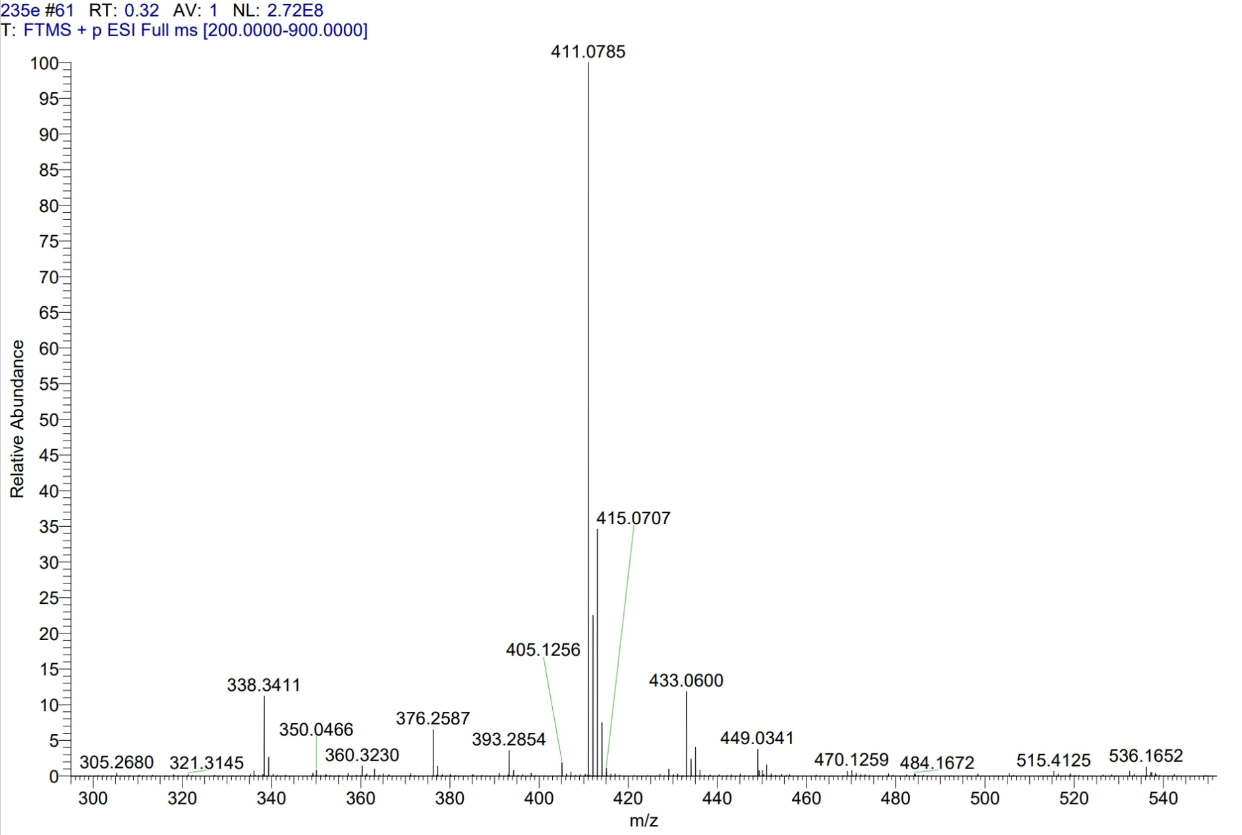


The HPLC spectrum of compound **6i**


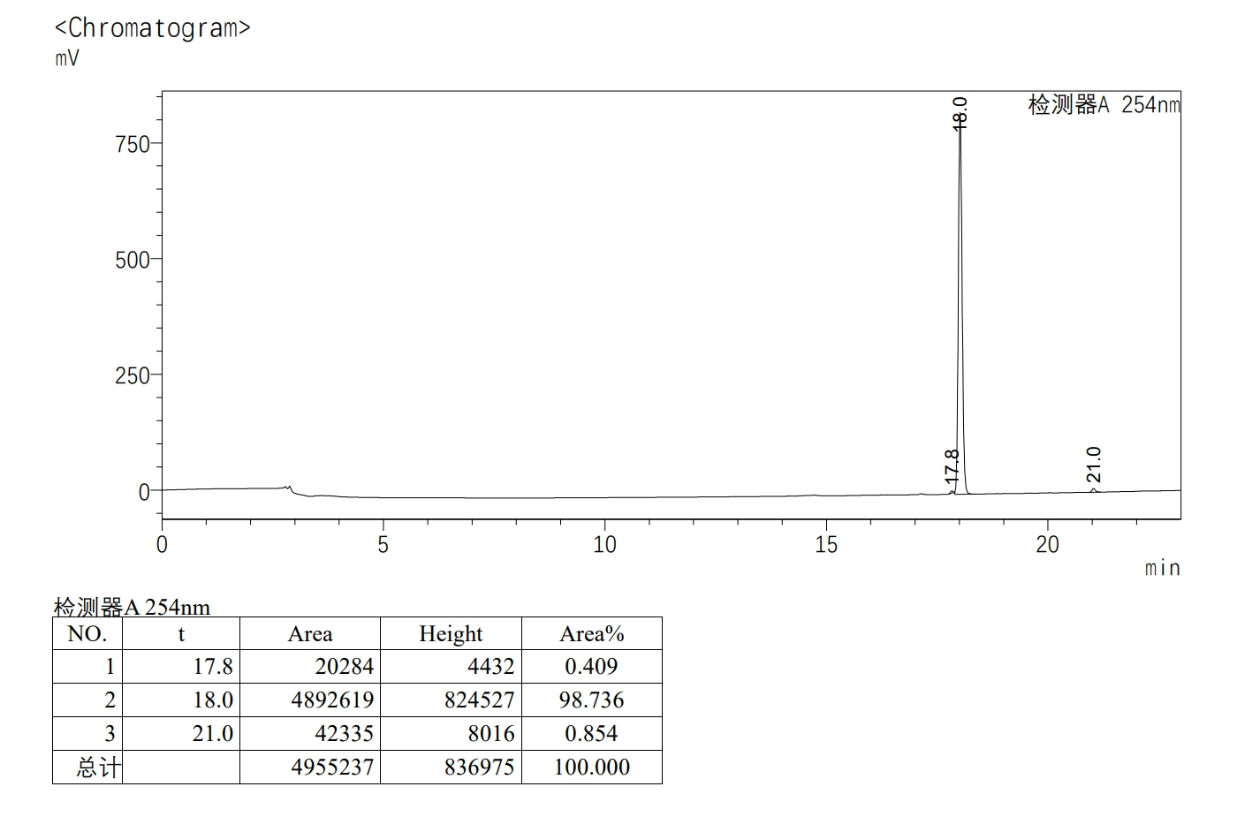


The ^1^H-NMR spectrum of compound **6j**


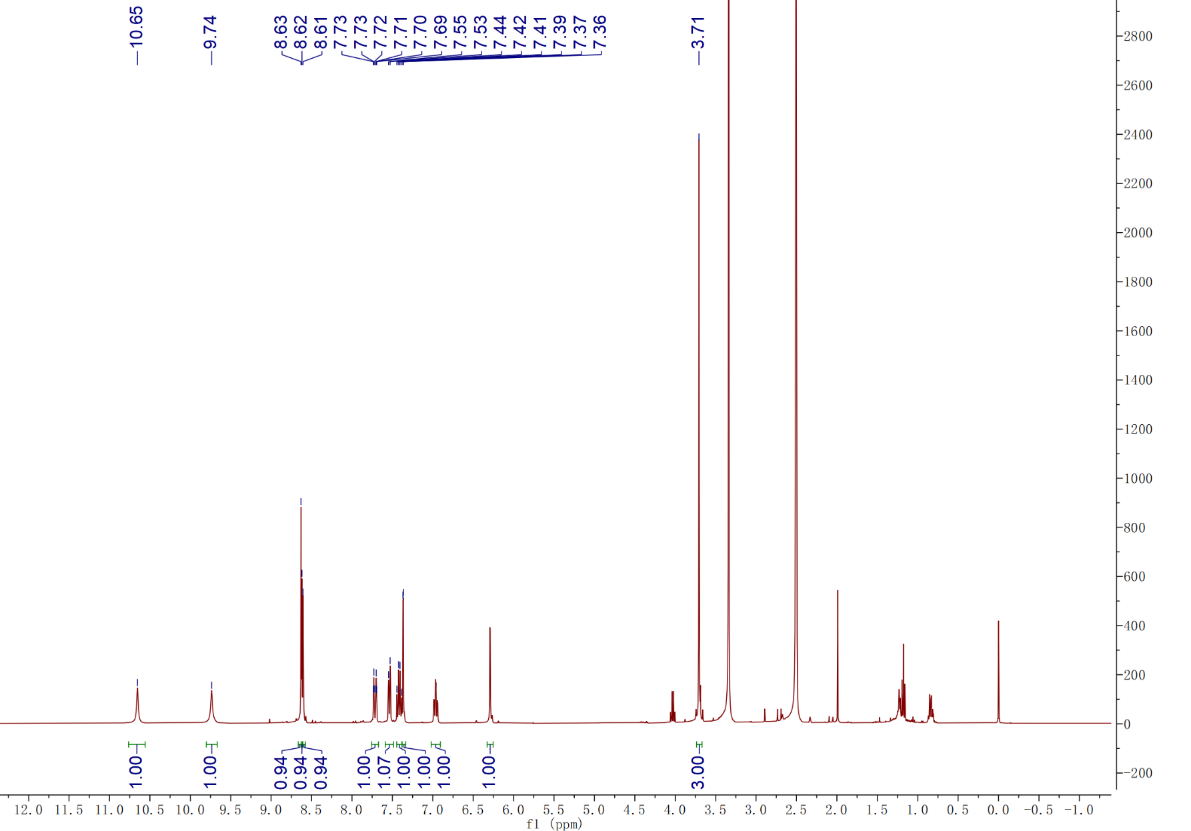


The ^13^C-NMR spectrum of compound **6j**


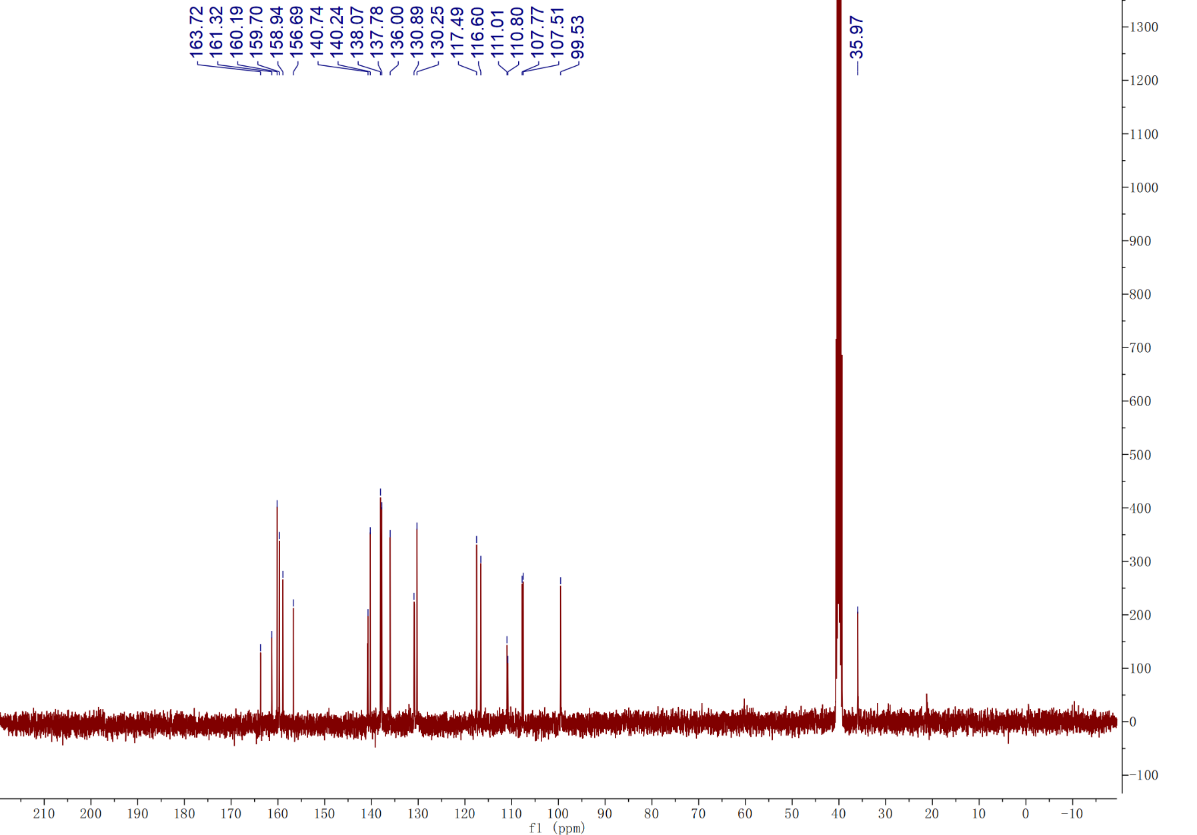


The MS data of compound **6j**


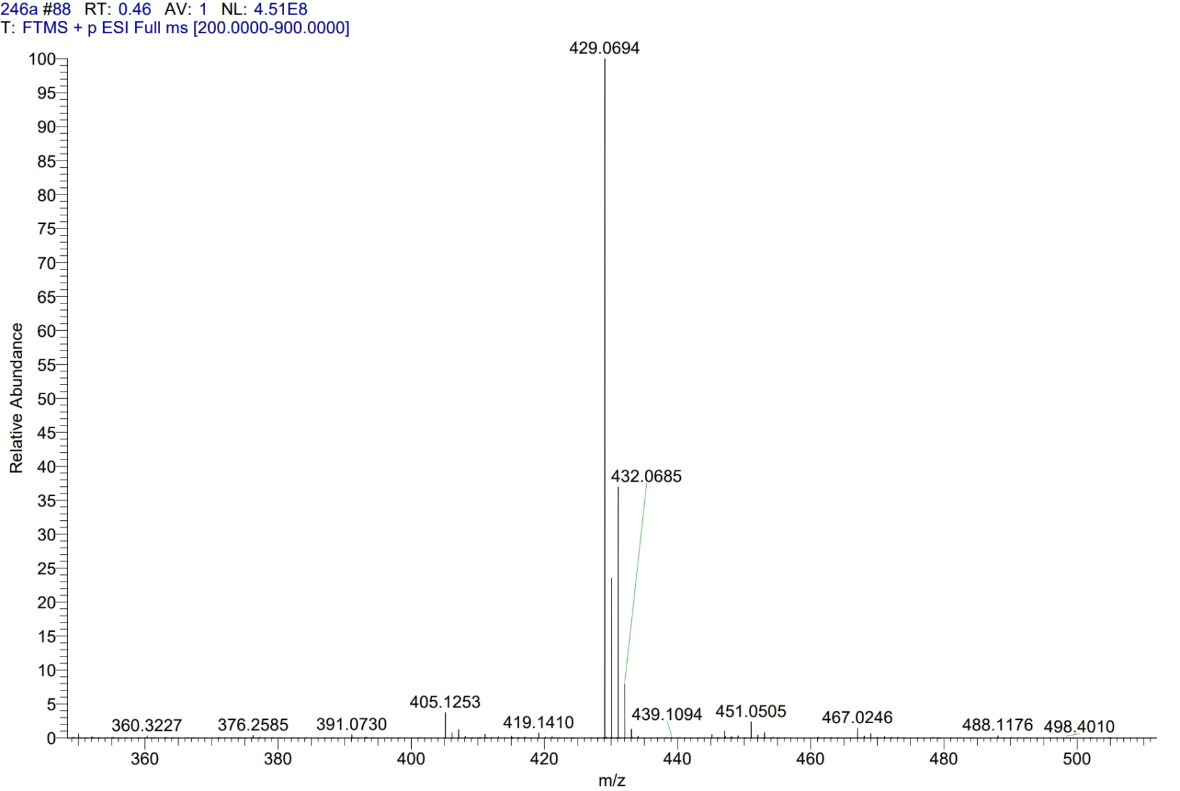


The HPLC spectrum of compound **6j**


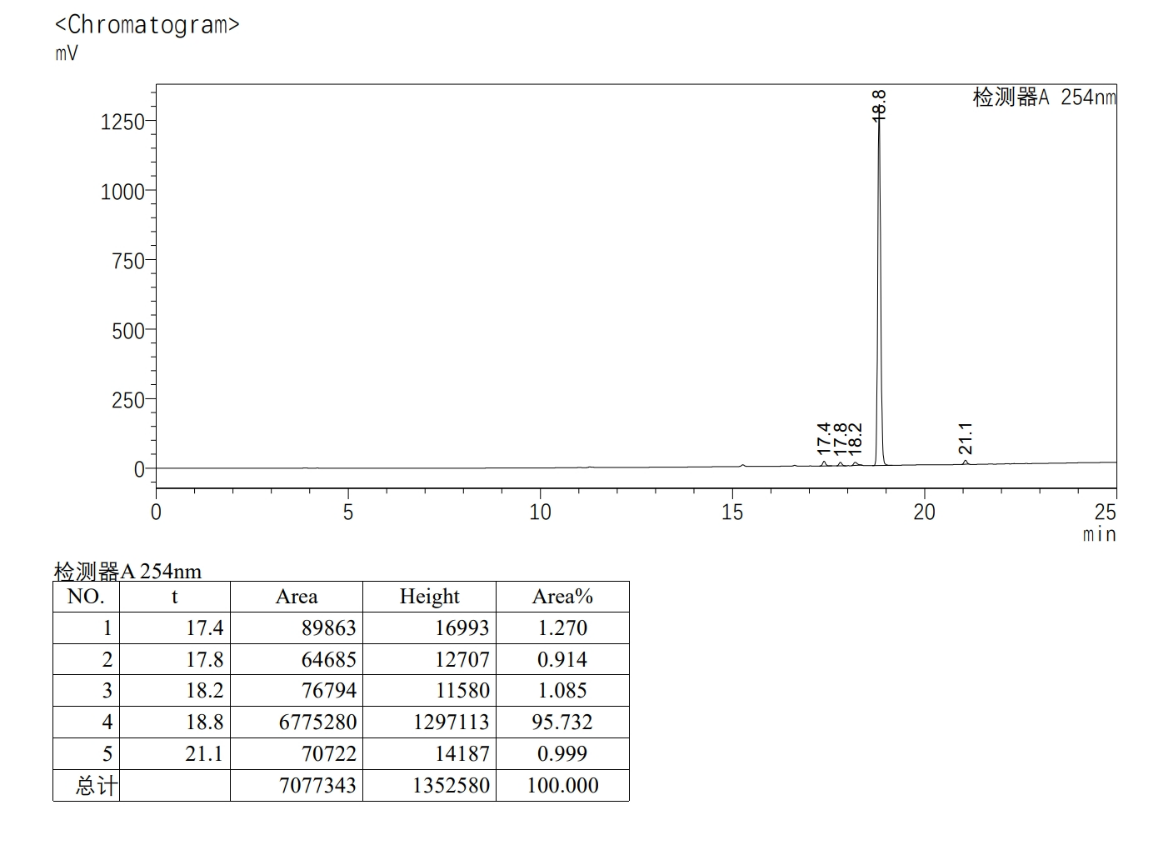


The ^1^H-NMR spectrum of compound **6k**


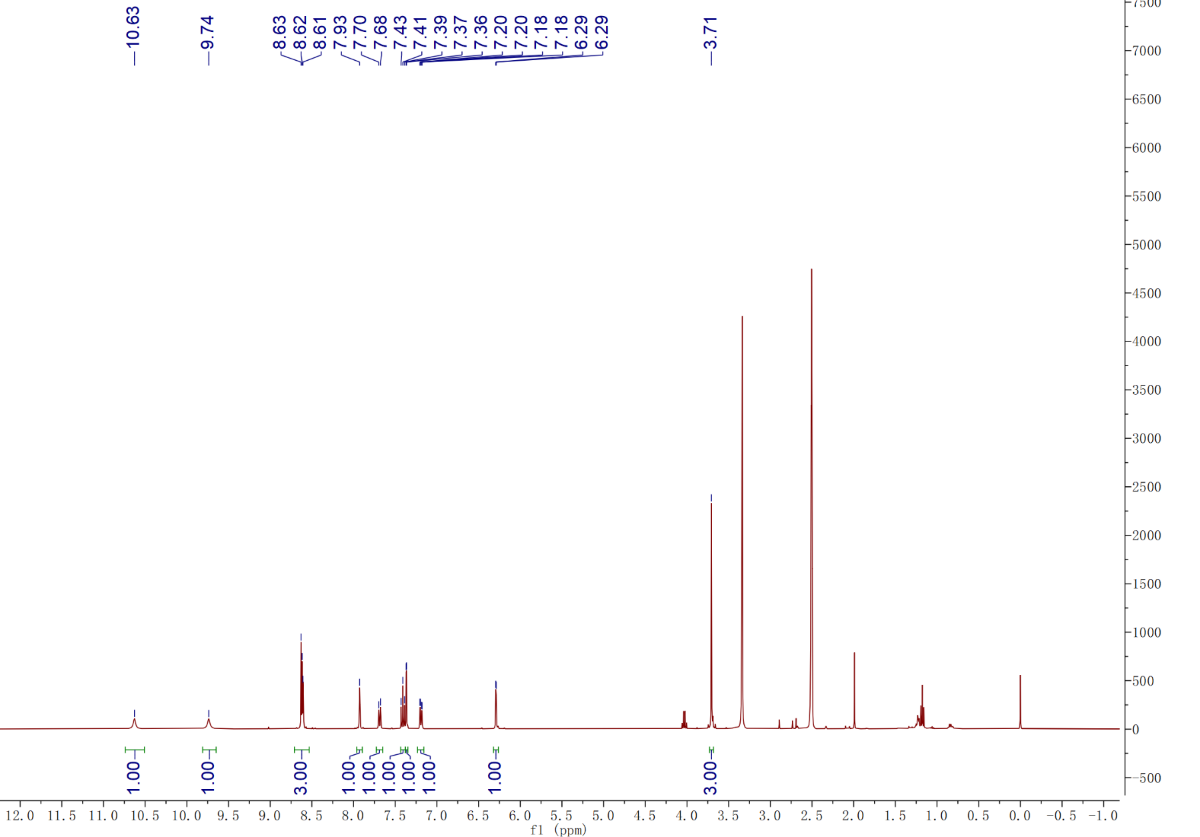


The ^13^C-NMR spectrum of compound **6k**


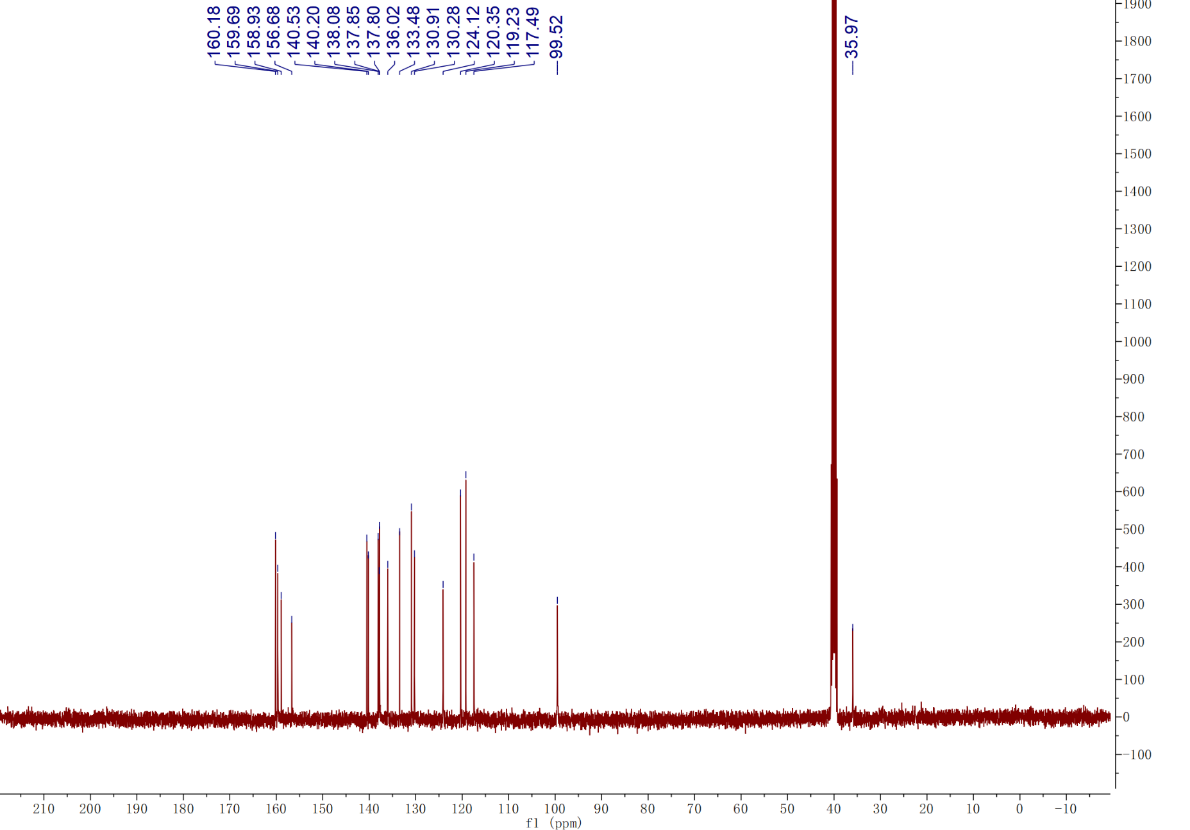


The MS data of compound **6k**


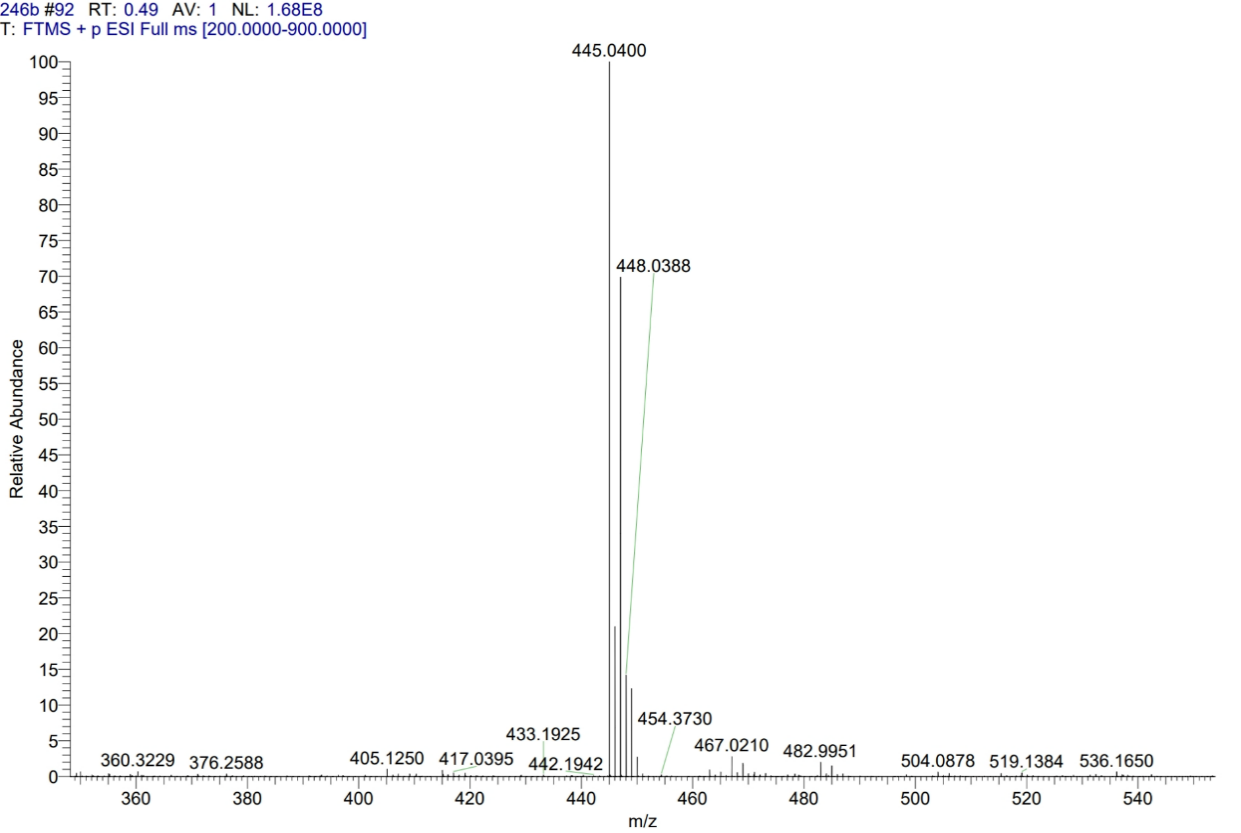


The HPLC spectrum of compound **6k**


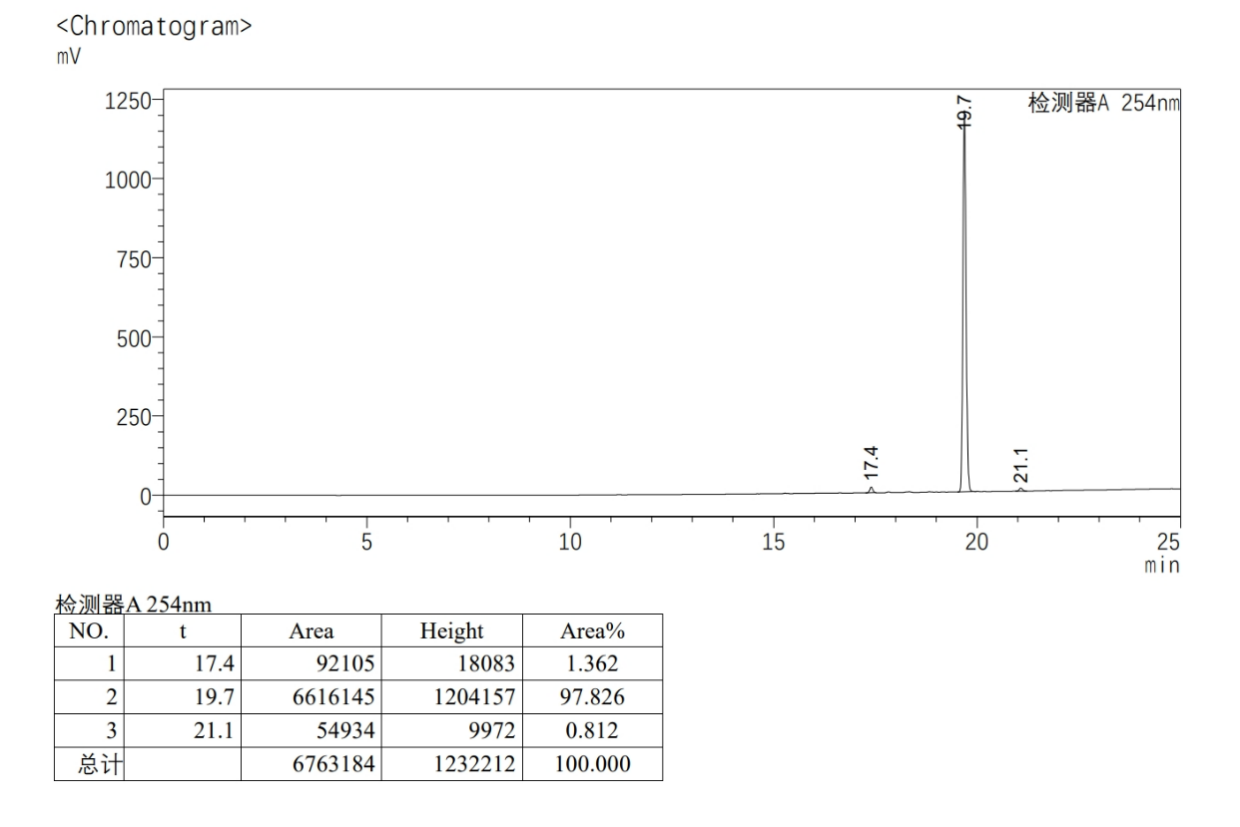


The ^1^H-NMR spectrum of compound **6l**


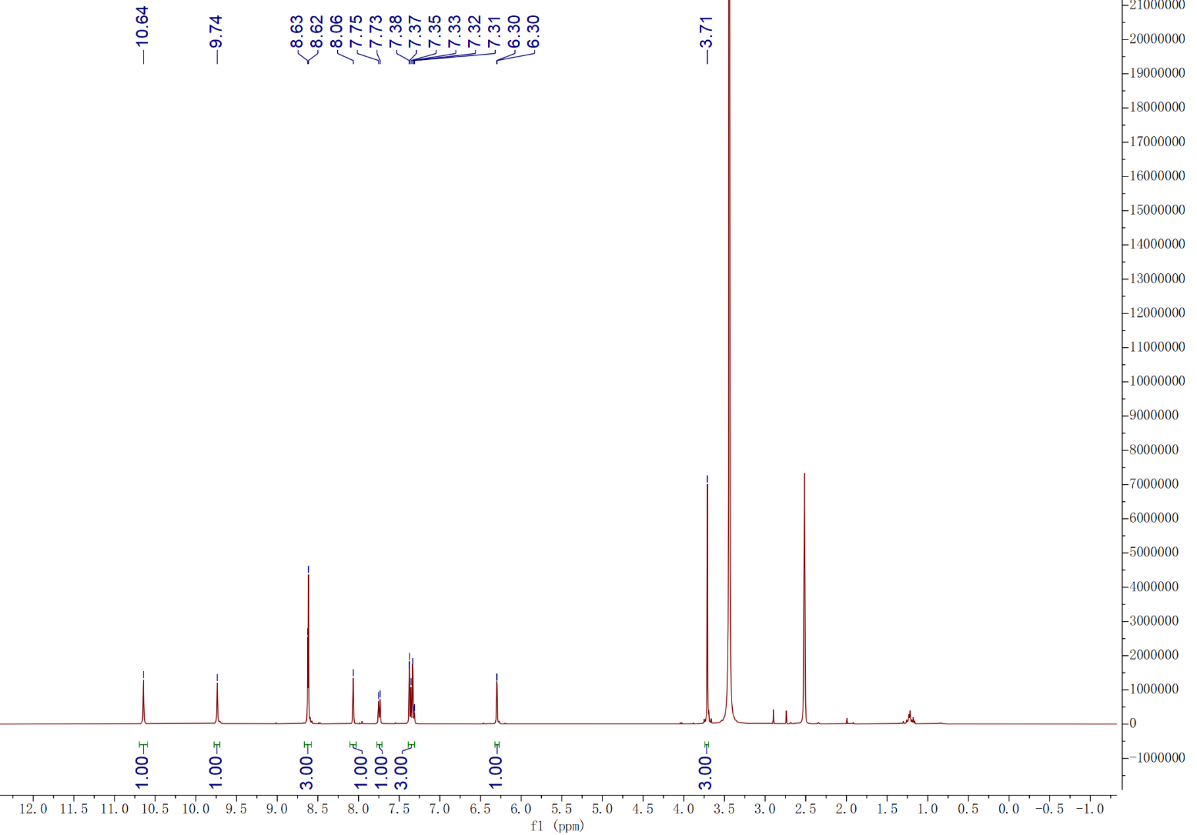


The ^13^C-NMR spectrum of compound **6l**


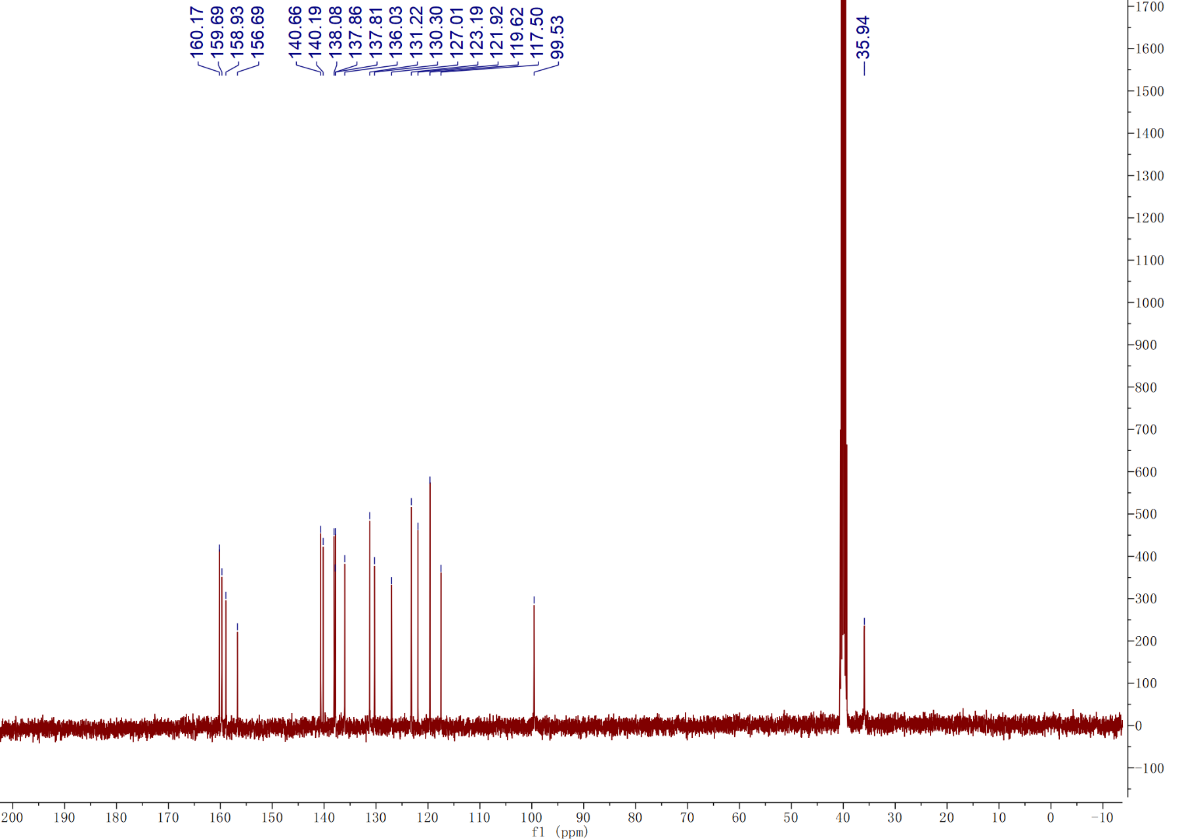


The MS data of compound **6l**


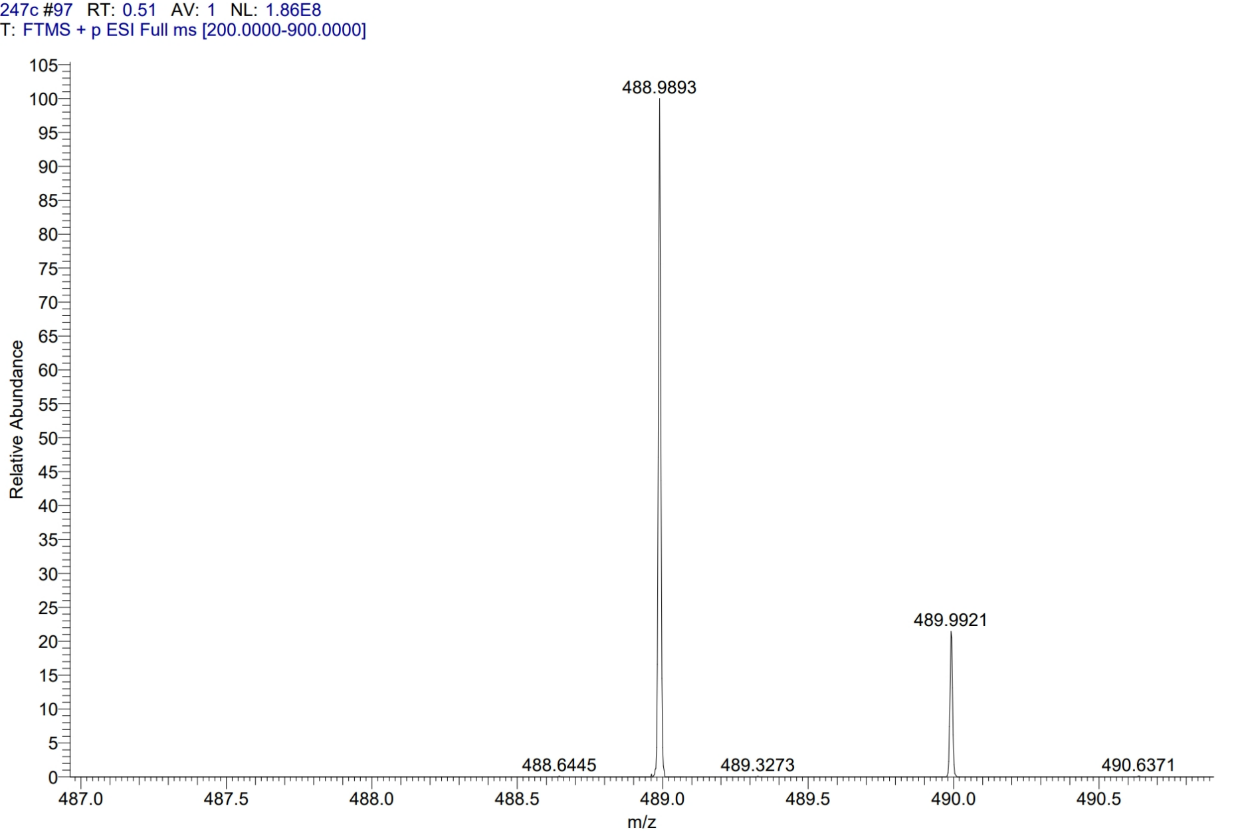


The HPLC spectrum of compound **6l**


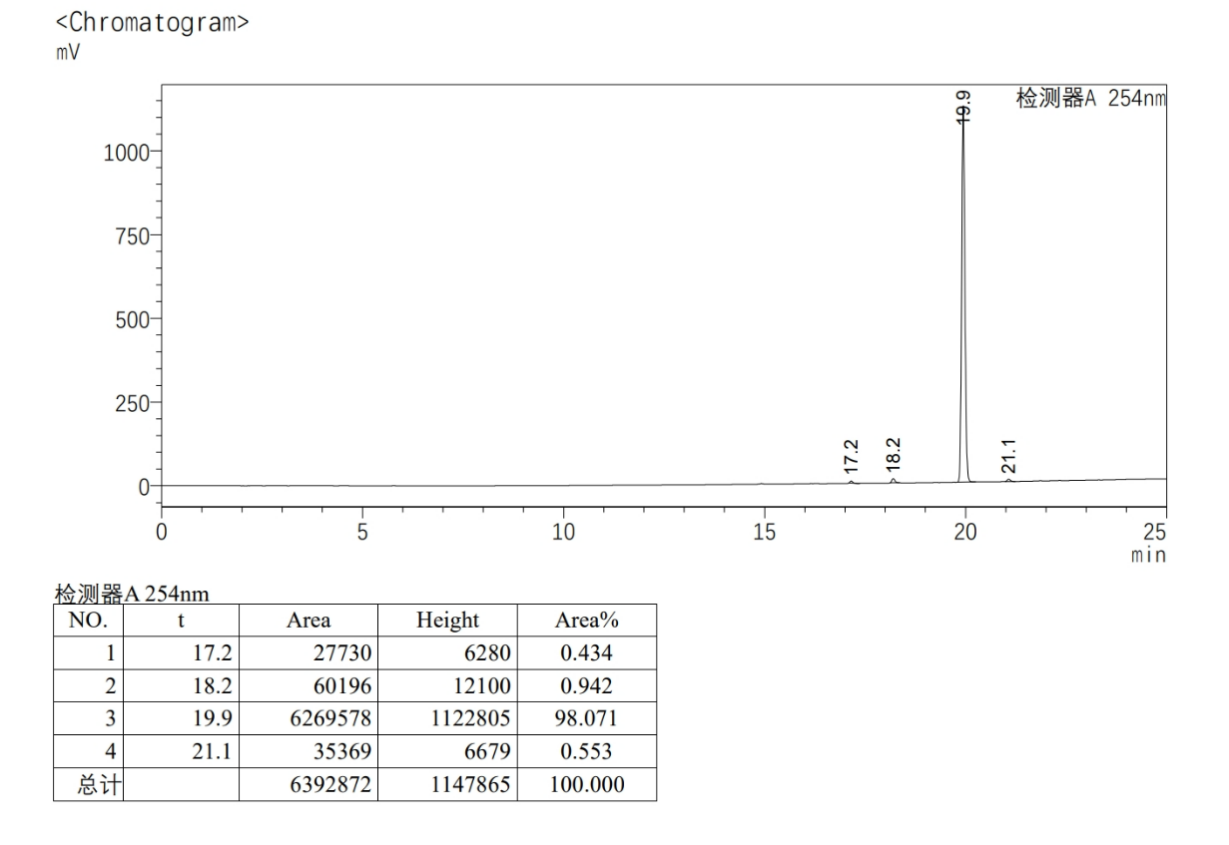


The ^1^H-NMR spectrum of compound **6m**


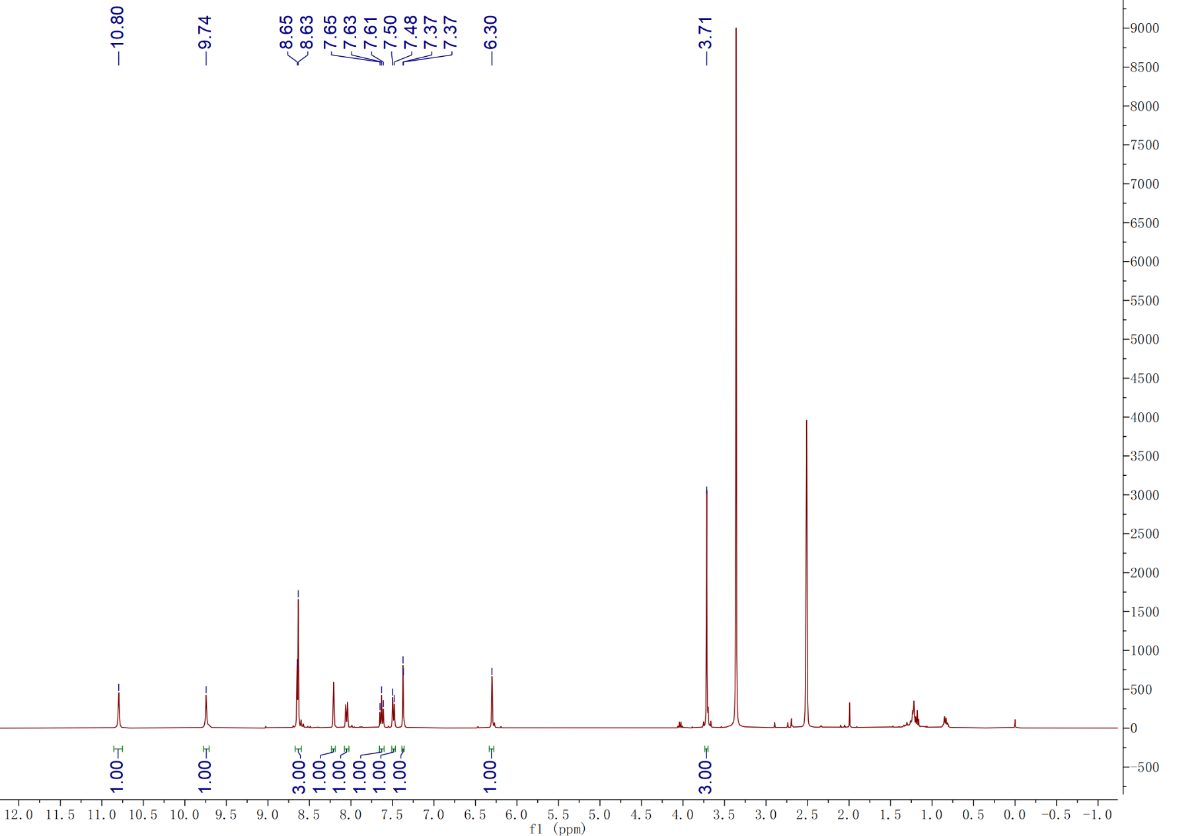


The ^13^C-NMR spectrum of compound **6m**


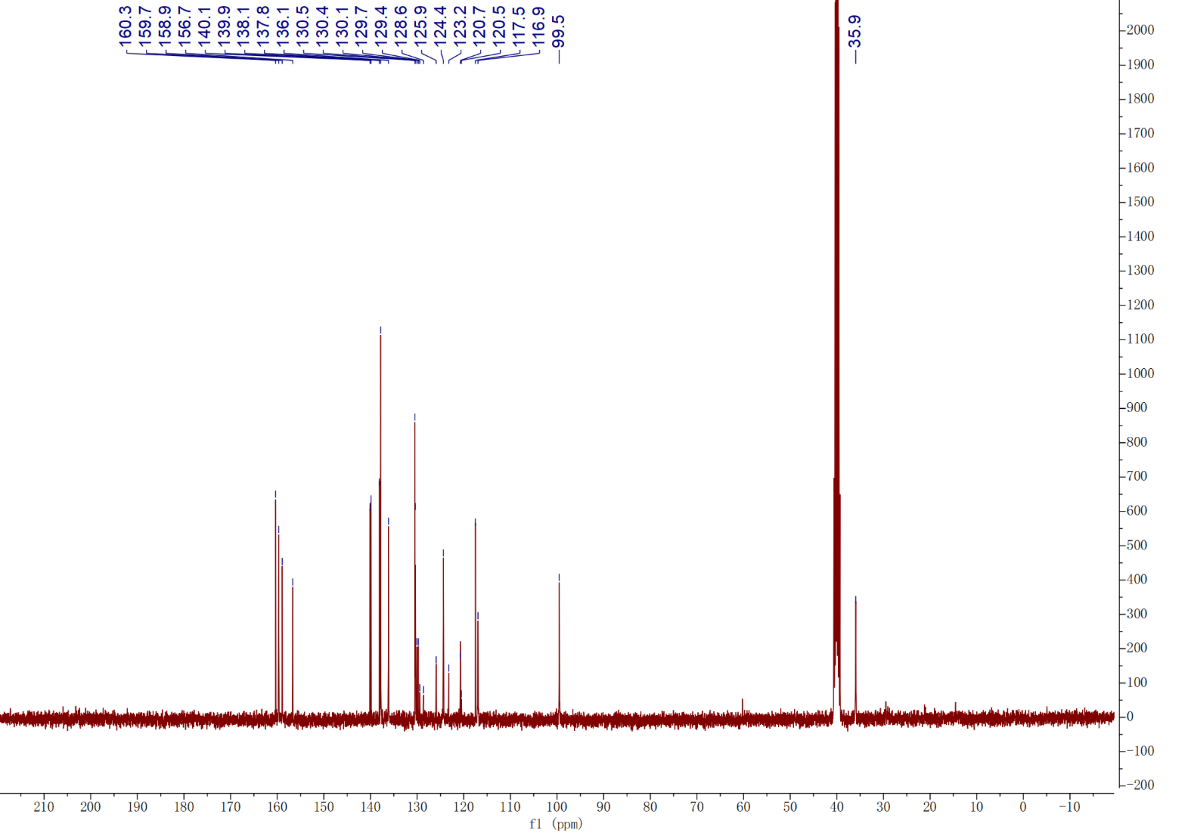


The MS data of compound **6m**


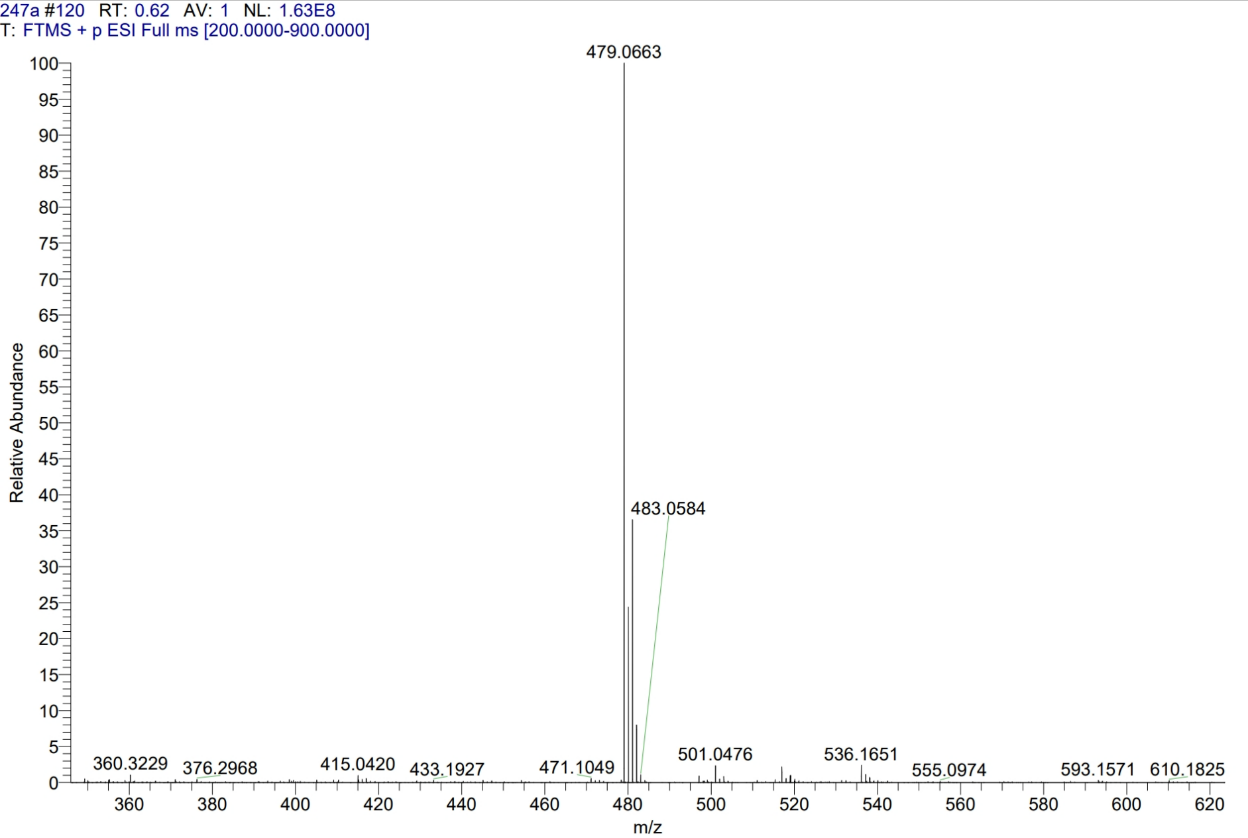


The HPLC spectrum of compound **6m**


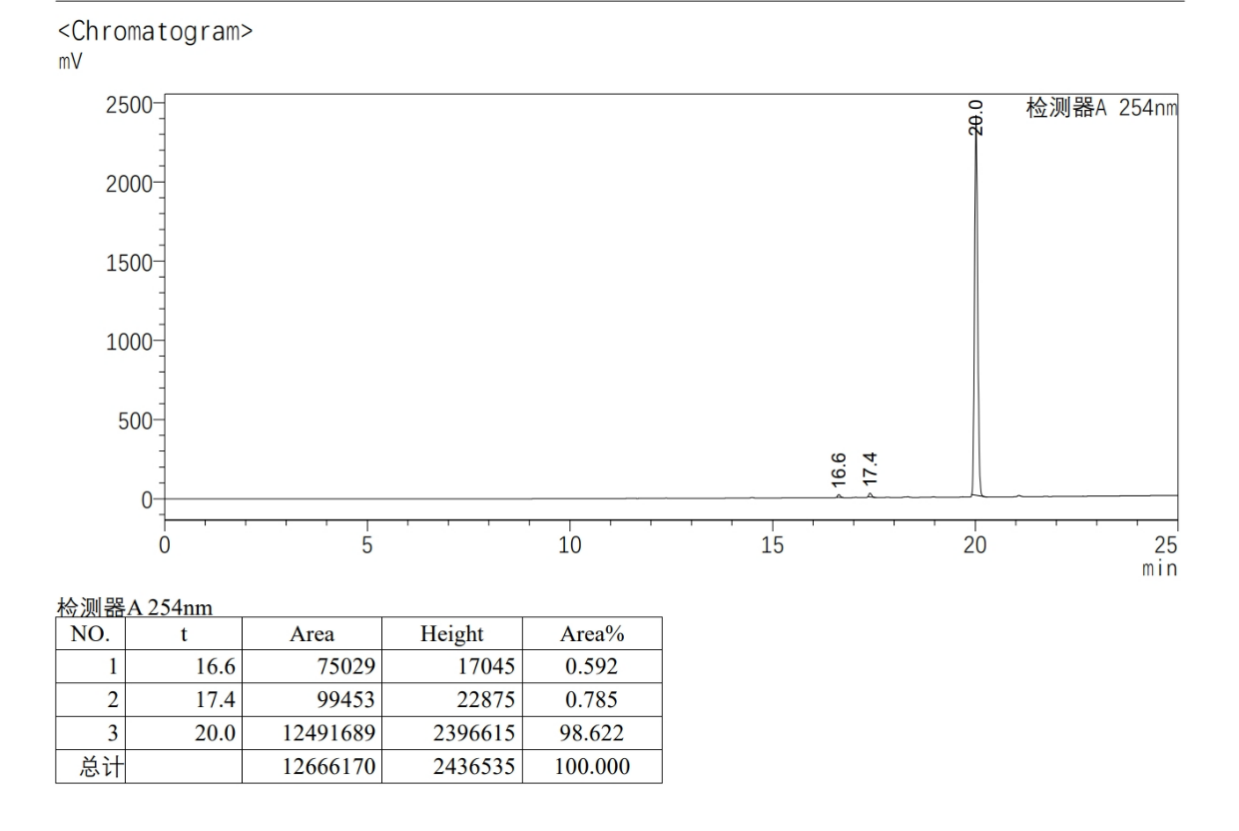


The ^1^H-NMR spectrum of compound **6n**


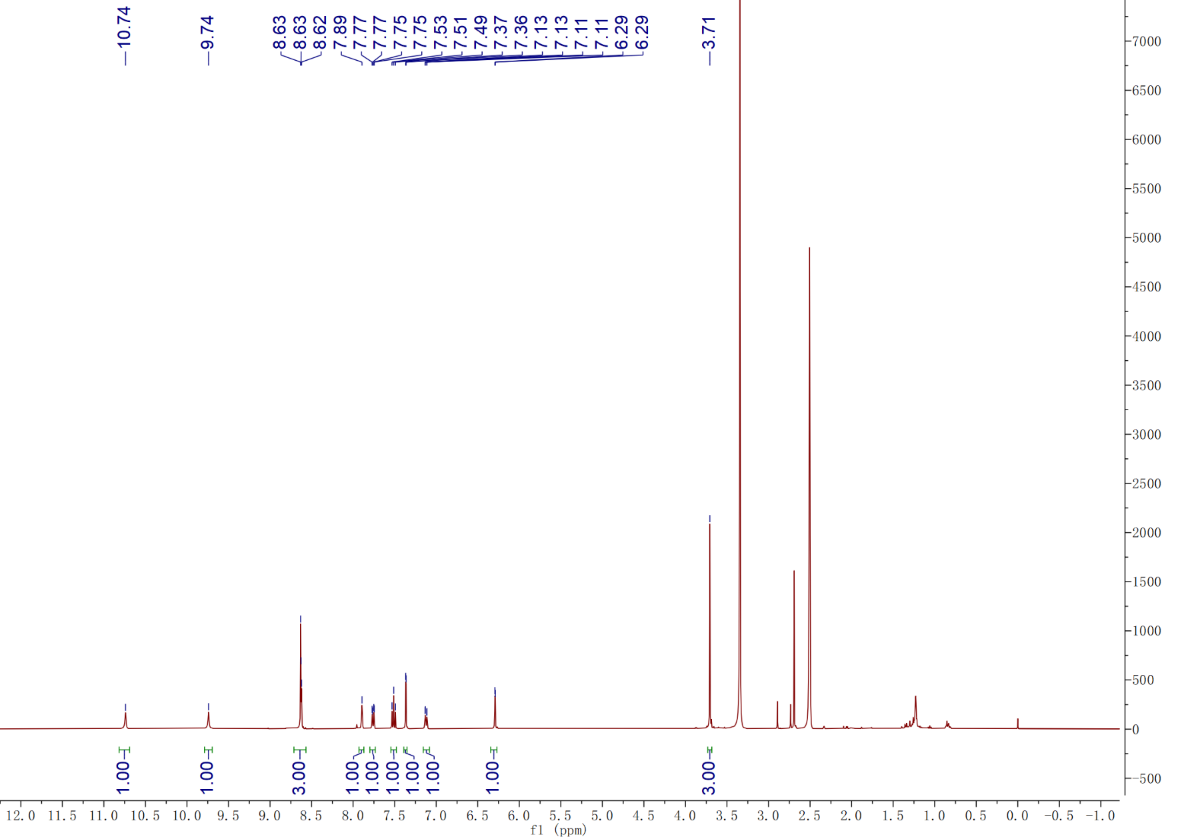


The ^13^C-NMR spectrum of compound **6n**


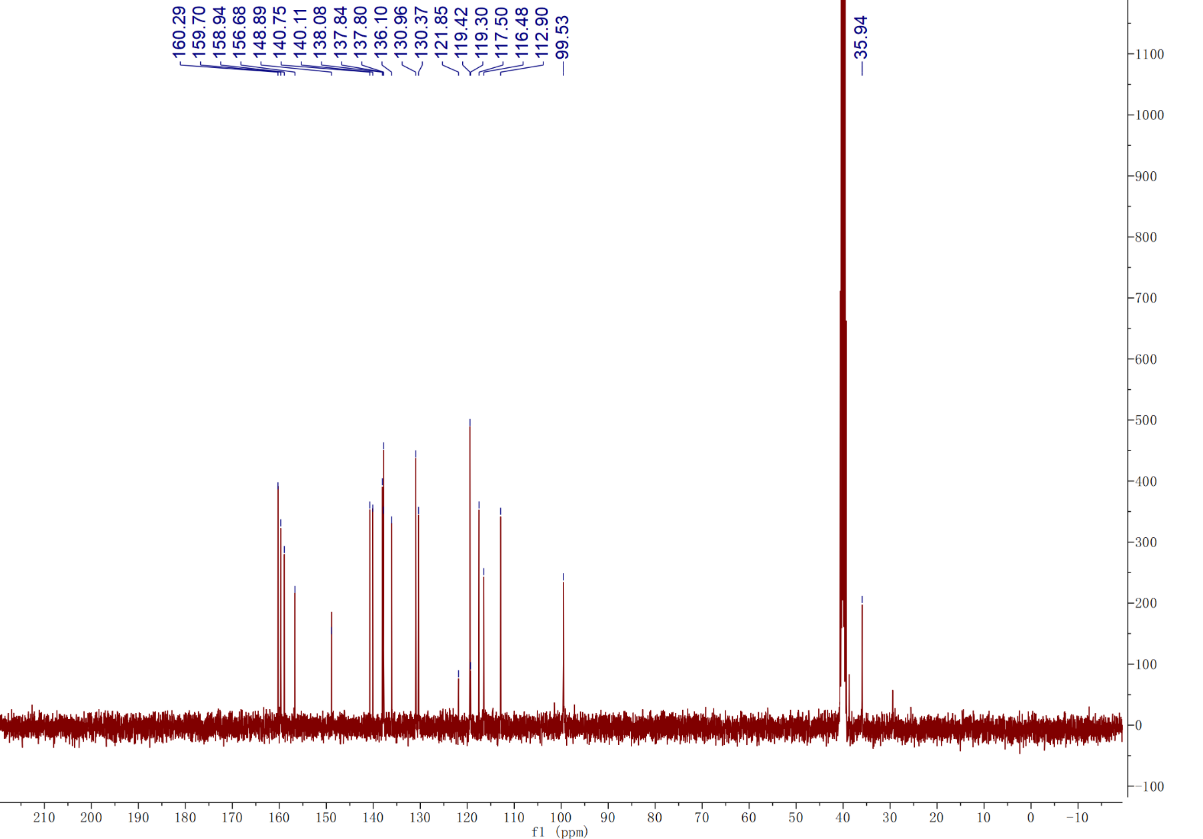


The MS data of compound **6n**


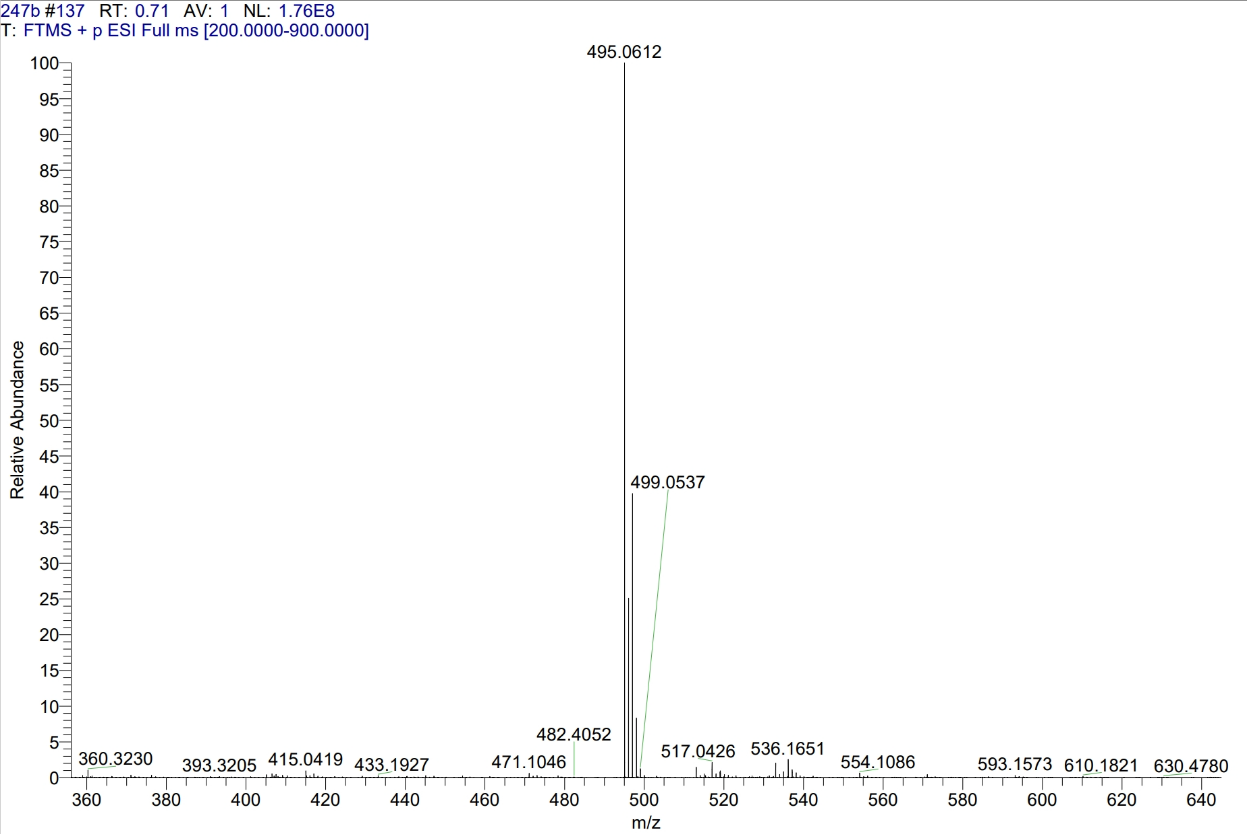


The HPLC spectrum of compound **6n**


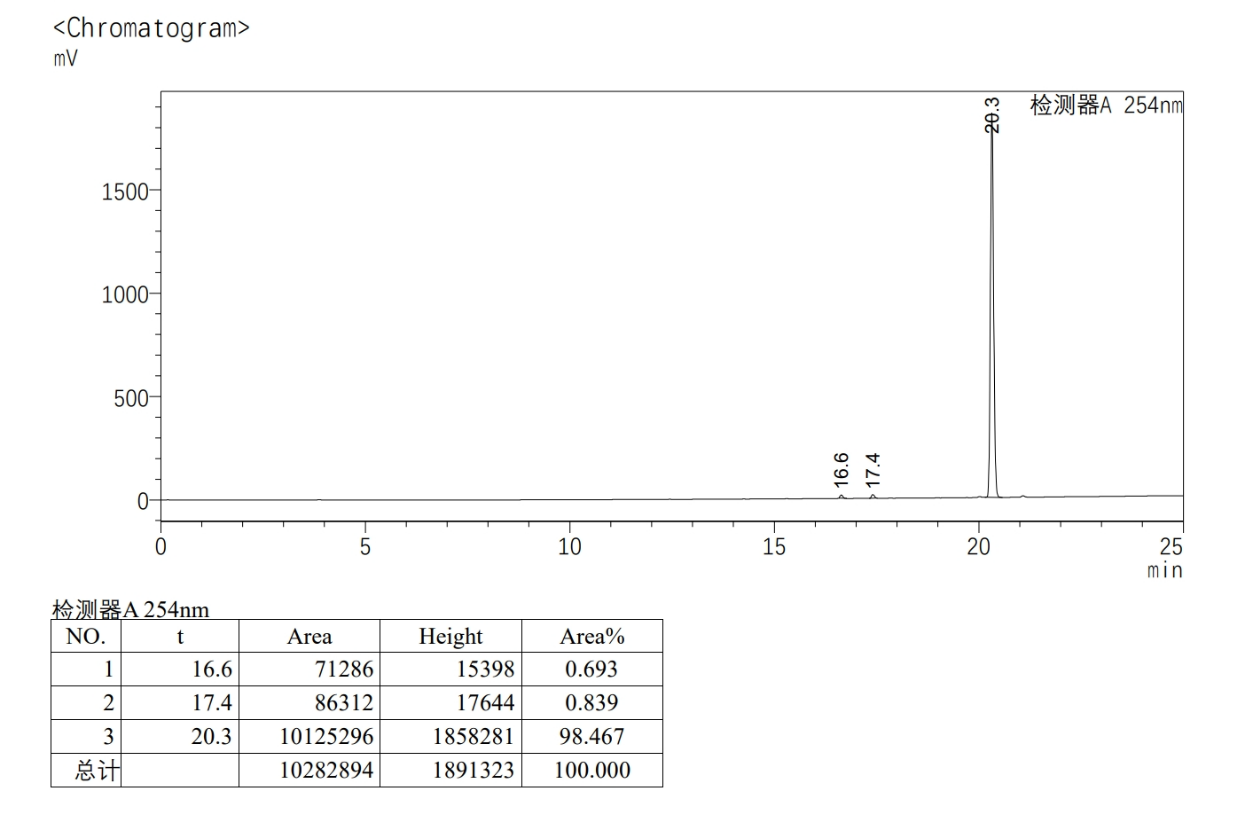


The ^1^H-NMR spectrum of compound **6o**


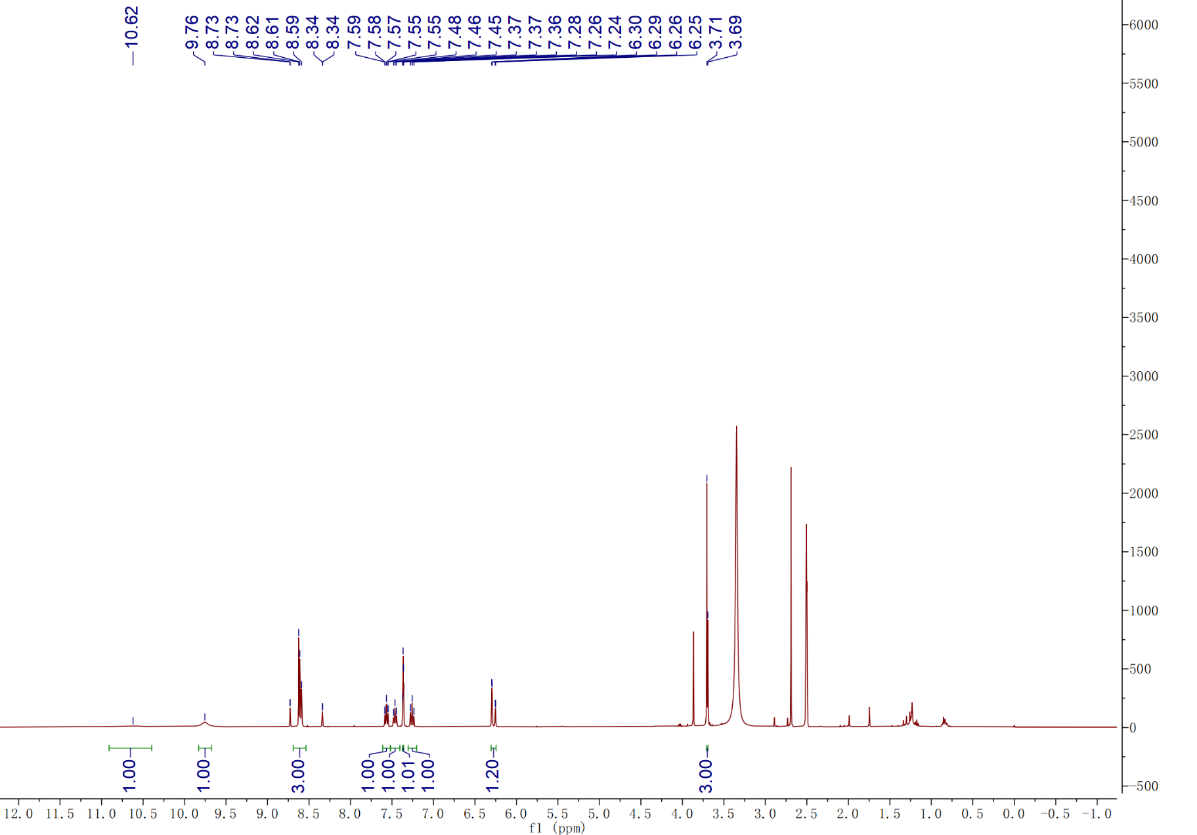


The ^13^C-NMR spectrum of compound **6o**


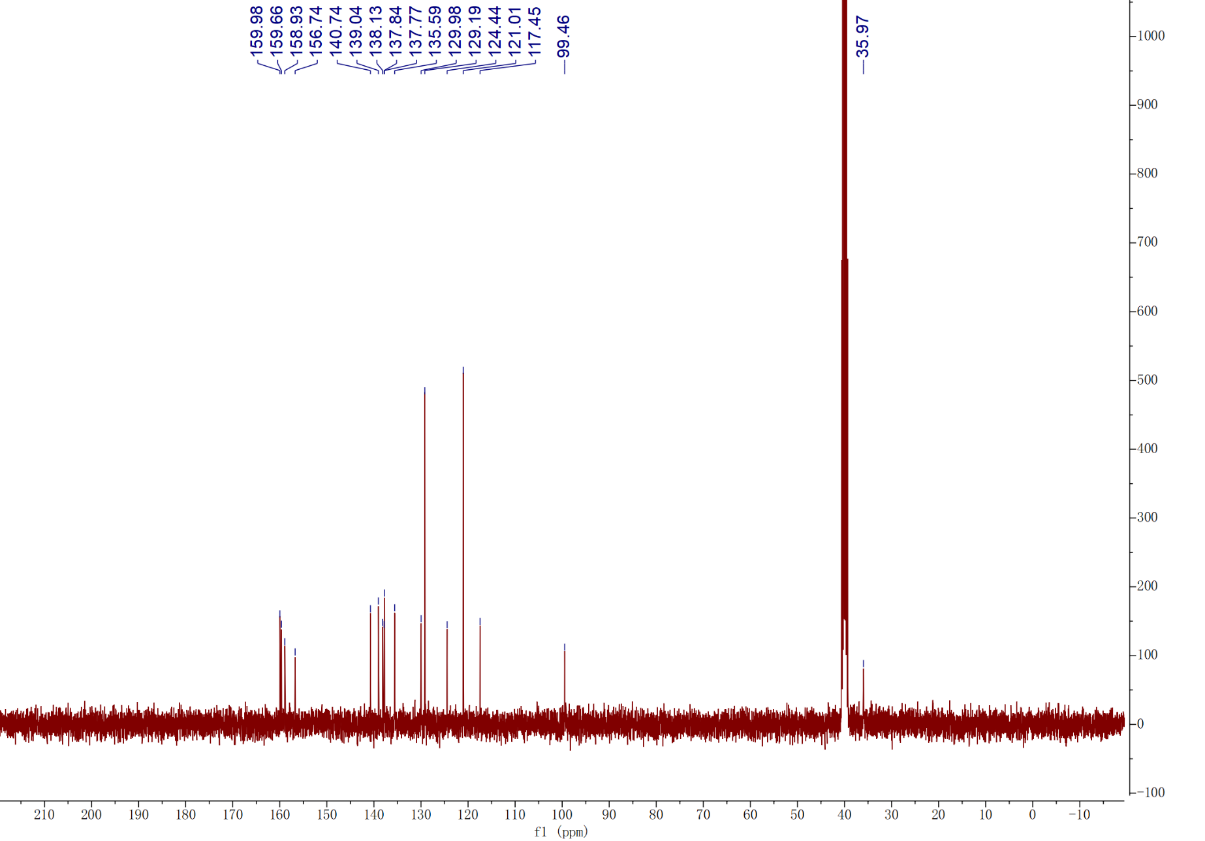


The MS data of compound **6o**


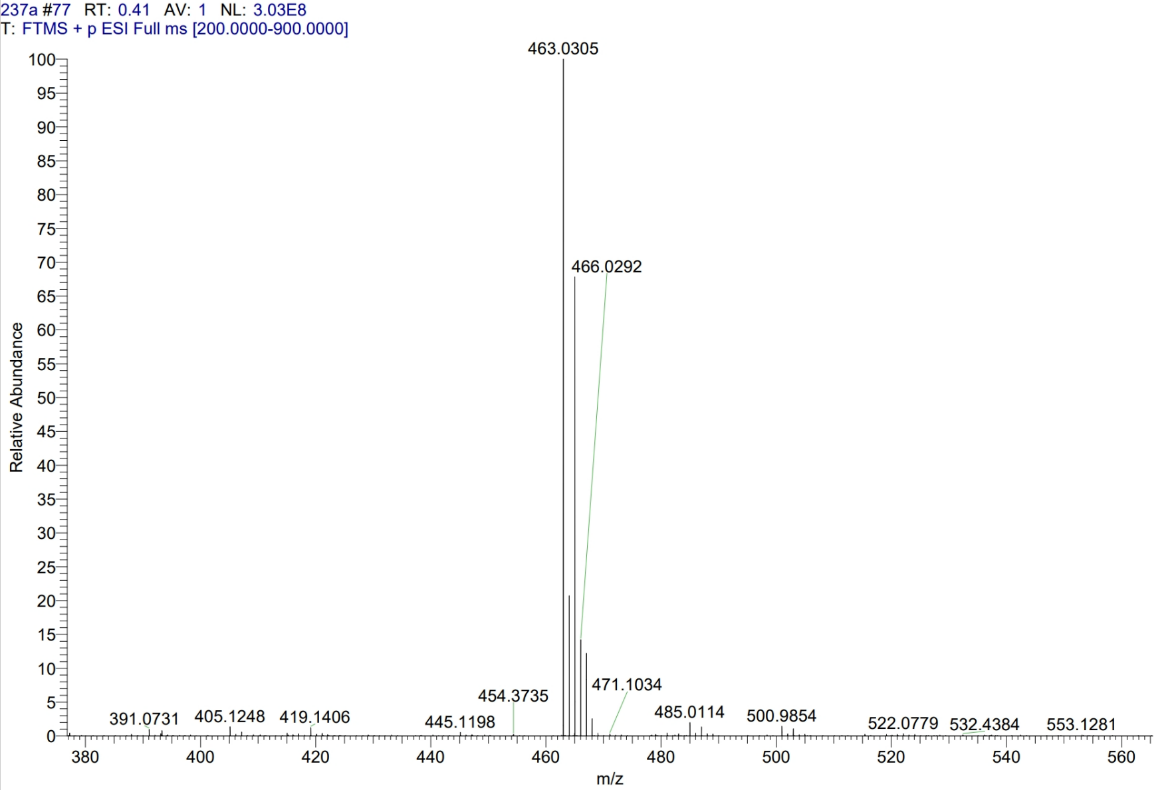


The HPLC spectrum of compound **6o**


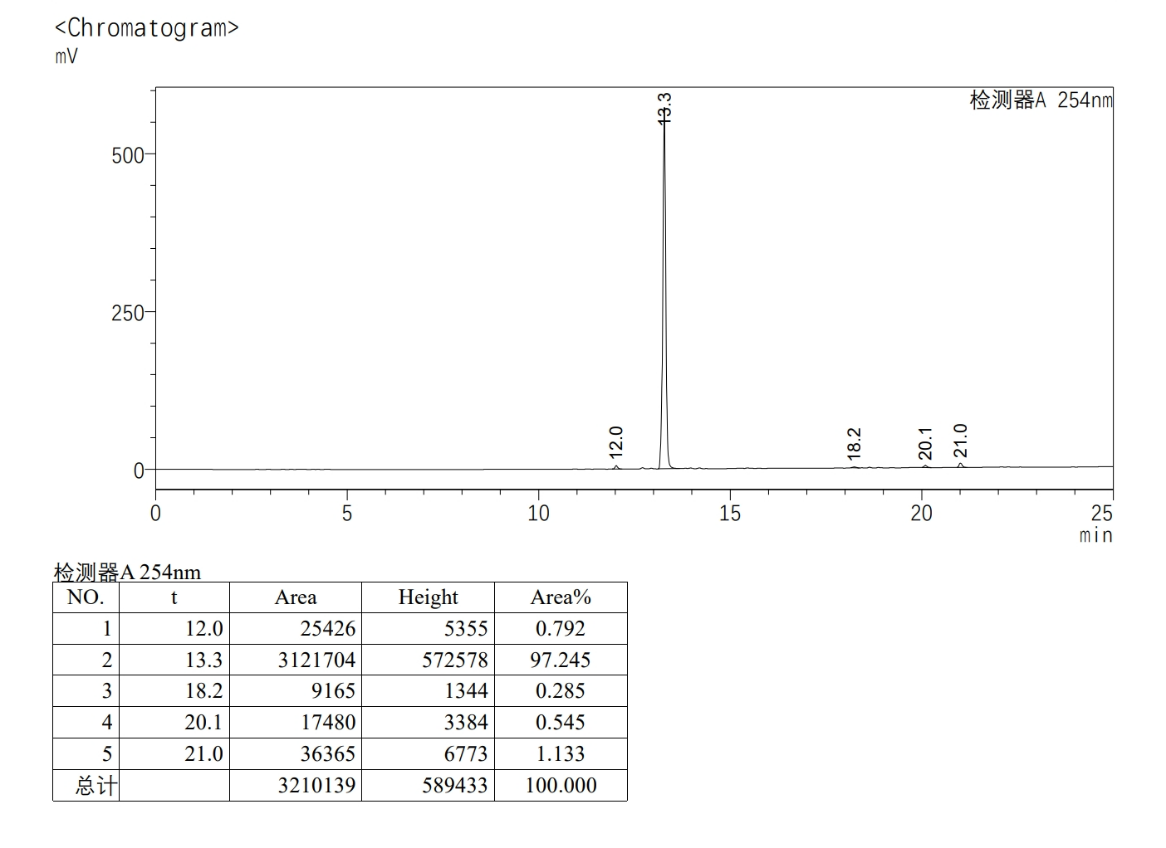


The ^1^H-NMR spectrum of compound **6p**


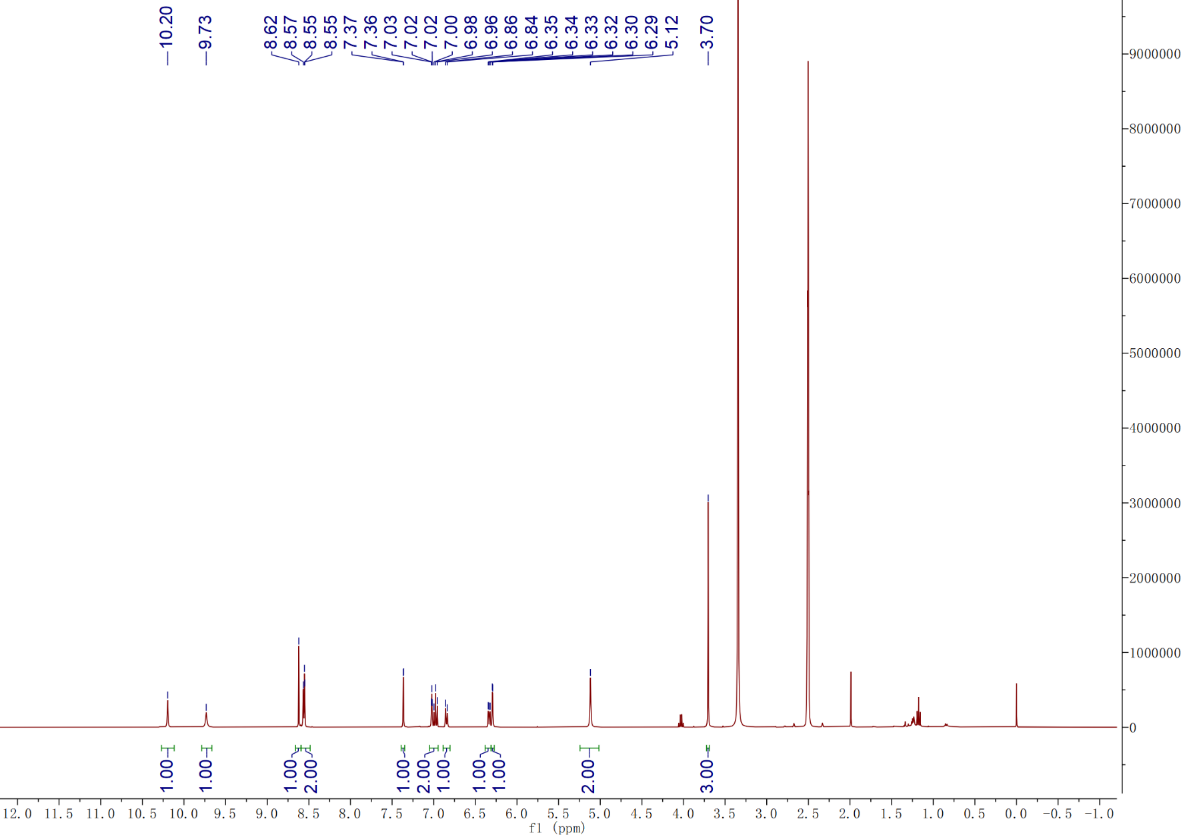


The ^13^C-NMR spectrum of compound **6p**


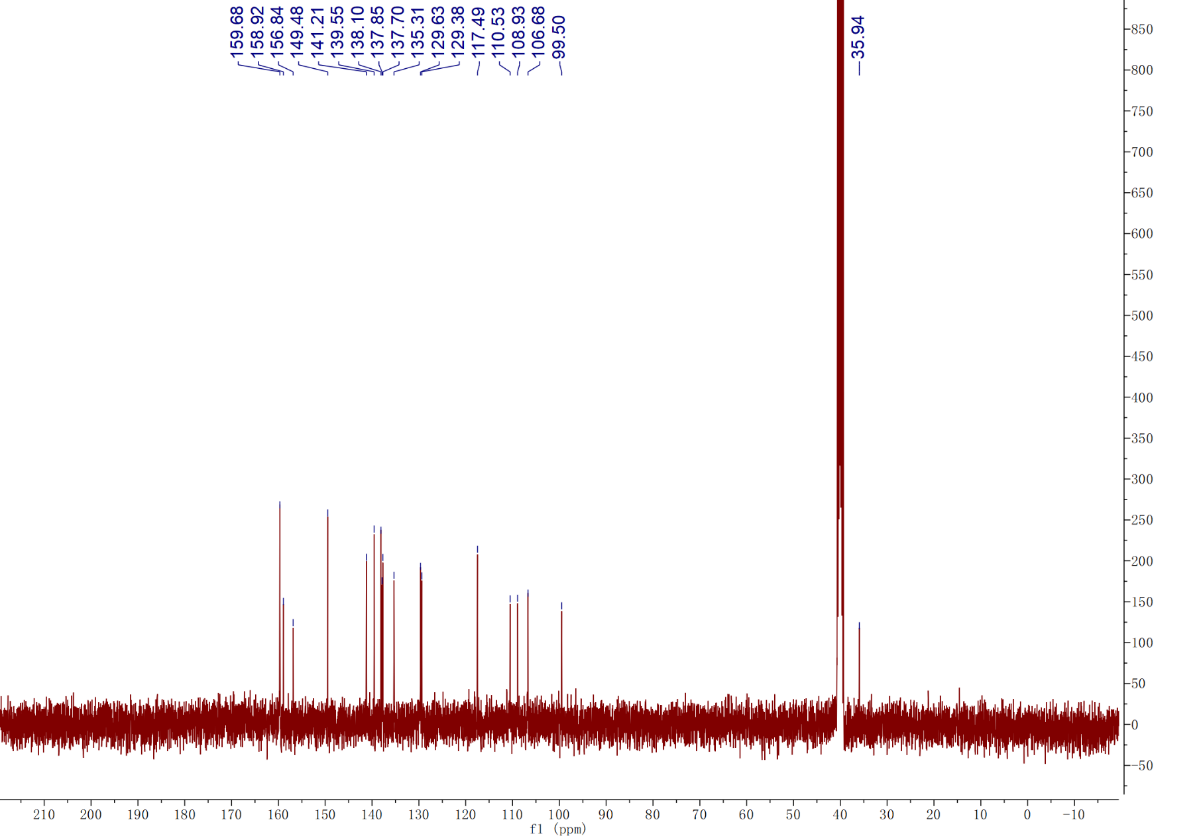


The MS data of compound **6p**


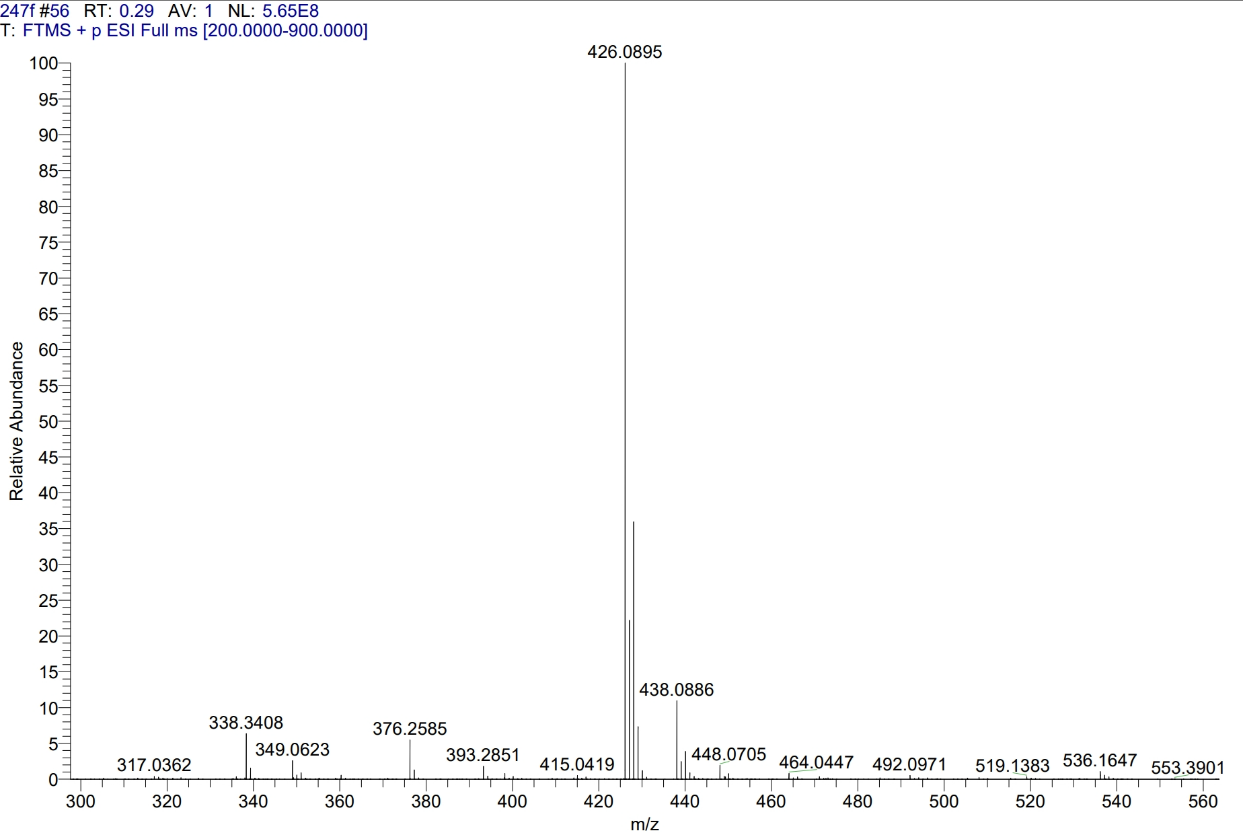


The HPLC spectrum of compound **6p**


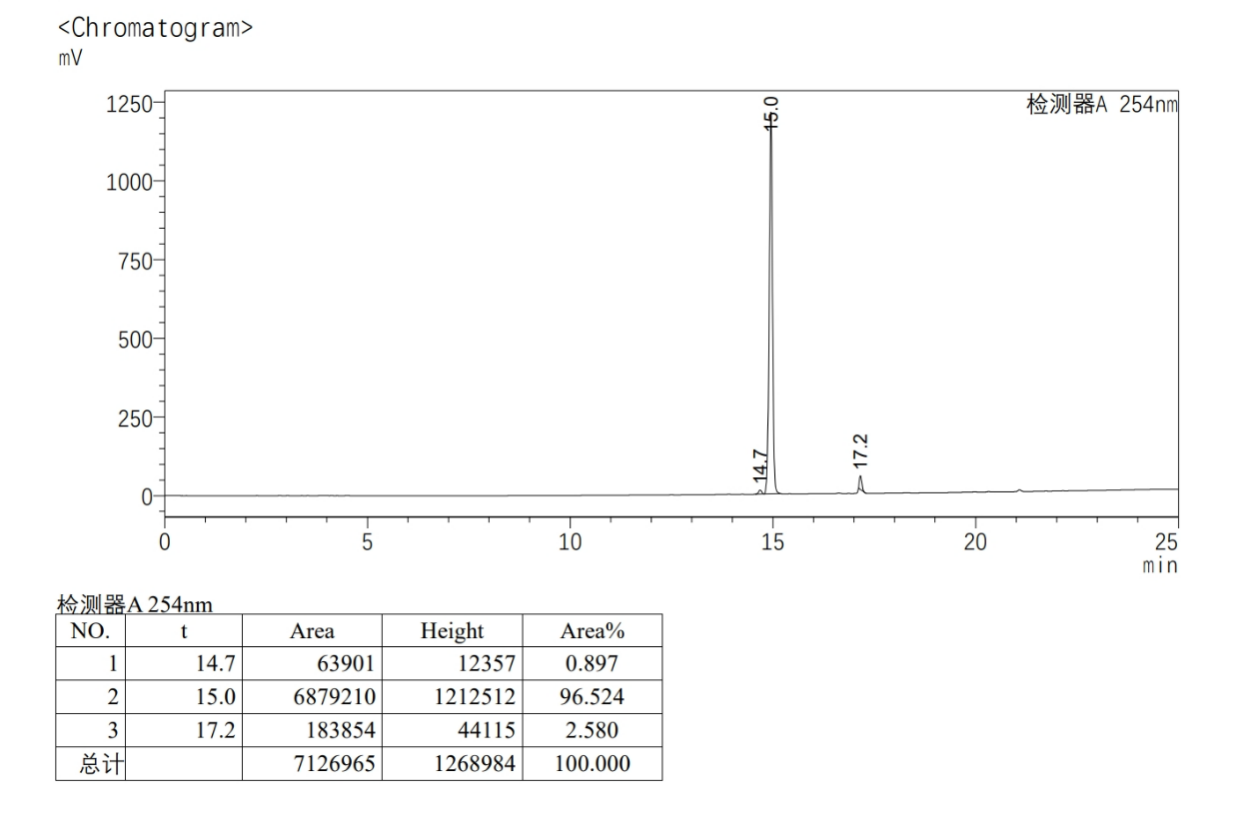


The ^1^H-NMR spectrum of compound **6q**


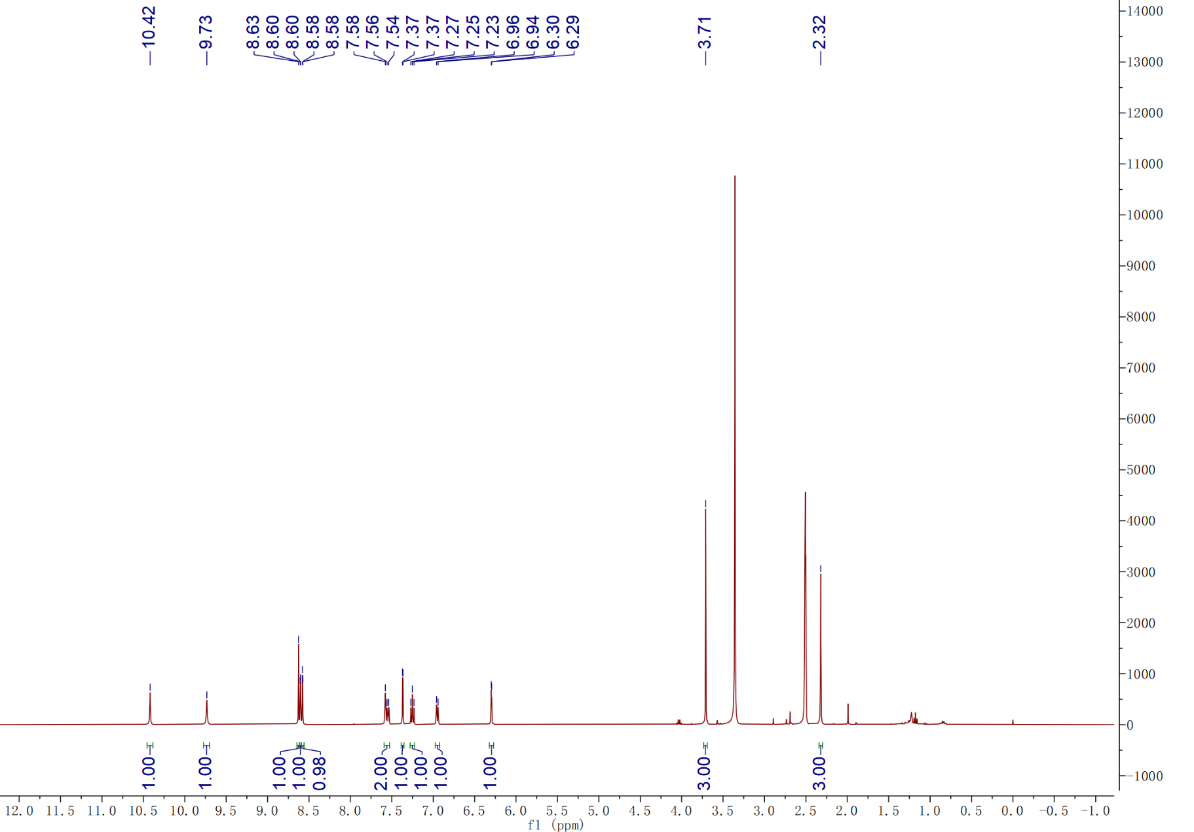


The ^13^C-NMR spectrum of compound **6q**


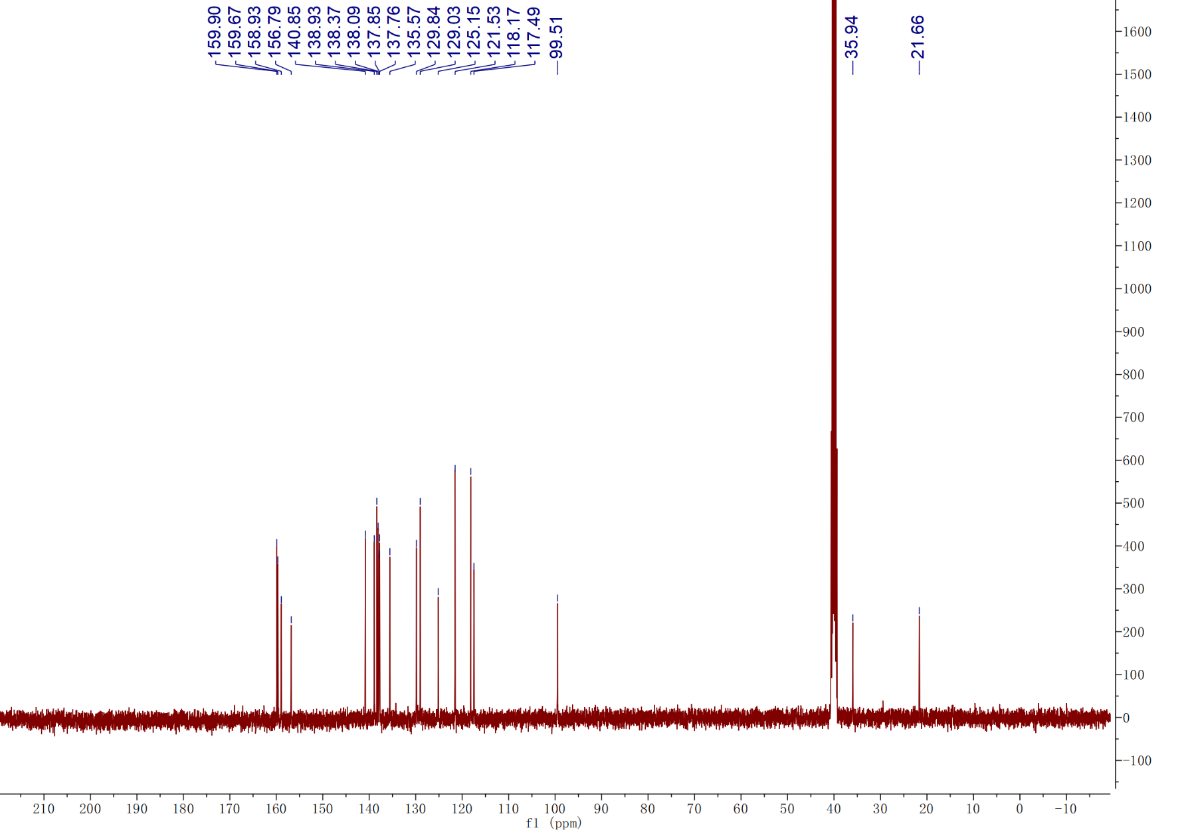


The MS data of compound **6q**


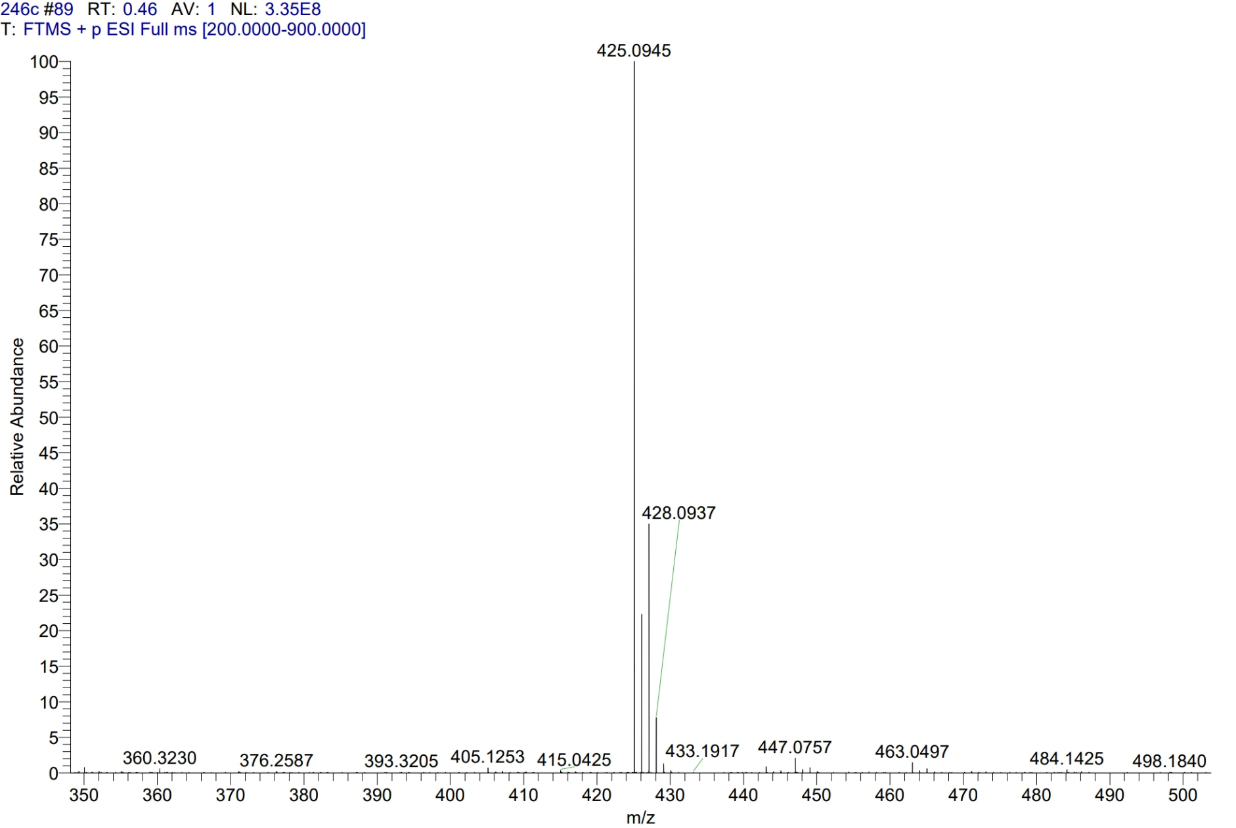


The HPLC spectrum of compound **6q**


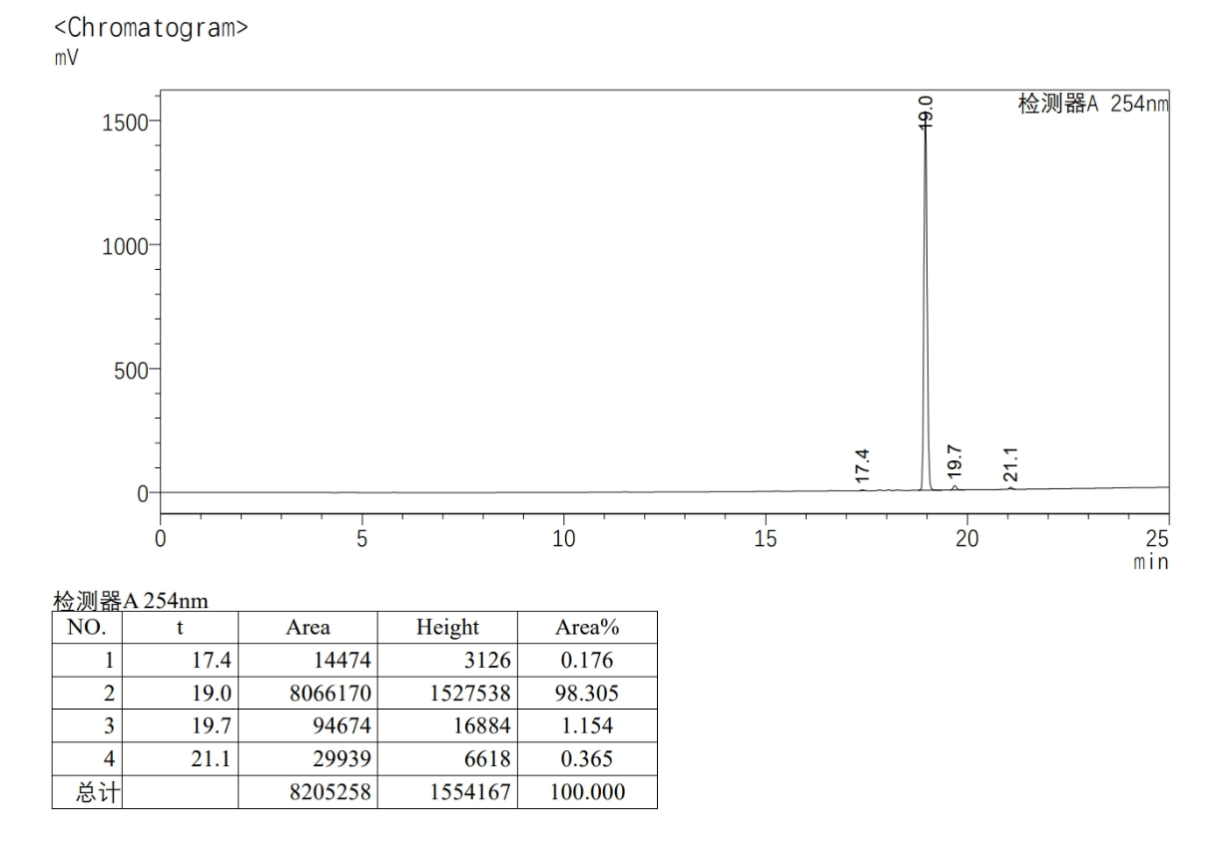


The ^1^H-NMR spectrum of compound **6r**


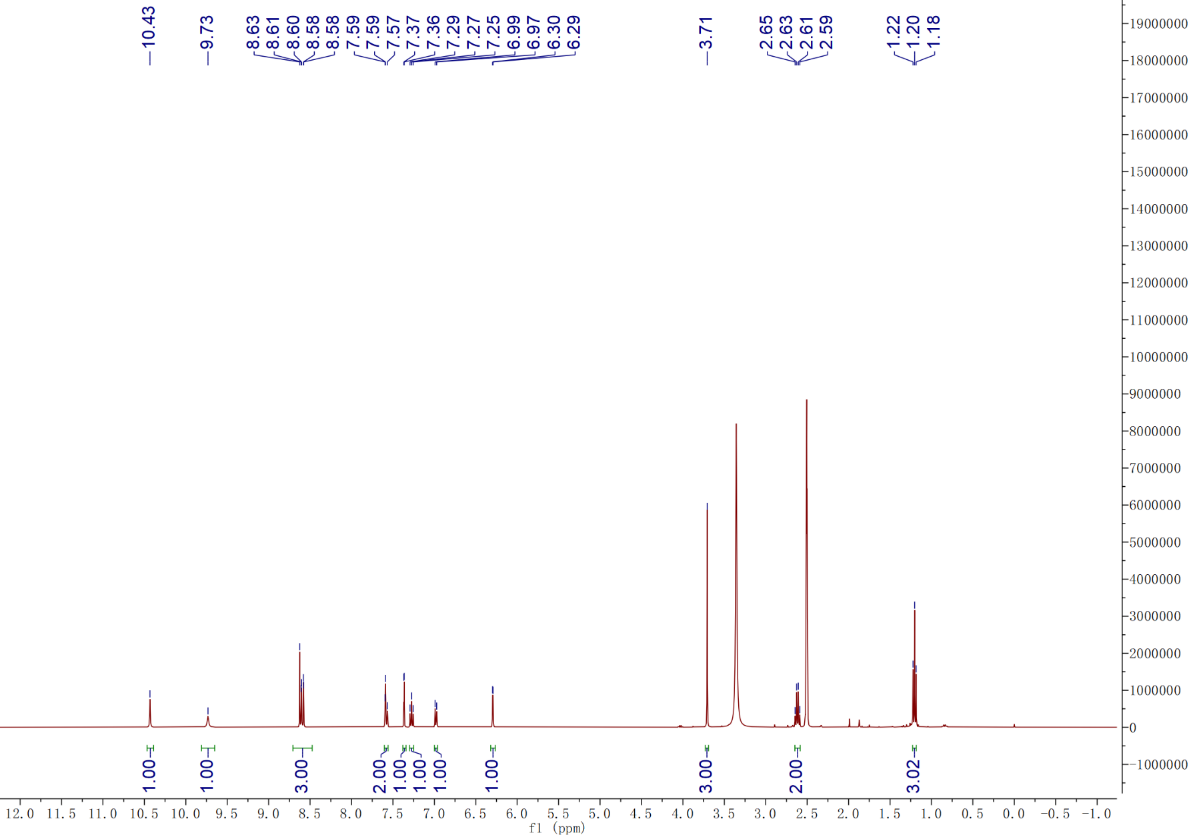


The ^13^C-NMR spectrum of compound **6r**


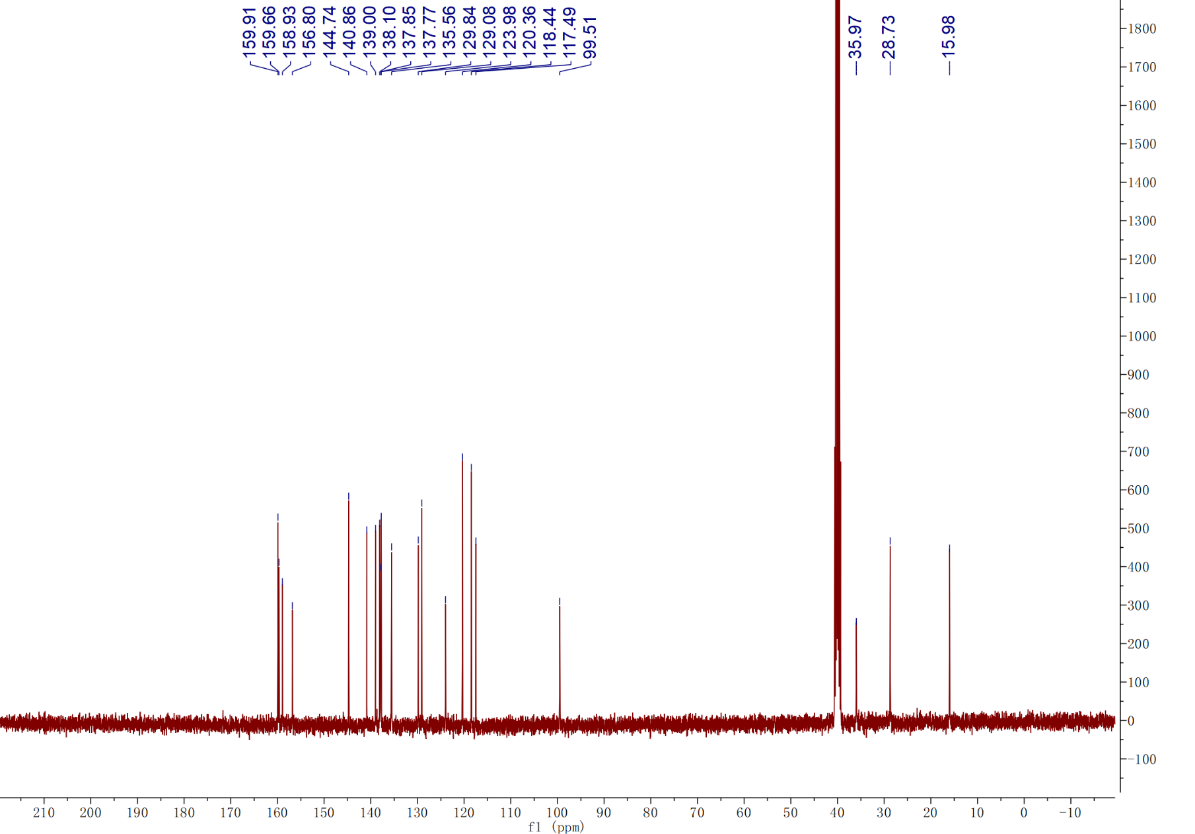


The MS data of compound **6r**


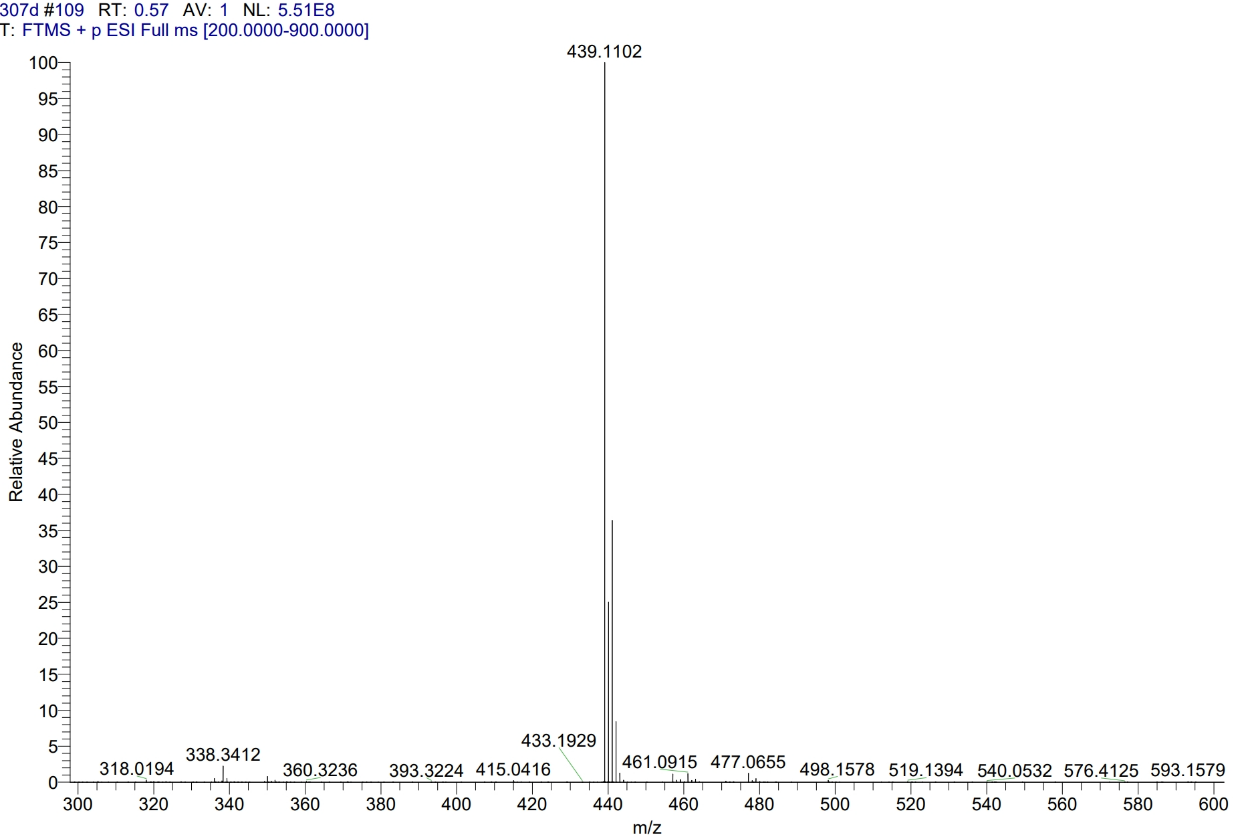


The HPLC spectrum of compound **6r**


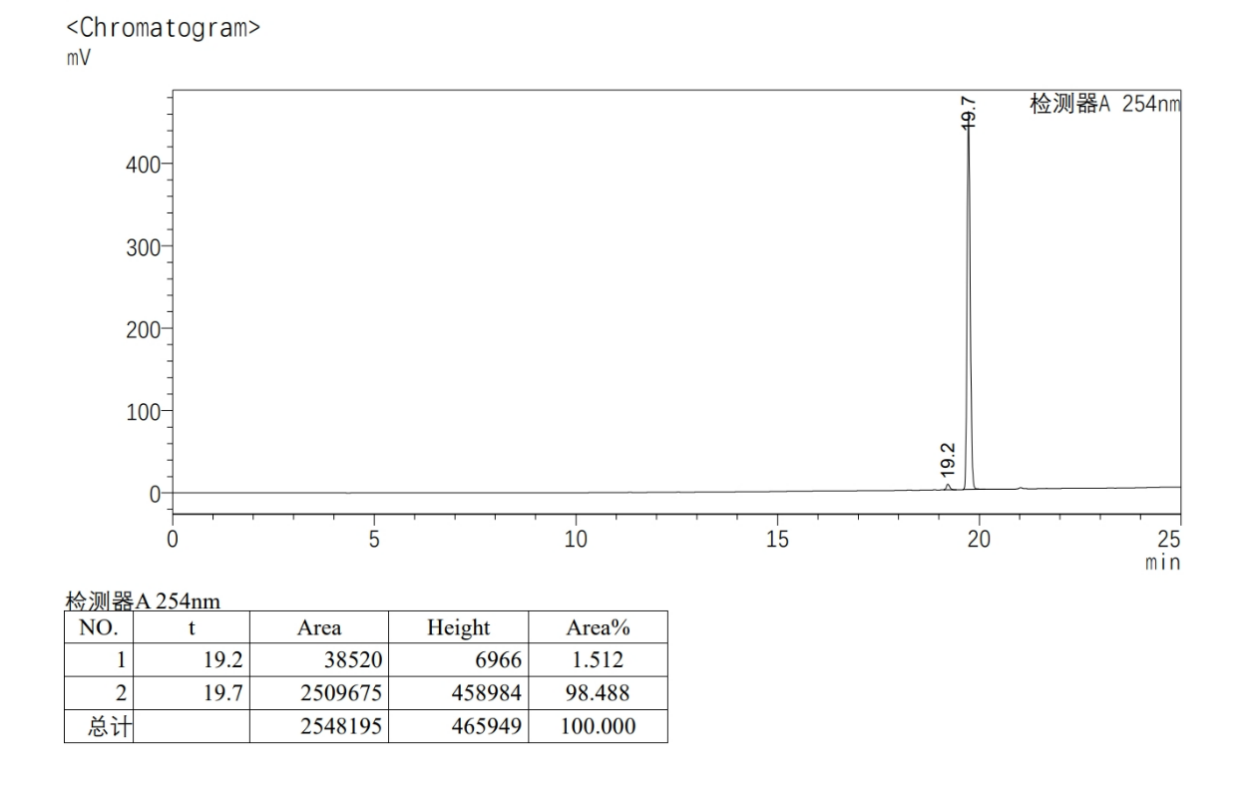


The ^1^H-NMR spectrum of compound **6s**


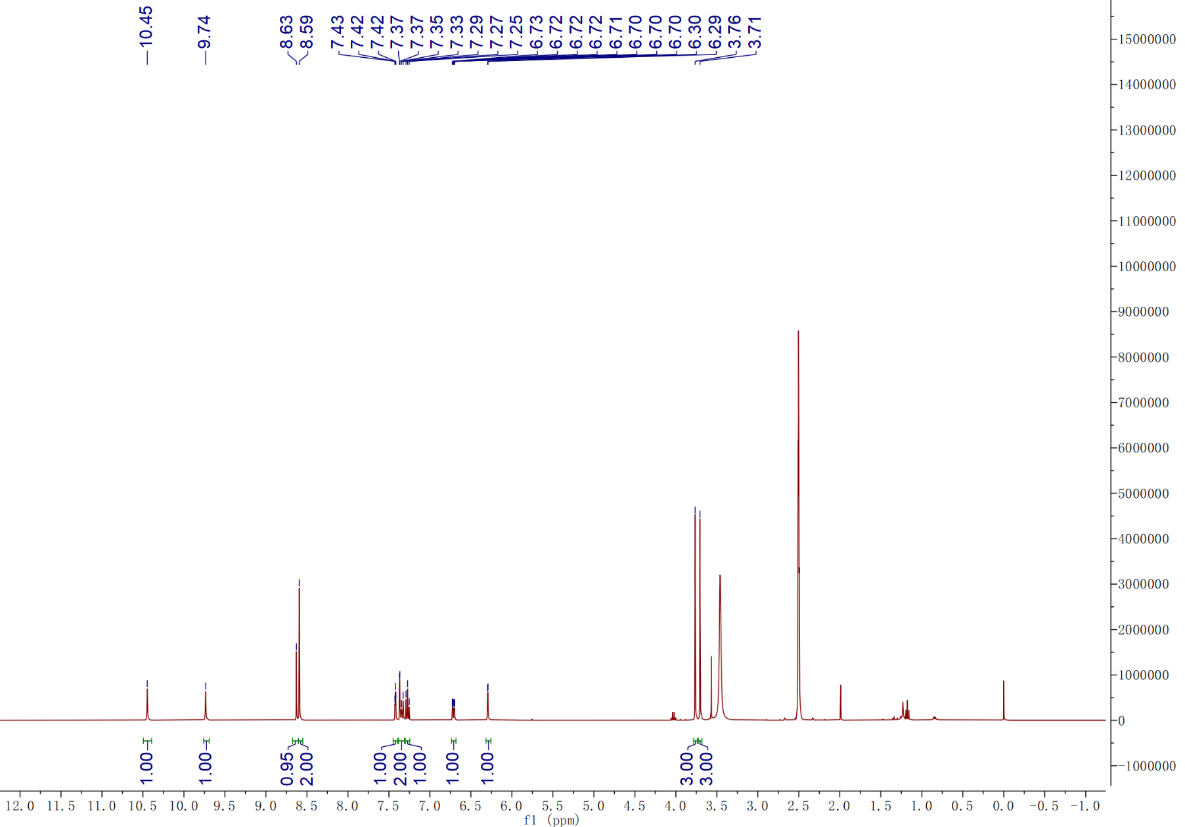


The ^13^C-NMR spectrum of compound **6s**


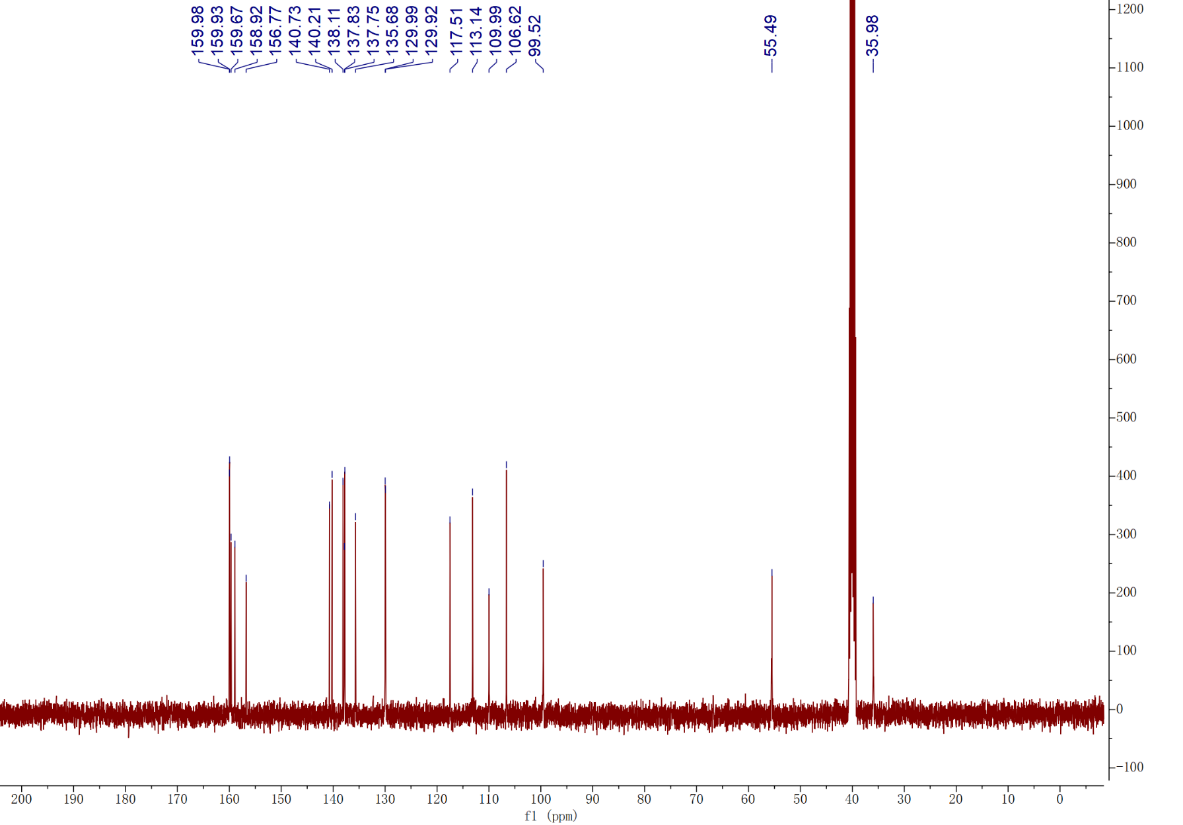


The MS data of compound **6s**


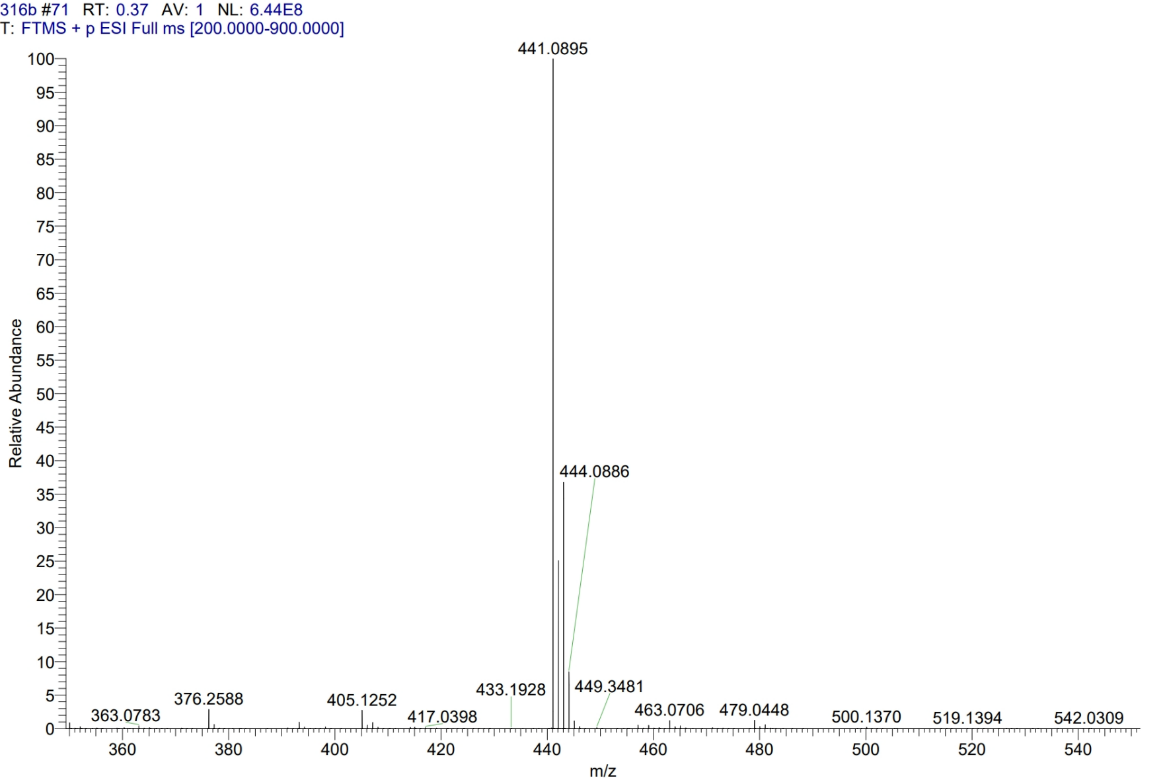


The HPLC spectrum of compound **6s**


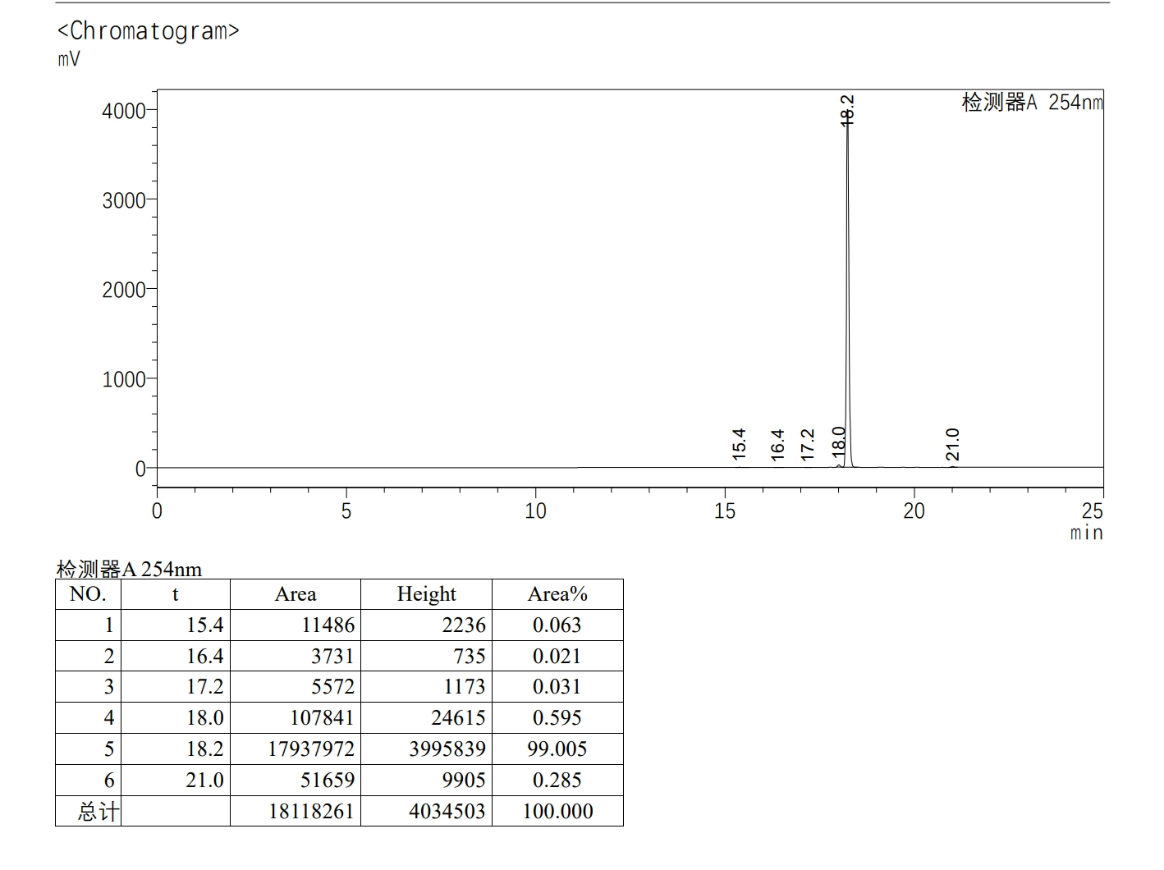


The ^1^H-NMR spectrum of compound **6t**


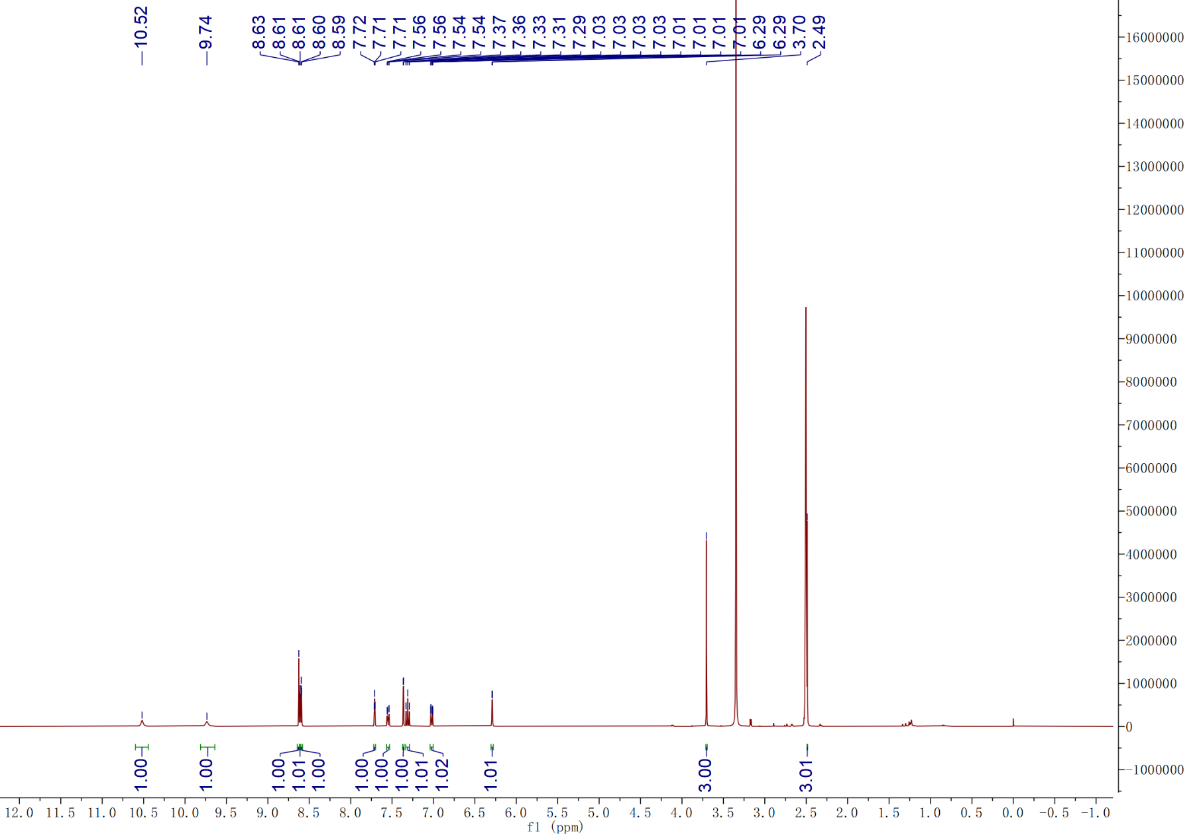


The ^13^C-NMR spectrum of compound **6t**


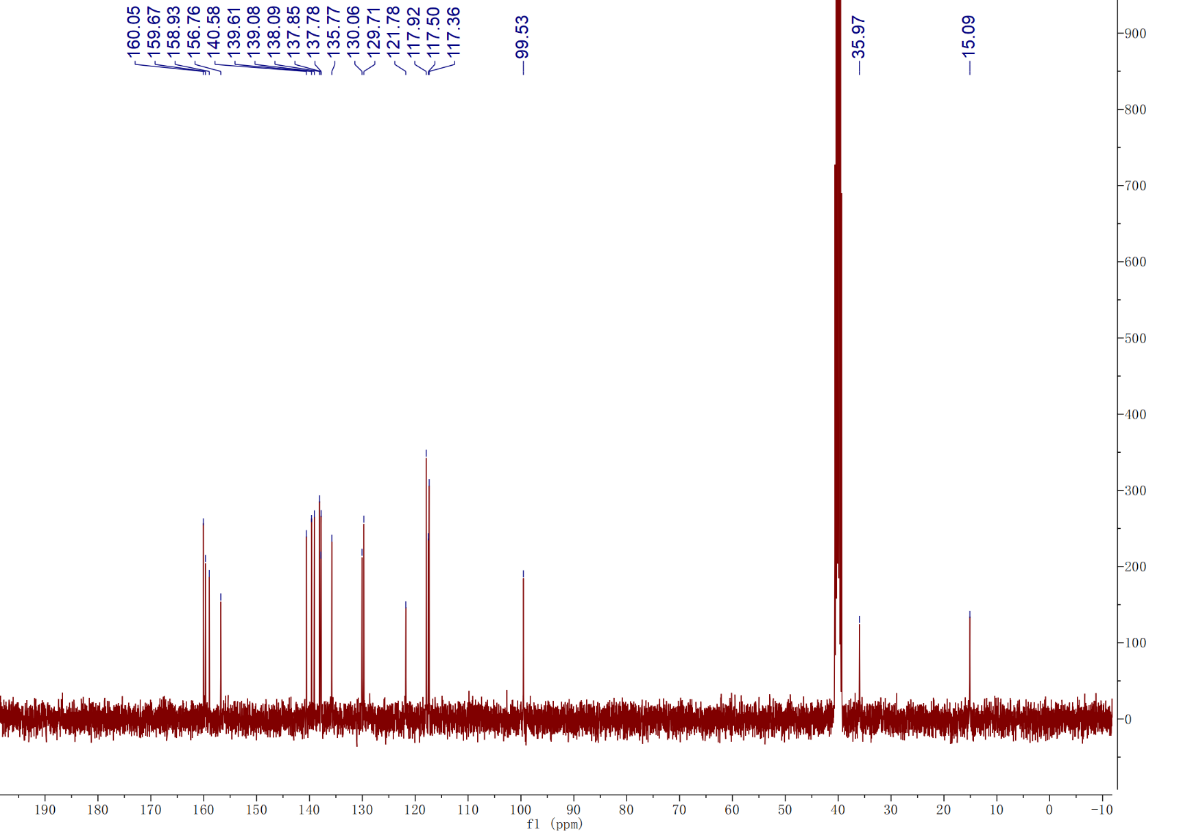


The MS data of compound **6t**


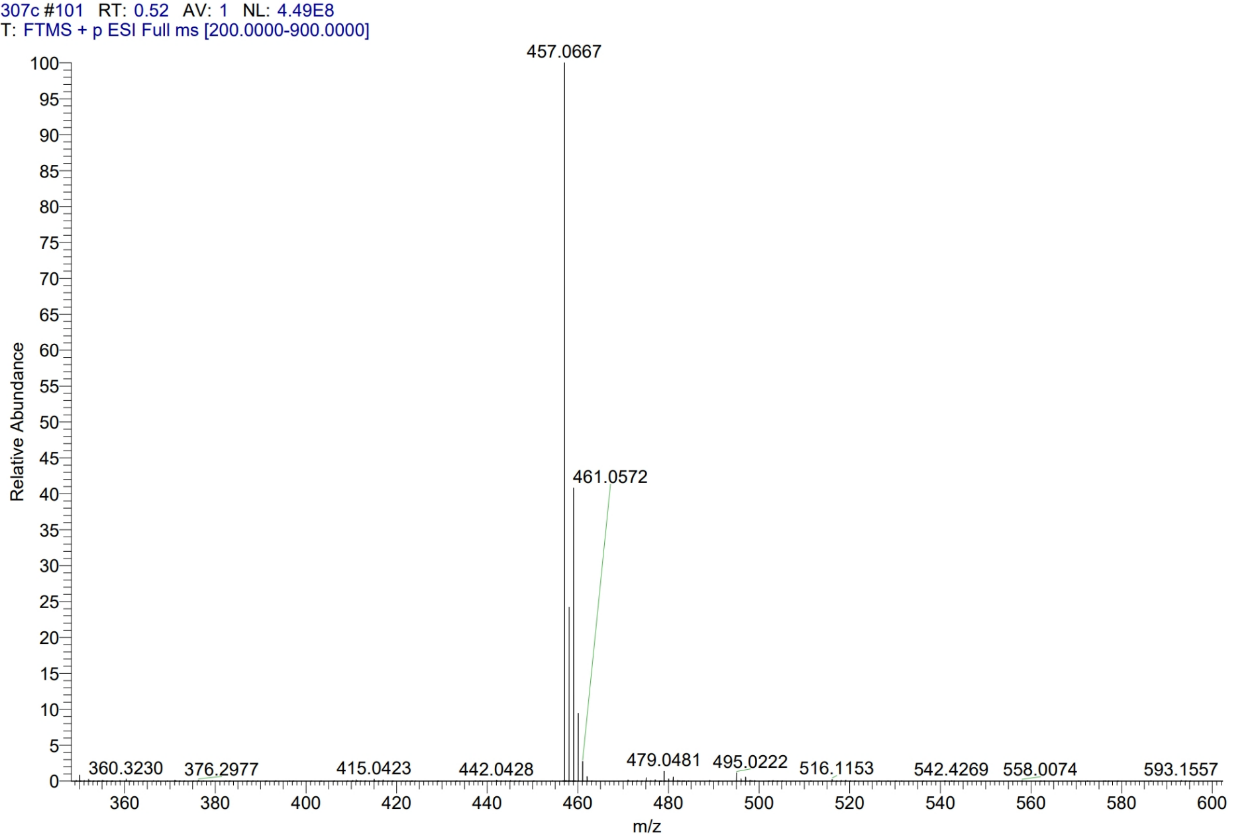


The HPLC spectrum of compound **6t**


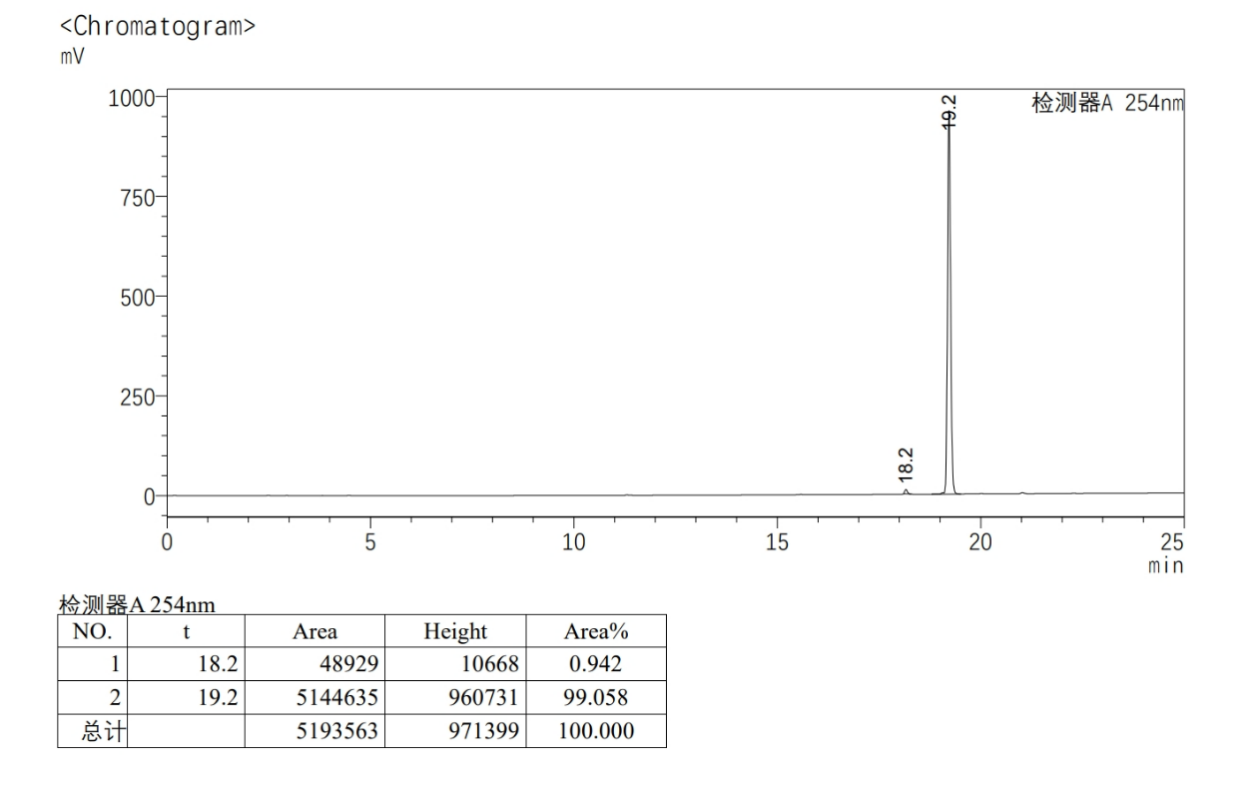


The ^1^H-NMR spectrum of compound **6u**


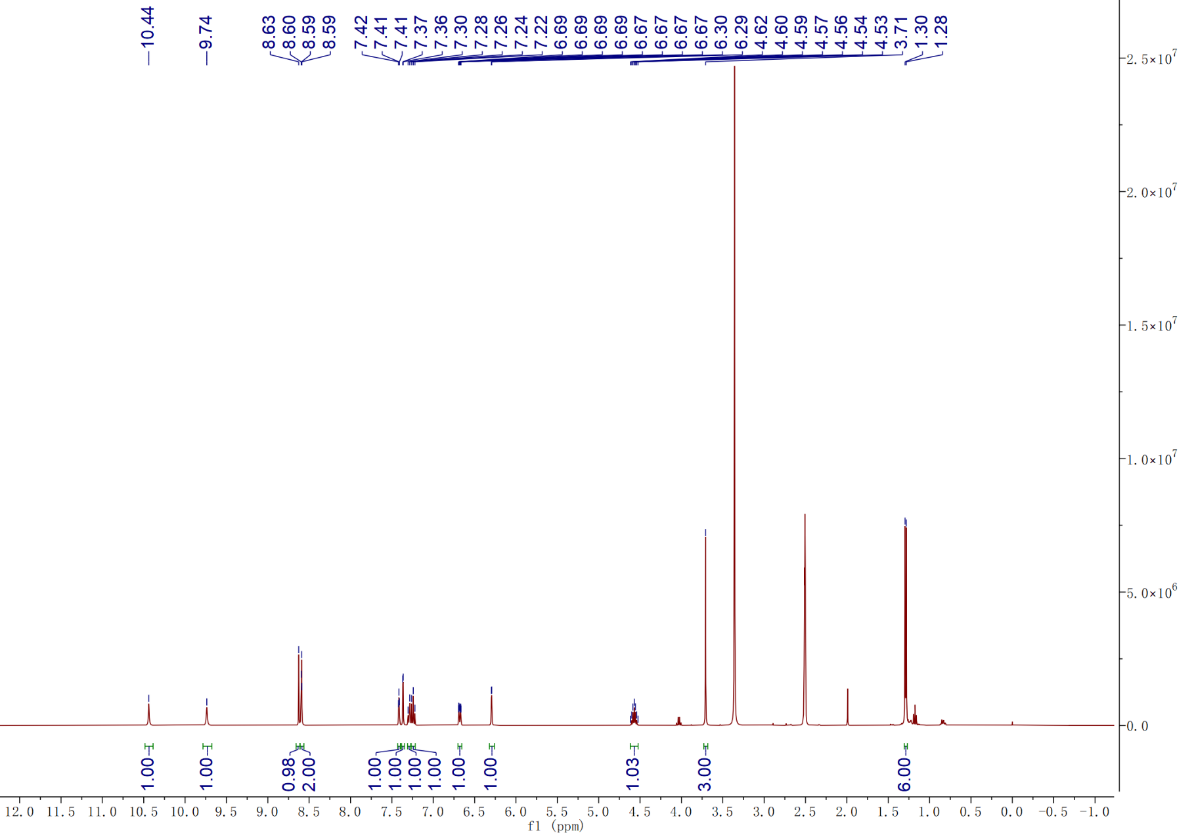


The ^13^C-NMR spectrum of compound **6u**


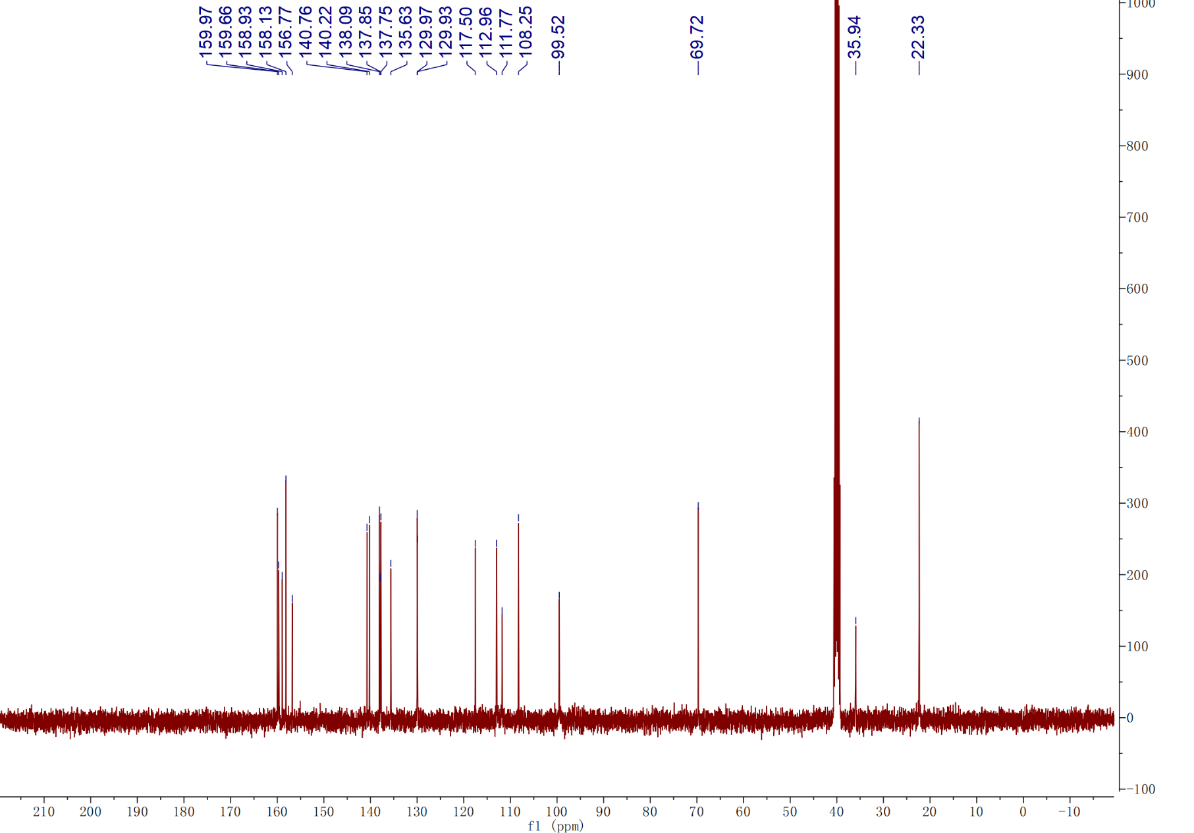


The MS data of compound **6u**


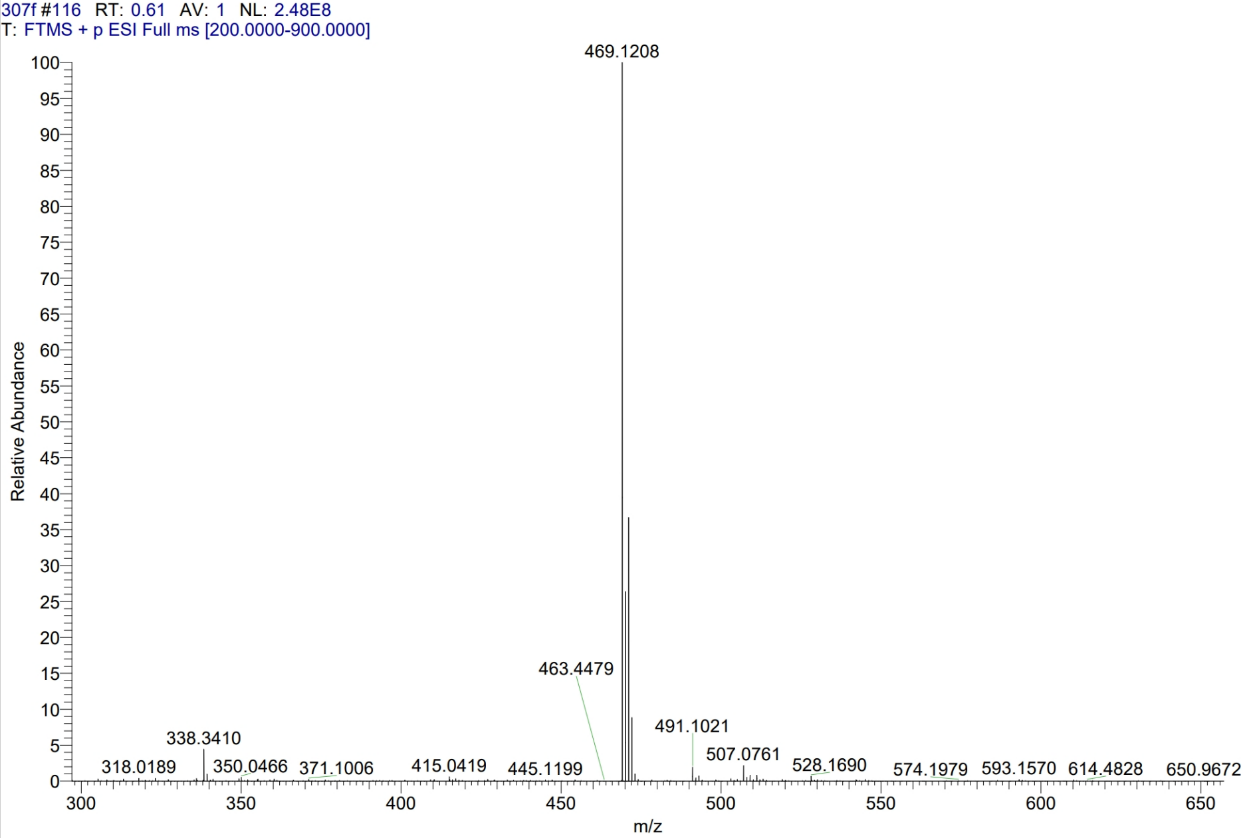


The HPLC spectrum of compound **6u**


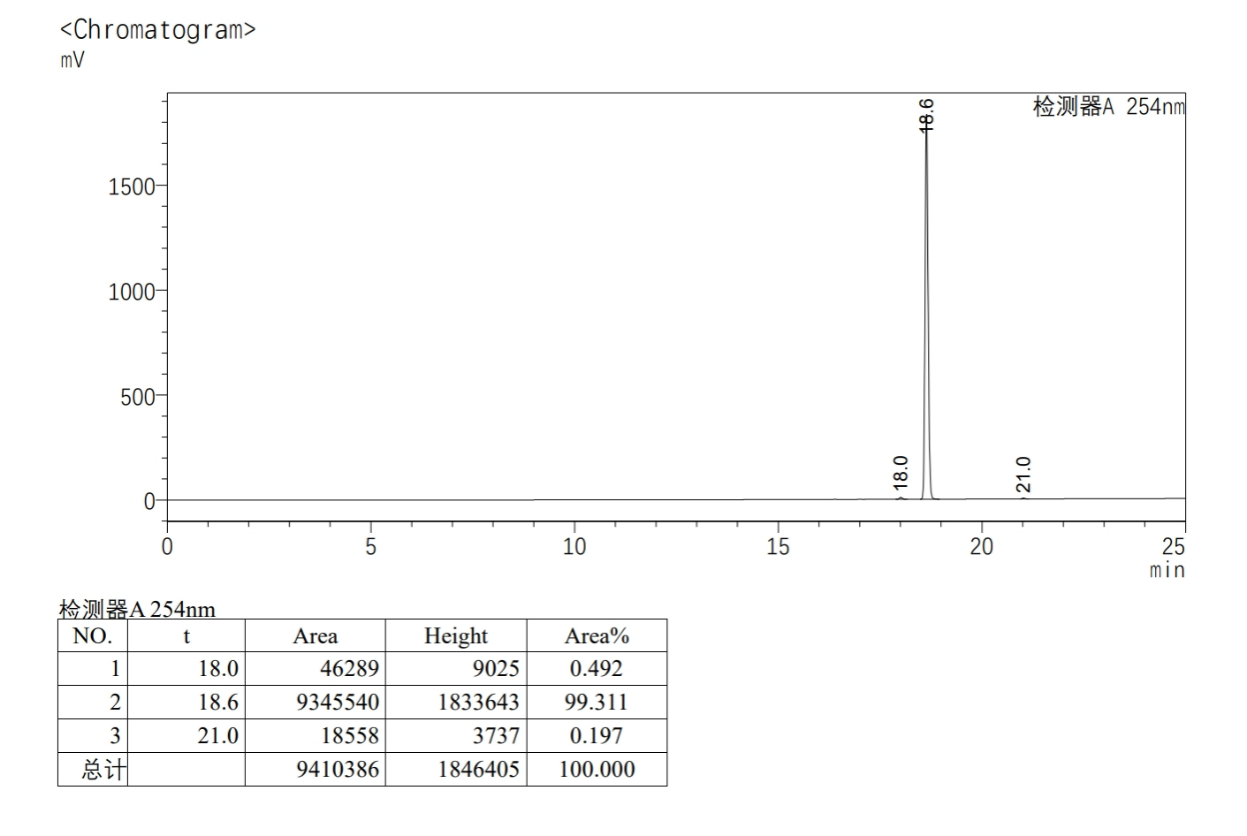


The ^1^H-NMR spectrum of compound **6v**


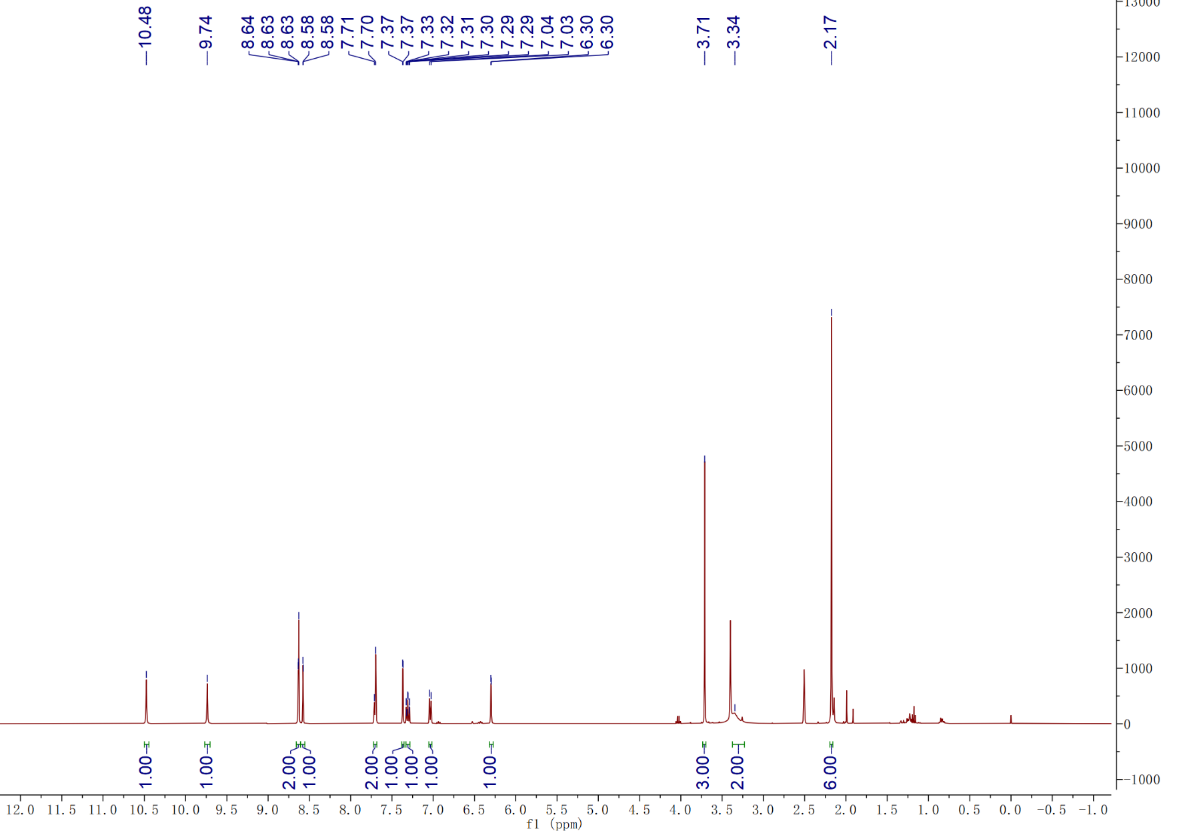


The ^13^C-NMR spectrum of compound **6v**


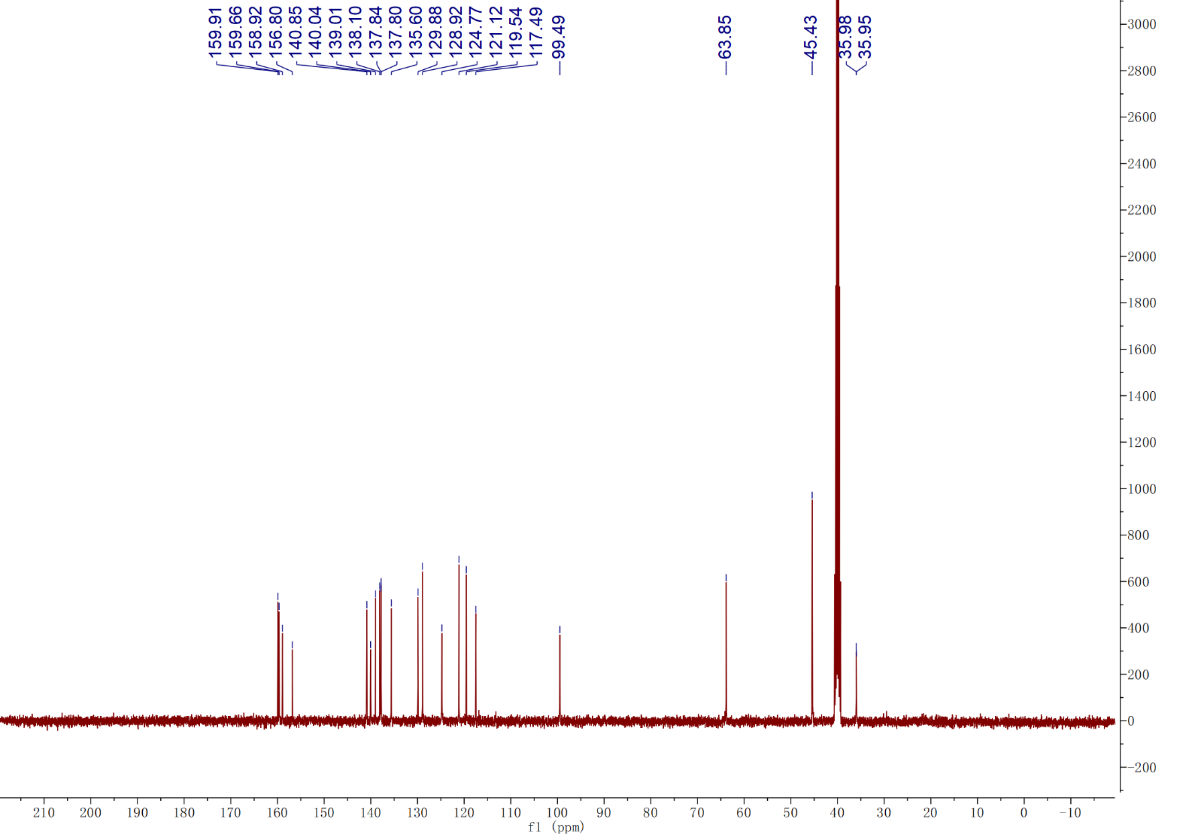


The MS data of compound **6v**


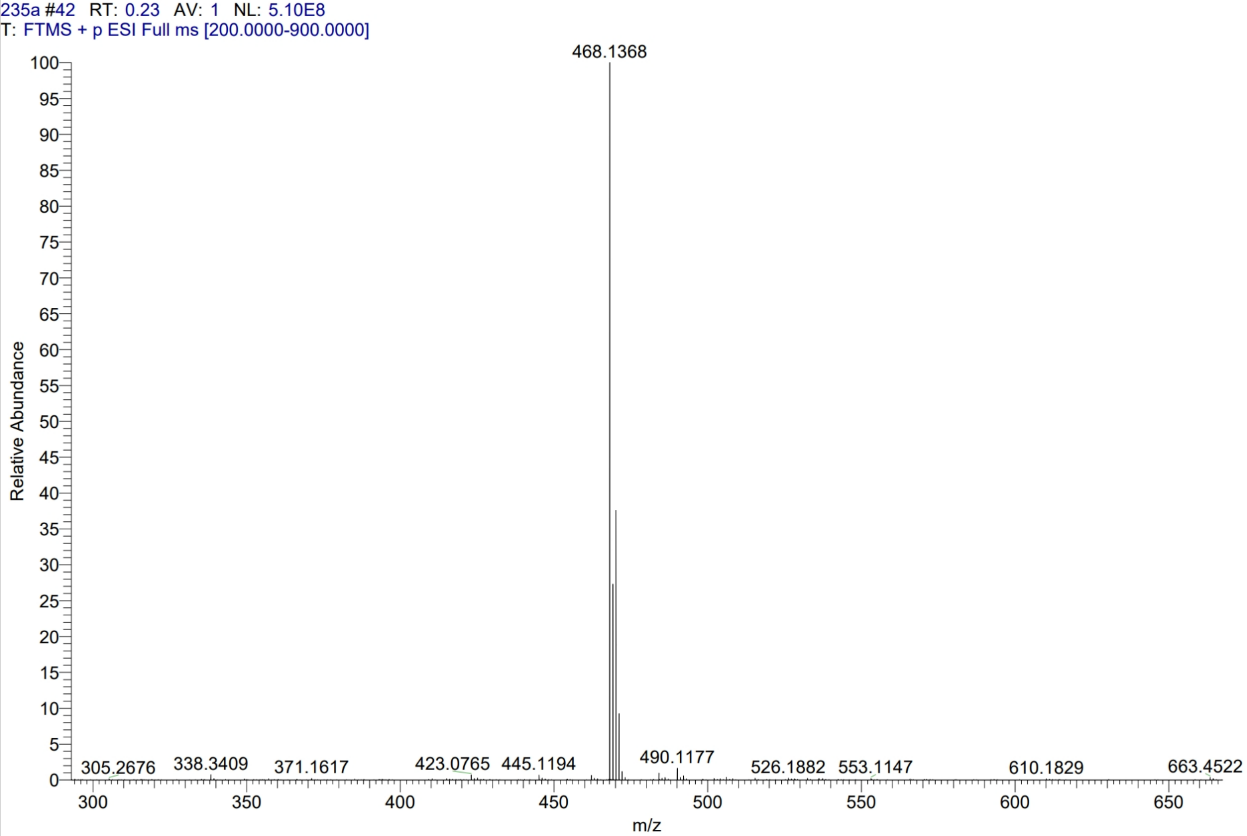


The HPLC spectrum of compound **6v**


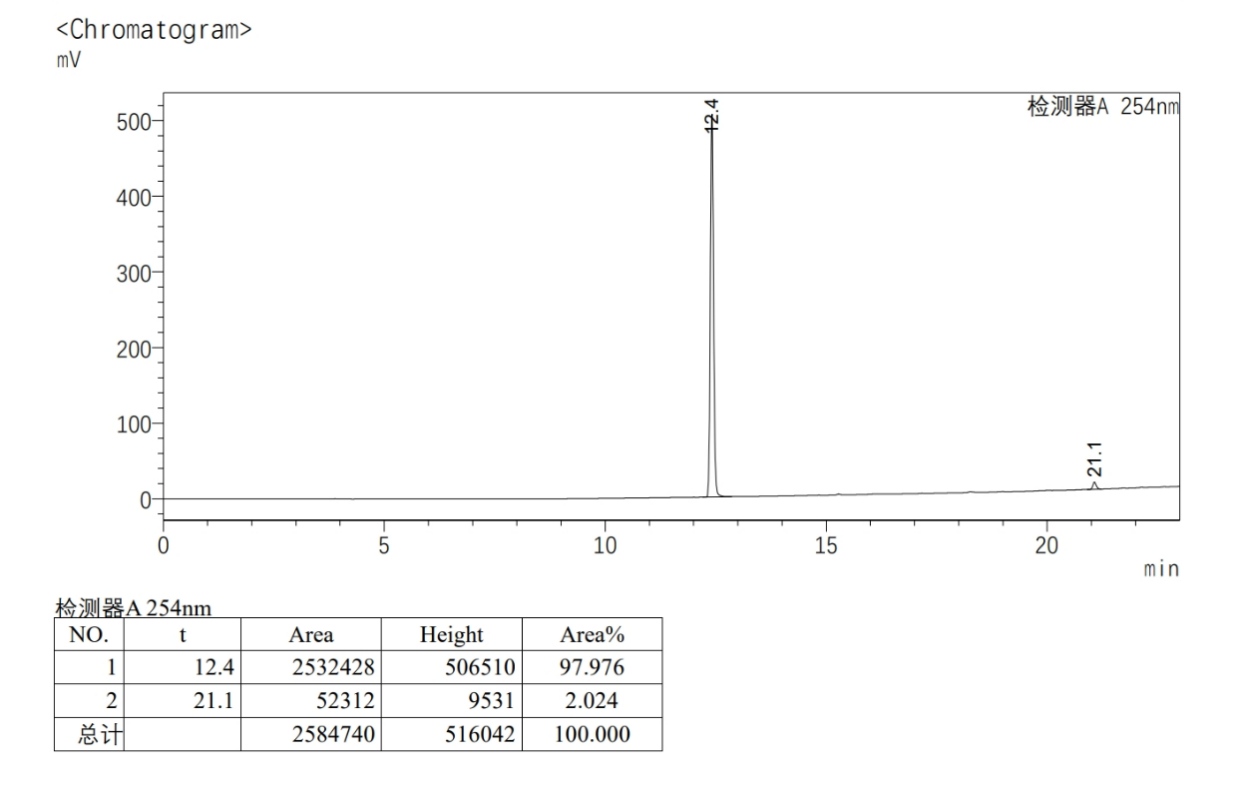


The ^1^H-NMR spectrum of compound **6w**


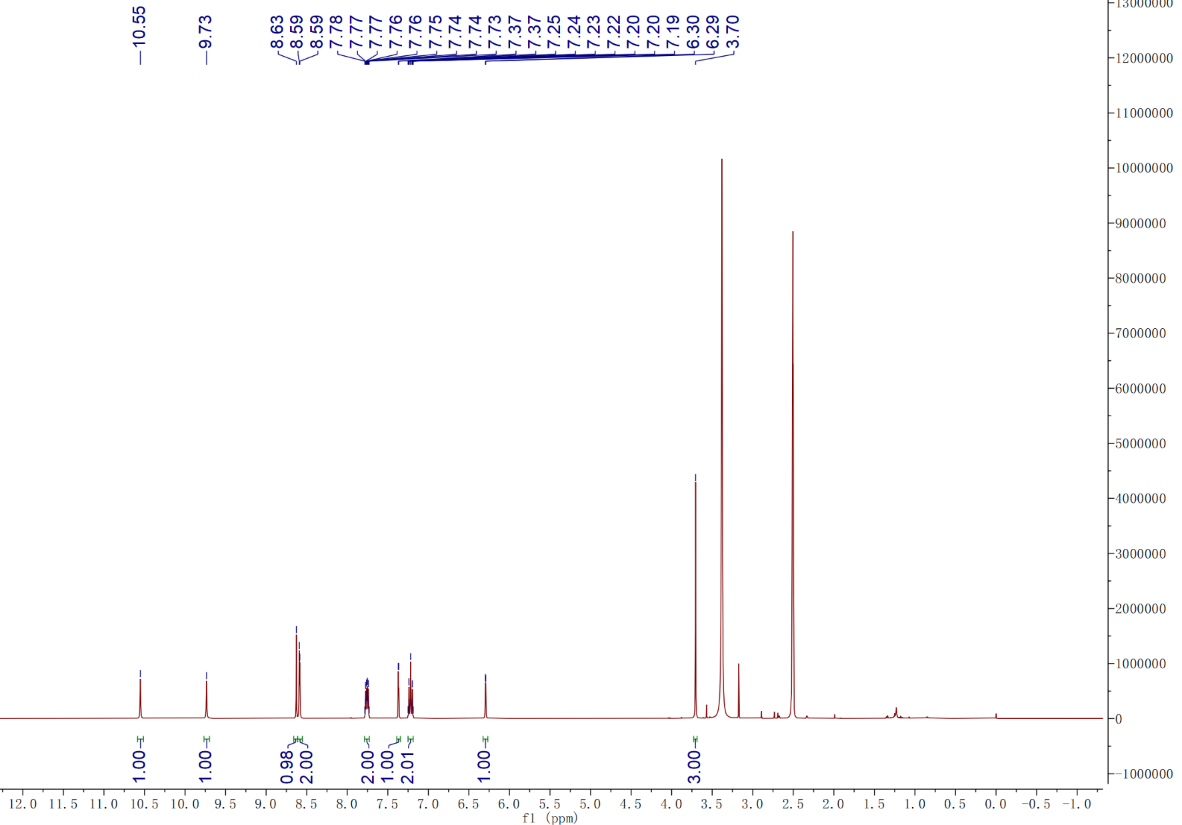


The ^13^C-NMR spectrum of compound **6w**


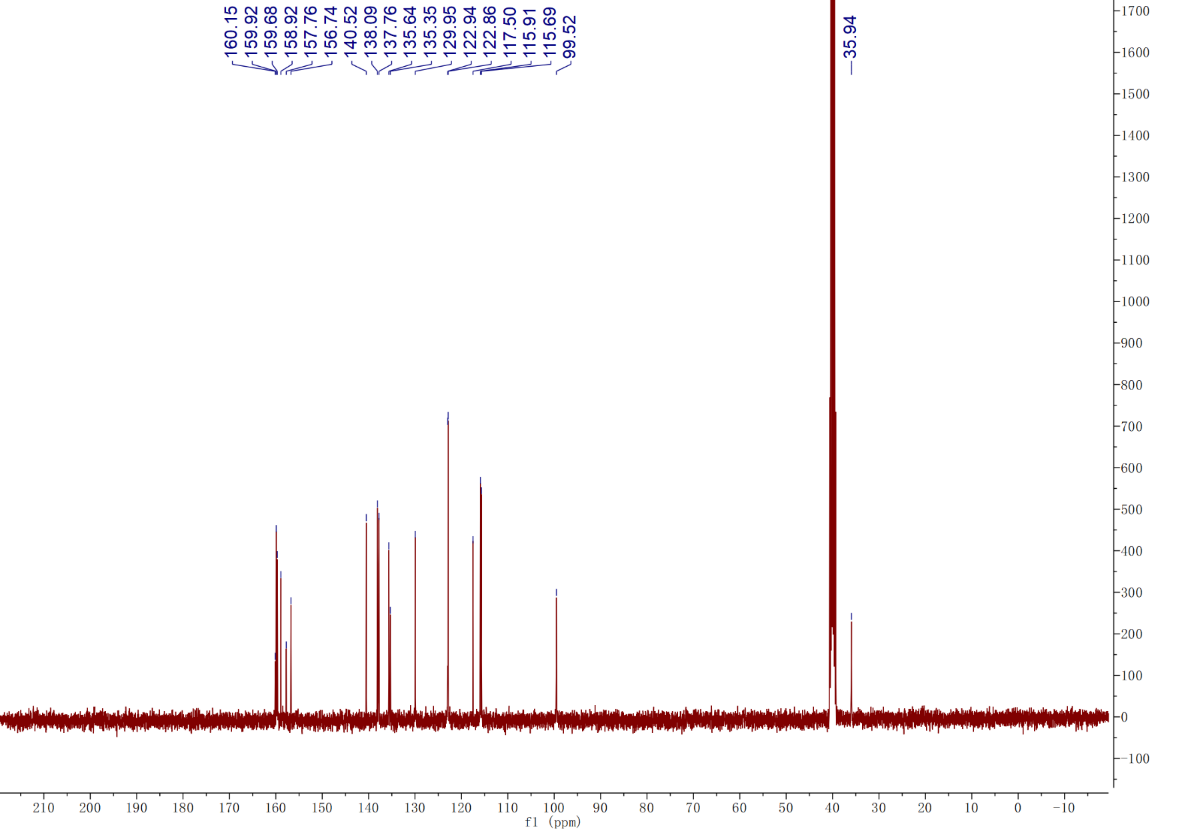


The MS data of compound **6w**

The HPLC spectrum of compound **6w**

The ^1^H-NMR spectrum of compound **6x**

The ^13^C-NMR spectrum of compound **6x**

The MS data of compound **6x**

The HPLC spectrum of compound **6x**

The ^1^H-NMR spectrum of compound **6y**

The ^13^C-NMR spectrum of compound **6y**

The MS data of compound **6y**

The HPLC spectrum of compound **6y**

The ^1^H-NMR spectrum of compound **6z**

The ^13^C-NMR spectrum of compound **6z**

The MS data of compound **6z**

The HPLC spectrum of compound **6z**

The ^1^H-NMR spectrum of compound **6aa**

The ^13^C-NMR spectrum of compound **6aa**

The MS data of compound **6aa**

The HPLC spectrum of compound **6aa**

The ^1^H-NMR spectrum of compound **6ab**

The ^13^C-NMR spectrum of compound **6ab**

The MS data of compound **6ab**

The HPLC spectrum of compound **6ab**

The ^1^H-NMR spectrum of compound **6ac**

The ^13^C-NMR spectrum of compound **6ac**

The MS data of compound **6ac**

The HPLC spectrum of compound **6ac**

The ^1^H-NMR spectrum of compound **6ad**

The ^13^C-NMR spectrum of compound **6ad**

The MS data of compound **6ad**

The HPLC spectrum of compound **6ad**

The ^1^H-NMR spectrum of compound **6ae**

The ^13^C-NMR spectrum of compound **6ae**

The MS data of compound **6ae**

The HPLC spectrum of compound **6ae**

The ^1^H-NMR spectrum of compound **6af**

The ^13^C-NMR spectrum of compound **6af**

The MS data of compound **6af**

The HPLC spectrum of compound **6af**

The ^1^H-NMR spectrum of compound **6ag**

The ^13^C-NMR spectrum of compound **6ag**

The MS data of compound **6ag**

The HPLC spectrum of compound **6ag**

The ^1^H-NMR spectrum of compound **9a**

The ^13^C-NMR spectrum of compound **9a**

The MS data of compound **9a**

The HPLC spectrum of compound **9a**

The ^1^H-NMR spectrum of compound **9b**

The ^13^C-NMR spectrum of compound **9b**

The MS data of compound **9b**

The HPLC spectrum of compound **9b**

The ^1^H-NMR spectrum of compound **9c**

The ^13^C-NMR spectrum of compound **9c**

The MS data of compound **9c**

The HPLC spectrum of compound **9c**

The ^1^H-NMR spectrum of compound **9d**

The ^13^C-NMR spectrum of compound **9d**

The MS data of compound **9d**

The HPLC spectrum of compound **9d**

The ^1^H-NMR spectrum of compound **9e**

The ^13^C-NMR spectrum of compound **9e**

The MS data of compound **9e**

The HPLC spectrum of compound **9e**

The ^1^H-NMR spectrum of compound **9f**

The ^13^C-NMR spectrum of compound **9f**

The MS data of compound **9f**

The HPLC spectrum of compound **9f**

The ^1^H-NMR spectrum of compound **9g**

The ^13^C-NMR spectrum of compound **9g**

The MS data of compound **9g**

The HPLC spectrum of compound **9g**

The ^1^H-NMR spectrum of compound **9h**

The ^13^C-NMR spectrum of compound **9h**

The MS data of compound **9h**

The HPLC spectrum of compound **9h**

The ^1^H-NMR spectrum of compound **9i**

The ^13^C-NMR spectrum of compound **9i**

The MS data of compound **9i**

The HPLC spectrum of compound **9i**

The ^1^H-NMR spectrum of compound **15a**

The ^13^C-NMR spectrum of compound **15a**

The MS data of compound **15a**

The HPLC spectrum of compound **15a**

The ^1^H-NMR spectrum of compound **15b**

The ^13^C-NMR spectrum of compound **15b**

The MS data of compound **15b**

The HPLC spectrum of compound **15b**

The ^1^H-NMR spectrum of compound **15c**

The ^13^C-NMR spectrum of compound **15c**

The MS data of compound **15c**

The HPLC spectrum of compound **15c**

The ^1^H-NMR spectrum of compound **15d**

The ^13^C-NMR spectrum of compound **15d**

The MS data of compound **15d**

The HPLC spectrum of compound **15d**

The ^1^H-NMR spectrum of compound **15e**

The ^13^C-NMR spectrum of compound **15e**

The MS data of compound **15e**

The HPLC spectrum of compound **15e**

The ^1^H-NMR spectrum of compound **18a**

The ^13^C-NMR spectrum of compound **18a**

The MS data of compound **18a**

The HPLC spectrum of compound **18a**

The ^1^H-NMR spectrum of compound **18b**

The ^13^C-NMR spectrum of compound **18b**

The MS data of compound **18b**

The HPLC spectrum of compound **18b**

The ^1^H-NMR spectrum of compound **18c**

The ^13^C-NMR spectrum of compound **18c**

The MS data of compound **18c**

The HPLC spectrum of compound **18c**

The ^1^H-NMR spectrum of compound **18d**

The ^13^C-NMR spectrum of compound **18d**

The MS data of compound **18d**

The HPLC spectrum of compound **18d**

The ^1^H-NMR spectrum of compound **18e**

The ^13^C-NMR spectrum of compound **18e**

The MS data of compound **18e**

The HPLC spectrum of compound **18e**

The ^1^H-NMR spectrum of compound **18f**

The ^13^C-NMR spectrum of compound **18f**

The MS data of compound **18f**

The HPLC spectrum of compound **18f**

The ^1^H-NMR spectrum of compound **18g**

The ^13^C-NMR spectrum of compound **18g**

The MS data of compound **18g**

The HPLC spectrum of compound **18g**

The ^1^H-NMR spectrum of compound **18h**

The ^13^C-NMR spectrum of compound **18h**

The MS data of compound **18h**

The HPLC spectrum of compound **18h**

The ^1^H-NMR spectrum of compound **18i**

The ^13^C-NMR spectrum of compound **18i**

The MS data of compound **18i**

The HPLC spectrum of compound **18i**

The ^1^H-NMR spectrum of compound **18j**

The ^13^C-NMR spectrum of compound **18j**

The MS data of compound **18j**

The HPLC spectrum of compound **18j**

The ^1^H-NMR spectrum of compound **18k**

The ^13^C-NMR spectrum of compound **18k**

The MS data of compound **18k**

The HPLC spectrum of compound **18k**

The ^1^H-NMR spectrum of compound **18l**

The ^13^C-NMR spectrum of compound **18l**

The MS data of compound **18l**

The HPLC spectrum of compound **18l**

The ^1^H-NMR spectrum of compound **18m**

The ^13^C-NMR spectrum of compound **18m**

The MS data of compound **18m**

The HPLC spectrum of compound **18m**

The ^1^H-NMR spectrum of compound **18n**

The MS data of compound **18n**

The HPLC spectrum of compound **18n**

The ^1^H-NMR spectrum of compound **18o/SKLB-D18**

The ^13^C-NMR spectrum of compound **18o/SKLB-D18**

The MS data of compound **18o/SKLB-D18**

The HPLC spectrum of compound **18o/SKLB-D18**

The ^1^H-NMR spectrum of compound **18p**

The ^13^C-NMR spectrum of compound **18p**

The MS data of compound **18p**

The HPLC spectrum of compound **18p**

**The** **kinase selectivity analysis of SKLB-D18 at 1 μM**

| **Kinases** | **Inhibition%** | **Kinases** | **Inhibition%** |
| --- | --- | --- | --- |
| MAPK3(h) | 96 | Aurora-B(h) | 66 |
| MAPK1(h) | 94 | CDKL4(h) | 66 |
| MAPK7(h) | 93 | DCAMKL3(h) | 66 |
| STK16(h) | 85 | JNK3(h) | 66 |
| TYK2(h) | 83 | KDR(h) | 66 |
| Ret(h) | 81 | NDR1(h) | 66 |
| GAK(h) | 78 | NEK4(h) | 66 |
| PRK1(h) | 73 | PKD2(h) | 66 |
| ARK5(h) | 72 | Rsk3(h) | 66 |
| TRB2(h) | 71 | CK2α2(h) | 65 |
| FGFR1(h) | 70 | c-RAF(h) | 65 |
| cKit(h) | 69 | LRRK2(h) | 65 |
| GCN2(h) | 69 | PAR-1Bα(h) | 65 |
| Mer(h) | 69 | Yes(h) | 65 |
| MLK3(h) | 69 | HIPK3(h) | 64 |
| CK2(h) | 68 | IKKε(h) | 64 |
| CLK4(h) | 68 | MARK1(h) | 64 |
| GSK3β(h) | 68 | MARK4(h) | 64 |
| HIPK1(h) | 68 | PKCμ(h) | 64 |
| HPK1(h) | 68 | PRK2(h) | 64 |
| Lck(h) activated | 68 | TAK1(h) | 64 |
| MARK3(h) | 68 | TAO2(h) | 64 |
| MYLK2(h) | 68 | TBK1(h) | 64 |
| NEK5(h) | 68 | ZIPK(h) | 64 |
| DAPK1(h) | 67 | A-Raf(h) | 63 |
| DYRK1B(h) | 67 | Flt4(h) | 63 |
| HIPK2(h) | 67 | Lck(h) | 63 |
| HIPK4(h) | 67 | NEK3(h) | 63 |
| JAK2(h) | 67 | PhKγ1(h) | 63 |
| JAK3(h) | 67 | Rsk4(h) | 63 |
| NUAK2(h) | 67 | STK33(h) | 63 |
| SIK2(h) | 67 | MELK(h) | 62 |
| ULK2(h) | 67 | Mnk1(h) | 62 |
| Aurora-C(h) | 66 | TAO1(h) | 62 |
| CDK16/cyclinY(h) | 66 | AMPKα1(h) |  |
| AMPKα1(h) | 61 | Flt3(h) | 55 |
| Met(h) | 61 | PKD3(h) | 55 |
| MLK1(h) | 61 | SBK1(h) | 55 |
| RIPK2(h) | 61 | CDKL3(h) | 54 |
| AMPKα2(h) | 60 | DAPK2(h) | 53 |
| CDK4/cyclinD3(h) | 60 | Fes(h) | 53 |
| Fyn(h) | 60 | IKKβ(h) | 53 |
| IRAK1(h) | 60 | MEKK2(h) | 53 |
| Lyn(h) | 60 | SAPK4(h) | 53 |
| MAP4K3(h) | 60 | TAF1L(h) | 53 |
| TSSK1(h) | 60 | TSSK3(h) | 53 |
| B-Raf(h) | 59 | BIKe(h) | 52 |
| JAK1(h) | 59 | TLK2(h) | 52 |
| MEK2(h) | 59 | Aurora-A(h) | 51 |
| MLK2(h) | 59 | MAP4K5(h) | 51 |
| Mnk2(h) | 59 | Rsk1(h) | 51 |
| CDK14/cyclinY(h) | 58 | CaMKIIγ(h) | 50 |
| GSK3α(h) | 58 | NIM1(h) | 50 |
| AAK1(h) | 58 | ULK1(h) | 49 |
| CDK2/cyclinA(h) | 58 | CK2α1(h) | 49 |
| CLK2(h) | 58 | DYRK3(h) | 49 |
| Ron(h) | 58 | Wee1B(h) | 49 |
| CDK7/cyclinH/MAT1(h) | 57 | TrkA(h) | 48 |
| CDK17/cyclinY(h) | 57 | CDK18/cyclinY(h) | 48 |
| CDK5/p35(h) | 57 | CDK2/cyclinE(h) | 48 |
| GCK(h) | 57 | CDKL1(h) | 48 |
| IRR(h) | 57 | JNK2α2(h) | 48 |
| p70S6K(h) | 57 | NEK1(h) | 48 |
| MAPK2(h) | 56 | TNIK(h) | 48 |
| NEK9(h) | 56 | Wee1(h) | 48 |
| CDK5/p25(h) | 55 | CaMKIδ(h) | 47 |
| cSRC(h) | 55 | CaMKIIδ(h) | 47 |
| DCAMKL2(h) | 55 | CDK6/cyclinD3(h) | 47 |
| FGFR2(h) | 55 | IRAK4(h) | 47 |
| FGFR3(h) | 55 | Bmx(h) | 46 |
| MAK(h) | 46 | TSSK4(h) | 36 |
| Pyk2(h) | 46 | DDR1(h) | 35 |
| TrkB(h) | 46 | PTK5(h) | 35 |
| Abl(h) | 45 | Fms(h) | 34 |
| MST1(h) | 45 | IKKα(h) | 34 |
| PAK1(h) | 45 | MRCKγ(h) | 34 |
| ULK3(h) | 45 | ROCK-II(h) | 34 |
| DRAK1(h) | 44 | TTK(h) | 34 |
| CaMKIIβ(h) | 44 | Pim-3(h) | 33 |
| CDK1/cyclinB(h) | 44 | BRK(h) | 32 |
| Tie2 (h) | 44 | Hck(h) activated | 32 |
| Arg(h) | 43 | MOK(h) | 31 |
| PrKX(h) | 43 | Cdc7/cyclinB1(h) | 30 |
| TrkC(h) | 43 | TGFBR2(h) | 30 |
| Fgr(h) | 42 | DRAK2(h) | 29 |
| NEK2(h) | 42 | LKB1(h) | 29 |
| PDK1(h) | 42 | CDK9/cyclin T1(h) | 28 |
| DYRK2(h) | 41 | OSR1(h) | 28 |
| Rsk2(h) | 41 | PKCβII(h) | 28 |
| JNK1α1(h) | 40 | Snk(h) | 28 |
| MST2(h) | 40 | NDR2(h) | 27 |
| CDKL2(h) | 39 | PAK3(h) | 27 |
| Flt1(h) | 39 | PKCγ(h) | 27 |
| EphA7(h) | 38 | ALK4(h) | 26 |
| HRI(h) | 38 | NLK(h) | 26 |
| LTK(h) | 38 | PAK5(h) | 26 |
| Plk4(h) | 38 | Pim-2(h) | 26 |
| Syk(h) | 38 | PKR(h) | 26 |
| LATS2(h) | 37 | SIK(h) | 26 |
| PDGFRα(h) | 37 | Txk(h) | 26 |
| Pim-1(h) | 37 | SAPK3(h) | 25 |
| STK39(h) | 37 | CaMKIV(h) | 24 |
| Axl(h) | 36 | MLK4(h) | 24 |
| CHK1(h) | 36 | TLK1(h) | 24 |
| TAO3(h) | 36 | WNK2(h) | 24 |
| BrSK2(h) | 23 | CaMKI(h) | 16 |
| EphB1(h) | 23 | CDK3/cyclinE(h) | 16 |
| Itk(h) | 23 | CK1γ1(h) | 16 |
| PKCδ(h) | 23 | CK1δ(h) | 16 |
| MINK(h) | 22 | Fer(h) | 16 |
| SIK3(h) | 22 | GRK4(h) | 16 |
| BMPR2(h) | 21 | MLCK(h) | 16 |
| CaMKK2(h) | 21 | MSK2(h) | 16 |
| LATS1(h) | 21 | PKCη(h) | 16 |
| PAK4(h) | 21 | VRK1(h) | 16 |
| PDGFRβ(h) | 21 | MEK1(h) | 15 |
| PKCα(h) | 21 | MRCKβ(h) | 15 |
| ALK1(h) | 20 | MST4(h) | 15 |
| Hck(h) | 20 | p70S6Kβ(h) | 15 |
| ICK(h) | 20 | ACK1(h) | 14 |
| LIMK1(h) | 20 | CK1γ3(h) | 14 |
| MuSK(h) | 20 | EphA8(h) | 14 |
| TGFBR1(h) | 20 | MAP4K4(h) | 14 |
| CHK2(h) | 19 | PDHK2(h) | 14 |
| CRIK(h) | 19 | PKBγ(h) | 14 |
| MST3(h) | 19 | PKCε(h) | 14 |
| PhKγ2(h) | 19 | DYRK1A(h) | 13 |
| PKA(h) | 19 | ALK(h) | 13 |
| PRP4(h) | 19 | IR(h), activated | 13 |
| CK1ε(h) | 18 | PASK(h) | 13 |
| CK1γ2(h) | 18 | ATR/ATRIP(h) | 13 |
| PRKG2(h) | 18 | CaMKIγ(h) | 12 |
| WNK4(h) | 18 | CaMKK1(h) | 12 |
| CLK1(h) | 17 | CLIK1(h) | 12 |
| DCAMKL1(h) | 17 | EphA3(h) | 12 |
| EphA2(h) | 17 | Haspin(h) | 12 |
| FAK(h) | 17 | PAK2(h) | 12 |
| LIMK2(h) | 17 | PKAcβ(h) | 12 |
| MSK1(h) | 17 | PKBα(h) | 12 |
| ALK2(h) | 16 | PKCβI(h) | 12 |
| Rse(h) | 12 | GRK7(h) | 5 |
| WNK1(h) | 12 | MKK6(h) | 5 |
| CLK3(h) | 11 | PDHK4(h) | 5 |
| SGK(h) | 11 | ZAK(h) | 5 |
| SGK2(h) | 11 | MKK3(h) | 4 |
| SRMS(h) | 11 | MYO3B(h) | 4 |
| ACTR2(h) | 10 | PKG1β(h) | 4 |
| ALK6(h) | 10 | SAPK2b(h) | 4 |
| EphA1(h) | 10 | SLK(h) | 4 |
| PAK6(h) | 10 | PI3 Kinase (p120g)(h) | 4 |
| ROCK-I(h) | 10 | EphA5(h) | 3 |
| DNA-PK(h) | 10 | EphB3(h) | 3 |
| EphB4(h) | 9 | GRK2(h) | 3 |
| GRK1(h) | 9 | PEK(h) | 3 |
| PKG1α(h) | 9 | BrSK1(h) | 2 |
| RIPK1(h) | 9 | GRK3(h) | 2 |
| SAPK2a(h) | 9 | GRK6(h) | 2 |
| SGK3(h) | 9 | IRE1(h) | 2 |
| SRPK2(h) | 9 | LOK(h) | 2 |
| WNK3(h) | 9 | MATK(h) | 2 |
| BTK(h) | 8 | MRCKα(h) | 2 |
| CK1α(h) | 8 | CaMKIβ(h) | 1 |
| DMPK(h) | 8 | MAPKAP-K2(h) | 1 |
| FGFR4(h) | 8 | STK32C(h) | 1 |
| MEKK3(h) | 8 | ZAP-70(h) | 1 |
| mTOR/FKBP12(h) | 8 | PI3KC2a(h) | 1 |
| PKCι(h) | 8 | ChaK1(h) | 0 |
| Tec(h) activated | 8 | IGF-1R(h), activated | 0 |
| PI3 Kinase (p110b/p85a)(h) | 8 | IR(h) | 0 |
| IGF-1R(h) | 7 | NEK6(h) | 0 |
| PKCθ(h) | 7 | TTBK2(h) | 0 |
| STK25(h) | 7 | PI3 Kinase (p110d/p85a)(h) | 0 |
| ATM(h) | 7 | EphB2(h) | -1 |
| EphA4(h) | 6 | GRK5(h) | -1 |
| Plk3(h) | 6 | PIP4K2a(h) | -1 |
| DDR2(h) | -2 | Blk(h) | -5 |
| eEF-2K(h) | -2 | COT(h) | -5 |
| NEK7(h) | -2 | Plk1(h) | -5 |
| PRAK(h) | -2 | TTBK1(h) | -5 |
| SRPK1(h) | -2 | PIP5K1a(h) | -5 |
| TSSK2(h) | -2 | PI3KC2g(h) | -7 |
| PI3 Kinase (p110a/p85a)(h) | -2 | EGFR(h) | -10 |
| ErbB2(h) | -3 | MAPKAP-K3(h) | -10 |
| STK32A(h) | -3 | CSK(h) | -11 |
| STK32B(h) | -3 | PKBβ(h) | -12 |
| VRK2(h) | -3 | SNRK(h) | -14 |
| mTOR(h) | -4 | PKCζ(h) | -15 |
| NEK11(h) | -4 | ErbB4(h) | -16 |
| Ros(h) | -4 | MSSK1(h) | -19 |
| PIP5K1g(h) | -4 | CaMKIIα(h) | -25 |
| ASK1(h) | -5 |  |  |

**Original films of immunoblots**

**Figure 1**

**Figure 2**

**Figure 3**

**Figure 4**

**Figure 5**

**Figure 6**

**Fig S1**

**Fig S6**

**Fig S7**
